# Evaluation of common prescription analgesics and adjuvant analgesics as markers of suicide risk: a longitudinal population-based study in England

**DOI:** 10.1016/j.lanepe.2023.100695

**Published:** 2023-07-20

**Authors:** Danah Alothman, Edward Tyrrell, Sarah Lewis, Timothy Card, Andrew William Fogarty

**Affiliations:** School of Medicine, University of Nottingham, United Kingdom

**Keywords:** Analgesics, Pain, Suicide, Pregabalin, Gabapentin, Anticonvulsants

## Abstract

**Background:**

Analgesics prescriptions may provide a marker for identifying individuals at higher risk of suicide. In particular, awareness of which analgesics are implicated may help clinicians assess and modify risk.

**Method:**

A case–control study in England using the Clinical Practice Research Datalink (for primary care records) linked with hospital and national mortality electronic registries. We included patients aged ≥15 who died by suicide between 2001 and 2019 (N = 14,515), to whom we individually matched 580,159 controls by suicide date and general practice (N = 594,674). Odds ratios (ORs) for suicide, controlled for age and sex, were assessed using conditional logistic regression.

**Findings:**

Suicide risks were highest in those prescribed adjuvant analgesics (pregabalin, gabapentin and carbamazepine) (adjusted OR 4.07; 95% confidence intervals CI: 3.62–4.57), followed by those prescribed opioids (adjusted OR 2.01; 95% CI: 1.88–2.15) and those prescribed non-opioid analgesics (adjusted OR 1.48; 95% CI: 1.39–1.58) compared to those not prescribed these medications. By individual analgesic, the highest suicide risks were seen in patients prescribed oxycodone (adjusted OR 6.70; 95% CI: 4.49–9.37); pregabalin (adjusted OR 6.50; 95% CI: 5.41–7.81); morphine (adjusted OR 4.54; 95% CI: 3.73–5.52); and gabapentin (adjusted OR 3.12; 95% CI: 2.59–3.75). Suicide risk increased linearly with the number of analgesic prescriptions in the final year (p < 0.01 based on the likelihood ratio test), and the more different analgesics categories were prescribed in the final year (p < 0.01 based on the likelihood ratio test).

**Interpretation:**

Analgesic prescribing was associated with higher suicide risk. This is a particular issue with regard to adjuvant non-opiate analgesics.

**Funding:**

There was no funding for this study.

**Research in context**

**Evidence before this study**

We searched Pubmed, with no date restriction, for articles published in English using the following indexed terms: (analgesic∗ OR painkiller∗ OR “pain relie∗” OR opioid∗ OR nonopioid∗ OR “nonsteroidal anti-inflammatory∗” OR gabapentin∗ OR pregabalin∗) AND suicid∗. Relevant studies suggested that analgesics and suicide risk could be linked raising the viability of employing analgesics prescribing as markers for suicide risk.

**Added value of this study**

For the first time, a broad range of prescribed analgesics in primary care were analysed to identify patients at higher suicide risk. In this case–control study of 594,674 patients in England, there were significantly higher risks of suicide in those prescribed adjuvant analgesics (gabapentinoids and carbamazepine), opioid analgesics, and non-opioid analgesics who were more than 4 times, 2 times, and around 1.5 times more likely to die from suicide, compared to individuals not prescribed those drugs, respectively. There was a dose response association between suicide risk and the number of individual analgesic prescriptions as well as the number of different analgesics categories prescribed.

**Implications of all the available evidence**

Prescribed analgesics are a risk factor for suicide mortality. This can inform the evaluation and management of both chronic pain and suicide risk.

## Introduction

Identifying individuals at an increased risk of death by suicide helps in risk stratification and potential prevention. The hypothesis that receiving analgesic prescriptions is a potential indicator for suicide risk stems from several observations. Pain, irrespective of severity or the presence of psychiatric disorders, has been consistently documented to be a risk factor for suicide,^1^^,^^2^ but prescriptions of pain-killers as an additional marker for identifying patients at higher risk are yet to be assessed. Earlier studies suggest a link between access to analgesics and suicide risk, which further raises the viability of employing prescriptions of analgesics as indicators for suicide risk. For example, in the United Kingdom (UK), suicide rates were reduced after restricting the number of over-the-counter paracetamol pills per pack^3^ and in the United States (US), suicide rates increased with increases in exposure rates to over-the counter analgesics.^4^ Another important intervention in England during this study period was the withdrawal of dextropropoxyphene (an opioid) from the market, and this was associated with decreases in poisoning deaths and suicides by this drug with little observed change in deaths from other analgesics.^5^

In 2020, over 16,000 deaths in the USA involved opioid prescriptions (nearly 18% of all the opioid-related deaths in the USA).^6^ Though not all overdose deaths are attributable to suicide, up to one third may be.^7^ Ilgen et al. reported a dose-response relationship between prescriptions of opioids and suicide risk amongst US veteran patients^8^; nevertheless, very little is known about suicide risk in the wider population (and in relation to other types of prescribed analgesics). With a rise in opioid prescriptions in the UK,^9^^,^^10^ identifying patients at higher risk of suicide according to their opioid prescriptions could, thus, potentially serve as a life saving measure. Of further concern are the increasing trends in gabapentinoid prescriptions in both the USA^11^ and UK,^12^ prescribed as supposedly safer alternatives to opioid analgesics.^11^ However, there is a dearth of information on the relative risk of suicide recognised from gapapentinoid analgesics prescribing.

One important question is whether increasing the frequency of receipt of analgesic medication prescriptions reduces suicide risk (potentially by reducing morbidity from pain) or potentially increases suicide risk (by increasing analgesics availability to use as a poison).^13^^,^^14^ Yet a study of a wide range of analgesics as an indicator of suicide risk has so far not been conducted. To this end, we used a large population-based dataset to examine for the first time the association between a broad range of prescribed analgesics in primary care to quantify any subsequent at higher risks of suicide associated with the use of these medications.

## Methods

### Study design and settings

We conducted a case–control study in England between 2001 and 2019. Multiple integrated electronic health databases were used. Primary care records were derived from the Clinical Practice Research Datalink (CPRD), from both CPRD GOLD involving 22,003,009 patients in the UK as of 2020 and CPRD Aurum involving 48,062,524 patients in the UK as of 2020, with 6,768,901 patients concurrently occurring in GOLD and Aurum. Hence, in total there were 63,296,632 distinct patient records in CPRD databases at the time our data were extracted. The large CPRD database is considered representative of the UK in terms of age, sex and ethnicity and has been previously described in detail.^15^^,^^16^ The study included only patients whose data could be linked with Health Episode Statistics (HES) inpatient data for secondary care records and the Office for National Statistics (ONS) for cause of death to determine suicide. We also restricted our analysis to records meeting two quality criteria used within the CPRD: patients deemed to have acceptable records and general practices deemed to be up to standard (for CPRD GOLD only) in their collection of records.

### Study population

#### Cases

We included all CPRD patients linkable to ONS records who died of suicide (or open verdict) between 2001 and 2019 at age 15 years or above who fulfill the study's eligibility criteria. The inclusion of open verdict is recommended in studies of suicide as it improves suicide death misclassification error.^17^ Patients who had less than one year of complete records in CPRD prior to the suicide were excluded.

#### Controls

Controls were risk-set sampled from the cohort of patients fulfilling similar criteria to those for suicide cases. Of 63,296,632 patients in CPRD database, 22,339,028 (35.2%) were eligible for sampling in our study. For each suicide case, to maximise statistical efficiency, we individually and randomly selected up to 40 live controls from the same general practice as the cases matching on the date of suicide death (risk-set sampling). I.e., controls had to be alive and contributing data to CPRD at the date on which their matched case died by suicide. The date by which each control was matched to a case (the date of the matched case's suicide death) is henceforth referred to as the index date of that control individual. Of 14,515 suicide cases analysed in our study, 14,240 (98.1% of cases) had 40 matched controls and 275 (1.9%) had between 9 and 39 matched controls (where there were insufficient patients eligible to be matched). This study design was chosen as not only does it allow examining multiple hypotheses, but also given its risk-set nature, allows time-dependent covariates to be indirectly addressed through the sampling process and odds ratios (ORs) to be appropriately interpreted as rate ratios.^18^

### Outcome

Death from suicide was delineated from ONS cause of death records using the International Classification of Diseases 10th revision (ICD-10) codes X60-84, Y10-34 (excluding Y33.9), Y87.0, and Y87.2. The inclusion of ICD-10 codes referring to open verdicts (where the cause of the suspicious death was not specified by the coroner verdict) is recommended in studies of suicide as the majority of those deaths are suicide.^17^ The ONS is deemed as the gold standard for obtaining mortality statistics in the UK, including deaths caused by suicide.^19^

### Exposure

Any prescription of analgesics in primary care in the 12 months prior to suicide/index date for cases/controls was considered an exposure. We gathered cumulative data on all prescriptions of analgesics during the final year (before suicide/index date). Analgesics analysed in our study were collated from and categorised according to the British National Formulary 78th edition (BNF 78). We analysed three main groups of analgesics:

• non-opioid analgesics (including non-steroidal anti-inflammatory drugs)
• opioid analgesics (compound analgesics containing non-opioids and opioids were analysed in the opioid category only)
• adjuvant analgesics. These are the anti-convulsant medications pregabalin, gabapentin and carbamazepine commonly used as adjuvant treatment for neuropathic pain. Amitriptyline was not included here due to its frequent use for other indications, such as supporting sleep, in addition to its use for neuropathic pain. However, it was included in a sensitivity analysis.

Please refer to Supplementary Information 1 for detailed information on the list of analgesics analysed in this study and their categorisation. The codes used to identify analgesics prescribed in CPRD are also available in Supplementary Information 1.

The analysis included analgesics dispensed in oral, transdermal patch, and local (e.g., gel/cream) forms. Other forms, such as intravenous and mucosal analgesics were excluded as they are mainly used in palliative care and/or emergency situations whilst our aim is to reflect analgesics issued outside of these situations in routine practice in primary care. We also excluded antidepressants. In CPRD, prescribing data are automatically recorded to generate electronic prescriptions; hence CPRD can offer a comprehensive dataset with well-documented prescribing information.^20^^,^^21^

### Covariates

Sex and age at suicide death for cases and at index date for controls were considered as *a priori* confounders. Known psychiatric morbidities that were diagnosed by clinicians (whether in CPRD or HES) were considered a covariate in our study. These include the following categories: affective disorders including depressive disorders; schizophrenia spectrum and other psychosis; anxiety disorders including generalised anxiety disorder and obsessive compulsive disorders; personality disorders; eating disorders; sleep disorders including insomnia; and substance misuse disorders including alcohol and illegal substance consumption. Please refer to Supplementary Information 2 for the code lists of all psychiatric illnesses analysed in our study.

### Analysis

We determined the top ten analgesics by proportion of cases and controls who had at least one prescription of them in the previous year (i.e., greatest prescription prevalence). We also determined the top ten analgesics by total prescriptions issued in the previous year (i.e., most prescriptions).

Conditional logistic regression was used to estimate ORs. We developed two multivariable models. In the first model (our main model), age and sex were considered a *priori* confounders and age was fitted into four quantiles guided by the results of the likelihood ratio test (LRT). In the second model, further control for the presence of psychiatric illnesses was carried out fitting psychiatric illnesses categorically. Psychiatric conditions, a strong risk factor for suicide,^22^ can predate pain conditions increasing use of analgesic medications (thereby acting as a potential confounder in the association between analgesic medications and suicide risk); can occur secondary to pain conditions (thereby acting as a potential mediator); and can possibly hold a bidirectional relationship with pain.^23^, ^24^, ^25^ In awareness of psychiatric illnesses to likely be lying in the causal pathway between analgesic prescribing and suicide risk, we considered the fully adjusted model (adjusted for age, sex, and psychiatric conditions) as the subsidiary model and controlled for psychiatric illness to explore whether any of the effects persist beyond the co-presence of psychiatric conditions. We used a LRT test to assess the linearity of the association between suicide risk and the number of different categories of prescribed analgesic and the frequency of receipt of prescriptions in the final year, and effect modification by age and sex were tested using the LRT by fitting an interaction term. For a sense of temporal proximity, we also assessed the risk of suicide across the main prescribed analgesic categories during the final three months before suicide/index date.

To assess the public health significance of commonly prescribed analgesics in relation to suicide risk, we estimated population attributable fractions (PAFs). As suicide is a relatively uncommon outcome, ORs were used as appropriate surrogates for relative risk in the Miettinen PAF formula.^26^^,^^27^ We estimated PAFs in all three main analgesic categories using fully adjusted ORs (controlled for age, sex and psychiatric illnesses).

A post-hoc analysis was performed to explore whether changes in analgesic prescribing patterns influenced the risk of suicide, particularly that by poisoning. In the post-hoc analysis, suicide cases were stratified into poisoning (by any substance) versus non-poisoning suicide using ICD-10 codes in ONS which generally specifies the method of suicide, and the risk of suicide was assessed across the main analgesic categories before and after 2016. For more details on this analysis, please refer to Supplementary Information 3. We received approval for our study from the MHRA Independent Scientific Advisory Committee (reference number 20_186RA). For reporting our study, we conformed with the Observational Routinely Collected Data (RECORD) guidelines.^28^ There was no direct funding for this study and the analysis was done as part of a PhD thesis.

### Sensitivity analyses

The adjuvant analgesics analysed can be prescribed for indications other than pain, although in the UK 94% of recorded indications for gabapentinoids are for pain.^29^ We therefore conducted a sensitivity analysis excluding all subjects in either CPRD or HES with diagnoses of epilepsy, bipolar mood disorder and generalised anxiety disorder which are other common indications for adjuvant medications (pregabalin can be prescribed for generalised anxiety disorder). Given that amitriptyline can be prescribed for pain, but is a type of antidepressant unlike the anticonvulsant group of adjuvant analgesics, another sensitivity analysis was conducted to explore the effect of amitriptyline inclusion to the adjuvant analgesic category. Substance misuse disorders including alcoholism were adjusted for in our analysis as part of our adjustment for known psychiatric conditions. When tested in the regression model, both the index of multiple deprivation (used as a proxy for socioeconomic status) and ethnicity did not change the effect of interest by more than 10% (hence did not demonstrate significant confounding effect) and as such were not included in the multivariable model. However, we did not examine the effect of body mass index on the association between analgesics prescribing and suicide risk as we lacked the data.

## Results

There were 594,674 individuals who provided data for the analysis of whom 14,515 (2.4%) were suicide cases and 580,159 (97.6%) were live controls. Of the 14,515 patients who died of suicide, 10,850 (74.8%) were males and 3665 (25.3%) were females. Of the 580,159 (97.6%) control patients, 289,769 (50.0%) were males and 290,390 (50.1%) were females. The median age at suicide death was significantly lower (47.7; interquartile range 36.0–59.7) than the median age at death for controls (who were alive at the date of matching but died from other causes at a later point) (81.6; interquartile range 71.95–88.37, p < 0.001). 86.9% of suicide cases and 89.9% of controls were not prescribed any analgesics in the final year. In the year before the suicide/index date, non-opioid analgesics comprised the highest proportion of an analgesic ever prescribed amongst both cases and controls followed by opioid analgesics then adjuvant analgesics (Table 1). The top four analgesics by prescription prevalence were the same for both cases and controls which were (in descending order): paracetamol, co-codamol (a compound analgesic comprised of paracetamol and codeine), ibuprofen and diclofenac. In terms of total prescriptions, paracetamol and co-codamol were the most prescribed analgesics for cases and controls. Pregabalin (by prescription prevalence) and oxycodone and morphine (by total prescriptions) were amongst the ten most commonly prescribed analgesics for suicide cases but not for controls.

**Table 1:** Analgesics commonly prescribed in primary care.

	Cases N = 14,515 (2.4%)	Control N = 580,159 (97.6%)
Not prescribed any analgesics in the final year	12,620 (86.94%)	521,103 (89.82%)
Analgesics Category (descending order)		
	Non-opioids 1288 (8.87%)	Non-opioids 44,391 (7.65%)
	Opioids 1132 (7.80%)	Opioids 30,440 (5.25%)
	Adjuvant 341 (2.35%) (141 prescriptions for pregabalin; 127 for gabapentin; and 93 for carbamazepine)	Adjuvant 4037 (0.70%) (1062 prescriptions for pregabalin; 1969 for gabapentin; and 1246 for carbamazepine)
Top ten analgesics by prescription prevalence^a^ (descending order)		
	Paracetamol 575 (3.96%)	Paracetamol 17,669 (3.05%)
	Co-codamol (paracetamol & codeine) 473 (3.26%)	Co-codamol (paracetamol & codeine) 14,887 (2.57%)
	Ibuprofen 345 (2.38%)	Ibuprofen 12,013 (2.07%)
	Diclofenac 326 (2.25%)	Diclofenac 11,248 (1.94%)
	Tramadol 283 (1.95%)	Naproxen 8074 (1.39%)
	Codeine 253 (1.74%)	Codeine 7035 (1.21%)
	Naproxen 198 (1.36%)	Tramadol 5646 (0.97%)
	Co-dydramol 171 (1.18%)	Co-dydramol 5112 (0.88%)
	Pregabalin 141 (0.97%)	Gabapentin 1969 (0.34%)
	Gabapentin 127 (0.87%)	Dihydrocodeine 1798 (0.31%)
Top ten analgesics by total prescriptions^b^ (per 100 person-year) (descending order)		
	Paracetamol 3146 prescriptions (21.67 per 100 person-year)	Paracetamol 88,490 prescriptions (15.25 per 100 person-year)
	Co-codamol (paracetamol & codeine) 2272 prescriptions (15.65 per 100 person-year)	Co-codamol (paracetamol & codeine) 56,194 prescriptions (9.69 per 100 person-year)
	Pregabalin 1970 prescriptions (13.57 per 100 person-year)	Ibuprofen 28,439 prescriptions (4.90 per 100 person-year)
	Tramadol 1800 (12.40 per 100 person-year)	Tramadol 28,309 (4.88 per 100 person-year)
	Dihydrocodeine 1388 prescriptions (9.56 per 100 person-year)	Diclofenac 27,226 prescriptions (4.69 per 100 person-year)
	Gabapentin 1282 prescriptions (8.83 per 100 person-year)	Codeine 25,353 prescriptions (4.37 per 100 person-year)
	Oxycodone 1270 (8.75 per 100 person-year)	Naproxen 19,098 (3.29 per 100 person-year)
	Codeine phosphate 1245 (8.58 per 100 person-year)	Co-dydramol 17,830 (3.07 per 100 person-year)
	Morphine 1120 (7.71 per 100 person-year)	Gabapentin 12,668 (2.18 per 100 person-year)
	Ibuprofen 993 (6.84 per 100 person-year)	Carbamazepine 10,701 (1.84 per 100 person-year)

a Those analgesics were determined according to the proportion of cases and controls in the sample ever prescribed those analgesics in the year leading to suicide.

b Those analgesics were determined based on all the total prescription issued in the year leading to suicide.

As seen in Table 2, the OR of suicide adjusted for the effect of age and sex was increased amongst all categories of patients prescribed analgesics; the adjusted OR was highest in those prescribed adjuvant analgesics (OR 4.07, 95% confidence interval; CI: 3.62–4.57) followed by those prescribed opioids (OR 2.01; 95% CI: 1.88–2.15) then by those prescribed non-opioids (OR 1.48; 95% CI: 1.39–1.58). Controlling for psychiatric illnesses in addition to the original model attenuated suicide risk in all three analgesics categories, but the risk remained significantly elevated in those prescribed adjuvant analgesics (fully adjusted OR 2.10; 95% CI: 1.86–2.37) and opioids (fully adjusted OR 1.21; 95% CI: 1.13–1.30). There was a dose-response relationship between the number of different categories of prescribed analgesics and the risk of suicide (p < 0.001 for linearity). Patients prescribed three different categories of analgesics in the year leading to suicide were 4.74 (95% CI: 3.91–5.75) times more likely to die from suicide, after adjusting for age and sex, and 2.02 (95% CI: 1.66–2.47) times more likely to die from suicide after further control for psychiatric illnesses than those not prescribed analgesics in the year leading to suicide.

**Table 2:** Odds ratios for suicide risk in relation to analgesics prescriptions.

	Unadjusted odds ratios (95% CI)	Adjusted odds ratios^a^ (95% CI)	Fully adjusted odds ratios^b^ (95% CI)
Analgesics Category			
Opioids	1.62 (1.52–1.73)	2.01 (1.88–2.15)	1.21 (1.13–1.30)
Non-opioids	1.21 (1.13–1.29)	1.48 (1.39–1.58)	1.06 (0.99–1.13)
Adjuvant	3.60 (3.20–4.03)	4.07 (3.62–4.57)	2.10 (1.86–2.37)
Number of analgesic categories prescribed in previous year			
0 categories	1	1	1
1 category	1.26 (1.18–1.35)	1.53 (1.43–1.64)	1.08 (1.00–1.15)
2 categories	1.66 (1.53–1.82)	2.18 (1.99–2.38)	1.24 (1.14–1.36)
3 categories	3.63 (3.00–4.38)	4.74 (3.91–5.75)	2.02 (1.66–2.47)
p value for linearity based on the LRT	<0.001	<0.001	<0.001
Commonly prescribed analgesics^c^			
Oxycodone	5.72 (4.11–7.95)	6.70 (4.79–9.37)	2.98 (2.09–4.24)
Pregabalin	5.57 (4.65–6.67)	6.50 (5.41–7.81)	3.12 (2.57–3.78)
Morphine	3.72 (3.07–4.51)	4.54 (3.73–5.52)	2.35 (1.92–2.89)
Gabapentin	2.64 (2.20–3.17)	3.12 (2.59–3.75)	1.64 (1.36–1.99)
Dihydrocodeine	2.70 (2.24–3.27)	3.03 (2.50–3.67)	1.59 (1.30–1.93)
Tramadol	2.07 (1.83–2.34)	2.47 (2.18–2.80)	1.35 (1.19–1.54)
Paracetamol	1.34 (1.23–1.46)	1.87 (1.70–2.05)	1.25 (1.14–1.37)
Codeine	1.47 (1.29–1.68)	1.79 (1.57–2.04)	1.17 (1.02–1.34)
Co-dydramol	1.35 (1.16–1.58)	1.65 (1.41–1.93)	1.10 (0.94–1.30)
Co-codamol	1.30 (1.18–1.43)	1.60 (1.45–1.76)	1.03 (0.93–1.14)
Ibuprofen	1.16 (1.04–1.30)	1.43 (1.28–1.60)	1.07 (0.95–1.20)
Diclofenac	1.17 (1.04–1.31)	1.22 (1.09–1.37)	0.98 (0.88–1.11)
Naproxen	0.98 (0.85–1.13)	1.03 (0.89–1.19)	0.79 (0.68–0.92)

LRT indicates the likelihood ratio test.

a Multivariable model adjusted for sex and age (at suicide death date for cases and index date for controls).

b Multivariable model adjusted for sex and age (at suicide death date for cases and index date for controls) and psychiatric diagnoses.

c Those refer to commonly prescribed analgesics whether by proportion of cases and controls ever prescribed those analgesics in the year leading to suicide or by total prescriptions issued in the year leading to suicide.

Amongst commonly prescribed individual analgesics, the highest suicide risk adjusted for age and sex was observed in those prescribed oxycodone (adjusted OR 6.70; 95% CI: 4.49–9.37); pregabalin (adjusted OR 6.50, 95% CI: 5.41–7.81); morphine (adjusted OR 4.54; 95% CI: 3.73–5.52); gabapentin (adjusted OR 3.12, 95% CI: 2.59–3.75); and dihydrocodeine (adjusted OR 3.03; 95% CI: 2.50–3.67) compared to patients who were not prescribed these medications. Further adjustment for psychiatric illnesses attenuated the risk, but the risk remained significantly elevated in patients prescribed those medications; fully adjusted OR was 2.98 (95% CI: 2.09–4.24) for oxycodone; 3.12 (95% CI: 2.57–3.78) for pregabalin, 2.35 (95% CI: 1.92–2.89) for morphine; 1.64 (95% CI: 1.36–1.99) for gabapentin; and 1.59 (95% CI: 1.30–1.93) for dihydrocodeine.

**Fig. 1:**
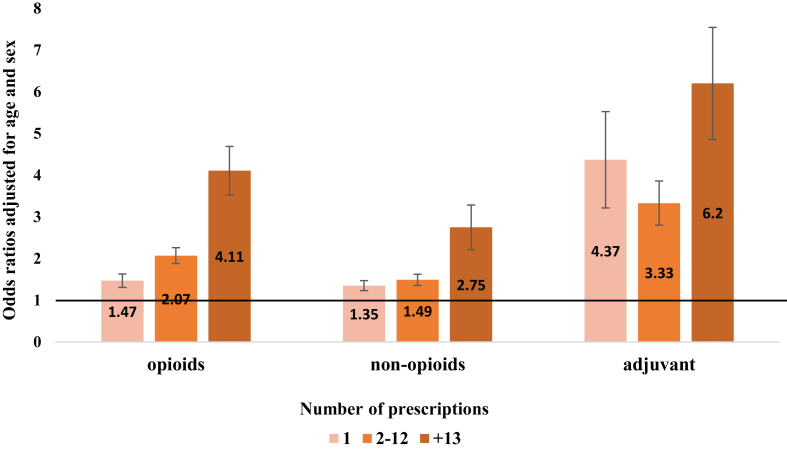
Odds ratios of suicide in relation to the number of prescriptions across the three main analgesic categories referenced to no prescriptions in the final year. Note: error bars represent the 95% confidence intervals. Likelihood ratio test suggested a linear association between prescriptions of analgesics and suicide risk. Increasing by 1.51 times per additional opioid analgesic prescription (p = 0.003). Increasing by 1.28 times per additional nonopioid analgesic prescription (p < 0.0001). Increasing by 1.87 times per additional adjuvant analgesic prescription (p < 0.0001).

Fig. 1 depicts the OR of suicide by frequency of the receipt of analgesic prescriptions. Across all analgesic categories, the higher the number of prescriptions, the higher was the risk (p value for linearity <0.01). Suicide risk was highest amongst those prescribed ≥13 times in the final year (equating to more than once per month) across all three analgesics categories: opioid analgesics (adjusted ORs 4.11; 95% CI: 3.57–4.73); non-opioid analgesics (adjusted ORs 2.75; 95% CI 2.27–3.34); and adjuvant analgesics (adjusted ORs 6.20; 95% CI: 5.00–7.69).

There was no significant modification of effect by sex for the analgesic categories apart from that related to non-opioids (p = 0.005). Age was a significant modifier of effect across all three categories of analgesics (p < 0.05). As demonstrated in Table 3, after adjusting for sex and psychiatric disorders, suicide risk was elevated with statistical significance in patients prescribed opioids aged 35 to <55 years (fully adjusted OR 1.35; 95% CI: 1.20–1.52) and those aged 55 years or over (fully adjusted OR 1.17; 95% CI: 1.05–1.31) compared to their counterparts who were not prescribed opioid analgesics. Suicide risk adjusted for sex and psychiatric illnesses was elevated across all age groups in patients prescribed adjuvant analgesics and was highest amongst patients aged 35 to <55 years (fully adjusted OR 2.47; 95% CI: 2.01–3.04) and patients aged 15 to <35 years (fully adjusted OR 2.12; 95% CI: 1.23–3.66) compared to their counterparts who were not prescribed adjuvant analgesics. A focused examination of the risk of suicide across the main prescribed analgesics in the final three months leading up to suicide/index date showed that the risk, adjusted for age and sex, was 2.24 (95% CI: 2.07–2.43) in those prescribed opioid analgesics, 1.78 (95% CI: 1.57–1.97) in those prescribed non-opioid analgesics and 4.05 (95% CI: 3.54–4.62) in those prescribed adjuvant analgesics.

**Table 3:** Odds ratios for analgesics categories across age groups with no analgesics prescribed as the reference group along with the corresponding estimated population attributable fractions.

Analgesic category, years	Unadjusted Odds ratios (95% CI)	Adjusted Odds ratios^a^ (95% CI)	Fully adjusted Odds ratios^b^ (95% CI)	% PAF (95% CI)
**Opioids**				1.4 (0.83–1.9)
15 to <35	1.71 (1.39–2.12)	2.07 (1.67–2.57)	1.12 (0.90–1.40)	
35 to <55	2.17 (1.95–2.41)	2.50 (2.25–2.80)	1.35 (1.20–1.52)	
+55	1.51 (1.36–1.68)	1.68 (1.51–1.87)	1.17 (1.05–1.31)	
**Non-opioids**				0.52 (0.053–1.1)
15 to <35	1.31 (1.09–1.57)	1.51 (1.26–1.82)	0.99 (0.82–1.21)	
35 to <55	1.44 (1.30–1.60)	1.66 (1.49–1.84)	1.10 (0.98–1.23)	
+55	1.18 (1.07–1.30)	1.29 (1.17–1.43)	1.04 (0.94–1.16)	
**Adjuvant**				1.2 (0.97–1.5)
15 to <35	4.68 (3.06–7.16)	4.99 (3.16–7.89)	2.12 (1.23–3.66)	
35 to <55	4.61 (3.85–5.51)	5.17 (4.30–6.22)	2.47 (2.01–3.04)	
+55	3.03 (2.51–3.66)	3.26 (2.68–3.96)	1.96 (1.59–2.41)	

PAF: Population attributable fraction. This was estimated using odds ratios adjusted for age, sex and psychiatric illnesses.

Note: p-value for effect modification by age-groups was <0.05 across all three analgesics categories based on results from the likelihood ratio test for interaction terms.

a Multivariable model adjusted for sex.

b Multivariable model adjusted for sex and psychiatric diagnoses.

To provide an estimate of the potential public health importance of these observations, PAFs were calculated for the impact of opiate analgesics, non-opiate analgesics and adjuvant analgesics. These gave values of 1.4%, 0.53% and 1.2% for the risk of mortality from suicide in the whole population, respectively.

### *Post-hoc* analysis

Across all analgesic categories, and across the two epochs (from 2001 to 2016 and from 2016 to 2019), the risk of suicide, controlled for age and sex, was particularly increased in relation to suicide by any substance poisoning. Over time, a large increase in the risk of poisoning suicide using any substance was noted in relation to gabapentinoids prescribing, from an adjusted OR of 6.63 (95% CI: 5.38–8.18, between 2001 and 2016) to 10.11 (95% CI: 7.16–14.28, between 2016 and 2019). Across the same epochs, the increase in the risk of any-substance poisoning suicide in relation to opioids prescribing was not as high, increasing from 3.82 (95% CI: 3.40–4.31, between 2001 and 2016) to 4.12 (95% CI: 3.06–5.54, between 2016 and 2019).

### Sensitivity analyses

After excluding all patients with diagnoses of epilepsy, bipolar mood disorder and/or generalised anxiety disoder, fully adjusted suicide odds pertaining to adjuvant analgesics remained relatively consistent (sensitivity analysis 1). Moreover, the inclusion of amitriptyline to adjuvant analgesics did not lead to significant changes in results related to suicide risk (sensitivity analysis 2). Amitriptyline, nevertheless, was associated with 1.62 times (95% CI: 1.46–1.80) increased odds of suicide risk, after adjusting for sex, age and psychiatric illnesses. Please refer to Supplementary Information 4 for further information on the sensitivity analyses.

## Discussion

This is the first study to examine a broad range of prescribed analgesics in primary care in England as indicators of suicide risk. While our study does not imply causality between analgesics prescribing and suicide risk, it showed that being prescribed analgesics can be a valuable marker to identify (and potentially prevent) patients at higher risk of suicide. Patients prescribed non-opioid analgesics, opioid analgesics and adjuvant analgesics were all found to be at a significantly increased risk of suicide compared to those who were not prescribed those medications, with those prescribed adjuvant analgesics demonstrating the highest risk. Markers of highest risk included more categories of analgesics prescribing; frequent prescribing (≥13 times in final year) of any category of analgesics; Oxycodone, pregabalin and morphine prescribing; and opioid and adjuvant analgesics prescribing in patients below the age of 55. Adjustment for psychiatric illnesses attenuated the risk of suicide in all three analgesics categories, but prescriptions of opioid analgesics and adjuvant analgesics were significant markers of suicide risk above and beyond the confounding effect of psychiatric illnesses.

The study's strengths are derived from its large population-based sample allowing the examination, for the first time, of suicide risk in relation to a broad range of prescribed analgesics. The population-based nature of the sample in addition means our results are generalisable to England's population. Equally importantly, the use of routinely collected data that was collated with no awareness of the hypothesis of interest, minimises the risk of bias in recording. The use of nationally mandated ONS death recording to define suicide as an outcome measure will ensure a high level of specificity in recording.

However, we acknowledge that there are several limitations in this study. Firstly, we have looked at commonly prescribed analgesics in primary care as markers for increased suicide risk regardless of indication and have not established reasons that could link analgesics to suicide risk. A particular consideration of importance is potential confounding by indication for pain of other causes, as our data allow confidence that the medication was prescribed along with a timestamp, but no details as to what the underlying indication was. Given that adjuvant analgesics analysed in our study have other indications, particularly that for epilepsy and bipolar mood disorder (and generalised anxiety disorder for pregabalin) there is the risk of also confounding by those indications. Nonetheless, the considerable robustness of results demonstrated in our sensitivity analysis, coupled with the fact that 94% of recorded indications for gabapentinoids in the UK are for pain,^29^ suggest this is unlikely. Secondly, while we were able to analyse the prescription of analgesics by primary care physicians, we were neither able to analyse analgesic medications that have been obtained from over-the-counter retail outlets or other undocumented sources nor were we able to study the actual consumption of these medications. Nevertheless, examining recorded analgesic prescribing is potentially more relevant to the prescribing information available to a primary care clinician at the time of consulting to identify at-risk patients. Another important limitation is that we have not included prescription-related information such as dosage of analgesics and duration, nor indications for those prescriptions. These are areas of high priority for future research to explore. Adjusting for a prior diagnosis of a psychiatric disease is potentially going to be modified by access to healthcare, socioeconomic status and ethnicity (as mental illness can be considered a stigma in some sectors of society). This is another limitation in our study.

Our results are in line with previous work demonstrating increased suicide risk amongst patients prescribed opioids.^8^ Although we did not examine mechanisms by which analgesics might be related to suicide risk, we hypothesize two broad ones. Firstly, availability of analgesics providing a potentially lethal poison for suicide and secondly medical conditions resulting in pain increase the risk of suicide. Other potential reasons may include the complex interplay between pain, analgesia and suicide risk. For example, there is growing evidence of neural overlap between physical pain and psychological pain^30^^,^^31^ (defined as a lasting, unpleasant and unsustainable feeling^32^), of which the latter too has been associated with increased risk of suicide.^31^^,^^33^ As psychological pain was reported to be modulated by opioid and cannabinoid receptors,^34^ analgesics prescribed in primary care may hypothetically be a marker of help-seeking behaviour in some patients with psychological pain (or pre-existing undiagnosed psychiatric morbidities) camouflaged as physical pain, for which they may be seeking relief via “pain killers”. This may be noteworthy for clinicians when assessing patients’ complaints of pain, and a more thorough examination is required to both screen for psychiatric conditions as well as to understand the source of that pain, as outlined in current National Institute for Health and Care Excellence guidance on chronic pain.^35^

Additional factors proposed by the integrated motivational–volitional model of suicidal behaviour^36^ could explain the relationship between analgesics prescribing and suicide risk. In the final phase of suicidal behaviour, discusses the model, acquired capability for suicide in the form of fearlessness about death and increased physical pain endurance, can govern the transition from suicidal ideation to suicide attempt/death; by numbing pain, analgesics may play a role in increasing pain tolerance thereby easing fatal self-harm activities.

The proportional increase in suicide risk with increased number of analgesic prescriptions as well as with more types of analgesics prescribing may reflect a subgroup of patients suffering from more severe and chronic pain or those receiving suboptimal pain management, or perhaps both. Frequent prescribing of analgesics and prescribing of multiple different categories of analgesics may suggest poor access to other non-pharmacological treatment modalities in which clinicians, when faced with uncontrolled and chronic pain, may be inclined to prescribe more and different types of analgesics rather than using effective evidence-based non-pharmacological treatments (such as physiotherapy and cognitive behavioural therapy^37^^,^^38^).

The higher suicide risk associated with the number of analgesic prescriptions recorded and a greater number of categories of analgesics prescribed may simply be a marker of greater access to medications precipitating suicide by overdose, highlighting the need for clinicians to be mindful of a patient's access to potentially lethal overdoses of prescribed medication when making prescribing decisions. Although our analysis of poisoning suicide was in relation to any substance and not specific to the analgesic category under question, the larger increase in suicide by poisoning related to gabapentinoid analgesic prescriptions during the two epochs compared to opioid analgesic prescriptions, may suggest a potential shift in suicide risk from one analgesic category to another, corresponding possibly to the relative change in analgesics prescribing patterns. While a recent systematic review emphasised the effective role of limiting the quantity of medications supplied in reducing suicide by medicine overdose (without an equivalent shift in other suicide-specific methods),^39^ new findings showed that stopping opioid analgesics were associated with increased suicide risk.^40^ It appears that there is a fine line between excessive access to analgesic medications and unnecessary endurance of pain warranting further research.

There has been a sharp increase in recent years in the prescription of gabapentinoids.^11^^,^^12^ With an increase in the trend of using those medications for neuropathic pain as an alternative to opioids, our results together with previous findings raise concern. Previous small studies have shown mixed results relating to suicide risk and anticonvulsants, including gabapentinoids.^41^^,^^42^ However, a more recent, population-based cohort study in Sweden reported elevated risk of suicidal behaviour and death in patients prescribed gabapentoids with the risk being strongest in those prescribed pregabalin over gabapentin and in younger age groups.^43^ This is consistent with our data in an English population. Although the reasons by which adjuvant analgesics may be linked to suicide risk are unclear,^44^ clinicians are recommended to assess patients for psychiatric morbidities and suicide risk both prior to and during treatment with these medications.

In summary, prescriptions of analgesic medication could be an important marker of suicide risk recognition and hence a potential point for intervention. A pain management approach that involves careful consideration of patients’ complaints; optimisation of pain control (through the integration of effective non-pharmacological pain management); psychiatric conditions screening and suicide risk assessment may help mitigate the increased risk of suicide identified in patients prescribed analgesics. Our data raise concerns that as gabapentinoids are being used to reduce opiate prescribing in the community, the risks associated with the use of strong analgesics is simply being transferred from one category of medication to another.

## Contributors

Concept and design: Alothman, Lewis, Card, Fogarty.

Data access and curation: Alothman and Card.

Statistical analysis: Alothman.

Review of statistical analysis: Fogarty.

Acquisition, analysis, or interpretation of data: Alothman, Tyrrell, Lewis, Card, Fogarty.

Drafting of the manuscript: Alothman.

Critical revision of the manuscript: Tyrrell, Lewis, Card, Fogarty.

Supervision: Tyrrell, Lewis, Card, Fogarty.

## Data sharing statement

The data used for this work were obtained under license from CPRD. This license does not permit further sharing. However, anyone wishing to access the data can obtain it direct from CPRD subject to their licensing requirements.

## Declaration of interests

On behalf of all authors, the corresponding author states that there is no conflict of interest.
