# Supplementary material for: Evaluation of common prescription analgesics and adjuvant analgesics as markers of suicide risk: a longitudinal population-based study in England
Source: Lancet Reg Health Eur. 2023 Jul 20;32:100695. doi: 10.1016/j.lanepe.2023.100695 (PMC10393825; doi:10.1016/j.lanepe.2023.100695)
Supplement: Supplementary Information 1 [file mmc1.docx]

## Supplementary information 1

Analgesics analysed in our study were collated from and categorised according to the British National Formulary 78^th^ edition (BNF 78). We analysed three main groups of analgesics: non-opioid analgesics, opioid analgesics and adjuvant analgesics.

Non-opioid

For non-opioid analgesics, we delineated medications falling under chapter 4 section 6 for treatment of pain (i.e., section 4.6), specifically under the subheading non-opioid analgesics. Non-steroidal anti-inflammatory drugs mentioned under section 10.4 for pain and inflammation in musculoskeletal disorders were added to the non-opioid analgesics category.

Opioid analgesics:

For opioid analgesics, we analysed those medications mentioned under section 4.6 for pain, specifically under the subheading opioid analgesics.

Adjuvant analgesics

We analysed three medications commonly known to be prescribed for neuropathic pain as adjuvant analgesics: pregabalin, gabapentin and carbamazepine. These are anti-convulsant medications listed under section 4.6.2 for neuropathic pain. We focused our analysis on these medications and precluded other types (e.g. anti-depressants, such as amitriptyline and corticosteroids) as the other types have a broader range of indications which they are commonly used for as well as being from different classes of medications (hence, different mechanisms of action).

Table S1 below shows all analgesics analysed in our study and their categories. Table S2 and Table S3 lists all analgesics analysed in our study and their codes for CPRD GOLD and CPRD Aurum, respectively.

Table S1:List of analgesics and their categories analysed in our study.

| **Analgesic name** | **Category** |
| --- | --- |
| Paracetamol | Non-Opioid |
| Nefopam hydrochloride | Non-Opioid |
| Paracetamol with isometheptene | Non-Opioid |
| Paracetamol with metoclopramide | Non-Opioid |
| Tolfenamic acid | Non-Opioid |
| Aceclofenac | Non-Opioid |
| Celecoxib | Non-Opioid |
| Dexibuprofen | Non-Opioid |
| Dexketoprofen | Non-Opioid |
| Diclofenac potassium | Non-Opioid |
| Diclofenac sodium | Non-Opioid |
| Etodolac | Non-Opioid |
| Etoricoxib | Non-Opioid |
| Felbinac | Non-Opioid |
| Flurbiprofen | Non-Opioid |
| Ibuprofen | Non-Opioid |
| Indometacin | Non-Opioid |
| Ketoprofen | Non-Opioid |
| Mefenamic acid | Non-Opioid |
| Meloxicam | Non-Opioid |
| Nabumetone | Non-Opioid |
| Naproxen | Non-Opioid |
| Piroxicam | Non-Opioid |
| Sulindac | Non-Opioid |
| Tenoxicam | Non-Opioid |
| Tiaprofenic acid | Non-Opioid |
| Diclofenac sodium with misoprostol | Non-Opioid |
| Naproxen with esomeprazole | Non-Opioid |
| Codeine phosphate | Opioid |
| Diamorphine hydrochloride | Opioid |
| Dihydrocodeine tartrate | Opioid |
| Fentanyl | Opioid |
| Hydromorphone hydrochloride | Opioid |
| Meptazinol | Opioid |
| Morphine | Opioid |
| Oxycodone hydrochloride | Opioid |
| Papaveretum | Opioid |
| Pentazocine | Opioid |
| Pethidine hydrochloride | Opioid |
| Sufentanil | Opioid |
| Tapentadol | Opioid |
| Tramadol hydrochloride | Opioid |
| Buprenorphine | Opioid |
| Co-codaprin | Opioid |
| Co-codamol | Opioid |
| Co-dydramol | Opioid |
| Dipipanone hydrochloride with cyclizine | Opioid |
| Morphine with cyclizine | Opioid |
| Oxycodone with naloxone | Opioid |
| Tramadol with dexketoprofen | Opioid |
| Tramadol with paracetamol | Opioid |
| Paracetamol with buclizine hydrochloride and codeine phosphate | Opioid |
| Gabapentin | Adjuvant |
| pregabalin | Adjuvant |
| Carbamazepine | Adjuvant |

Table S2: Codes used for identifying analgesics prescribed in CPRD GOLD.

| Analgesics | Product code |
| --- | --- |
| Aceclofenac | 9474 |
| Aceclofenac | 526 |
| Buprenorphine | 67356 |
| Buprenorphine | 5936 |
| Buprenorphine | 68889 |
| Buprenorphine | 66470 |
| Buprenorphine | 74562 |
| Buprenorphine | 6917 |
| Buprenorphine | 68241 |
| Buprenorphine | 72160 |
| Buprenorphine | 67901 |
| Buprenorphine | 6040 |
| Buprenorphine | 78845 |
| Buprenorphine | 69254 |
| Buprenorphine | 68890 |
| Buprenorphine | 69243 |
| Buprenorphine | 71310 |
| Buprenorphine | 78477 |
| Buprenorphine | 68472 |
| Buprenorphine | 70124 |
| Buprenorphine | 68848 |
| Buprenorphine | 68402 |
| Buprenorphine | 69315 |
| Buprenorphine | 7555 |
| Buprenorphine | 70461 |
| Buprenorphine | 73406 |
| Buprenorphine | 59392 |
| Buprenorphine | 69795 |
| Buprenorphine | 66280 |
| Buprenorphine | 13300 |
| Buprenorphine | 68172 |
| Buprenorphine | 56671 |
| Buprenorphine | 72098 |
| Buprenorphine | 78142 |
| Buprenorphine | 75334 |
| Buprenorphine | 68743 |
| Buprenorphine | 68888 |
| Buprenorphine | 70139 |
| Buprenorphine | 70631 |
| Buprenorphine | 68479 |
| Buprenorphine | 60170 |
| Buprenorphine | 10205 |
| Buprenorphine | 68167 |
| Buprenorphine | 68559 |
| Buprenorphine | 73545 |
| Buprenorphine | 59618 |
| Buprenorphine | 67018 |
| Buprenorphine | 66463 |
| Buprenorphine | 68196 |
| Buprenorphine | 59146 |
| Buprenorphine | 7236 |
| Buprenorphine | 70117 |
| Buprenorphine | 59473 |
| Buprenorphine | 7334 |
| Buprenorphine | 60943 |
| Buprenorphine | 54806 |
| Buprenorphine | 71711 |
| Buprenorphine | 58766 |
| Buprenorphine | 6879 |
| Buprenorphine | 78750 |
| Buprenorphine | 66689 |
| Buprenorphine | 66695 |
| Buprenorphine | 7238 |
| Buprenorphine | 70460 |
| Buprenorphine | 74337 |
| Buprenorphine | 6181 |
| Buprenorphine | 11584 |
| Capsaicin | 33500 |
| Carbamazepine | 45941 |
| Carbamazepine | 76453 |
| Carbamazepine | 66058 |
| Carbamazepine | 75692 |
| Carbamazepine | 48397 |
| Carbamazepine | 3569 |
| Carbamazepine | 70901 |
| Carbamazepine | 40404 |
| Carbamazepine | 60133 |
| Carbamazepine | 43417 |
| Carbamazepine | 57898 |
| Carbamazepine | 2388 |
| Carbamazepine | 59887 |
| Carbamazepine | 34370 |
| Carbamazepine | 18763 |
| Carbamazepine | 13524 |
| Carbamazepine | 52840 |
| Carbamazepine | 66073 |
| Carbamazepine | 70902 |
| Carbamazepine | 70773 |
| Carbamazepine | 71365 |
| Carbamazepine | 59211 |
| Carbamazepine | 51760 |
| Carbamazepine | 49833 |
| Carbamazepine | 32900 |
| Carbamazepine | 43451 |
| Carbamazepine | 53188 |
| Carbamazepine | 37800 |
| Carbamazepine | 18011 |
| Carbamazepine | 53492 |
| Carbamazepine | 46972 |
| Carbamazepine | 59745 |
| Carbamazepine | 76197 |
| Carbamazepine | 63930 |
| Carbamazepine | 2823 |
| Carbamazepine | 37058 |
| Carbamazepine | 71149 |
| Carbamazepine | 41726 |
| Carbamazepine | 1158 |
| Carbamazepine | 56734 |
| Carbamazepine | 57726 |
| Carbamazepine | 59697 |
| Carbamazepine | 78250 |
| Carbamazepine | 59746 |
| Carbamazepine | 71432 |
| Carbamazepine | 4982 |
| Carbamazepine | 17978 |
| Carbamazepine | 4066 |
| Carbamazepine | 63735 |
| Carbamazepine | 59429 |
| Carbamazepine | 30509 |
| Carbamazepine | 652 |
| Carbamazepine | 71350 |
| Carbamazepine | 72875 |
| Carbamazepine | 46888 |
| Carbamazepine | 60187 |
| Carbamazepine | 62457 |
| Carbamazepine | 432 |
| Carbamazepine | 72018 |
| Carbamazepine | 12932 |
| Carbamazepine | 62538 |
| Carbamazepine | 73827 |
| Carbamazepine | 79384 |
| Carbamazepine | 58515 |
| Carbamazepine | 18762 |
| Carbamazepine | 34958 |
| Carbamazepine | 66375 |
| Carbamazepine | 32931 |
| Carbamazepine | 5977 |
| Carbamazepine | 596 |
| Carbamazepine | 54445 |
| Carbamazepine | 59430 |
| Carbamazepine | 26012 |
| Carbamazepine | 4913 |
| Carbamazepine | 79486 |
| Carbamazepine | 60181 |
| Carbamazepine | 60405 |
| Carbamazepine | 45903 |
| Carbamazepine | 12931 |
| Carbamazepine | 12880 |
| Carbamazepine | 2824 |
| Carbamazepine | 59428 |
| Carbamazepine | 59696 |
| Carbamazepine | 30763 |
| Carbamazepine | 74819 |
| Carbamazepine | 40403 |
| Carbamazepine | 59038 |
| Carbamazepine | 79244 |
| Carbamazepine | 13112 |
| Carbamazepine | 69155 |
| Carbamazepine | 34904 |
| Carbamazepine | 61143 |
| Carbamazepine | 47294 |
| Carbamazepine | 55904 |
| Carbamazepine | 53893 |
| Carbamazepine | 61377 |
| Carbamazepine | 2085 |
| Carbamazepine | 63213 |
| Carbamazepine | 11696 |
| Carbamazepine | 63341 |
| Carbamazepine | 28345 |
| Celecoxib | 474 |
| Celecoxib | 66757 |
| Celecoxib | 64245 |
| Celecoxib | 43616 |
| Celecoxib | 72293 |
| Celecoxib | 5175 |
| Celecoxib | 80017 |
| Celecoxib | 72925 |
| Celecoxib | 77553 |
| Celecoxib | 5254 |
| Celecoxib | 65016 |
| Celecoxib | 5080 |
| Celecoxib | 62840 |
| Celecoxib | 50059 |
| Celecoxib | 64935 |
| Celecoxib | 52420 |
| Celecoxib | 55582 |
| Celecoxib | 66571 |
| Co-codamol | 65904 |
| Co-codamol | 22817 |
| Co-codamol | 36993 |
| Co-codamol | 65092 |
| Co-codamol | 34229 |
| Co-codamol | 58855 |
| Co-codamol | 47847 |
| Co-codamol | 55044 |
| Co-codamol | 67753 |
| Co-codamol | 73804 |
| Co-codamol | 33643 |
| Co-codamol | 29488 |
| Co-codamol | 24209 |
| Co-codamol | 63551 |
| Co-codamol | 46511 |
| Co-codamol | 64545 |
| Co-codamol | 800 |
| Co-codamol | 64387 |
| Co-codamol | 48311 |
| Co-codamol | 70497 |
| Co-codamol | 34784 |
| Co-codamol | 59705 |
| Co-codamol | 33961 |
| Co-codamol | 34518 |
| Co-codamol | 14602 |
| Co-codamol | 66553 |
| Co-codamol | 59479 |
| Co-codamol | 51819 |
| Co-codamol | 34815 |
| Co-codamol | 42791 |
| Co-codamol | 46633 |
| Co-codamol | 39340 |
| Co-codamol | 56461 |
| Co-codamol | 2794 |
| Co-codamol | 65314 |
| Co-codamol | 41276 |
| Co-codamol | 40385 |
| Co-codamol | 57839 |
| Co-codamol | 67106 |
| Co-codamol | 59442 |
| Co-codamol | 57900 |
| Co-codamol | 57 |
| Co-codamol | 77587 |
| Co-codamol | 29342 |
| Co-codamol | 73606 |
| Co-codamol | 74968 |
| Co-codamol | 74354 |
| Co-codamol | 57929 |
| Co-codamol | 41259 |
| Co-codamol | 56266 |
| Co-codamol | 73060 |
| Co-codamol | 56171 |
| Co-codamol | 34865 |
| Co-codamol | 27785 |
| Co-codamol | 34845 |
| Co-codamol | 53679 |
| Co-codamol | 57465 |
| Co-codamol | 52085 |
| Co-codamol | 41275 |
| Co-codamol | 46729 |
| Co-codamol | 58636 |
| Co-codamol | 66538 |
| Co-codamol | 60517 |
| Co-codamol | 53287 |
| Co-codamol | 63900 |
| Co-codamol | 7072 |
| Co-codamol | 40663 |
| Co-codamol | 62169 |
| Co-codamol | 27784 |
| Co-codamol | 53702 |
| Co-codamol | 77373 |
| Co-codamol | 34840 |
| Co-codamol | 58288 |
| Co-codamol | 65440 |
| Co-codamol | 56549 |
| Co-codamol | 625 |
| Co-codamol | 48775 |
| Co-codamol | 57097 |
| Co-codamol | 71614 |
| Co-codamol | 51084 |
| Co-codamol | 34257 |
| Co-codamol | 810 |
| Co-codamol | 34264 |
| Co-codamol | 57353 |
| Co-codamol | 43414 |
| Co-codamol | 33653 |
| Co-codamol | 1261 |
| Co-codamol | 58501 |
| Co-codamol | 43244 |
| Co-codamol | 64726 |
| Co-codamol | 72389 |
| Co-codamol | 46906 |
| Co-codamol | 31577 |
| Co-codamol | 41682 |
| Co-codamol | 68252 |
| Co-codamol | 46987 |
| Co-codamol | 36608 |
| Co-codamol | 3029 |
| Co-codamol | 59131 |
| Co-codamol | 57865 |
| Co-codamol | 44924 |
| Co-codamol | 42213 |
| Co-codamol | 34667 |
| Co-codamol | 34497 |
| Co-codamol | 61647 |
| Co-codamol | 75920 |
| Co-codamol | 19 |
| Co-codamol | 56565 |
| Co-codamol | 96 |
| Co-codamol | 79785 |
| Co-codamol | 34968 |
| Co-codamol | 59986 |
| Co-codamol | 40662 |
| Co-codamol | 17808 |
| Co-codamol | 33679 |
| Co-codamol | 32692 |
| Co-codamol | 66904 |
| Co-codamol | 55465 |
| Co-codamol | 34495 |
| Co-codamol | 30556 |
| Co-codamol | 70518 |
| Co-codamol | 36488 |
| Co-codamol | 78919 |
| Co-codamol | 69304 |
| Co-codamol | 56340 |
| Co-codamol | 65806 |
| Co-codamol | 43238 |
| Co-codamol | 37904 |
| Co-codamol | 66352 |
| Co-codamol | 56006 |
| Co-codamol | 74916 |
| Co-codamol | 33688 |
| Co-dydramol | 62635 |
| Co-dydramol | 5955 |
| Co-dydramol | 72844 |
| Co-dydramol | 65035 |
| Co-dydramol | 67779 |
| Co-dydramol | 79373 |
| Co-dydramol | 30444 |
| Co-dydramol | 72258 |
| Co-dydramol | 71006 |
| Co-dydramol | 9855 |
| Co-dydramol | 21229 |
| Co-dydramol | 40422 |
| Co-dydramol | 57197 |
| Co-dydramol | 72282 |
| Co-dydramol | 37291 |
| Co-dydramol | 28780 |
| Co-dydramol | 72110 |
| Co-dydramol | 71566 |
| Co-dydramol | 34737 |
| Co-dydramol | 77224 |
| Co-dydramol | 71523 |
| Co-dydramol | 36019 |
| Co-dydramol | 11 |
| Co-dydramol | 72111 |
| Co-dydramol | 33340 |
| Co-dydramol | 53079 |
| Co-dydramol | 61698 |
| Co-dydramol | 41278 |
| Co-dydramol | 19206 |
| Co-dydramol | 64074 |
| Co-dydramol | 32926 |
| Co-dydramol | 43441 |
| Co-dydramol | 55530 |
| Co-dydramol | 38430 |
| Co-dydramol | 21927 |
| Co-dydramol | 34939 |
| Co-dydramol | 30165 |
| Co-dydramol | 47071 |
| Co-dydramol | 15198 |
| Co-dydramol | 7063 |
| Co-dydramol | 34920 |
| Co-dydramol | 69006 |
| Co-dydramol | 61372 |
| Codeine phosphate | 34789 |
| Codeine phosphate | 65269 |
| Codeine phosphate | 9432 |
| Codeine phosphate | 2250 |
| Codeine phosphate | 46898 |
| Codeine phosphate | 28606 |
| Codeine phosphate | 382 |
| Codeine phosphate | 5498 |
| Codeine phosphate | 10178 |
| Codeine phosphate | 34168 |
| Codeine phosphate | 18221 |
| Codeine phosphate | 9129 |
| Codeine phosphate | 539 |
| Codeine phosphate | 51644 |
| Codeine phosphate | 66602 |
| Codeine phosphate | 2998 |
| Codeine phosphate | 7770 |
| Codeine phosphate | 3156 |
| Codeine phosphate | 7534 |
| Codeine phosphate | 44159 |
| Codeine phosphate | 13992 |
| Codeine phosphate | 38085 |
| Codeine phosphate | 10701 |
| Codeine phosphate | 28756 |
| Codeine phosphate | 11665 |
| Codeine phosphate | 56205 |
| Codeine phosphate | 66115 |
| Codeine phosphate | 5685 |
| Codeine phosphate | 24304 |
| Codeine phosphate | 24517 |
| Codeine phosphate | 58909 |
| Codeine phosphate | 6886 |
| Codeine phosphate | 71227 |
| Codeine phosphate | 38363 |
| Codeine phosphate | 9457 |
| Codeine phosphate | 30021 |
| Codeine phosphate | 24996 |
| Codeine phosphate | 14912 |
| Codeine phosphate | 21703 |
| Codeine phosphate | 52966 |
| Codeine phosphate | 142 |
| Codeine phosphate | 5572 |
| Codeine phosphate | 47081 |
| Codeine phosphate | 8053 |
| Codeine phosphate | 52888 |
| Codeine phosphate | 55309 |
| Codeine phosphate | 22764 |
| Codeine phosphate | 14785 |
| Codeine phosphate | 1708 |
| Codeine phosphate | 17563 |
| Codeine phosphate | 23420 |
| Codeine phosphate | 32510 |
| Codeine phosphate | 9917 |
| Codeine phosphate | 34172 |
| Codeine phosphate | 48136 |
| Codeine phosphate | 68861 |
| Codeine phosphate | 21673 |
| Codeine phosphate | 20077 |
| Codeine phosphate | 19622 |
| Codeine phosphate | 24859 |
| Codeine phosphate | 65118 |
| Codeine phosphate | 25330 |
| Codeine phosphate | 52929 |
| Codeine phosphate | 20565 |
| Codeine phosphate | 37298 |
| Codeine phosphate | 3713 |
| Codeine phosphate | 41416 |
| Codeine phosphate | 50659 |
| Codeine phosphate | 8246 |
| Codeine phosphate | 656 |
| Codeine phosphate | 31498 |
| Codeine phosphate | 9462 |
| Codeine phosphate | 20127 |
| Codeine phosphate | 152 |
| Codeine phosphate | 28784 |
| Codeine phosphate | 47508 |
| Codeine phosphate | 11461 |
| Codeine phosphate | 47003 |
| Codeine phosphate | 9516 |
| Codeine phosphate | 53600 |
| Codeine phosphate | 74247 |
| Codeine phosphate | 42792 |
| Codeine phosphate | 10602 |
| Codeine phosphate | 36013 |
| Codeine phosphate | 8879 |
| Codeine phosphate | 36152 |
| Codeine phosphate | 34152 |
| Codeine phosphate | 10582 |
| Codeine phosphate | 3034 |
| Codeine phosphate | 23977 |
| Codeine phosphate | 67751 |
| Codeine phosphate | 78049 |
| Codeine phosphate | 47919 |
| Codeine phosphate | 24498 |
| Codeine phosphate | 73631 |
| Codeine phosphate | 4671 |
| Codeine phosphate | 13893 |
| Codeine phosphate | 1616 |
| Codeine phosphate | 8732 |
| Codeine phosphate | 13598 |
| Codeine phosphate | 77131 |
| Codeine phosphate | 16039 |
| Codeine phosphate | 241 |
| Codeine phosphate | 7104 |
| Codeine phosphate | 14378 |
| Codeine phosphate | 7696 |
| Codeine phosphate | 4805 |
| Codeine phosphate | 11250 |
| Codeine phosphate | 58131 |
| Codeine phosphate | 8335 |
| Codeine phosphate | 306 |
| Codeine phosphate | 11009 |
| Codeine phosphate | 32436 |
| Codeine phosphate | 12709 |
| Codeine phosphate | 71987 |
| Codeine phosphate | 213 |
| Codeine phosphate | 61049 |
| Codeine phosphate | 30123 |
| Codeine phosphate | 19854 |
| Codeine phosphate | 9460 |
| Codeine phosphate | 10099 |
| Codeine phosphate | 17707 |
| Codeine phosphate | 50421 |
| Codeine phosphate | 4349 |
| Codeine phosphate | 8500 |
| Codeine phosphate | 69576 |
| Codeine phosphate | 7542 |
| Codeine phosphate | 25529 |
| Codeine phosphate | 3435 |
| Codeine phosphate | 41599 |
| Codeine phosphate | 34437 |
| Codeine phosphate | 60958 |
| Codeine phosphate | 42706 |
| Codeine phosphate | 56817 |
| Codeine phosphate | 25109 |
| Codeine phosphate | 19724 |
| Codeine phosphate | 72438 |
| Codeine phosphate | 4369 |
| Codeine phosphate | 16467 |
| Codeine phosphate | 2764 |
| Codeine phosphate | 16818 |
| Codeine phosphate | 31871 |
| Codeine phosphate | 21693 |
| Codeine phosphate | 66807 |
| Codeine phosphate | 72952 |
| Codeine phosphate | 11945 |
| Codeine phosphate | 32891 |
| Codeine phosphate | 51937 |
| Codeine phosphate | 26291 |
| Codeine phosphate | 11554 |
| Codeine phosphate | 16592 |
| Codeine phosphate | 65245 |
| Codeine phosphate | 8329 |
| Codeine phosphate | 33528 |
| Codeine phosphate | 27598 |
| Codeine phosphate | 11325 |
| Codeine phosphate | 4487 |
| Codeine phosphate | 51327 |
| Codeine phosphate | 29373 |
| Codeine phosphate | 48964 |
| Codeine phosphate | 63683 |
| Codeine phosphate | 71492 |
| Codeine phosphate | 12992 |
| Codeine phosphate | 6665 |
| Codeine phosphate | 15831 |
| Codeine phosphate | 34383 |
| Codeine phosphate | 76456 |
| Codeine phosphate | 4718 |
| Codeine phosphate | 20256 |
| Codeine phosphate | 8835 |
| Codeine phosphate | 17998 |
| Codeine phosphate | 53999 |
| Codeine phosphate | 22129 |
| Codeine phosphate | 76493 |
| Codeine phosphate | 3185 |
| Codeine phosphate | 158 |
| Codeine phosphate | 68562 |
| Codeine phosphate | 1640 |
| Codeine phosphate | 10226 |
| Codeine phosphate | 8233 |
| Codeine phosphate | 39461 |
| Codeine phosphate | 2846 |
| Codeine phosphate | 25514 |
| Codeine phosphate | 64108 |
| Codeine phosphate | 31155 |
| Codeine phosphate | 33260 |
| Codeine phosphate | 24828 |
| Codeine phosphate | 7518 |
| Codeine phosphate | 9044 |
| Codeine phosphate | 53617 |
| Codeine phosphate | 34090 |
| Codeine phosphate | 47200 |
| Codeine phosphate | 9742 |
| Codeine phosphate | 48004 |
| Codeine phosphate | 27353 |
| Codeine phosphate | 21880 |
| Codeine phosphate | 34176 |
| Codeine phosphate | 68509 |
| Codeine phosphate | 3272 |
| Codeine phosphate | 38987 |
| Codeine phosphate | 2917 |
| Codeine phosphate | 37348 |
| Codeine phosphate | 2178 |
| Codeine phosphate | 34444 |
| Codeine phosphate | 2698 |
| Codeine phosphate | 24187 |
| Codeine phosphate | 23952 |
| Codeine phosphate | 15779 |
| Codeine phosphate | 11961 |
| Codeine phosphate | 41523 |
| Codeine phosphate | 21251 |
| Codeine phosphate | 41214 |
| Codeine phosphate | 69066 |
| Codeine phosphate | 9202 |
| Codeine phosphate | 36846 |
| Codeine phosphate | 15937 |
| Codeine phosphate | 7499 |
| Codeine phosphate | 15871 |
| Codeine phosphate | 64752 |
| Codeine phosphate | 913 |
| Codeine phosphate | 22627 |
| Codeine phosphate | 51381 |
| Codeine phosphate | 48153 |
| Codeine phosphate | 17158 |
| Codeine phosphate | 62228 |
| Codeine phosphate | 56559 |
| Codeine phosphate | 7976 |
| Codeine phosphate | 47952 |
| Codeine phosphate | 2211 |
| Codeine phosphate | 35965 |
| Codeine phosphate | 35792 |
| Codeine phosphate | 61091 |
| Codeine phosphate | 38088 |
| Codeine phosphate | 31894 |
| Codeine phosphate | 57381 |
| Codeine phosphate | 50468 |
| Codeine phosphate | 24124 |
| Codeine phosphate | 2988 |
| Codeine phosphate | 48066 |
| Codeine phosphate | 31700 |
| Codeine phosphate | 12171 |
| Codeine phosphate | 14964 |
| Codeine phosphate | 43550 |
| Codeine phosphate | 41535 |
| Codeine phosphate | 34373 |
| Codeine phosphate | 66893 |
| Codeine phosphate | 21104 |
| Codeine phosphate | 767 |
| Codeine phosphate | 60640 |
| Codeine phosphate | 37816 |
| Codeine phosphate | 10176 |
| Codeine phosphate | 3724 |
| Codeine phosphate | 10519 |
| Codeine phosphate | 52889 |
| Codeine phosphate | 34348 |
| Codeine phosphate | 29828 |
| Codeine phosphate | 68538 |
| Codeine phosphate | 31452 |
| Codeine phosphate | 16096 |
| Codeine phosphate | 23580 |
| Codeine phosphate | 34099 |
| Codeine phosphate | 60040 |
| Codeine phosphate | 43504 |
| Codeine phosphate | 17926 |
| Codeine phosphate | 44210 |
| Codeine phosphate | 74109 |
| Codeine phosphate | 31943 |
| Codeine phosphate | 11807 |
| Codeine phosphate | 1617 |
| Codeine phosphate | 33495 |
| Codeine phosphate | 20853 |
| Codeine phosphate | 203 |
| Codeine phosphate | 69285 |
| Codeine phosphate | 21746 |
| Codeine phosphate | 57752 |
| Codeine phosphate | 14676 |
| Codeine phosphate | 57487 |
| Codeine phosphate | 24125 |
| Codeine phosphate | 32519 |
| Codeine phosphate | 34552 |
| Codeine phosphate | 22450 |
| Dexibuprofen | 21419 |
| Dexibuprofen | 21421 |
| Dexibuprofen | 10325 |
| Dexibuprofen | 11907 |
| Dexketoprofen | 5173 |
| Dexketoprofen | 9637 |
| Diclofenac potassium | 52338 |
| Diclofenac potassium | 69477 |
| Diclofenac potassium | 51099 |
| Diclofenac potassium | 65783 |
| Diclofenac potassium | 5401 |
| Diclofenac potassium | 58572 |
| Diclofenac potassium | 38817 |
| Diclofenac potassium | 5085 |
| Diclofenac potassium | 48871 |
| Diclofenac potassium | 48059 |
| Diclofenac potassium | 51343 |
| Diclofenac potassium | 45814 |
| Diclofenac potassium | 628 |
| Diclofenac potassium | 51293 |
| Diclofenac potassium | 43045 |
| Diclofenac potassium | 597 |
| Diclofenac potassium | 44112 |
| Diclofenac potassium | 39722 |
| Diclofenac potassium | 53345 |
| Diclofenac potassium | 47820 |
| Diclofenac potassium | 70468 |
| Diclofenac potassium | 50602 |
| Diclofenac potassium | 70145 |
| Diclofenac potassium | 58071 |
| Diclofenac sodium | 11540 |
| Diclofenac sodium | 28764 |
| Diclofenac sodium | 17491 |
| Diclofenac sodium | 31589 |
| Diclofenac sodium | 24128 |
| Diclofenac sodium | 33559 |
| Diclofenac sodium | 589 |
| Diclofenac sodium | 54021 |
| Diclofenac sodium | 917 |
| Diclofenac sodium | 48218 |
| Diclofenac sodium | 39264 |
| Diclofenac sodium | 58415 |
| Diclofenac sodium | 39823 |
| Diclofenac sodium | 79845 |
| Diclofenac sodium | 32536 |
| Diclofenac sodium | 53164 |
| Diclofenac sodium | 57162 |
| Diclofenac sodium | 47501 |
| Diclofenac sodium | 50269 |
| Diclofenac sodium | 2386 |
| Diclofenac sodium | 59880 |
| Diclofenac sodium | 19382 |
| Diclofenac sodium | 1075 |
| Diclofenac sodium | 16225 |
| Diclofenac sodium | 1139 |
| Diclofenac sodium | 497 |
| Diclofenac sodium | 21444 |
| Diclofenac sodium | 74835 |
| Diclofenac sodium | 11168 |
| Diclofenac sodium | 37688 |
| Diclofenac sodium | 56898 |
| Diclofenac sodium | 1692 |
| Diclofenac sodium | 31787 |
| Diclofenac sodium | 20105 |
| Diclofenac sodium | 30790 |
| Diclofenac sodium | 38948 |
| Diclofenac sodium | 30282 |
| Diclofenac sodium | 71362 |
| Diclofenac sodium | 60786 |
| Diclofenac sodium | 45213 |
| Diclofenac sodium | 18371 |
| Diclofenac sodium | 8789 |
| Diclofenac sodium | 50785 |
| Diclofenac sodium | 4631 |
| Diclofenac sodium | 928 |
| Diclofenac sodium | 55099 |
| Diclofenac sodium | 29181 |
| Diclofenac sodium | 10917 |
| Diclofenac sodium | 38881 |
| Diclofenac sodium | 20653 |
| Diclofenac sodium | 65877 |
| Diclofenac sodium | 61762 |
| Diclofenac sodium | 65528 |
| Diclofenac sodium | 24121 |
| Diclofenac sodium | 78675 |
| Diclofenac sodium | 26631 |
| Diclofenac sodium | 24236 |
| Diclofenac sodium | 77405 |
| Diclofenac sodium | 71088 |
| Diclofenac sodium | 54660 |
| Diclofenac sodium | 46844 |
| Diclofenac sodium | 30942 |
| Diclofenac sodium | 14085 |
| Diclofenac sodium | 64595 |
| Diclofenac sodium | 28390 |
| Diclofenac sodium | 17532 |
| Diclofenac sodium | 31950 |
| Diclofenac sodium | 33645 |
| Diclofenac sodium | 17525 |
| Diclofenac sodium | 20395 |
| Diclofenac sodium | 7458 |
| Diclofenac sodium | 70438 |
| Diclofenac sodium | 34362 |
| Diclofenac sodium | 1233 |
| Diclofenac sodium | 18921 |
| Diclofenac sodium | 71100 |
| Diclofenac sodium | 17128 |
| Diclofenac sodium | 35711 |
| Diclofenac sodium | 57006 |
| Diclofenac sodium | 38992 |
| Diclofenac sodium | 57045 |
| Diclofenac sodium | 32108 |
| Diclofenac sodium | 4506 |
| Diclofenac sodium | 21807 |
| Diclofenac sodium | 26351 |
| Diclofenac sodium | 47350 |
| Diclofenac sodium | 59289 |
| Diclofenac sodium | 58842 |
| Diclofenac sodium | 39876 |
| Diclofenac sodium | 16286 |
| Diclofenac sodium | 64759 |
| Diclofenac sodium | 32854 |
| Diclofenac sodium | 25362 |
| Diclofenac sodium | 21610 |
| Diclofenac sodium | 14678 |
| Diclofenac sodium | 4880 |
| Diclofenac sodium | 25790 |
| Diclofenac sodium | 2904 |
| Diclofenac sodium | 50317 |
| Diclofenac sodium | 42793 |
| Diclofenac sodium | 162 |
| Diclofenac sodium | 30806 |
| Diclofenac sodium | 20805 |
| Diclofenac sodium | 28256 |
| Diclofenac sodium | 31383 |
| Diclofenac sodium | 21387 |
| Diclofenac sodium | 39708 |
| Diclofenac sodium | 1446 |
| Diclofenac sodium | 49059 |
| Diclofenac sodium | 29330 |
| Diclofenac sodium | 9688 |
| Diclofenac sodium | 75136 |
| Diclofenac sodium | 58048 |
| Diclofenac sodium | 54075 |
| Diclofenac sodium | 34218 |
| Diclofenac sodium | 69584 |
| Diclofenac sodium | 612 |
| Diclofenac sodium | 56071 |
| Diclofenac sodium | 27200 |
| Diclofenac sodium | 9222 |
| Diclofenac sodium | 14084 |
| Diclofenac sodium | 1984 |
| Diclofenac sodium | 649 |
| Diclofenac sodium | 18798 |
| Diclofenac sodium | 417 |
| Diclofenac sodium | 42905 |
| Diclofenac sodium | 21824 |
| Diclofenac sodium | 1096 |
| Diclofenac sodium | 29037 |
| Diclofenac sodium | 77392 |
| Diclofenac sodium | 80099 |
| Diclofenac sodium | 9465 |
| Diclofenac sodium | 30297 |
| Diclofenac sodium | 580 |
| Diclofenac sodium | 14672 |
| Diclofenac sodium | 59595 |
| Diclofenac sodium | 34487 |
| Diclofenac sodium | 71117 |
| Diclofenac sodium | 32916 |
| Diclofenac sodium | 15732 |
| Diclofenac sodium | 34091 |
| Diclofenac sodium | 60443 |
| Diclofenac sodium | 6435 |
| Diclofenac sodium | 71064 |
| Diclofenac sodium | 56078 |
| Diclofenac sodium | 8062 |
| Diclofenac sodium | 6115 |
| Diclofenac sodium | 17124 |
| Diclofenac sodium | 34212 |
| Diclofenac sodium | 65179 |
| Diclofenac sodium | 29455 |
| Diclofenac sodium | 74028 |
| Diclofenac sodium | 60368 |
| Diclofenac sodium | 16222 |
| Diclofenac sodium | 34271 |
| Diclofenac sodium | 33669 |
| Diclofenac sodium | 34744 |
| Diclofenac sodium | 14707 |
| Diclofenac sodium | 74211 |
| Diclofenac sodium | 27362 |
| Diclofenac sodium | 1766 |
| Diclofenac sodium | 53384 |
| Diclofenac sodium | 4625 |
| Diclofenac sodium | 28553 |
| Diclofenac sodium | 33457 |
| Diclofenac sodium | 71307 |
| Diclofenac sodium | 75442 |
| Diclofenac sodium | 51808 |
| Diclofenac sodium | 3416 |
| Diclofenac sodium | 29523 |
| Diclofenac sodium | 79586 |
| Diclofenac sodium | 11522 |
| Diclofenac sodium | 3421 |
| Diclofenac sodium | 447 |
| Diclofenac sodium | 80121 |
| Diclofenac sodium | 40 |
| Diclofenac sodium | 66123 |
| Diclofenac sodium | 66577 |
| Diclofenac sodium | 42406 |
| Diclofenac sodium | 68354 |
| Diclofenac sodium | 2387 |
| Diclofenac sodium | 62636 |
| Diclofenac sodium | 72396 |
| Diclofenac sodium | 25329 |
| Diclofenac sodium | 65007 |
| Diclofenac sodium | 54518 |
| Diclofenac sodium | 61596 |
| Diclofenac sodium | 64303 |
| Diclofenac sodium | 40086 |
| Diclofenac sodium | 11322 |
| Diclofenac sodium | 6881 |
| Diclofenac sodium | 30849 |
| Diclofenac sodium | 9500 |
| Diclofenac sodium | 54906 |
| Diclofenac sodium | 26165 |
| Diclofenac sodium | 25358 |
| Diclofenac sodium | 60666 |
| Diclofenac sodium | 26888 |
| Diclofenac sodium | 40756 |
| Diclofenac sodium | 16221 |
| Diclofenac sodium | 17029 |
| Diclofenac sodium | 74451 |
| Diclofenac sodium | 4692 |
| Diclofenac sodium | 27055 |
| Diclofenac sodium | 36486 |
| Diclofenac sodium | 50058 |
| Diclofenac sodium | 72546 |
| Diclofenac sodium | 35893 |
| Diclofenac sodium | 80098 |
| Diclofenac sodium | 67220 |
| Diclofenac sodium | 25361 |
| Diclofenac sodium | 79731 |
| Diclofenac sodium | 1115 |
| Diclofenac sodium | 74048 |
| Diclofenac sodium | 17030 |
| Diclofenac sodium | 33994 |
| Diclofenac sodium | 17126 |
| Diclofenac sodium | 15201 |
| Diclofenac sodium | 3852 |
| Diclofenac sodium | 54463 |
| Diclofenac sodium | 42455 |
| Diclofenac sodium | 20384 |
| Diclofenac sodium | 31944 |
| Diclofenac sodium | 25283 |
| Diclofenac sodium | 16272 |
| Diclofenac sodium | 9886 |
| Diclofenac sodium | 20621 |
| Diclofenac sodium | 73131 |
| Diclofenac sodium | 68849 |
| Diclofenac sodium | 24122 |
| Dihydrocodeine tartrate | 73145 |
| Dihydrocodeine tartrate | 38521 |
| Dihydrocodeine tartrate | 38950 |
| Dihydrocodeine tartrate | 54713 |
| Dihydrocodeine tartrate | 34440 |
| Dihydrocodeine tartrate | 59989 |
| Dihydrocodeine tartrate | 33743 |
| Dihydrocodeine tartrate | 6234 |
| Dihydrocodeine tartrate | 4950 |
| Dihydrocodeine tartrate | 34662 |
| Dihydrocodeine tartrate | 34579 |
| Dihydrocodeine tartrate | 17917 |
| Dihydrocodeine tartrate | 9209 |
| Dihydrocodeine tartrate | 33654 |
| Dihydrocodeine tartrate | 7989 |
| Dihydrocodeine tartrate | 72209 |
| Dihydrocodeine tartrate | 73147 |
| Dihydrocodeine tartrate | 34730 |
| Dihydrocodeine tartrate | 64079 |
| Dihydrocodeine tartrate | 10122 |
| Dihydrocodeine tartrate | 2555 |
| Dihydrocodeine tartrate | 10023 |
| Dihydrocodeine tartrate | 9275 |
| Dihydrocodeine tartrate | 53 |
| Dihydrocodeine tartrate | 34008 |
| Dihydrocodeine tartrate | 9163 |
| Dihydrocodeine tartrate | 4823 |
| Dihydrocodeine tartrate | 72210 |
| Dihydrocodeine tartrate | 26653 |
| Dihydrocodeine tartrate | 72265 |
| Dihydrocodeine tartrate | 9562 |
| Dihydrocodeine tartrate | 39558 |
| Dihydrocodeine tartrate | 55425 |
| Dihydrocodeine tartrate | 2041 |
| Dihydrocodeine tartrate | 78685 |
| Dihydrocodeine tartrate | 2040 |
| Dihydrocodeine tartrate | 191 |
| Dihydrocodeine tartrate | 28598 |
| Dihydrocodeine tartrate | 50532 |
| Dihydrocodeine tartrate | 77505 |
| Dihydrocodeine tartrate | 64368 |
| Dihydrocodeine tartrate | 40159 |
| Dihydrocodeine tartrate | 66121 |
| Dihydrocodeine tartrate | 38970 |
| Dihydrocodeine tartrate | 58848 |
| Dihydrocodeine tartrate | 42208 |
| Dihydrocodeine tartrate | 3698 |
| Dihydrocodeine tartrate | 4556 |
| Dihydrocodeine tartrate | 48133 |
| Dihydrocodeine tartrate | 8456 |
| Dihydrocodeine tartrate | 59978 |
| Dihydrocodeine tartrate | 7469 |
| Dihydrocodeine tartrate | 14688 |
| Dihydrocodeine tartrate | 65689 |
| Dihydrocodeine tartrate | 30295 |
| Dihydrocodeine tartrate | 9785 |
| Dihydrocodeine tartrate | 9313 |
| Dihydrocodeine tartrate | 21113 |
| Dihydrocodeine tartrate | 16112 |
| Dihydrocodeine tartrate | 54354 |
| Dipipanone | 38301 |
| Dipipanone | 12020 |
| Dipipanone | 9001 |
| Etodolac | 4368 |
| Etodolac | 35653 |
| Etodolac | 38770 |
| Etodolac | 5455 |
| Etodolac | 76419 |
| Etodolac | 8451 |
| Etodolac | 71908 |
| Etodolac | 52714 |
| Etodolac | 3311 |
| Etodolac | 24356 |
| Etodolac | 66323 |
| Etodolac | 16194 |
| Etodolac | 20386 |
| Etodolac | 5266 |
| Etodolac | 10033 |
| Etodolac | 8969 |
| Etoricoxib | 650 |
| Etoricoxib | 78096 |
| Etoricoxib | 9822 |
| Etoricoxib | 51874 |
| Etoricoxib | 75549 |
| Etoricoxib | 37562 |
| Etoricoxib | 56584 |
| Etoricoxib | 53576 |
| Etoricoxib | 62658 |
| Etoricoxib | 62251 |
| Etoricoxib | 66486 |
| Etoricoxib | 5812 |
| Etoricoxib | 5938 |
| Etoricoxib | 74952 |
| Etoricoxib | 64521 |
| Etoricoxib | 6464 |
| Etoricoxib | 37587 |
| Etoricoxib | 51284 |
| Etoricoxib | 6498 |
| Etoricoxib | 78072 |
| Etoricoxib | 62843 |
| Felbinac | 9350 |
| Felbinac | 10338 |
| Felbinac | 8446 |
| Felbinac | 3587 |
| Felbinac | 559 |
| Fentany | 38553 |
| Fentany | 69023 |
| Fentany | 47413 |
| Fentany | 22066 |
| Fentany | 45460 |
| Fentany | 10922 |
| Fentany | 74779 |
| Fentany | 61156 |
| Fentany | 36040 |
| Fentany | 50671 |
| Fentany | 37719 |
| Fentany | 55752 |
| Fentany | 37923 |
| Fentany | 37960 |
| Fentany | 70376 |
| Fentany | 46560 |
| Fentany | 46559 |
| Fentany | 60766 |
| Fentany | 56670 |
| Fentany | 14900 |
| Fentany | 39251 |
| Fentany | 38326 |
| Fentany | 37954 |
| Fentany | 28189 |
| Fentany | 4691 |
| Fentany | 70810 |
| Fentany | 46733 |
| Fentany | 67830 |
| Fentany | 51235 |
| Fentany | 73333 |
| Fentany | 76724 |
| Fentany | 38351 |
| Fentany | 39180 |
| Fentany | 44837 |
| Fentany | 43152 |
| Fentany | 42021 |
| Fentany | 60477 |
| Fentany | 7126 |
| Fentany | 11982 |
| Fentany | 35968 |
| Fentany | 37968 |
| Fentany | 5657 |
| Fentany | 67425 |
| Fentany | 42576 |
| Fentany | 42591 |
| Fentany | 65359 |
| Fentany | 16618 |
| Fentany | 7082 |
| Fentany | 68209 |
| Fentany | 44487 |
| Fentany | 46658 |
| Fentany | 31053 |
| Fentany | 37928 |
| Fentany | 70988 |
| Fentany | 75193 |
| Fentany | 61305 |
| Fentany | 36211 |
| Fentany | 757 |
| Fentany | 46657 |
| Fentany | 63139 |
| Fentany | 73664 |
| Fentany | 7397 |
| Fentany | 67766 |
| Fentany | 67474 |
| Fentany | 6298 |
| Fentany | 41161 |
| Fentany | 38365 |
| Fentany | 42590 |
| Fentany | 7107 |
| Fentany | 65168 |
| Fentany | 50929 |
| Fentany | 72342 |
| Fentany | 748 |
| Fentany | 59490 |
| Fentany | 38031 |
| Fentany | 72820 |
| Fentany | 620 |
| Fentany | 48571 |
| Fentany | 78444 |
| Fentany | 5048 |
| Fentany | 15350 |
| Fentany | 61086 |
| Fentany | 79446 |
| Fentany | 67258 |
| Fentany | 73649 |
| Fentany | 59482 |
| Fentany | 45549 |
| Fentany | 65437 |
| Fentany | 39084 |
| Fentany | 37779 |
| Fentany | 54979 |
| Flurbiprofen | 3182 |
| Flurbiprofen | 34725 |
| Flurbiprofen | 6249 |
| Flurbiprofen | 2366 |
| Flurbiprofen | 9439 |
| Flurbiprofen | 3266 |
| Flurbiprofen | 38944 |
| Flurbiprofen | 4043 |
| Gabapentin | 67091 |
| Gabapentin | 78801 |
| Gabapentin | 70459 |
| Gabapentin | 48060 |
| Gabapentin | 78641 |
| Gabapentin | 77136 |
| Gabapentin | 34716 |
| Gabapentin | 55624 |
| Gabapentin | 44187 |
| Gabapentin | 10007 |
| Gabapentin | 67969 |
| Gabapentin | 55535 |
| Gabapentin | 70738 |
| Gabapentin | 71013 |
| Gabapentin | 77695 |
| Gabapentin | 64981 |
| Gabapentin | 68047 |
| Gabapentin | 54609 |
| Gabapentin | 28713 |
| Gabapentin | 79400 |
| Gabapentin | 70954 |
| Gabapentin | 73587 |
| Gabapentin | 64306 |
| Gabapentin | 73047 |
| Gabapentin | 34946 |
| Gabapentin | 59196 |
| Gabapentin | 58382 |
| Gabapentin | 7538 |
| Gabapentin | 63432 |
| Gabapentin | 76014 |
| Gabapentin | 6304 |
| Gabapentin | 25815 |
| Gabapentin | 48035 |
| Gabapentin | 73635 |
| Gabapentin | 660 |
| Gabapentin | 5221 |
| Gabapentin | 27454 |
| Gabapentin | 1584 |
| Gabapentin | 70506 |
| Gabapentin | 16215 |
| Gabapentin | 57120 |
| Gabapentin | 76604 |
| Gabapentin | 64302 |
| Gabapentin | 47579 |
| Gabapentin | 34606 |
| Gabapentin | 59147 |
| Gabapentin | 64213 |
| Gabapentin | 4781 |
| Gabapentin | 58162 |
| Gabapentin | 51118 |
| Gabapentin | 17564 |
| Gabapentin | 58960 |
| Gabapentin | 34506 |
| Gabapentin | 44022 |
| Gabapentin | 58383 |
| Gabapentin | 63375 |
| Gabapentin | 53296 |
| Gabapentin | 70247 |
| Gabapentin | 53784 |
| Gabapentin | 18211 |
| Gabapentin | 76435 |
| Gabapentin | 68049 |
| Gabapentin | 61266 |
| Gabapentin | 44261 |
| Gabapentin | 57649 |
| Gabapentin | 57527 |
| Gabapentin | 66617 |
| Gabapentin | 78410 |
| Gabapentin | 60389 |
| Gabapentin | 58472 |
| Gabapentin | 69914 |
| Gabapentin | 72849 |
| Hydromorphone hydrochloride | 21275 |
| Hydromorphone hydrochloride | 15792 |
| Hydromorphone hydrochloride | 24736 |
| Hydromorphone hydrochloride | 9330 |
| Hydromorphone hydrochloride | 15798 |
| Hydromorphone hydrochloride | 5137 |
| Hydromorphone hydrochloride | 9325 |
| Hydromorphone hydrochloride | 9331 |
| Hydromorphone hydrochloride | 21285 |
| Hydromorphone hydrochloride | 5138 |
| Hydromorphone hydrochloride | 19954 |
| Hydromorphone hydrochloride | 19972 |
| Hydromorphone hydrochloride | 9332 |
| Hydromorphone hydrochloride | 9615 |
| Ibuprofen | 27438 |
| Ibuprofen | 74806 |
| Ibuprofen | 51943 |
| Ibuprofen | 65514 |
| Ibuprofen | 67740 |
| Ibuprofen | 5767 |
| Ibuprofen | 55233 |
| Ibuprofen | 69935 |
| Ibuprofen | 51923 |
| Ibuprofen | 46342 |
| Ibuprofen | 38493 |
| Ibuprofen | 32365 |
| Ibuprofen | 37972 |
| Ibuprofen | 47401 |
| Ibuprofen | 2938 |
| Ibuprofen | 19046 |
| Ibuprofen | 37850 |
| Ibuprofen | 44730 |
| Ibuprofen | 73433 |
| Ibuprofen | 3597 |
| Ibuprofen | 34354 |
| Ibuprofen | 34621 |
| Ibuprofen | 44483 |
| Ibuprofen | 56441 |
| Ibuprofen | 30382 |
| Ibuprofen | 28348 |
| Ibuprofen | 11980 |
| Ibuprofen | 43904 |
| Ibuprofen | 345 |
| Ibuprofen | 46860 |
| Ibuprofen | 24305 |
| Ibuprofen | 36329 |
| Ibuprofen | 27968 |
| Ibuprofen | 32704 |
| Ibuprofen | 42108 |
| Ibuprofen | 27782 |
| Ibuprofen | 16193 |
| Ibuprofen | 4911 |
| Ibuprofen | 37002 |
| Ibuprofen | 32862 |
| Ibuprofen | 1621 |
| Ibuprofen | 37553 |
| Ibuprofen | 66247 |
| Ibuprofen | 30892 |
| Ibuprofen | 37053 |
| Ibuprofen | 48675 |
| Ibuprofen | 57112 |
| Ibuprofen | 77525 |
| Ibuprofen | 76041 |
| Ibuprofen | 54137 |
| Ibuprofen | 65471 |
| Ibuprofen | 32875 |
| Ibuprofen | 76284 |
| Ibuprofen | 4762 |
| Ibuprofen | 61260 |
| Ibuprofen | 45145 |
| Ibuprofen | 20978 |
| Ibuprofen | 69271 |
| Ibuprofen | 38527 |
| Ibuprofen | 11550 |
| Ibuprofen | 33589 |
| Ibuprofen | 40394 |
| Ibuprofen | 34911 |
| Ibuprofen | 35749 |
| Ibuprofen | 7261 |
| Ibuprofen | 73112 |
| Ibuprofen | 23425 |
| Ibuprofen | 56039 |
| Ibuprofen | 29352 |
| Ibuprofen | 29316 |
| Ibuprofen | 28479 |
| Ibuprofen | 50813 |
| Ibuprofen | 48568 |
| Ibuprofen | 55313 |
| Ibuprofen | 6853 |
| Ibuprofen | 13807 |
| Ibuprofen | 34961 |
| Ibuprofen | 10149 |
| Ibuprofen | 53604 |
| Ibuprofen | 33704 |
| Ibuprofen | 59502 |
| Ibuprofen | 2129 |
| Ibuprofen | 25794 |
| Ibuprofen | 48644 |
| Ibuprofen | 19036 |
| Ibuprofen | 48138 |
| Ibuprofen | 26095 |
| Ibuprofen | 50314 |
| Ibuprofen | 66567 |
| Ibuprofen | 43032 |
| Ibuprofen | 28172 |
| Ibuprofen | 71949 |
| Ibuprofen | 72853 |
| Ibuprofen | 62238 |
| Ibuprofen | 34663 |
| Ibuprofen | 51614 |
| Ibuprofen | 332 |
| Ibuprofen | 15930 |
| Ibuprofen | 29749 |
| Ibuprofen | 27783 |
| Ibuprofen | 43456 |
| Ibuprofen | 76476 |
| Ibuprofen | 20967 |
| Ibuprofen | 71968 |
| Ibuprofen | 15363 |
| Ibuprofen | 849 |
| Ibuprofen | 13083 |
| Ibuprofen | 34359 |
| Ibuprofen | 45988 |
| Ibuprofen | 29332 |
| Ibuprofen | 14333 |
| Ibuprofen | 40083 |
| Ibuprofen | 50266 |
| Ibuprofen | 5648 |
| Ibuprofen | 74216 |
| Ibuprofen | 7141 |
| Ibuprofen | 48562 |
| Ibuprofen | 26970 |
| Ibuprofen | 38182 |
| Ibuprofen | 53397 |
| Ibuprofen | 73743 |
| Ibuprofen | 21811 |
| Ibuprofen | 10265 |
| Ibuprofen | 68582 |
| Ibuprofen | 48326 |
| Ibuprofen | 21821 |
| Ibuprofen | 71779 |
| Ibuprofen | 407 |
| Ibuprofen | 784 |
| Ibuprofen | 25257 |
| Ibuprofen | 2622 |
| Ibuprofen | 8401 |
| Ibuprofen | 40516 |
| Ibuprofen | 55434 |
| Ibuprofen | 112 |
| Ibuprofen | 34536 |
| Ibuprofen | 55153 |
| Ibuprofen | 9201 |
| Ibuprofen | 46141 |
| Ibuprofen | 4216 |
| Ibuprofen | 24469 |
| Ibuprofen | 52154 |
| Ibuprofen | 4298 |
| Ibuprofen | 28168 |
| Ibuprofen | 79280 |
| Ibuprofen | 45331 |
| Ibuprofen | 61878 |
| Ibuprofen | 46921 |
| Ibuprofen | 68220 |
| Ibuprofen | 43426 |
| Ibuprofen | 25619 |
| Ibuprofen | 17068 |
| Ibuprofen | 34425 |
| Ibuprofen | 51769 |
| Ibuprofen | 53331 |
| Ibuprofen | 71314 |
| Ibuprofen | 44233 |
| Ibuprofen | 76093 |
| Ibuprofen | 75338 |
| Ibuprofen | 35292 |
| Ibuprofen | 402 |
| Ibuprofen | 36650 |
| Ibuprofen | 50363 |
| Ibuprofen | 21815 |
| Ibuprofen | 37253 |
| Ibuprofen | 19398 |
| Ibuprofen | 59562 |
| Ibuprofen | 32242 |
| Ibuprofen | 26159 |
| Ibuprofen | 76234 |
| Ibuprofen | 71374 |
| Ibuprofen | 76300 |
| Ibuprofen | 40253 |
| Ibuprofen | 1739 |
| Ibuprofen | 68018 |
| Ibuprofen | 71041 |
| Ibuprofen | 63036 |
| Ibuprofen | 28888 |
| Ibuprofen | 1468 |
| Ibuprofen | 12776 |
| Ibuprofen | 14570 |
| Ibuprofen | 34931 |
| Ibuprofen | 20442 |
| Ibuprofen | 29068 |
| Ibuprofen | 22206 |
| Ibuprofen | 1270 |
| Ibuprofen | 58888 |
| Ibuprofen | 32136 |
| Ibuprofen | 30811 |
| Ibuprofen | 31482 |
| Ibuprofen | 17201 |
| Ibuprofen | 60035 |
| Ibuprofen | 15 |
| Ibuprofen | 41701 |
| Ibuprofen | 59553 |
| Ibuprofen | 2693 |
| Ibuprofen | 36606 |
| Ibuprofen | 66648 |
| Ibuprofen | 77228 |
| Ibuprofen | 24086 |
| Ibuprofen | 18527 |
| Ibuprofen | 38332 |
| Ibuprofen | 37235 |
| Ibuprofen | 29345 |
| Ibuprofen | 16192 |
| Ibuprofen | 77335 |
| Ibuprofen | 37731 |
| Ibuprofen | 25205 |
| Ibuprofen | 66544 |
| Ibuprofen | 43096 |
| Ibuprofen | 65591 |
| Ibuprofen | 33935 |
| Ibuprofen | 78149 |
| Ibuprofen | 30164 |
| Ibuprofen | 49133 |
| Ibuprofen | 29524 |
| Ibuprofen | 30724 |
| Ibuprofen | 65025 |
| Ibuprofen | 28522 |
| Ibuprofen | 45216 |
| Ibuprofen | 7058 |
| Ibuprofen | 36787 |
| Ibuprofen | 56213 |
| Ibuprofen | 75677 |
| Ibuprofen | 392 |
| Ibuprofen | 1156 |
| Ibuprofen | 75305 |
| Ibuprofen | 416 |
| Ibuprofen | 39873 |
| Ibuprofen | 36597 |
| Ibuprofen | 29704 |
| Ibuprofen | 60510 |
| Ibuprofen | 39019 |
| Ibuprofen | 18364 |
| Ibuprofen | 25800 |
| Ibuprofen | 586 |
| Ibuprofen | 31469 |
| Ibuprofen | 49277 |
| Ibuprofen | 31054 |
| Ibuprofen | 70878 |
| Ibuprofen | 79167 |
| Ibuprofen | 54514 |
| Ibuprofen | 18196 |
| Ibuprofen | 48546 |
| Ibuprofen | 32100 |
| Ibuprofen | 14385 |
| Ibuprofen | 51828 |
| Ibuprofen | 39758 |
| Ibuprofen | 63079 |
| Ibuprofen | 4648 |
| Ibuprofen | 61953 |
| Ibuprofen | 8510 |
| Ibuprofen | 20907 |
| Ibuprofen | 59067 |
| Ibuprofen | 44892 |
| Ibuprofen | 34850 |
| Ibuprofen | 53803 |
| Ibuprofen | 70821 |
| Ibuprofen | 18820 |
| Ibuprofen | 34757 |
| Ibuprofen | 16001 |
| Ibuprofen | 59203 |
| Ibuprofen | 48738 |
| Ibuprofen | 72156 |
| Ibuprofen | 29587 |
| Ibuprofen | 21813 |
| Ibuprofen | 48062 |
| Ibuprofen | 33785 |
| Ibuprofen | 45842 |
| Ibuprofen | 62892 |
| Ibuprofen | 43911 |
| Ibuprofen | 18812 |
| Ibuprofen | 647 |
| Ibuprofen | 34980 |
| Ibuprofen | 66353 |
| Ibuprofen | 22283 |
| Ibuprofen | 28519 |
| Ibuprofen | 76342 |
| Ibuprofen | 66461 |
| Ibuprofen | 71072 |
| Ibuprofen | 66194 |
| Ibuprofen | 35265 |
| Ibuprofen | 215 |
| Ibuprofen | 55009 |
| Ibuprofen | 50652 |
| Ibuprofen | 65121 |
| Ibuprofen | 69018 |
| Ibuprofen | 4731 |
| Ibuprofen | 63132 |
| Ibuprofen | 5896 |
| Ibuprofen | 33357 |
| Ibuprofen | 15767 |
| Ibuprofen | 39502 |
| Ibuprofen | 7535 |
| Ibuprofen | 19575 |
| Ibuprofen | 49432 |
| Ibuprofen | 32509 |
| Ibuprofen | 34527 |
| Ibuprofen | 30243 |
| Ibuprofen | 34447 |
| Ibuprofen | 74313 |
| Ibuprofen | 34729 |
| Ibuprofen | 48084 |
| Ibuprofen | 71584 |
| Ibuprofen | 67594 |
| Ibuprofen | 50117 |
| Ibuprofen | 52617 |
| Ibuprofen | 21045 |
| Ibuprofen | 10209 |
| Ibuprofen | 46904 |
| Ibuprofen | 1086 |
| Ibuprofen | 28822 |
| Ibuprofen | 37094 |
| Ibuprofen | 49266 |
| Ibuprofen | 46942 |
| Ibuprofen | 32366 |
| Ibuprofen | 39354 |
| Ibuprofen | 41513 |
| Ibuprofen | 35890 |
| Ibuprofen | 70299 |
| Ibuprofen | 58652 |
| Ibuprofen | 45320 |
| Ibuprofen | 3599 |
| Ibuprofen | 24887 |
| Ibuprofen | 360 |
| Ibuprofen | 78978 |
| Ibuprofen | 50628 |
| Ibuprofen | 15068 |
| Ibuprofen | 42397 |
| Ibuprofen | 10785 |
| Ibuprofen | 1392 |
| Ibuprofen | 34550 |
| Ibuprofen | 52009 |
| Ibuprofen | 29232 |
| Ibuprofen | 1030 |
| Ibuprofen | 4309 |
| Ibuprofen | 75893 |
| Ibuprofen | 37648 |
| Ibuprofen | 34889 |
| Ibuprofen | 73040 |
| Indometacin | 10558 |
| Indometacin | 17680 |
| Indometacin | 33318 |
| Indometacin | 68708 |
| Indometacin | 33113 |
| Indometacin | 45256 |
| Indometacin | 24193 |
| Indometacin | 1210 |
| Indometacin | 24320 |
| Indometacin | 75765 |
| Indometacin | 76583 |
| Indometacin | 15005 |
| Indometacin | 76610 |
| Indometacin | 41817 |
| Indometacin | 23026 |
| Indometacin | 60930 |
| Indometacin | 1051 |
| Indometacin | 736 |
| Indometacin | 76608 |
| Indometacin | 34190 |
| Indometacin | 52141 |
| Indometacin | 31959 |
| Indometacin | 17750 |
| Indometacin | 51339 |
| Indometacin | 120 |
| Indometacin | 58523 |
| Indometacin | 77102 |
| Indometacin | 3168 |
| Indometacin | 1496 |
| Indometacin | 44313 |
| Indometacin | 24308 |
| Indometacin | 28900 |
| Indometacin | 18662 |
| Indometacin | 24212 |
| Indometacin | 42003 |
| Indometacin | 2200 |
| Indometacin | 18234 |
| Indometacin | 41615 |
| Indometacin | 13606 |
| Indometacin | 33321 |
| Indometacin | 67756 |
| Indometacin | 77359 |
| Indometacin | 72972 |
| Indometacin | 2671 |
| Indometacin | 60772 |
| Indometacin | 36577 |
| Indometacin | 13639 |
| Indometacin | 1688 |
| Indometacin | 14476 |
| Indometacin | 23204 |
| Indometacin | 41521 |
| Indometacin | 3216 |
| Indometacin | 62643 |
| Indometacin | 24137 |
| Indometacin | 10625 |
| Indometacin | 60916 |
| Indometacin | 79433 |
| Indometacin | 41823 |
| Indometacin | 32097 |
| Indometacin | 32641 |
| Indometacin | 70904 |
| Indometacin | 177 |
| Ketoprofen | 41364 |
| Ketoprofen | 3043 |
| Ketoprofen | 41365 |
| Ketoprofen | 21050 |
| Ketoprofen | 31916 |
| Ketoprofen | 11999 |
| Ketoprofen | 18647 |
| Ketoprofen | 27082 |
| Ketoprofen | 21955 |
| Ketoprofen | 62615 |
| Ketoprofen | 2606 |
| Ketoprofen | 41366 |
| Ketoprofen | 71376 |
| Ketoprofen | 12122 |
| Ketoprofen | 7840 |
| Ketoprofen | 17818 |
| Ketoprofen | 46920 |
| Ketoprofen | 27778 |
| Ketoprofen | 1571 |
| Ketoprofen | 71127 |
| Ketoprofen | 40215 |
| Ketoprofen | 8385 |
| Ketoprofen | 29772 |
| Ketoprofen | 13347 |
| Ketoprofen | 78779 |
| Ketoprofen | 30327 |
| Ketoprofen | 75581 |
| Ketoprofen | 3326 |
| Ketoprofen | 32227 |
| Ketoprofen | 838 |
| Ketoprofen | 77293 |
| Ketoprofen | 71104 |
| Ketoprofen | 3077 |
| Ketoprofen | 9421 |
| Ketoprofen | 67803 |
| Ketoprofen | 25701 |
| Ketoprofen | 46919 |
| Ketoprofen | 42500 |
| Ketoprofen | 40664 |
| Ketoprofen | 74005 |
| Ketoprofen | 24617 |
| Ketoprofen | 75771 |
| Ketoprofen | 77459 |
| Ketoprofen | 46940 |
| Ketoprofen | 40484 |
| Ketoprofen | 73657 |
| Ketoprofen | 75573 |
| Ketoprofen | 41367 |
| Ketoprofen | 40336 |
| Ketoprofen | 389 |
| Ketoprofen | 77582 |
| Ketoprofen | 40141 |
| Ketoprofen | 15286 |
| Ketoprofen | 31178 |
| Ketoprofen | 33568 |
| Ketoprofen | 57943 |
| Ketoprofen | 1231 |
| Ketoprofen | 27013 |
| Ketoprofen | 40185 |
| Ketoprofen | 31962 |
| Ketoprofen | 33180 |
| Mefenamic acid | 25013 |
| Mefenamic acid | 34595 |
| Mefenamic acid | 76310 |
| Mefenamic acid | 46967 |
| Mefenamic acid | 46968 |
| Mefenamic acid | 14541 |
| Mefenamic acid | 13459 |
| Mefenamic acid | 57007 |
| Mefenamic acid | 1246 |
| Mefenamic acid | 30391 |
| Mefenamic acid | 34924 |
| Mefenamic acid | 26247 |
| Mefenamic acid | 41677 |
| Mefenamic acid | 70221 |
| Mefenamic acid | 26522 |
| Mefenamic acid | 36260 |
| Mefenamic acid | 32090 |
| Mefenamic acid | 32234 |
| Mefenamic acid | 41524 |
| Mefenamic acid | 20709 |
| Mefenamic acid | 1073 |
| Mefenamic acid | 61581 |
| Mefenamic acid | 79173 |
| Mefenamic acid | 22230 |
| Mefenamic acid | 259 |
| Mefenamic acid | 27528 |
| Mefenamic acid | 57297 |
| Mefenamic acid | 30389 |
| Mefenamic acid | 1983 |
| Mefenamic acid | 4710 |
| Mefenamic acid | 9736 |
| Mefenamic acid | 51827 |
| Mefenamic acid | 34898 |
| Mefenamic acid | 296 |
| Mefenamic acid | 34910 |
| Mefenamic acid | 64103 |
| Mefenamic acid | 33801 |
| Mefenamic acid | 34793 |
| Mefenamic acid | 21831 |
| Mefenamic acid | 75569 |
| Mefenamic acid | 34438 |
| Mefenamic acid | 48810 |
| Mefenamic acid | 126 |
| Mefenamic acid | 66452 |
| Mefenamic acid | 75154 |
| Mefenamic acid | 32105 |
| Meloxicam | 61235 |
| Meloxicam | 1469 |
| Meloxicam | 850 |
| Meloxicam | 57370 |
| Meloxicam | 2243 |
| Meloxicam | 71989 |
| Meloxicam | 1470 |
| Meloxicam | 60705 |
| Meloxicam | 76191 |
| Meloxicam | 57475 |
| Meloxicam | 76595 |
| Meloxicam | 35935 |
| Meloxicam | 66364 |
| Meloxicam | 79307 |
| Meloxicam | 56275 |
| Meloxicam | 68932 |
| Meloxicam | 78629 |
| Meloxicam | 77260 |
| Meptazinol | 3239 |
| Meptazinol | 8447 |
| Meptazinol | 39842 |
| Morphine | 27749 |
| Morphine | 53639 |
| Morphine | 9183 |
| Morphine | 12889 |
| Morphine | 13711 |
| Morphine | 73009 |
| Morphine | 5652 |
| Morphine | 8460 |
| Morphine | 9371 |
| Morphine | 29591 |
| Morphine | 9960 |
| Morphine | 28837 |
| Morphine | 12608 |
| Morphine | 14063 |
| Morphine | 58279 |
| Morphine | 5991 |
| Morphine | 47985 |
| Morphine | 29019 |
| Morphine | 5777 |
| Morphine | 8959 |
| Morphine | 24453 |
| Morphine | 55052 |
| Morphine | 29970 |
| Morphine | 23060 |
| Morphine | 20008 |
| Morphine | 15339 |
| Morphine | 17893 |
| Morphine | 8867 |
| Morphine | 9557 |
| Morphine | 29379 |
| Morphine | 18468 |
| Morphine | 42380 |
| Morphine | 63593 |
| Morphine | 5555 |
| Morphine | 5681 |
| Morphine | 31599 |
| Morphine | 43657 |
| Morphine | 19317 |
| Morphine | 18792 |
| Morphine | 56788 |
| Morphine | 19738 |
| Morphine | 20219 |
| Morphine | 6366 |
| Morphine | 2957 |
| Morphine | 17271 |
| Morphine | 18700 |
| Morphine | 11838 |
| Morphine | 27298 |
| Morphine | 24840 |
| Morphine | 13588 |
| Morphine | 8220 |
| Morphine | 18734 |
| Morphine | 27058 |
| Morphine | 495 |
| Morphine | 12604 |
| Morphine | 7875 |
| Morphine | 61423 |
| Morphine | 18977 |
| Morphine | 78584 |
| Morphine | 32687 |
| Morphine | 54520 |
| Morphine | 21972 |
| Morphine | 12900 |
| Morphine | 18801 |
| Morphine | 5714 |
| Morphine | 26805 |
| Morphine | 22756 |
| Morphine | 8823 |
| Morphine | 30252 |
| Morphine | 17092 |
| Morphine | 30597 |
| Morphine | 8766 |
| Morphine | 58879 |
| Morphine | 34477 |
| Morphine | 23775 |
| Morphine | 13995 |
| Morphine | 13420 |
| Morphine | 14050 |
| Morphine | 8866 |
| Morphine | 47154 |
| Morphine | 72225 |
| Morphine | 19351 |
| Morphine | 12508 |
| Morphine | 2997 |
| Morphine | 47753 |
| Morphine | 40563 |
| Morphine | 18656 |
| Morphine | 17163 |
| Morphine | 4266 |
| Morphine | 15964 |
| Morphine | 7801 |
| Morphine | 26284 |
| Morphine | 18881 |
| Morphine | 60518 |
| Morphine | 19092 |
| Morphine | 7114 |
| Morphine | 2425 |
| Morphine | 607 |
| Morphine | 14226 |
| Morphine | 10907 |
| Morphine | 11698 |
| Morphine | 16273 |
| Morphine | 34073 |
| Morphine | 26407 |
| Morphine | 6002 |
| Morphine | 31407 |
| Morphine | 10749 |
| Morphine | 17936 |
| Morphine | 7181 |
| Morphine | 23581 |
| Morphine | 26283 |
| Morphine | 6232 |
| Morphine | 55206 |
| Morphine | 19477 |
| Morphine | 32459 |
| Morphine | 18639 |
| Morphine | 17825 |
| Morphine | 17943 |
| Morphine | 11755 |
| Morphine | 28503 |
| Morphine | 22622 |
| Morphine | 23128 |
| Morphine | 22026 |
| Morphine | 8735 |
| Morphine | 78234 |
| Morphine | 58215 |
| Morphine | 13997 |
| Morphine | 25503 |
| Morphine | 21868 |
| Morphine | 4693 |
| Morphine | 10239 |
| Morphine | 22690 |
| Morphine | 4280 |
| Morphine | 22051 |
| Morphine | 5840 |
| Morphine | 10473 |
| Morphine | 47949 |
| Morphine | 24808 |
| Morphine | 20752 |
| Morphine | 23777 |
| Morphine | 7197 |
| Morphine | 7729 |
| Morphine | 29592 |
| Morphine | 11342 |
| Morphine | 9945 |
| Morphine | 6736 |
| Morphine | 9337 |
| Morphine | 29500 |
| Morphine | 32520 |
| Morphine | 54017 |
| Morphine | 9484 |
| Morphine | 27441 |
| Morphine | 24424 |
| Morphine | 14156 |
| Morphine | 13117 |
| Morphine | 31033 |
| Morphine | 655 |
| Morphine | 68712 |
| Morphine | 20713 |
| Morphine | 8876 |
| Morphine | 31044 |
| Morphine | 10631 |
| Morphine | 19449 |
| Morphine | 47867 |
| Morphine | 30761 |
| Morphine | 12591 |
| Morphine | 22024 |
| Morphine | 15781 |
| Morphine | 29020 |
| Morphine | 7872 |
| Morphine | 9672 |
| Morphine | 9342 |
| Morphine | 23785 |
| Morphine | 19291 |
| Morphine | 15950 |
| Morphine | 1503 |
| Morphine | 27436 |
| Morphine | 8420 |
| Morphine | 59584 |
| Morphine | 24697 |
| Morphine | 27352 |
| Morphine | 4477 |
| Morphine | 9602 |
| Morphine | 43652 |
| Morphine | 15064 |
| Morphine | 25650 |
| Morphine | 8039 |
| Morphine | 18727 |
| Morphine | 5563 |
| Morphine | 9126 |
| Morphine | 32460 |
| Morphine | 25830 |
| Morphine | 13364 |
| Morphine | 23778 |
| Morphine | 45736 |
| Morphine | 24816 |
| Morphine | 3919 |
| Morphine | 32357 |
| Morphine | 27338 |
| Morphine | 23063 |
| Morphine | 15815 |
| Morphine | 9137 |
| Morphine | 58499 |
| Morphine | 18965 |
| Morphine | 26144 |
| Morphine | 28711 |
| Morphine | 13423 |
| Morphine | 148 |
| Morphine | 15514 |
| Morphine | 28396 |
| Morphine | 4476 |
| Morphine | 18166 |
| Morphine | 11971 |
| Morphine | 12583 |
| Morphine | 60950 |
| Morphine | 64417 |
| Morphine | 8822 |
| Morphine | 6269 |
| Morphine | 25649 |
| Morphine | 30320 |
| Morphine | 13114 |
| Morphine | 79003 |
| Morphine | 31960 |
| Morphine | 19471 |
| Morphine | 9381 |
| Morphine | 18626 |
| Morphine | 12011 |
| Morphine | 53273 |
| Nabumetone | 42821 |
| Nabumetone | 64297 |
| Nabumetone | 13818 |
| Nabumetone | 10295 |
| Nabumetone | 11466 |
| Nabumetone | 2235 |
| Nabumetone | 16473 |
| Nabumetone | 77421 |
| Nabumetone | 73655 |
| Nabumetone | 2234 |
| Nabumetone | 16474 |
| Naproxen | 25342 |
| Naproxen | 4045 |
| Naproxen | 66993 |
| Naproxen | 34923 |
| Naproxen | 2391 |
| Naproxen | 27366 |
| Naproxen | 65952 |
| Naproxen | 34743 |
| Naproxen | 70698 |
| Naproxen | 46440 |
| Naproxen | 59878 |
| Naproxen | 1866 |
| Naproxen | 28255 |
| Naproxen | 20704 |
| Naproxen | 20385 |
| Naproxen | 68470 |
| Naproxen | 69645 |
| Naproxen | 56762 |
| Naproxen | 79577 |
| Naproxen | 25750 |
| Naproxen | 52931 |
| Naproxen | 72030 |
| Naproxen | 21816 |
| Naproxen | 76869 |
| Naproxen | 26231 |
| Naproxen | 78172 |
| Naproxen | 661 |
| Naproxen | 39317 |
| Naproxen | 31429 |
| Naproxen | 73961 |
| Naproxen | 39693 |
| Naproxen | 3431 |
| Naproxen | 54304 |
| Naproxen | 56925 |
| Naproxen | 55505 |
| Naproxen | 38511 |
| Naproxen | 67117 |
| Naproxen | 26242 |
| Naproxen | 65348 |
| Naproxen | 23323 |
| Naproxen | 3901 |
| Naproxen | 63357 |
| Naproxen | 76073 |
| Naproxen | 39085 |
| Naproxen | 34738 |
| Naproxen | 3496 |
| Naproxen | 51360 |
| Naproxen | 58213 |
| Naproxen | 73629 |
| Naproxen | 78343 |
| Naproxen | 5268 |
| Naproxen | 4320 |
| Naproxen | 3432 |
| Naproxen | 21843 |
| Naproxen | 70393 |
| Naproxen | 25092 |
| Naproxen | 67768 |
| Naproxen | 2288 |
| Naproxen | 67363 |
| Naproxen | 33111 |
| Naproxen | 34769 |
| Naproxen | 56106 |
| Naproxen | 24007 |
| Naproxen | 2197 |
| Naproxen | 46848 |
| Naproxen | 54783 |
| Naproxen | 78985 |
| Naproxen | 30982 |
| Naproxen | 47994 |
| Naproxen | 65862 |
| Naproxen | 8663 |
| Naproxen | 34922 |
| Naproxen | 44986 |
| Naproxen | 31945 |
| Naproxen | 63843 |
| Naproxen | 56554 |
| Naproxen | 80088 |
| Naproxen | 59246 |
| Naproxen | 77951 |
| Naproxen | 17733 |
| Naproxen | 34143 |
| Naproxen | 60408 |
| Naproxen | 70828 |
| Naproxen | 75095 |
| Naproxen | 15180 |
| Naproxen | 68685 |
| Naproxen | 28313 |
| Naproxen | 51242 |
| Naproxen | 40401 |
| Naproxen | 53626 |
| Naproxen | 15104 |
| Naproxen | 34610 |
| Naproxen | 55486 |
| Naproxen | 53700 |
| Naproxen | 69828 |
| Naproxen | 71709 |
| Naproxen | 53980 |
| Naproxen | 17165 |
| Naproxen | 60115 |
| Naproxen | 72047 |
| Naproxen | 34289 |
| Naproxen | 45262 |
| Naproxen | 28816 |
| Naproxen | 77680 |
| Naproxen | 26216 |
| Naproxen | 34977 |
| Naproxen | 55894 |
| Naproxen | 48161 |
| Naproxen | 3817 |
| Naproxen | 76404 |
| Naproxen | 19007 |
| Naproxen | 23121 |
| Naproxen | 51829 |
| Naproxen | 15023 |
| Naproxen | 807 |
| Naproxen | 58708 |
| Naproxen | 21840 |
| Naproxen | 34670 |
| Naproxen | 25341 |
| Naproxen | 44800 |
| Naproxen | 58221 |
| Naproxen | 30168 |
| Naproxen | 55454 |
| Naproxen | 3053 |
| Naproxen | 34290 |
| Naproxen | 68097 |
| Naproxen | 76955 |
| Naproxen | 61695 |
| Naproxen | 1043 |
| Naproxen | 5407 |
| Naproxen | 3972 |
| Naproxen | 24020 |
| Naproxen | 54476 |
| Nefopam hydrochloride | 4016 |
| Nefopam hydrochloride | 68271 |
| Nefopam hydrochloride | 64313 |
| Nefopam hydrochloride | 66814 |
| Nefopam hydrochloride | 65308 |
| Nefopam hydrochloride | 70428 |
| Nefopam hydrochloride | 37979 |
| Nefopam hydrochloride | 3794 |
| Oxycodone hydrochloride | 58039 |
| Oxycodone hydrochloride | 6557 |
| Oxycodone hydrochloride | 76895 |
| Oxycodone hydrochloride | 7275 |
| Oxycodone hydrochloride | 7372 |
| Oxycodone hydrochloride | 6609 |
| Oxycodone hydrochloride | 51384 |
| Oxycodone hydrochloride | 45929 |
| Oxycodone hydrochloride | 64333 |
| Oxycodone hydrochloride | 65392 |
| Oxycodone hydrochloride | 73428 |
| Oxycodone hydrochloride | 54694 |
| Oxycodone hydrochloride | 79802 |
| Oxycodone hydrochloride | 80228 |
| Oxycodone hydrochloride | 9874 |
| Oxycodone hydrochloride | 74294 |
| Oxycodone hydrochloride | 52592 |
| Oxycodone hydrochloride | 62322 |
| Oxycodone hydrochloride | 7389 |
| Oxycodone hydrochloride | 61779 |
| Oxycodone hydrochloride | 74549 |
| Oxycodone hydrochloride | 69559 |
| Oxycodone hydrochloride | 61936 |
| Oxycodone hydrochloride | 76649 |
| Oxycodone hydrochloride | 64965 |
| Oxycodone hydrochloride | 75624 |
| Oxycodone hydrochloride | 58493 |
| Oxycodone hydrochloride | 65932 |
| Oxycodone hydrochloride | 7167 |
| Oxycodone hydrochloride | 53113 |
| Oxycodone hydrochloride | 49742 |
| Oxycodone hydrochloride | 66619 |
| Oxycodone hydrochloride | 65933 |
| Oxycodone hydrochloride | 39475 |
| Oxycodone hydrochloride | 45790 |
| Oxycodone hydrochloride | 61836 |
| Oxycodone hydrochloride | 66606 |
| Oxycodone hydrochloride | 74909 |
| Oxycodone hydrochloride | 11405 |
| Oxycodone hydrochloride | 66837 |
| Oxycodone hydrochloride | 76692 |
| Oxycodone hydrochloride | 74910 |
| Oxycodone hydrochloride | 6708 |
| Oxycodone hydrochloride | 75369 |
| Oxycodone hydrochloride | 53116 |
| Oxycodone hydrochloride | 58853 |
| Oxycodone hydrochloride | 80226 |
| Oxycodone hydrochloride | 6608 |
| Oxycodone hydrochloride | 76698 |
| Oxycodone hydrochloride | 63714 |
| Oxycodone hydrochloride | 52217 |
| Oxycodone hydrochloride | 68797 |
| Oxycodone hydrochloride | 77740 |
| Oxycodone hydrochloride | 45788 |
| Oxycodone hydrochloride | 50095 |
| Oxycodone hydrochloride | 61935 |
| Oxycodone hydrochloride | 45745 |
| Oxycodone hydrochloride | 45766 |
| Oxycodone hydrochloride | 79584 |
| Oxycodone hydrochloride | 64807 |
| Oxycodone hydrochloride | 80227 |
| Oxycodone hydrochloride | 79112 |
| Oxycodone hydrochloride | 66616 |
| Oxycodone hydrochloride | 45830 |
| Oxycodone hydrochloride | 76236 |
| Oxycodone hydrochloride | 52216 |
| Oxycodone hydrochloride | 51896 |
| Oxycodone hydrochloride | 10021 |
| Oxycodone hydrochloride | 77709 |
| Oxycodone hydrochloride | 76699 |
| Oxycodone hydrochloride | 64150 |
| Oxycodone hydrochloride | 56665 |
| Oxycodone hydrochloride | 27548 |
| Oxycodone hydrochloride | 6769 |
| Oxycodone hydrochloride | 60196 |
| Oxycodone hydrochloride | 58114 |
| Oxycodone hydrochloride | 75283 |
| Oxycodone hydrochloride | 64164 |
| Oxycodone hydrochloride | 76394 |
| Oxycodone hydrochloride | 78005 |
| Oxycodone hydrochloride | 52809 |
| Oxycodone hydrochloride | 60158 |
| Oxycodone hydrochloride | 7406 |
| Oxycodone hydrochloride | 75980 |
| Oxycodone hydrochloride | 46187 |
| Oxycodone hydrochloride | 67446 |
| Oxycodone hydrochloride | 69474 |
| Oxycodone hydrochloride | 75357 |
| Oxycodone hydrochloride | 50733 |
| Oxycodone hydrochloride | 63198 |
| Oxycodone hydrochloride | 76755 |
| Oxycodone hydrochloride | 9973 |
| Oxycodone hydrochloride | 79815 |
| Oxycodone hydrochloride | 74908 |
| Oxycodone hydrochloride | 73841 |
| Oxycodone hydrochloride | 6790 |
| Oxycodone hydrochloride | 6948 |
| Oxycodone hydrochloride | 78941 |
| Oxycodone hydrochloride | 57052 |
| Oxycodone hydrochloride | 77879 |
| Oxycodone hydrochloride | 5585 |
| Oxycodone hydrochloride | 60146 |
| Oxycodone hydrochloride | 45827 |
| Oxycodone hydrochloride | 64552 |
| Oxycodone hydrochloride | 39498 |
| Oxycodone hydrochloride | 9927 |
| Oxycodone hydrochloride | 49787 |
| Oxycodone hydrochloride | 66298 |
| Oxycodone hydrochloride | 65390 |
| Oxycodone hydrochloride | 40616 |
| Oxycodone hydrochloride | 63332 |
| Oxycodone hydrochloride | 77078 |
| Oxycodone hydrochloride | 49940 |
| Oxycodone hydrochloride | 52220 |
| Oxycodone hydrochloride | 59865 |
| Oxycodone hydrochloride | 64426 |
| Oxycodone hydrochloride | 40785 |
| Oxycodone hydrochloride | 78942 |
| Oxycodone hydrochloride | 57033 |
| Oxycodone hydrochloride | 71335 |
| Oxycodone hydrochloride | 51789 |
| Oxycodone hydrochloride | 58217 |
| Oxycodone hydrochloride | 66760 |
| Oxycodone hydrochloride | 5843 |
| Oxycodone hydrochloride | 5599 |
| Oxycodone hydrochloride | 69993 |
| Papaveretum | 685 |
| Papaveretum | 6226 |
| Papaveretum | 166 |
| Papaveretum | 19764 |
| Papaveretum | 18261 |
| Paracetamol | 9712 |
| Paracetamol | 34266 |
| Paracetamol | 66097 |
| Paracetamol | 32672 |
| Paracetamol | 27804 |
| Paracetamol | 56877 |
| Paracetamol | 29951 |
| Paracetamol | 43233 |
| Paracetamol | 8020 |
| Paracetamol | 33230 |
| Paracetamol | 855 |
| Paracetamol | 39291 |
| Paracetamol | 54179 |
| Paracetamol | 8093 |
| Paracetamol | 7621 |
| Paracetamol | 33642 |
| Paracetamol | 35888 |
| Paracetamol | 10123 |
| Paracetamol | 48178 |
| Paracetamol | 30590 |
| Paracetamol | 8700 |
| Paracetamol | 59599 |
| Paracetamol | 34700 |
| Paracetamol | 33710 |
| Paracetamol | 30994 |
| Paracetamol | 48444 |
| Paracetamol | 24133 |
| Paracetamol | 22085 |
| Paracetamol | 35967 |
| Paracetamol | 9476 |
| Paracetamol | 22062 |
| Paracetamol | 1147 |
| Paracetamol | 34319 |
| Paracetamol | 58214 |
| Paracetamol | 15845 |
| Paracetamol | 34209 |
| Paracetamol | 44258 |
| Paracetamol | 24075 |
| Paracetamol | 42371 |
| Paracetamol | 48354 |
| Paracetamol | 27446 |
| Paracetamol | 20068 |
| Paracetamol | 33386 |
| Paracetamol | 34409 |
| Paracetamol | 34761 |
| Paracetamol | 23274 |
| Paracetamol | 63908 |
| Paracetamol | 12332 |
| Paracetamol | 57092 |
| Paracetamol | 26988 |
| Paracetamol | 36754 |
| Paracetamol | 15439 |
| Paracetamol | 57433 |
| Paracetamol | 52948 |
| Paracetamol | 4186 |
| Paracetamol | 15352 |
| Paracetamol | 27459 |
| Paracetamol | 11925 |
| Paracetamol | 12394 |
| Paracetamol | 48816 |
| Paracetamol | 29558 |
| Paracetamol | 8259 |
| Paracetamol | 12778 |
| Paracetamol | 1404 |
| Paracetamol | 42345 |
| Paracetamol | 17412 |
| Paracetamol | 56945 |
| Paracetamol | 124 |
| Paracetamol | 58857 |
| Paracetamol | 28636 |
| Paracetamol | 48129 |
| Paracetamol | 27197 |
| Paracetamol | 41142 |
| Paracetamol | 16106 |
| Paracetamol | 22288 |
| Paracetamol | 28712 |
| Paracetamol | 35889 |
| Paracetamol | 26295 |
| Paracetamol | 24283 |
| Paracetamol | 48301 |
| Paracetamol | 1609 |
| Paracetamol | 32963 |
| Paracetamol | 12869 |
| Paracetamol | 58631 |
| Paracetamol | 62372 |
| Paracetamol | 21491 |
| Paracetamol | 32178 |
| Paracetamol | 11614 |
| Paracetamol | 13193 |
| Paracetamol | 20495 |
| Paracetamol | 42201 |
| Paracetamol | 49219 |
| Paracetamol | 62323 |
| Paracetamol | 10196 |
| Paracetamol | 7489 |
| Paracetamol | 45276 |
| Paracetamol | 34858 |
| Paracetamol | 10901 |
| Paracetamol | 53208 |
| Paracetamol | 7650 |
| Paracetamol | 42834 |
| Paracetamol | 36929 |
| Paracetamol | 58743 |
| Paracetamol | 23052 |
| Paracetamol | 18799 |
| Paracetamol | 9914 |
| Paracetamol | 15367 |
| Paracetamol | 79319 |
| Paracetamol | 32609 |
| Paracetamol | 46498 |
| Paracetamol | 24480 |
| Paracetamol | 28344 |
| Paracetamol | 901 |
| Paracetamol | 31257 |
| Paracetamol | 34554 |
| Paracetamol | 17773 |
| Paracetamol | 39739 |
| Paracetamol | 62257 |
| Paracetamol | 65107 |
| Paracetamol | 47026 |
| Paracetamol | 43536 |
| Paracetamol | 30966 |
| Paracetamol | 27337 |
| Paracetamol | 27128 |
| Paracetamol | 40880 |
| Paracetamol | 25562 |
| Paracetamol | 18675 |
| Paracetamol | 39656 |
| Paracetamol | 60442 |
| Paracetamol | 28236 |
| Paracetamol | 71270 |
| Paracetamol | 75935 |
| Paracetamol | 68714 |
| Paracetamol | 75966 |
| Paracetamol | 44369 |
| Paracetamol | 23841 |
| Paracetamol | 67370 |
| Paracetamol | 54737 |
| Paracetamol | 45298 |
| Paracetamol | 66481 |
| Paracetamol | 32970 |
| Paracetamol | 41414 |
| Paracetamol | 44984 |
| Paracetamol | 27979 |
| Paracetamol | 32839 |
| Paracetamol | 14384 |
| Paracetamol | 67755 |
| Paracetamol | 36720 |
| Paracetamol | 24400 |
| Paracetamol | 40158 |
| Paracetamol | 21370 |
| Paracetamol | 77370 |
| Paracetamol | 7205 |
| Paracetamol | 14560 |
| Paracetamol | 45469 |
| Paracetamol | 50504 |
| Paracetamol | 18482 |
| Paracetamol | 34396 |
| Paracetamol | 77663 |
| Paracetamol | 66664 |
| Paracetamol | 37607 |
| Paracetamol | 3714 |
| Paracetamol | 28346 |
| Paracetamol | 46846 |
| Paracetamol | 36598 |
| Paracetamol | 483 |
| Paracetamol | 48166 |
| Paracetamol | 65299 |
| Paracetamol | 51595 |
| Paracetamol | 8930 |
| Paracetamol | 25941 |
| Paracetamol | 17651 |
| Paracetamol | 57650 |
| Paracetamol | 46638 |
| Paracetamol | 23107 |
| Paracetamol | 22305 |
| Paracetamol | 3073 |
| Paracetamol | 24445 |
| Paracetamol | 48597 |
| Paracetamol | 55817 |
| Paracetamol | 30958 |
| Paracetamol | 46876 |
| Paracetamol | 14627 |
| Paracetamol | 63543 |
| Paracetamol | 43891 |
| Paracetamol | 14551 |
| Paracetamol | 32554 |
| Paracetamol | 74576 |
| Paracetamol | 175 |
| Paracetamol | 17845 |
| Paracetamol | 31151 |
| Paracetamol | 14829 |
| Paracetamol | 45497 |
| Paracetamol | 27553 |
| Paracetamol | 38299 |
| Paracetamol | 30070 |
| Paracetamol | 39940 |
| Paracetamol | 34468 |
| Paracetamol | 10748 |
| Paracetamol | 68857 |
| Paracetamol | 11986 |
| Paracetamol | 45649 |
| Paracetamol | 20403 |
| Paracetamol | 43479 |
| Paracetamol | 10399 |
| Paracetamol | 77117 |
| Paracetamol | 33104 |
| Paracetamol | 61851 |
| Paracetamol | 22125 |
| Paracetamol | 35800 |
| Paracetamol | 46895 |
| Paracetamol | 68738 |
| Paracetamol | 34669 |
| Paracetamol | 30116 |
| Paracetamol | 4 |
| Paracetamol | 36614 |
| Paracetamol | 79236 |
| Paracetamol | 46830 |
| Paracetamol | 43447 |
| Paracetamol | 26967 |
| Paracetamol | 33042 |
| Paracetamol | 27452 |
| Paracetamol | 22988 |
| Paracetamol | 62743 |
| Paracetamol | 74500 |
| Paracetamol | 33614 |
| Paracetamol | 61124 |
| Paracetamol | 65683 |
| Paracetamol | 74171 |
| Paracetamol | 4607 |
| Paracetamol | 70018 |
| Paracetamol | 78357 |
| Paracetamol | 37411 |
| Paracetamol | 9239 |
| Paracetamol | 62491 |
| Paracetamol | 24803 |
| Paracetamol | 14151 |
| Paracetamol | 35885 |
| Paracetamol | 23909 |
| Paracetamol | 64125 |
| Paracetamol | 46763 |
| Paracetamol | 22236 |
| Paracetamol | 63943 |
| Paracetamol | 38984 |
| Paracetamol | 890 |
| Paracetamol | 38943 |
| Paracetamol | 33060 |
| Paracetamol | 50166 |
| Paracetamol | 59773 |
| Paracetamol | 20116 |
| Paracetamol | 29670 |
| Paracetamol | 30984 |
| Paracetamol | 26133 |
| Paracetamol | 23318 |
| Paracetamol | 41435 |
| Paracetamol | 45259 |
| Paracetamol | 24534 |
| Paracetamol | 39934 |
| Paracetamol | 27492 |
| Paracetamol | 8091 |
| Paracetamol | 18583 |
| Paracetamol | 34350 |
| Paracetamol | 58526 |
| Paracetamol | 47920 |
| Paracetamol | 24000 |
| Paracetamol | 49105 |
| Paracetamol | 49314 |
| Paracetamol | 32163 |
| Paracetamol | 42915 |
| Paracetamol | 27145 |
| Paracetamol | 15238 |
| Paracetamol | 65330 |
| Paracetamol | 50881 |
| Paracetamol | 38122 |
| Paracetamol | 25895 |
| Paracetamol | 68070 |
| Paracetamol | 10293 |
| Paracetamol | 72288 |
| Paracetamol | 3514 |
| Paracetamol | 43252 |
| Paracetamol | 22014 |
| Paracetamol | 37446 |
| Paracetamol | 39333 |
| Paracetamol | 69043 |
| Paracetamol | 28211 |
| Paracetamol | 2546 |
| Paracetamol | 62088 |
| Paracetamol | 49575 |
| Paracetamol | 30954 |
| Paracetamol | 28955 |
| Paracetamol | 9329 |
| Paracetamol | 72131 |
| Paracetamol | 67784 |
| Paracetamol | 39647 |
| Paracetamol | 23840 |
| Paracetamol | 39629 |
| Paracetamol | 55245 |
| Paracetamol | 9193 |
| Paracetamol | 23617 |
| Paracetamol | 31196 |
| Paracetamol | 40107 |
| Paracetamol | 2055 |
| Paracetamol | 28253 |
| Paracetamol | 48295 |
| Paracetamol | 43199 |
| Paracetamol | 28946 |
| Paracetamol | 49849 |
| Paracetamol | 55418 |
| Paracetamol | 33826 |
| Paracetamol | 61963 |
| Paracetamol | 892 |
| Paracetamol | 8292 |
| Paracetamol | 32093 |
| Paracetamol | 2800 |
| Paracetamol | 64073 |
| Paracetamol | 41680 |
| Paracetamol | 34954 |
| Paracetamol | 48561 |
| Paracetamol | 79619 |
| Paracetamol | 34500 |
| Paracetamol | 53952 |
| Paracetamol | 52456 |
| Paracetamol | 25607 |
| Paracetamol | 27064 |
| Paracetamol | 32993 |
| Paracetamol | 74869 |
| Paracetamol | 48330 |
| Paracetamol | 46544 |
| Paracetamol | 36945 |
| Paracetamol | 5239 |
| Paracetamol | 45645 |
| Paracetamol | 34718 |
| Paracetamol | 23114 |
| Paracetamol | 39481 |
| Paracetamol | 78301 |
| Paracetamol | 7668 |
| Paracetamol | 38032 |
| Paracetamol | 49524 |
| Paracetamol | 49096 |
| Paracetamol | 27837 |
| Paracetamol | 29375 |
| Paracetamol | 20627 |
| Paracetamol | 31499 |
| Paracetamol | 35679 |
| Paracetamol | 59801 |
| Paracetamol | 65080 |
| Paracetamol | 29074 |
| Paracetamol | 34305 |
| Paracetamol | 7 |
| Paracetamol | 32942 |
| Paracetamol | 8139 |
| Paracetamol | 54853 |
| Paracetamol | 9231 |
| Paracetamol | 68434 |
| Paracetamol | 34597 |
| Paracetamol | 4633 |
| Paracetamol | 70326 |
| Paracetamol | 3316 |
| Paracetamol | 48622 |
| Paracetamol | 1762 |
| Paracetamol | 54353 |
| Paracetamol | 32184 |
| Paracetamol | 31998 |
| Paracetamol | 50482 |
| Paracetamol | 23108 |
| Paracetamol | 1946 |
| Paracetamol | 2586 |
| Paracetamol | 40624 |
| Paracetamol | 64932 |
| Paracetamol | 41407 |
| Paracetamol | 43028 |
| Paracetamol | 1862 |
| Paracetamol | 55951 |
| Paracetamol | 12447 |
| Paracetamol | 26294 |
| Paracetamol | 27435 |
| Paracetamol | 57425 |
| Paracetamol | 42514 |
| Paracetamol | 60812 |
| Paracetamol | 7769 |
| Paracetamol | 61485 |
| Paracetamol | 19626 |
| Paracetamol | 32681 |
| Paracetamol | 3313 |
| Paracetamol | 40948 |
| Paracetamol | 27856 |
| Paracetamol | 21770 |
| Paracetamol | 56046 |
| Paracetamol | 72796 |
| Paracetamol | 3074 |
| Paracetamol | 43204 |
| Paracetamol | 47211 |
| Paracetamol | 55290 |
| Paracetamol | 1689 |
| Paracetamol | 19917 |
| Paracetamol | 1865 |
| Paracetamol | 34022 |
| Paracetamol | 43554 |
| Paracetamol | 28937 |
| Paracetamol | 73478 |
| Paracetamol | 52455 |
| Paracetamol | 61522 |
| Paracetamol | 15241 |
| Paracetamol | 50984 |
| Paracetamol | 33687 |
| Paracetamol | 45231 |
| Paracetamol | 23075 |
| Paracetamol | 2306 |
| Paracetamol | 34546 |
| Paracetamol | 47116 |
| Paracetamol | 32626 |
| Paracetamol | 71178 |
| Paracetamol | 21754 |
| Paracetamol | 39893 |
| Paracetamol | 10993 |
| Paracetamol | 29401 |
| Paracetamol | 9581 |
| Paracetamol | 62305 |
| Paracetamol | 20194 |
| Paracetamol | 24309 |
| Paracetamol | 44537 |
| Paracetamol | 56566 |
| Paracetamol | 19255 |
| Paracetamol | 75720 |
| Paracetamol | 33666 |
| Paracetamol | 79079 |
| Paracetamol | 14655 |
| Paracetamol | 32035 |
| Paracetamol | 38323 |
| Paracetamol | 139 |
| Paracetamol | 50009 |
| Paracetamol | 44977 |
| Paracetamol | 24947 |
| Paracetamol | 20646 |
| Paracetamol | 26746 |
| Paracetamol | 64154 |
| Paracetamol | 47834 |
| Paracetamol | 49954 |
| Paracetamol | 1068 |
| Paracetamol | 262 |
| Paracetamol | 78114 |
| Paracetamol | 26572 |
| Paracetamol | 4203 |
| Paracetamol | 20650 |
| Paracetamol | 48157 |
| Paracetamol | 75375 |
| Paracetamol | 2462 |
| Paracetamol | 18922 |
| Paracetamol | 22073 |
| Paracetamol | 899 |
| Paracetamol | 759 |
| Paracetamol | 258 |
| Paracetamol | 23716 |
| Paracetamol | 28792 |
| Paracetamol | 4600 |
| Paracetamol | 48535 |
| Paracetamol | 34164 |
| Paracetamol | 33995 |
| Paracetamol | 44622 |
| Paracetamol | 50232 |
| Paracetamol | 15461 |
| Paracetamol | 65239 |
| Paracetamol | 44632 |
| Paracetamol | 45348 |
| Paracetamol | 31717 |
| Paracetamol | 9271 |
| Paracetamol | 49417 |
| Paracetamol | 60378 |
| Paracetamol | 7520 |
| Paracetamol | 28878 |
| Paracetamol | 59823 |
| Paracetamol | 69534 |
| Paracetamol | 18919 |
| Paracetamol | 34349 |
| Paracetamol | 67887 |
| Paracetamol | 28237 |
| Paracetamol | 63962 |
| Paracetamol | 37571 |
| Paracetamol | 55129 |
| Paracetamol | 16058 |
| Paracetamol | 58582 |
| Paracetamol | 53984 |
| Paracetamol | 65650 |
| Paracetamol | 23077 |
| Paracetamol | 71545 |
| Paracetamol | 6571 |
| Paracetamol | 67086 |
| Paracetamol | 34397 |
| Paracetamol | 33647 |
| Paracetamol | 33838 |
| Paracetamol | 34678 |
| Pentazocine | 328 |
| Pentazocine | 36472 |
| Pentazocine | 2367 |
| Pentazocine | 78223 |
| Pentazocine | 10509 |
| Pentazocine | 7450 |
| Pentazocine | 71170 |
| Pentazocine | 10769 |
| Pentazocine | 8375 |
| Pentazocine | 79369 |
| Pethidine hydrochloride | 234 |
| Pethidine hydrochloride | 31935 |
| Pethidine hydrochloride | 17386 |
| Pethidine hydrochloride | 30319 |
| Pethidine hydrochloride | 53709 |
| Pethidine hydrochloride | 54085 |
| Pethidine hydrochloride | 38103 |
| Pethidine hydrochloride | 57027 |
| Pethidine hydrochloride | 38013 |
| Pethidine hydrochloride | 56022 |
| Pethidine hydrochloride | 54790 |
| Pethidine hydrochloride | 2450 |
| Pethidine hydrochloride | 40239 |
| Pethidine hydrochloride | 22896 |
| Pethidine hydrochloride | 58737 |
| Piroxicam | 20663 |
| Piroxicam | 31777 |
| Piroxicam | 57523 |
| Piroxicam | 21123 |
| Piroxicam | 4965 |
| Piroxicam | 61314 |
| Piroxicam | 11495 |
| Piroxicam | 29465 |
| Piroxicam | 341 |
| Piroxicam | 62375 |
| Piroxicam | 67815 |
| Piroxicam | 2827 |
| Piroxicam | 3710 |
| Piroxicam | 141 |
| Piroxicam | 1544 |
| Piroxicam | 54870 |
| Piroxicam | 10169 |
| Piroxicam | 65594 |
| Piroxicam | 43541 |
| Piroxicam | 28695 |
| Piroxicam | 34616 |
| Piroxicam | 1755 |
| Piroxicam | 26234 |
| Piroxicam | 21864 |
| Piroxicam | 72544 |
| Piroxicam | 20699 |
| Piroxicam | 20742 |
| Piroxicam | 41622 |
| Piroxicam | 56651 |
| Piroxicam | 19320 |
| Piroxicam | 58298 |
| Piroxicam | 9630 |
| Piroxicam | 41621 |
| Piroxicam | 41624 |
| Piroxicam | 41623 |
| Piroxicam | 3935 |
| Piroxicam | 74803 |
| Piroxicam | 21846 |
| Piroxicam | 2858 |
| Piroxicam | 74343 |
| Piroxicam | 37750 |
| Piroxicam | 73981 |
| Piroxicam | 39109 |
| Piroxicam | 2463 |
| Piroxicam | 48071 |
| Piroxicam | 8882 |
| Piroxicam | 74659 |
| Piroxicam | 7524 |
| Piroxicam | 71027 |
| Piroxicam | 67608 |
| Piroxicam | 50343 |
| Piroxicam | 61715 |
| Piroxicam | 3409 |
| Piroxicam | 77185 |
| Piroxicam | 44703 |
| Piroxicam | 78260 |
| Piroxicam | 77694 |
| Sulindac | 13380 |
| Sulindac | 5482 |
| Sulindac | 7434 |
| Sulindac | 3897 |
| Tapentadol | 45811 |
| Tapentadol | 46461 |
| Tapentadol | 61764 |
| Tapentadol | 45800 |
| Tapentadol | 46022 |
| Tapentadol | 47460 |
| Tapentadol | 46021 |
| Tapentadol | 45982 |
| Tapentadol | 46159 |
| Tapentadol | 60759 |
| Tapentadol | 46020 |
| Tapentadol | 46019 |
| Tapentadol | 46659 |
| Tapentadol | 46018 |
| Tapentadol | 45936 |
| Tapentadol | 47399 |
| Targinact | 74780 |
| Targinact | 40645 |
| Targinact | 39478 |
| Targinact | 40961 |
| Targinact | 39477 |
| Tenoxicam | 24531 |
| Tenoxicam | 71152 |
| Tenoxicam | 47816 |
| Tenoxicam | 24682 |
| Tenoxicam | 12075 |
| Tenoxicam | 31064 |
| Tenoxicam | 42604 |
| Tenoxicam | 3974 |
| Tiaprofenic acid | 2863 |
| Tiaprofenic acid | 25643 |
| Tiaprofenic acid | 2257 |
| Tiaprofenic acid | 2382 |
| Tiaprofenic acid | 14776 |
| Tiaprofenic acid | 20059 |
| Tiaprofenic acid | 7913 |
| Tiaprofenic acid | 1778 |
| Tiaprofenic acid | 387 |
| Tolfenamic acid | 14994 |
| Tolfenamic acid | 78176 |
| Tolfenamic acid | 7222 |
| Tolfenamic acid | 20036 |
| Tolfenamic acid | 15159 |
| Tramacet | 62778 |
| Tramacet | 687 |
| Tramacet | 42332 |
| Tramadol hydrochloride | 16395 |
| Tramadol hydrochloride | 56491 |
| Tramadol hydrochloride | 73336 |
| Tramadol hydrochloride | 72646 |
| Tramadol hydrochloride | 74002 |
| Tramadol hydrochloride | 23625 |
| Tramadol hydrochloride | 16271 |
| Tramadol hydrochloride | 40805 |
| Tramadol hydrochloride | 9389 |
| Tramadol hydrochloride | 21797 |
| Tramadol hydrochloride | 40718 |
| Tramadol hydrochloride | 54023 |
| Tramadol hydrochloride | 58129 |
| Tramadol hydrochloride | 3378 |
| Tramadol hydrochloride | 74830 |
| Tramadol hydrochloride | 77642 |
| Tramadol hydrochloride | 65954 |
| Tramadol hydrochloride | 60751 |
| Tramadol hydrochloride | 73923 |
| Tramadol hydrochloride | 67744 |
| Tramadol hydrochloride | 38196 |
| Tramadol hydrochloride | 68833 |
| Tramadol hydrochloride | 78150 |
| Tramadol hydrochloride | 66299 |
| Tramadol hydrochloride | 34260 |
| Tramadol hydrochloride | 77356 |
| Tramadol hydrochloride | 4114 |
| Tramadol hydrochloride | 40060 |
| Tramadol hydrochloride | 34422 |
| Tramadol hydrochloride | 42280 |
| Tramadol hydrochloride | 37867 |
| Tramadol hydrochloride | 61272 |
| Tramadol hydrochloride | 43513 |
| Tramadol hydrochloride | 29324 |
| Tramadol hydrochloride | 75502 |
| Tramadol hydrochloride | 21777 |
| Tramadol hydrochloride | 67323 |
| Tramadol hydrochloride | 73890 |
| Tramadol hydrochloride | 13813 |
| Tramadol hydrochloride | 36873 |
| Tramadol hydrochloride | 37020 |
| Tramadol hydrochloride | 9739 |
| Tramadol hydrochloride | 75286 |
| Tramadol hydrochloride | 43198 |
| Tramadol hydrochloride | 39750 |
| Tramadol hydrochloride | 67197 |
| Tramadol hydrochloride | 11748 |
| Tramadol hydrochloride | 74887 |
| Tramadol hydrochloride | 52977 |
| Tramadol hydrochloride | 37831 |
| Tramadol hydrochloride | 69894 |
| Tramadol hydrochloride | 41976 |
| Tramadol hydrochloride | 86 |
| Tramadol hydrochloride | 64459 |
| Tramadol hydrochloride | 26336 |
| Tramadol hydrochloride | 5028 |
| Tramadol hydrochloride | 44371 |
| Tramadol hydrochloride | 11734 |
| Tramadol hydrochloride | 61775 |
| Tramadol hydrochloride | 66729 |
| Tramadol hydrochloride | 73069 |
| Tramadol hydrochloride | 32165 |
| Tramadol hydrochloride | 37021 |
| Tramadol hydrochloride | 11101 |
| Tramadol hydrochloride | 31734 |
| Tramadol hydrochloride | 75931 |
| Tramadol hydrochloride | 48090 |
| Tramadol hydrochloride | 52605 |
| Tramadol hydrochloride | 39798 |
| Tramadol hydrochloride | 80001 |
| Tramadol hydrochloride | 5169 |
| Tramadol hydrochloride | 5257 |
| Tramadol hydrochloride | 64871 |
| Tramadol hydrochloride | 64496 |
| Tramadol hydrochloride | 35806 |
| Tramadol hydrochloride | 23981 |
| Tramadol hydrochloride | 701 |
| Tramadol hydrochloride | 68210 |
| Tramadol hydrochloride | 21256 |
| Tramadol hydrochloride | 32450 |
| Tramadol hydrochloride | 40058 |
| Tramadol hydrochloride | 31107 |
| Tramadol hydrochloride | 46279 |
| Tramadol hydrochloride | 4115 |
| Tramadol hydrochloride | 4999 |
| Tramadol hydrochloride | 46643 |
| Tramadol hydrochloride | 14490 |
| Tramadol hydrochloride | 71358 |
| Tramadol hydrochloride | 29860 |
| Tramadol hydrochloride | 76224 |
| Tramadol hydrochloride | 3644 |
| Tramadol hydrochloride | 8416 |
| Tramadol hydrochloride | 21947 |
| Tramadol hydrochloride | 75864 |
| Tramadol hydrochloride | 78761 |
| Tramadol hydrochloride | 16076 |
| Tramadol hydrochloride | 35347 |
| Tramadol hydrochloride | 49323 |
| Tramadol hydrochloride | 58316 |
| Tramadol hydrochloride | 11746 |
| Tramadol hydrochloride | 50862 |
| Tramadol hydrochloride | 75135 |
| Tramadol hydrochloride | 63898 |
| Tramadol hydrochloride | 77485 |
| Tramadol hydrochloride | 39709 |
| Tramadol hydrochloride | 40254 |
| Tramadol hydrochloride | 38956 |
| Tramadol hydrochloride | 42798 |
| Tramadol hydrochloride | 21397 |
| Tramadol hydrochloride | 39505 |
| Tramadol hydrochloride | 77034 |
| Tramadol hydrochloride | 61610 |
| Tramadol hydrochloride | 80005 |
| Tramadol hydrochloride | 35656 |
| Tramadol hydrochloride | 38528 |
| Tramadol hydrochloride | 75930 |
| Tramadol hydrochloride | 46587 |
| Tramadol hydrochloride | 28728 |
| Tramadol hydrochloride | 36732 |
| Tramadol hydrochloride | 36035 |
| Tramadol hydrochloride | 73076 |
| Tramadol hydrochloride | 78059 |
| Tramadol hydrochloride | 74839 |
| Tramadol hydrochloride | 68427 |
| Tramadol hydrochloride | 75958 |
| Tramadol hydrochloride | 9396 |
| Tramadol hydrochloride | 11559 |
| Tramadol hydrochloride | 52495 |
| Tramadol hydrochloride | 71355 |
| Tramadol hydrochloride | 60121 |
| Tramadol hydrochloride | 73596 |
| Tramadol hydrochloride | 67310 |
| Tramadol hydrochloride | 47854 |
| Tramadol hydrochloride | 73924 |
| Tramadol hydrochloride | 36697 |
| Tramadol hydrochloride | 39811 |
| Tramadol hydrochloride | 34639 |
| Tramadol hydrochloride | 65266 |
| Tramadol hydrochloride | 40926 |
| Tramadol hydrochloride | 34521 |
| Tramadol hydrochloride | 34281 |
| Tramadol hydrochloride | 34808 |
| Tramadol hydrochloride | 34570 |
| Tramadol hydrochloride | 26986 |
| Tramadol hydrochloride | 40883 |
| Tramadol hydrochloride | 79001 |
| Tramadol hydrochloride | 4834 |
| Tramadol hydrochloride | 27591 |
| Tramadol hydrochloride | 64731 |
| Tramadol hydrochloride | 40061 |
| Tramadol hydrochloride | 187 |
| Tramadol hydrochloride | 31105 |
| Tramadol hydrochloride | 63047 |
| Tramadol hydrochloride | 6215 |
| Tramadol hydrochloride | 67161 |
| Tramadol hydrochloride | 19993 |
| Tramadol hydrochloride | 24383 |
| Tramadol hydrochloride | 49324 |
| Tramadol hydrochloride | 20310 |
| Tramadol hydrochloride | 75661 |
| Tramadol hydrochloride | 50947 |
| Tramadol hydrochloride | 40166 |
| Tramadol hydrochloride | 35438 |
| Tramadol hydrochloride | 6153 |
| Tramadol hydrochloride | 36949 |
| Tramadol hydrochloride | 35651 |
| Tramadol hydrochloride | 73306 |
| Tramadol hydrochloride | 11549 |
| Tramadol hydrochloride | 74498 |
| Tramadol hydrochloride | 80049 |
| Tramadol hydrochloride | 75304 |
| Tramadol hydrochloride | 34065 |
| Tramadol hydrochloride | 40249 |
| Tramadol hydrochloride | 6558 |
| co-codaprin | 23496 |
| co-codaprin | 2986 |
| co-codaprin | 46925 |
| co-codaprin | 42218 |
| co-codaprin | 63658 |
| co-codaprin | 73634 |
| co-codaprin | 52856 |
| co-codaprin | 2047 |
| pregabalin | 71659 |
| pregabalin | 73455 |
| pregabalin | 79320 |
| pregabalin | 63174 |
| pregabalin | 10189 |
| pregabalin | 63964 |
| pregabalin | 819 |
| pregabalin | 6936 |
| pregabalin | 64497 |
| pregabalin | 64041 |
| pregabalin | 6949 |
| pregabalin | 16509 |
| pregabalin | 69498 |
| pregabalin | 64038 |
| pregabalin | 64039 |
| pregabalin | 63877 |
| pregabalin | 68014 |
| pregabalin | 70648 |
| pregabalin | 66509 |
| pregabalin | 65069 |
| pregabalin | 69877 |
| pregabalin | 69554 |
| pregabalin | 69987 |
| pregabalin | 63069 |
| pregabalin | 7209 |
| pregabalin | 69799 |
| pregabalin | 65073 |
| pregabalin | 71533 |
| pregabalin | 7394 |
| pregabalin | 64005 |
| pregabalin | 69034 |
| pregabalin | 66941 |
| pregabalin | 63090 |
| pregabalin | 69499 |
| pregabalin | 78146 |
| pregabalin | 69781 |
| pregabalin | 73026 |
| pregabalin | 73387 |
| pregabalin | 70735 |
| pregabalin | 70730 |
| pregabalin | 72068 |
| pregabalin | 69497 |
| pregabalin | 68441 |
| pregabalin | 51924 |
| pregabalin | 70546 |
| pregabalin | 48253 |
| pregabalin | 67053 |
| pregabalin | 65218 |
| pregabalin | 55972 |
| pregabalin | 6999 |
| pregabalin | 69501 |
| pregabalin | 70731 |
| pregabalin | 63965 |
| pregabalin | 65606 |
| pregabalin | 67184 |
| pregabalin | 64568 |
| pregabalin | 70229 |
| pregabalin | 64042 |
| pregabalin | 52547 |
| pregabalin | 70478 |
| pregabalin | 70545 |
| pregabalin | 16542 |
| pregabalin | 63088 |
| pregabalin | 73817 |
| pregabalin | 790 |
| pregabalin | 63317 |
| pregabalin | 37801 |
| pregabalin | 38293 |
| pregabalin | 64040 |
| pregabalin | 60543 |
| pregabalin | 77847 |
| pregabalin | 7208 |
| pregabalin | 67384 |
| pregabalin | 71313 |
| pregabalin | 73424 |
| pregabalin | 77221 |
| pregabalin | 51227 |
| pregabalin | 64037 |
| pregabalin | 7005 |
| pregabalin | 69418 |
| pregabalin | 63091 |
| pregabalin | 69296 |
| pregabalin | 71461 |
| pregabalin | 63089 |
| pregabalin | 69125 |
| pregabalin | 70064 |
| pregabalin | 67440 |
| pregabalin | 76318 |
| pregabalin | 6631 |
| pregabalin | 6584 |
| pregabalin | 71221 |
| pregabalin | 64285 |
| pregabalin | 65863 |
| pregabalin | 63300 |
| pregabalin | 73584 |
| pregabalin | 70729 |
| pregabalin | 65787 |
| pregabalin | 70544 |

Table S3: Codes used for identifying analgesics prescribed in CPRD Aurum.

| Analgesics | Product code ID |
| --- | --- |
| Aceclofenac | 12041000033110 |
| Aceclofenac | 285541000033115 |
| Buprenorphine | 2737741000033115 |
| Buprenorphine | 3343341000033117 |
| Buprenorphine | 8882841000033114 |
| Buprenorphine | 2737641000033112 |
| Buprenorphine | 3343841000033114 |
| Buprenorphine | 8882741000033116 |
| Buprenorphine | 2737841000033113 |
| Buprenorphine | 2737541000033111 |
| Buprenorphine | 3343541000033112 |
| Buprenorphine | 167441000033112 |
| Buprenorphine | 2737941000033117 |
| Buprenorphine | 3343641000033113 |
| Buprenorphine | 3343441000033111 |
| Buprenorphine | 2738041000033119 |
| Buprenorphine | 3343741000033116 |
| Buprenorphine | 8882941000033118 |
| Capsaicin | 1766441000033119 |
| Carbamazepine | 1415041000033114 |
| Carbamazepine | 1428941000033110 |
| Carbamazepine | 178841000033117 |
| Carbamazepine | 2929441000033113 |
| Carbamazepine | 2266141000033116 |
| Carbamazepine | 2928641000033118 |
| Carbamazepine | 214841000033119 |
| Carbamazepine | 2668141000033117 |
| Carbamazepine | 1425941000033115 |
| Carbamazepine | 2730541000033110 |
| Carbamazepine | 212641000033113 |
| Carbamazepine | 2928741000033110 |
| Carbamazepine | 1428841000033119 |
| Carbamazepine | 178741000033110 |
| Carbamazepine | 208541000033112 |
| Carbamazepine | 9120041000033118 |
| Carbamazepine | 214741000033112 |
| Carbamazepine | 214941000033110 |
| Carbamazepine | 212541000033112 |
| Carbamazepine | 9119841000033112 |
| Carbamazepine | 1426041000033113 |
| Carbamazepine | 2668041000033116 |
| Carbamazepine | 218241000033113 |
| Carbamazepine | 3979441000033115 |
| Carbamazepine | 1729541000033118 |
| Carbamazepine | 218341000033115 |
| Carbamazepine | 2730441000033114 |
| Carbamazepine | 1425841000033111 |
| Carbamazepine | 2929341000033119 |
| Carbamazepine | 198141000033115 |
| Carbamazepine | 2266041000033115 |
| Carbamazepine | 1424241000033113 |
| Carbamazepine | 9119941000033116 |
| Carbamazepine | 1729741000033114 |
| Carbamazepine | 1415141000033113 |
| Carbamazepine | 208141000033115 |
| Carbamazepine | 212741000033116 |
| Carbamazepine | 1729641000033117 |
| Celecoxib | 2103241000033116 |
| Celecoxib | 2103341000033114 |
| Celecoxib | 5707541000033118 |
| Celecoxib | 2103041000033112 |
| Celecoxib | 5707441000033119 |
| Celecoxib | 2103141000033111 |
| Celecoxib | 1136241000033118 |
| Co-codamol | 2875341000033110 |
| Co-codamol | 372941000033118 |
| Co-codamol | 318041000033114 |
| Co-codamol | 6386441000033115 |
| Co-codamol | 370641000033114 |
| Co-codamol | 6431341000033116 |
| Co-codamol | 326141000033118 |
| Co-codamol | 373041000033111 |
| Co-codamol | 1588741000033112 |
| Co-codamol | 295441000033117 |
| Co-codamol | 5334541000033117 |
| Co-codamol | 294841000033118 |
| Co-codamol | 295341000033111 |
| Co-codamol | 4590641000033113 |
| Co-dydramol | 5891241000033112 |
| Co-dydramol | 3851441000033111 |
| Co-dydramol | 373141000033110 |
| Co-dydramol | 3180141000033114 |
| Co-dydramol | 3180241000033119 |
| Codeine phosphate | 140841000033118 |
| Codeine phosphate | 2228341000033113 |
| Codeine phosphate | 1403141000033111 |
| Codeine phosphate | 443241000033114 |
| Codeine phosphate | 1029741000033111 |
| Codeine phosphate | 3331541000033110 |
| Codeine phosphate | 831641000033117 |
| Codeine phosphate | 2746141000033115 |
| Codeine phosphate | 796641000033119 |
| Codeine phosphate | 922541000033116 |
| Codeine phosphate | 1512141000033114 |
| Codeine phosphate | 1621841000033112 |
| Codeine phosphate | 795741000033118 |
| Codeine phosphate | 2968641000033114 |
| Codeine phosphate | 6386541000033119 |
| Codeine phosphate | 2645041000033118 |
| Codeine phosphate | 336041000033115 |
| Codeine phosphate | 8246141000033119 |
| Codeine phosphate | 368541000033115 |
| Codeine phosphate | 3334541000033113 |
| Codeine phosphate | 624441000033112 |
| Codeine phosphate | 916741000033116 |
| Codeine phosphate | 3077941000033110 |
| Codeine phosphate | 3934441000033118 |
| Codeine phosphate | 335841000033117 |
| Codeine phosphate | 1140541000033115 |
| Codeine phosphate | 2968841000033110 |
| Codeine phosphate | 1040041000033117 |
| Codeine phosphate | 443141000033119 |
| Codeine phosphate | 368441000033116 |
| Codeine phosphate | 3057541000033118 |
| Codeine phosphate | 1741241000033116 |
| Codeine phosphate | 3229141000033111 |
| Codeine phosphate | 335641000033118 |
| Codeine phosphate | 371341000033114 |
| Codeine phosphate | 1830841000033114 |
| Codeine phosphate | 625141000033115 |
| Codeine phosphate | 1030141000033119 |
| Codeine phosphate | 371241000033116 |
| Codeine phosphate | 1479641000033111 |
| Codeine phosphate | 6431441000033110 |
| Codeine phosphate | 2745941000033112 |
| Codeine phosphate | 86941000033114 |
| Codeine phosphate | 1363841000033114 |
| Codeine phosphate | 2850041000033116 |
| Codeine phosphate | 1479741000033119 |
| Codeine phosphate | 4432041000033115 |
| Codeine phosphate | 1698041000033118 |
| Codeine phosphate | 1352541000033110 |
| Codeine phosphate | 6137941000033114 |
| Codeine phosphate | 3934341000033112 |
| Codeine phosphate | 336741000033117 |
| Codeine phosphate | 371141000033111 |
| Dexibuprofen | 3334141000033116 |
| Dexibuprofen | 3334441000033112 |
| Dexibuprofen | 3334241000033111 |
| Dexibuprofen | 3334341000033118 |
| Dexketoprofen | 1795941000033115 |
| Dexketoprofen | 1795841000033111 |
| Diclofenac potassium | 1848741000033117 |
| Diclofenac potassium | 6430441000033118 |
| Diclofenac potassium | 1848841000033110 |
| Diclofenac potassium | 4657341000033118 |
| Diclofenac potassium | 1849041000033111 |
| Diclofenac potassium | 4657241000033111 |
| Diclofenac potassium | 1848941000033119 |
| Diclofenac sodium | 3839241000033110 |
| Diclofenac sodium | 1845941000033114 |
| Diclofenac sodium | 2104841000033115 |
| Diclofenac sodium | 2295141000033114 |
| Diclofenac sodium | 4936441000033114 |
| Diclofenac sodium | 1525541000033119 |
| Diclofenac sodium | 3217641000033116 |
| Diclofenac sodium | 80941000033112 |
| Diclofenac sodium | 441941000033116 |
| Diclofenac sodium | 453441000033113 |
| Diclofenac sodium | 1673241000033113 |
| Diclofenac sodium | 453341000033119 |
| Diclofenac sodium | 1610441000033114 |
| Diclofenac sodium | 467041000033116 |
| Diclofenac sodium | 2787841000033117 |
| Diclofenac sodium | 1527041000033118 |
| Diclofenac sodium | 451541000033113 |
| Diclofenac sodium | 2778941000033116 |
| Diclofenac sodium | 2779041000033113 |
| Diclofenac sodium | 2780441000033116 |
| Diclofenac sodium | 2787741000033110 |
| Diclofenac sodium | 852141000033115 |
| Diclofenac sodium | 80741000033114 |
| Diclofenac sodium | 1673141000033118 |
| Diclofenac sodium | 1346841000033114 |
| Diclofenac sodium | 463141000033110 |
| Diclofenac sodium | 462441000033111 |
| Diclofenac sodium | 588641000033115 |
| Diclofenac sodium | 1845841000033118 |
| Diclofenac sodium | 2104941000033111 |
| Diclofenac sodium | 8494341000033116 |
| Diclofenac sodium | 3078441000033116 |
| Diclofenac sodium | 4936341000033115 |
| Diclofenac sodium | 930441000033118 |
| Diclofenac sodium | 8494241000033114 |
| Diclofenac sodium | 2787541000033119 |
| Diclofenac sodium | 2780541000033115 |
| Diclofenac sodium | 1526341000033118 |
| Diclofenac sodium | 1527241000033114 |
| Diclofenac sodium | 2787641000033118 |
| Diclofenac sodium | 1730241000033112 |
| Diclofenac sodium | 3078341000033110 |
| Diclofenac sodium | 2980141000033116 |
| Diclofenac sodium | 442141000033114 |
| Diclofenac sodium | 452241000033117 |
| Diclofenac sodium | 2980241000033111 |
| Diclofenac sodium | 845341000033110 |
| Diclofenac sodium | 2716541000033115 |
| Diclofenac sodium | 1905341000033110 |
| Diclofenac sodium | 1727941000033118 |
| Diclofenac sodium | 1527541000033111 |
| Diclofenac sodium | 852241000033110 |
| Diclofenac sodium | 1527641000033112 |
| Diclofenac sodium | 2583941000033115 |
| Diclofenac sodium | 453541000033114 |
| Diclofenac sodium | 845241000033117 |
| Diclofenac sodium | 5809541000033110 |
| Diclofenac sodium | 2730241000033113 |
| Diclofenac sodium | 550341000033119 |
| Diclofenac sodium | 1905241000033117 |
| Diclofenac sodium | 467141000033117 |
| Diclofenac sodium | 1922741000033112 |
| Diclofenac sodium | 1922841000033119 |
| Diclofenac sodium | 8113541000033114 |
| Diclofenac sodium | 2274341000033116 |
| Diclofenac sodium | 1527141000033119 |
| Diclofenac sodium | 3836241000033118 |
| Diclofenac sodium | 6515641000033110 |
| Diclofenac sodium | 463241000033115 |
| Diclofenac sodium | 3217541000033117 |
| Diclofenac sodium | 2583841000033111 |
| Diclofenac sodium | 467241000033112 |
| Diclofenac sodium | 2730341000033115 |
| Diclofenac sodium | 2295441000033118 |
| Diclofenac sodium | 8113641000033110 |
| Diclofenac sodium | 462341000033117 |
| Dihydrocodeine tartrate | 1164541000033115 |
| Dihydrocodeine tartrate | 468641000033111 |
| Dihydrocodeine tartrate | 1157541000033117 |
| Dihydrocodeine tartrate | 7859141000033110 |
| Dihydrocodeine tartrate | 433541000033110 |
| Dihydrocodeine tartrate | 443541000033111 |
| Dihydrocodeine tartrate | 433441000033114 |
| Dihydrocodeine tartrate | 442841000033115 |
| Dihydrocodeine tartrate | 433141000033118 |
| Dihydrocodeine tartrate | 462841000033114 |
| Dihydrocodeine tartrate | 469441000033116 |
| Dihydrocodeine tartrate | 1043041000033113 |
| Dihydrocodeine tartrate | 433341000033115 |
| Dihydrocodeine tartrate | 468741000033119 |
| Dihydrocodeine tartrate | 469541000033115 |
| Dihydrocodeine tartrate | 7859041000033111 |
| Dihydrocodeine tartrate | 468541000033110 |
| Dipipanone | 3179641000033116 |
| Etodolac | 548541000033114 |
| Etodolac | 546941000033116 |
| Etodolac | 547241000033111 |
| Etodolac | 2946341000033111 |
| Etodolac | 2295941000033111 |
| Etodolac | 845041000033113 |
| Etodolac | 4010241000033113 |
| Etodolac | 2295841000033115 |
| Etoricoxib | 4425141000033112 |
| Etoricoxib | 2731141000033113 |
| Etoricoxib | 2731241000033118 |
| Etoricoxib | 2731041000033114 |
| Etoricoxib | 2731441000033117 |
| Etoricoxib | 2731341000033111 |
| Etoricoxib | 4425241000033117 |
| Etoricoxib | 2731541000033116 |
| Felbinac | 1458241000033119 |
| Felbinac | 5052441000033114 |
| Felbinac | 566641000033116 |
| Felbinac | 566841000033115 |
| Felbinac | 1458541000033117 |
| Fentanyl | 4956141000033119 |
| Fentanyl | 3839441000033111 |
| Fentanyl | 490341000033116 |
| Fentanyl | 575241000033117 |
| Fentanyl | 574941000033113 |
| Fentanyl | 4022741000033113 |
| Fentanyl | 6440741000033114 |
| Fentanyl | 490541000033111 |
| Fentanyl | 3248341000033116 |
| Fentanyl | 5007441000033116 |
| Fentanyl | 4022641000033116 |
| Fentanyl | 5007541000033115 |
| Fentanyl | 8962341000033111 |
| Fentanyl | 4426041000033116 |
| Fentanyl | 3839341000033117 |
| Fentanyl | 9204441000033114 |
| Fentanyl | 3333041000033118 |
| Fentanyl | 490241000033114 |
| Fentanyl | 3333141000033119 |
| Fentanyl | 567641000033118 |
| Fentanyl | 8884441000033117 |
| Fentanyl | 4426141000033117 |
| Fentanyl | 575041000033113 |
| Fentanyl | 8884541000033116 |
| Fentanyl | 4386941000033118 |
| Fentanyl | 9204541000033110 |
| Fentanyl | 8884641000033115 |
| Fentanyl | 6440841000033116 |
| Fentanyl | 4502941000033115 |
| Fentanyl | 3248041000033118 |
| Fentanyl | 5007241000033117 |
| Fentanyl | 4022841000033115 |
| Fentanyl | 6441041000033119 |
| Fentanyl | 6441141000033115 |
| Fentanyl | 5300441000033116 |
| Fentanyl | 4425741000033111 |
| Fentanyl | 4426241000033112 |
| Fentanyl | 4022941000033111 |
| Fentanyl | 4386641000033113 |
| Fentanyl | 4503141000033112 |
| Fentanyl | 3332841000033115 |
| Fentanyl | 8884341000033111 |
| Fentanyl | 3248141000033119 |
| Fentanyl | 4386741000033116 |
| Fentanyl | 4426341000033119 |
| Fentanyl | 575141000033112 |
| Fentanyl | 4503041000033113 |
| Fentanyl | 4956241000033114 |
| Fentanyl | 3248241000033114 |
| Fentanyl | 4387041000033117 |
| Fentanyl | 4956041000033118 |
| Fentanyl | 4386841000033114 |
| Fentanyl | 5007341000033110 |
| Fentanyl | 490441000033110 |
| Fentanyl | 567741000033110 |
| Fentanyl | 3332941000033111 |
| Fentanyl | 4956341000033116 |
| Fentanyl | 4502841000033111 |
| Fentanyl | 8884741000033112 |
| Fentanyl | 4023041000033118 |
| Fentanyl | 6440941000033112 |
| Fentanyl | 8962241000033118 |
| Flurbiprofen | 614441000033119 |
| Flurbiprofen | 594041000033116 |
| Flurbiprofen | 577741000033116 |
| Flurbiprofen | 614541000033118 |
| Flurbiprofen | 609441000033115 |
| Flurbiprofen | 593941000033118 |
| Gabapentin | 2796341000033111 |
| Gabapentin | 620641000033115 |
| Gabapentin | 958241000033116 |
| Gabapentin | 5968241000033114 |
| Gabapentin | 5968141000033119 |
| Gabapentin | 2146241000033115 |
| Gabapentin | 620841000033119 |
| Gabapentin | 5993441000033118 |
| Gabapentin | 8237041000033117 |
| Gabapentin | 958041000033112 |
| Gabapentin | 2145941000033118 |
| Gabapentin | 2796241000033118 |
| Gabapentin | 5100341000033117 |
| Gabapentin | 5968641000033112 |
| Gabapentin | 5968341000033116 |
| Gabapentin | 8125341000033112 |
| Gabapentin | 2146141000033110 |
| Gabapentin | 5968441000033110 |
| Gabapentin | 958141000033111 |
| Gabapentin | 2146041000033111 |
| Gabapentin | 5890841000033118 |
| Gabapentin | 5968541000033111 |
| Gabapentin | 620741000033112 |
| Hydromorphone hydrochloride | 738641000033112 |
| Hydromorphone hydrochloride | 738341000033116 |
| Hydromorphone hydrochloride | 738541000033111 |
| Hydromorphone hydrochloride | 1029241000033117 |
| Hydromorphone hydrochloride | 738441000033110 |
| Hydromorphone hydrochloride | 1037041000033119 |
| Hydromorphone hydrochloride | 728641000033114 |
| Hydromorphone hydrochloride | 1029141000033112 |
| Hydromorphone hydrochloride | 1036741000033118 |
| Hydromorphone hydrochloride | 1036941000033115 |
| Hydromorphone hydrochloride | 728741000033117 |
| Hydromorphone hydrochloride | 738241000033114 |
| Hydromorphone hydrochloride | 1036841000033111 |
| Hydromorphone hydrochloride | 1036641000033110 |
| Ibuprofen | 5376741000033119 |
| Ibuprofen | 4123241000033119 |
| Ibuprofen | 419641000033118 |
| Ibuprofen | 747341000033119 |
| Ibuprofen | 4432641000033114 |
| Ibuprofen | 2970641000033119 |
| Ibuprofen | 746841000033113 |
| Ibuprofen | 2970741000033111 |
| Ibuprofen | 2616341000033119 |
| Ibuprofen | 1739441000033117 |
| Ibuprofen | 157841000033115 |
| Ibuprofen | 746441000033110 |
| Ibuprofen | 1739241000033118 |
| Ibuprofen | 746941000033117 |
| Ibuprofen | 2971241000033112 |
| Ibuprofen | 2839441000033116 |
| Ibuprofen | 4123741000033113 |
| Ibuprofen | 747141000033117 |
| Ibuprofen | 4803341000033111 |
| Ibuprofen | 1780141000033111 |
| Ibuprofen | 81641000033113 |
| Ibuprofen | 81741000033116 |
| Ibuprofen | 747241000033112 |
| Ibuprofen | 3248541000033111 |
| Ibuprofen | 4258141000033119 |
| Ibuprofen | 4451841000033119 |
| Ibuprofen | 2968541000033113 |
| Ibuprofen | 939741000033119 |
| Ibuprofen | 4661241000033110 |
| Ibuprofen | 1113041000033114 |
| Ibuprofen | 162941000033117 |
| Ibuprofen | 2968941000033119 |
| Ibuprofen | 4456341000033118 |
| Ibuprofen | 746341000033116 |
| Ibuprofen | 161341000033113 |
| Ibuprofen | 2970541000033115 |
| Ibuprofen | 2839541000033115 |
| Ibuprofen | 163041000033110 |
| Ibuprofen | 746641000033112 |
| Ibuprofen | 159441000033110 |
| Ibuprofen | 747041000033116 |
| Ibuprofen | 566941000033111 |
| Ibuprofen | 2273241000033112 |
| Ibuprofen | 4158541000033113 |
| Ibuprofen | 4456441000033112 |
| Ibuprofen | 2110741000033116 |
| Ibuprofen | 747441000033113 |
| Ibuprofen | 81841000033114 |
| Ibuprofen | 2765841000033110 |
| Ibuprofen | 162841000033113 |
| Ibuprofen | 746741000033115 |
| Ibuprofen | 2871341000033112 |
| Ibuprofen | 5376641000033111 |
| Ibuprofen | 4088341000033115 |
| Ibuprofen | 2797741000033114 |
| Ibuprofen | 939841000033112 |
| Ibuprofen | 747541000033114 |
| Ibuprofen | 3201741000033119 |
| Ibuprofen | 1780241000033116 |
| Ibuprofen | 4088541000033110 |
| Ibuprofen | 939941000033116 |
| Ibuprofen | 1739341000033111 |
| Ibuprofen | 6168541000033111 |
| Ibuprofen | 564041000033119 |
| Ibuprofen | 2974241000033110 |
| Ibuprofen | 4258241000033114 |
| Ibuprofen | 2131141000033117 |
| Ibuprofen | 1627241000033117 |
| Ibuprofen | 2765941000033119 |
| Ibuprofen | 4803141000033113 |
| Ibuprofen | 746541000033111 |
| Ibuprofen | 4088441000033114 |
| Indometacin | 3093441000033117 |
| Indometacin | 3093041000033114 |
| Indometacin | 3093641000033115 |
| Indometacin | 757441000033110 |
| Indometacin | 596241000033116 |
| Indometacin | 3093741000033112 |
| Indometacin | 757241000033114 |
| Indometacin | 4452141000033117 |
| Indometacin | 2128341000033111 |
| Indometacin | 756841000033110 |
| Indometacin | 757041000033118 |
| Indometacin | 767741000033111 |
| Indometacin | 756941000033119 |
| Indometacin | 4447641000033110 |
| Indometacin | 3093341000033111 |
| Indometacin | 757341000033116 |
| Indometacin | 596341000033114 |
| Indometacin | 3093541000033116 |
| Indometacin | 3093141000033113 |
| Indometacin | 1346341000033117 |
| Indometacin | 1168141000033117 |
| Indometacin | 5996241000033113 |
| Indometacin | 767941000033114 |
| Indometacin | 2799441000033111 |
| Indometacin | 595941000033119 |
| Ketoprofen | 1010241000033116 |
| Ketoprofen | 3929041000033111 |
| Ketoprofen | 3136641000033111 |
| Ketoprofen | 797941000033111 |
| Ketoprofen | 5324141000033111 |
| Ketoprofen | 797341000033112 |
| Ketoprofen | 797241000033119 |
| Ketoprofen | 798841000033119 |
| Ketoprofen | 801541000033112 |
| Ketoprofen | 6018341000033114 |
| Ketoprofen | 1013841000033111 |
| Ketoprofen | 797841000033115 |
| Ketoprofen | 797441000033118 |
| Ketoprofen | 801841000033114 |
| Ketoprofen | 5324241000033116 |
| Ketoprofen | 801741000033116 |
| Ketoprofen | 1010141000033111 |
| Ketoprofen | 5324341000033114 |
| Ketoprofen | 801641000033113 |
| Ketoprofen | 2781041000033116 |
| Ketoprofen | 2128241000033118 |
| Ketoprofen | 2928141000033111 |
| Ketoprofen | 1012141000033112 |
| Ketoprofen | 2781141000033117 |
| Ketoprofen | 801941000033118 |
| Ketoprofen | 5324041000033112 |
| Ketoprofen | 1013741000033118 |
| Ketoprofen | 1102841000033114 |
| Ketoprofen | 2104441000033118 |
| Ketoprofen | 1010341000033114 |
| Ketoprofen | 567941000033113 |
| Mefenamic acid | 872641000033110 |
| Mefenamic acid | 874641000033117 |
| Mefenamic acid | 491941000033111 |
| Mefenamic acid | 2661841000033116 |
| Mefenamic acid | 895741000033116 |
| Mefenamic acid | 889141000033118 |
| Mefenamic acid | 1109541000033115 |
| Mefenamic acid | 491841000033115 |
| Mefenamic acid | 1096241000033119 |
| Meloxicam | 938341000033110 |
| Meloxicam | 899241000033112 |
| Meloxicam | 938441000033116 |
| Meloxicam | 8617841000033117 |
| Meloxicam | 899341000033119 |
| Meloxicam | 8617741000033110 |
| Meptazinol | 896341000033113 |
| Meptazinol | 896441000033119 |
| Morphine | 944041000033117 |
| Morphine | 941341000033117 |
| Morphine | 936541000033119 |
| Morphine | 2099641000033110 |
| Morphine | 1924041000033119 |
| Morphine | 1744741000033112 |
| Morphine | 928741000033114 |
| Morphine | 1014541000033111 |
| Morphine | 1561541000033114 |
| Morphine | 930341000033112 |
| Morphine | 933241000033110 |
| Morphine | 940341000033113 |
| Morphine | 3032841000033119 |
| Morphine | 1923941000033116 |
| Morphine | 1561441000033113 |
| Morphine | 2068841000033115 |
| Morphine | 1561241000033112 |
| Morphine | 1014941000033117 |
| Morphine | 2068441000033118 |
| Morphine | 4434241000033119 |
| Morphine | 931841000033113 |
| Morphine | 2912141000033117 |
| Morphine | 795641000033110 |
| Morphine | 941041000033119 |
| Morphine | 930041000033110 |
| Morphine | 929241000033111 |
| Morphine | 1014441000033110 |
| Morphine | 1278541000033116 |
| Morphine | 2912441000033113 |
| Morphine | 930241000033119 |
| Morphine | 2753441000033117 |
| Morphine | 1924241000033110 |
| Morphine | 2912241000033112 |
| Morphine | 2068541000033117 |
| Morphine | 2753541000033116 |
| Morphine | 944341000033115 |
| Morphine | 931741000033115 |
| Morphine | 2753641000033115 |
| Morphine | 5966741000033116 |
| Morphine | 2180541000033110 |
| Morphine | 4817241000033113 |
| Morphine | 7859941000033112 |
| Morphine | 940041000033111 |
| Morphine | 927941000033113 |
| Morphine | 944241000033113 |
| Morphine | 933141000033115 |
| Morphine | 924841000033114 |
| Morphine | 929741000033117 |
| Morphine | 2180341000033115 |
| Morphine | 936641000033118 |
| Morphine | 936441000033115 |
| Morphine | 934341000033119 |
| Morphine | 928041000033111 |
| Morphine | 930741000033113 |
| Morphine | 930941000033111 |
| Morphine | 466241000033114 |
| Morphine | 944441000033114 |
| Morphine | 940441000033119 |
| Morphine | 1752541000033114 |
| Morphine | 939541000033110 |
| Morphine | 451141000033116 |
| Morphine | 940741000033114 |
| Morphine | 2753341000033111 |
| Morphine | 1276641000033119 |
| Morphine | 1561141000033117 |
| Morphine | 447541000033115 |
| Morphine | 931541000033111 |
| Morphine | 1561341000033119 |
| Morphine | 1278641000033115 |
| Morphine | 933341000033117 |
| Morphine | 3032941000033110 |
| Morphine | 928641000033117 |
| Morphine | 4556341000033112 |
| Morphine | 931641000033112 |
| Morphine | 938241000033117 |
| Morphine | 930141000033114 |
| Morphine | 451341000033118 |
| Morphine | 940141000033110 |
| Morphine | 2912341000033119 |
| Morphine | 941141000033115 |
| Morphine | 1924141000033115 |
| Morphine | 1752041000033116 |
| Morphine | 1014841000033113 |
| Morphine | 931941000033117 |
| Morphine | 936741000033110 |
| Morphine | 1924341000033117 |
| Morphine | 944141000033118 |
| Morphine | 931041000033118 |
| Morphine | 929941000033119 |
| Morphine | 447741000033111 |
| Morphine | 911641000033119 |
| Morphine | 944541000033110 |
| Morphine | 2180641000033111 |
| Morphine | 2068641000033116 |
| Morphine | 2180441000033114 |
| Morphine | 932041000033111 |
| Morphine | 940941000033112 |
| Morphine | 930841000033115 |
| Morphine | 2068741000033113 |
| Morphine | 2927041000033113 |
| Morphine | 928841000033116 |
| Morphine | 466141000033119 |
| Morphine | 940841000033116 |
| Morphine | 940241000033115 |
| Morphine | 928941000033112 |
| Morphine | 1014641000033112 |
| Morphine | 941241000033110 |
| Morphine | 447641000033119 |
| Nabumetone | 956241000033115 |
| Nabumetone | 2850541000033114 |
| Nabumetone | 1164641000033119 |
| Nabumetone | 954341000033118 |
| Nabumetone | 2850641000033110 |
| Naproxen | 950141000033113 |
| Naproxen | 2637841000033116 |
| Naproxen | 994741000033112 |
| Naproxen | 955041000033119 |
| Naproxen | 949841000033113 |
| Naproxen | 4452241000033112 |
| Naproxen | 956441000033119 |
| Naproxen | 994841000033119 |
| Naproxen | 953041000033118 |
| Naproxen | 954841000033110 |
| Naproxen | 952941000033111 |
| Naproxen | 950341000033111 |
| Naproxen | 82041000033112 |
| Naproxen | 954941000033119 |
| Naproxen | 954241000033111 |
| Naproxen | 956941000033112 |
| Naproxen | 950041000033114 |
| Naproxen | 1403341000033114 |
| Naproxen | 954141000033116 |
| Naproxen | 6037341000033119 |
| Naproxen | 949641000033112 |
| Naproxen | 952841000033115 |
| Naproxen | 81941000033118 |
| Naproxen | 5973641000033114 |
| Naproxen | 6037241000033112 |
| Naproxen | 949941000033117 |
| Naproxen | 2637941000033112 |
| Naproxen | 955141000033115 |
| Naproxen | 949741000033115 |
| Nefopam hydrochloride | 966741000033117 |
| Nefopam hydrochloride | 12241000033119 |
| Oxycodone hydrochloride | 9061541000033116 |
| Oxycodone hydrochloride | 1982141000033119 |
| Oxycodone hydrochloride | 7886641000033116 |
| Oxycodone hydrochloride | 9176841000033112 |
| Oxycodone hydrochloride | 9177141000033116 |
| Oxycodone hydrochloride | 1987341000033118 |
| Oxycodone hydrochloride | 1988441000033113 |
| Oxycodone hydrochloride | 6125441000033114 |
| Oxycodone hydrochloride | 9061741000033112 |
| Oxycodone hydrochloride | 1988041000033116 |
| Oxycodone hydrochloride | 1988241000033112 |
| Oxycodone hydrochloride | 8048141000033110 |
| Oxycodone hydrochloride | 2748441000033110 |
| Oxycodone hydrochloride | 9809641000033116 |
| Oxycodone hydrochloride | 1987941000033119 |
| Oxycodone hydrochloride | 8537341000033111 |
| Oxycodone hydrochloride | 7886341000033112 |
| Oxycodone hydrochloride | 1988141000033117 |
| Oxycodone hydrochloride | 4898241000033113 |
| Oxycodone hydrochloride | 5234241000033110 |
| Oxycodone hydrochloride | 8838841000033119 |
| Oxycodone hydrochloride | 8838541000033116 |
| Oxycodone hydrochloride | 8838741000033112 |
| Oxycodone hydrochloride | 6125541000033110 |
| Oxycodone hydrochloride | 1982041000033118 |
| Oxycodone hydrochloride | 9177341000033118 |
| Oxycodone hydrochloride | 1987541000033113 |
| Oxycodone hydrochloride | 1025241000033119 |
| Oxycodone hydrochloride | 1987441000033112 |
| Oxycodone hydrochloride | 8537441000033117 |
| Oxycodone hydrochloride | 7886541000033117 |
| Oxycodone hydrochloride | 8048441000033119 |
| Oxycodone hydrochloride | 1987741000033117 |
| Oxycodone hydrochloride | 5234341000033117 |
| Oxycodone hydrochloride | 4898141000033118 |
| Oxycodone hydrochloride | 1987241000033111 |
| Oxycodone hydrochloride | 8048041000033111 |
| Oxycodone hydrochloride | 1987841000033110 |
| Oxycodone hydrochloride | 1982541000033111 |
| Oxycodone hydrochloride | 6125641000033111 |
| Oxycodone hydrochloride | 1988341000033119 |
| Oxycodone hydrochloride | 6125241000033113 |
| Oxycodone hydrochloride | 1987641000033114 |
| Oxycodone hydrochloride | 7886241000033119 |
| Oxycodone hydrochloride | 6125741000033119 |
| Oxycodone hydrochloride | 8048341000033113 |
| Oxycodone hydrochloride | 1981941000033112 |
| Oxycodone hydrochloride | 1987141000033116 |
| Oxycodone hydrochloride | 6125141000033118 |
| Oxycodone hydrochloride | 8838641000033115 |
| Oxycodone hydrochloride | 6125041000033117 |
| Oxycodone hydrochloride | 8048241000033115 |
| Oxycodone hydrochloride | 2748541000033111 |
| Oxycodone hydrochloride | 6125341000033115 |
| Oxycodone hydrochloride | 8537541000033116 |
| Oxycodone hydrochloride | 7886441000033118 |
| Papaveretum | 1041941000033110 |
| Papaveretum | 86841000033118 |
| Papaveretum | 1034241000033116 |
| Papaveretum | 1038141000033116 |
| Papaveretum | 3849841000033110 |
| Paracetamol | 5415041000033113 |
| Paracetamol | 1039841000033116 |
| Paracetamol | 2711441000033111 |
| Paracetamol | 2828441000033111 |
| Paracetamol | 2968741000033117 |
| Paracetamol | 456241000033118 |
| Paracetamol | 8534841000033111 |
| Paracetamol | 1029941000033114 |
| Paracetamol | 3179841000033115 |
| Paracetamol | 6386741000033110 |
| Paracetamol | 3859241000033114 |
| Paracetamol | 1040341000033115 |
| Paracetamol | 1038941000033119 |
| Paracetamol | 456541000033116 |
| Paracetamol | 388641000033117 |
| Paracetamol | 1040941000033116 |
| Paracetamol | 4417841000033119 |
| Paracetamol | 3924441000033115 |
| Paracetamol | 2828141000033115 |
| Paracetamol | 1811641000033110 |
| Paracetamol | 2215141000033110 |
| Paracetamol | 2972441000033117 |
| Paracetamol | 5100741000033116 |
| Paracetamol | 1038041000033115 |
| Paracetamol | 8199741000033117 |
| Paracetamol | 468141000033118 |
| Paracetamol | 1846641000033110 |
| Paracetamol | 5890041000033113 |
| Paracetamol | 2269341000033112 |
| Paracetamol | 1038541000033113 |
| Paracetamol | 6466941000033119 |
| Paracetamol | 758841000033114 |
| Paracetamol | 3179741000033113 |
| Paracetamol | 2190441000033110 |
| Paracetamol | 1030341000033116 |
| Paracetamol | 2920041000033114 |
| Paracetamol | 1039141000033110 |
| Paracetamol | 1040541000033110 |
| Paracetamol | 2827641000033112 |
| Paracetamol | 2850241000033112 |
| Paracetamol | 2871941000033111 |
| Paracetamol | 2827741000033115 |
| Paracetamol | 1040441000033114 |
| Paracetamol | 4500141000033118 |
| Paracetamol | 6386841000033117 |
| Paracetamol | 480141000033118 |
| Paracetamol | 906441000033113 |
| Paracetamol | 1037941000033118 |
| Paracetamol | 207441000033110 |
| Paracetamol | 6001941000033119 |
| Paracetamol | 2828541000033112 |
| Paracetamol | 4576141000033112 |
| Paracetamol | 1038641000033114 |
| Paracetamol | 2971341000033119 |
| Paracetamol | 8944241000033117 |
| Paracetamol | 4508141000033115 |
| Paracetamol | 1037541000033112 |
| Paracetamol | 2827841000033113 |
| Paracetamol | 3180041000033110 |
| Paracetamol | 5576941000033119 |
| Paracetamol | 207841000033113 |
| Paracetamol | 373241000033115 |
| Paracetamol | 5896641000033116 |
| Paracetamol | 1038841000033110 |
| Paracetamol | 388541000033118 |
| Paracetamol | 1747841000033111 |
| Paracetamol | 8116141000033113 |
| Paracetamol | 2215041000033111 |
| Pentazocine | 1068041000033114 |
| Pentazocine | 1053941000033111 |
| Pentazocine | 609341000033114 |
| Pentazocine | 1044641000033117 |
| Pethidine hydrochloride | 1052341000033116 |
| Pethidine hydrochloride | 1066841000033118 |
| Pethidine hydrochloride | 1065641000033110 |
| Pethidine hydrochloride | 1052441000033110 |
| Pethidine hydrochloride | 1053841000033115 |
| Piroxicam | 1086541000033118 |
| Piroxicam | 567041000033112 |
| Piroxicam | 1084541000033113 |
| Piroxicam | 1084641000033114 |
| Piroxicam | 1088641000033118 |
| Piroxicam | 566041000033110 |
| Piroxicam | 1089541000033114 |
| Piroxicam | 808741000033112 |
| Piroxicam | 563841000033112 |
| Piroxicam | 1579741000033114 |
| Piroxicam | 1083341000033116 |
| Piroxicam | 808641000033115 |
| Piroxicam | 1083441000033110 |
| Piroxicam | 565941000033117 |
| Piroxicam | 2617041000033119 |
| Piroxicam | 563941000033116 |
| Piroxicam | 571741000033113 |
| Piroxicam | 1666941000033115 |
| Piroxicam | 1083541000033111 |
| Piroxicam | 1083241000033114 |
| Sulindac | 286441000033113 |
| Sulindac | 1396141000033113 |
| Sulindac | 286341000033119 |
| Sulindac | 1396041000033114 |
| Tapentadol | 6132241000033119 |
| Tapentadol | 6133541000033111 |
| Tapentadol | 6133941000033117 |
| Tapentadol | 9160241000033118 |
| Tapentadol | 6132341000033112 |
| Tapentadol | 6133241000033114 |
| Tapentadol | 6133141000033119 |
| Tapentadol | 6134141000033116 |
| Tapentadol | 6134241000033111 |
| Tapentadol | 6133341000033116 |
| Tapentadol | 6133841000033113 |
| Tapentadol | 6134041000033115 |
| Tapentadol | 6133441000033110 |
| Tapentadol | 6132041000033110 |
| Tapentadol | 6132141000033114 |
| Targinact | 4898441000033114 |
| Targinact | 4898341000033115 |
| Targinact | 5234441000033111 |
| Targinact | 5234541000033112 |
| Tenoxicam | 939041000033117 |
| Tenoxicam | 1417941000033119 |
| Tenoxicam | 1416641000033111 |
| Tenoxicam | 1418741000033118 |
| Tenoxicam | 1425241000033112 |
| Tiaprofenic acid | 1443741000033115 |
| Tiaprofenic acid | 1443041000033118 |
| Tiaprofenic acid | 1396641000033115 |
| Tiaprofenic acid | 1398841000033115 |
| Tiaprofenic acid | 1444841000033110 |
| Tiaprofenic acid | 1442941000033111 |
| Tiaprofenic acid | 1389641000033114 |
| Tolfenamic acid | 1451941000033119 |
| Tolfenamic acid | 1445141000033115 |
| Tramacet | 3057641000033117 |
| Tramacet | 5596041000033111 |
| Tramadol | 6389141000033118 |
| Tramadol | 1549441000033115 |
| Tramadol | 2078441000033111 |
| Tramadol | 4824341000033117 |
| Tramadol | 4459341000033112 |
| Tramadol | 1549341000033114 |
| Tramadol | 3344441000033113 |
| Tramadol | 3996441000033116 |
| Tramadol | 4899241000033117 |
| Tramadol | 1462741000033116 |
| Tramadol | 1564741000033116 |
| Tramadol | 1462441000033111 |
| Tramadol | 4899141000033112 |
| Tramadol | 3909541000033113 |
| Tramadol | 1702041000033114 |
| Tramadol | 4824241000033110 |
| Tramadol | 1462641000033113 |
| Tramadol | 1462541000033112 |
| Tramadol | 4259141000033113 |
| Tramadol | 3180341000033112 |
| Tramadol | 3344141000033117 |
| Tramadol | 1549241000033116 |
| Tramadol | 1548041000033116 |
| Tramadol | 1702241000033118 |
| Tramadol | 2183541000033115 |
| Tramadol | 4424241000033116 |
| Tramadol | 1850441000033112 |
| Tramadol | 4028641000033118 |
| Tramadol | 4459541000033117 |
| Tramadol | 2183641000033119 |
| Tramadol | 4523141000033117 |
| Tramadol | 4523041000033116 |
| Tramadol | 1850141000033116 |
| Tramadol | 1564341000033117 |
| Tramadol | 1850341000033118 |
| Tramadol | 4523341000033119 |
| Tramadol | 1462941000033118 |
| Tramadol | 4899341000033110 |
| Tramadol | 4259241000033118 |
| Tramadol | 3996641000033119 |
| Tramadol | 4028341000033114 |
| Tramadol | 1564841000033114 |
| Tramadol | 1549541000033119 |
| Tramadol | 2980341000033118 |
| Tramadol | 1462341000033117 |
| Tramadol | 4899441000033116 |
| Tramadol | 2078541000033112 |
| Tramadol | 2183741000033111 |
| Tramadol | 4417641000033115 |
| Tramadol | 4417541000033116 |
| Tramadol | 2183441000033116 |
| Tramadol | 3996541000033115 |
| Tramadol | 1465741000033113 |
| Tramadol | 1564641000033113 |
| Tramadol | 1454241000033114 |
| Tramadol | 1849941000033112 |
| Tramadol | 1850241000033111 |
| Tramadol | 1701941000033115 |
| Tramadol | 4523241000033112 |
| Tramadol | 3909641000033114 |
| Tramadol | 1564541000033112 |
| Tramadol | 4459441000033118 |
| Tramadol | 3344341000033119 |
| Tramadol | 1702141000033113 |
| Tramadol | 1454541000033111 |
| Tramadol | 2980441000033112 |
| Tramadol | 1850041000033115 |
| Tramadol | 1462841000033114 |
| Tramadol | 4028541000033119 |
| Tramadol | 3344241000033112 |
| Tramadol | 2078241000033110 |
| Tramadol | 4824441000033111 |
| Tramadol | 5595941000033118 |
| Tramadol | 4417741000033112 |
| Tramadol | 3909741000033117 |
| Tramadol | 2078141000033115 |
| Tramadol | 2078341000033117 |
| co-codaprin | 370941000033119 |
| co-codaprin | 371041000033112 |
| pregabalin | 3154341000033111 |
| pregabalin | 3153641000033117 |
| pregabalin | 7871641000033118 |
| pregabalin | 3154141000033113 |
| pregabalin | 7871541000033119 |
| pregabalin | 3993541000033113 |
| pregabalin | 3153441000033119 |
| pregabalin | 5997941000033119 |
| pregabalin | 3154541000033116 |
| pregabalin | 3154041000033114 |
| pregabalin | 4387741000033119 |
| pregabalin | 4387641000033111 |
| pregabalin | 3154641000033115 |
| pregabalin | 3153541000033118 |
| pregabalin | 3153941000033112 |
| pregabalin | 3153341000033113 |
| pregabalin | 3154441000033117 |
| pregabalin | 3154241000033118 |
| pregabalin | 3153841000033116 |
| pregabalin | 4957341000033119 |
| pregabalin | 3153741000033114 |
