# Supplementary material for: Evaluation of common prescription analgesics and adjuvant analgesics as markers of suicide risk: a longitudinal population-based study in England
Source: Lancet Reg Health Eur. 2023 Jul 20;32:100695. doi: 10.1016/j.lanepe.2023.100695 (PMC10393825; doi:10.1016/j.lanepe.2023.100695)
Supplement: Supplementary Information 2 [file mmc2.docx]

## Supplementary Information 2

All psychiatric illnesses codes and terms used in our study are available in Table S1 for CPRD GOLD and Table S2 for CPRD Aurum. Codes for psychiatric diagnoses used in CPRD GOLD were primarily adopted from previous studies [1–3] before being updated, but those used in CPRD Aurum were newly developed. International Statistical Classification of Diseases and Related Health Problems 10^th^ version (ICD-10) codes and categories for psychiatric illnesses identified in the Hospital Episode Statistics (HES) are available in Table S3.

[1] Abel KM, Hope H, Swift E, Parisi R, Ashcroft DM, Kosidou K, Osam CS, Dalman C, Pierce M. Prevalence of maternal mental illness among children and adolescents in the UK between 2005 and 2017: a national retrospective cohort analysis. The Lancet Public Health 2019;4:e291–e300.

[2] Springate DA, Kontopantelis E, Ashcroft DM, Olier I, Parisi R, Chamapiwa E, Reeves D. ClinicalCodes: An Online Clinical Codes Repository to Improve the Validity and Reproducibility of Research Using Electronic Medical Records. PLoS ONE 2014;9:e99825.

[3] Windfuhr K, While D, Kapur N, Ashcroft DM, Kontopantelis E, Carr MJ, Shaw J, Appleby L, Webb RT. Suicide risk linked with clinical consultation frequency, psychiatric diagnoses and psychotropic medication prescribing in a national study of primary-care patients. Psychological Medicine 2016;46:3407–3417.

Table S1: Read terms and codes used for identifying psychiatric illnesses in CPRD GOLD.

| Read Code | Term | Category |
| --- | --- | --- |
| 1B16.00 | Agitated | affective disorders |
| E112.11 | Agitated depression | affective disorders |
| E135.00 | Agitated depression | affective disorders |
| E004300 | Arteriosclerotic dementia with depression | affective disorders |
| E11y200 | Atypical depressive disorder | affective disorders |
| E11y100 | Atypical manic disorder | affective disorders |
| E114300 | Bipolar affect disord, currently manic, severe, no psychosis | affective disorders |
| E114400 | Bipolar affect disord, currently manic,severe with psychosis | affective disorders |
| E115500 | Bipolar affect disord, now depressed, part/unspec remission | affective disorders |
| E115400 | Bipolar affect disord, now depressed, severe with psychosis | affective disorders |
| E115300 | Bipolar affect disord, now depressed, severe, no psychosis | affective disorders |
| E114500 | Bipolar affect disord,currently manic, part/unspec remission | affective disorders |
| 212V.00 | Bipolar affective disorder resolved | affective disorders |
| E115.00 | Bipolar affective disorder, currently depressed | affective disorders |
| E115z00 | Bipolar affective disorder, currently depressed, NOS | affective disorders |
| E115100 | Bipolar affective disorder, currently depressed, mild | affective disorders |
| E115200 | Bipolar affective disorder, currently depressed, moderate | affective disorders |
| E115000 | Bipolar affective disorder, currently depressed, unspecified | affective disorders |
| E114.00 | Bipolar affective disorder, currently manic | affective disorders |
| E114z00 | Bipolar affective disorder, currently manic, NOS | affective disorders |
| E114600 | Bipolar affective disorder, currently manic, full remission | affective disorders |
| E114100 | Bipolar affective disorder, currently manic, mild | affective disorders |
| E114200 | Bipolar affective disorder, currently manic, moderate | affective disorders |
| E114000 | Bipolar affective disorder, currently manic, unspecified | affective disorders |
| E115600 | Bipolar affective disorder, now depressed, in full remission | affective disorders |
| E11..11 | Bipolar psychoses | affective disorders |
| E290.00 | Brief depressive reaction | affective disorders |
| E290z00 | Brief depressive reaction NOS | affective disorders |
| 1B17.11 | C/O - feeling depressed | affective disorders |
| 1B17.12 | C/O - feeling unhappy | affective disorders |
| 1B1I111 | C/O weepiness | affective disorders |
| E2B1.00 | Chronic depression | affective disorders |
| 1B17.00 | Depressed | affective disorders |
| 1BT..00 | Depressed mood | affective disorders |
| 9H90.00 | Depression annual review | affective disorders |
| 9H92.00 | Depression interim review | affective disorders |
| 8BK0.00 | Depression management programme | affective disorders |
| 9H91.00 | Depression medication review | affective disorders |
| 212S.00 | Depression resolved | affective disorders |
| E2B..00 | Depressive disorder NEC | affective disorders |
| E11..12 | Depressive psychoses | affective disorders |
| 1B1U.11 | Depressive symptoms | affective disorders |
| 1BY..00 | Elevated mood | affective disorders |
| E112.14 | Endogenous depression | affective disorders |
| E113.11 | Endogenous depression - recurrent | affective disorders |
| E112.12 | Endogenous depression first episode | affective disorders |
| E112.13 | Endogenous depression first episode | affective disorders |
| 1465.00 | H/O: depression | affective disorders |
| 146D.00 | H/O: manic depressive disorder | affective disorders |
| E110.11 | Hypomanic psychoses | affective disorders |
| 6657.11 | Lithium monitoring | affective disorders |
| 1BQ..00 | Loss of capacity for enjoyment | affective disorders |
| 1BU..00 | Loss of hope for the future | affective disorders |
| 1BT..11 | Low mood | affective disorders |
| E110.00 | Manic disorder, single episode | affective disorders |
| E110z00 | Manic disorder, single episode NOS | affective disorders |
| 1S42.00 | Manic mood | affective disorders |
| E11..13 | Manic psychoses | affective disorders |
| E115.11 | Manic-depressive - now depressed | affective disorders |
| E114.11 | Manic-depressive - now manic | affective disorders |
| E11z200 | Masked depression | affective disorders |
| L184300 | Mental disorder during pregnancy - baby not yet delivered | affective disorders |
| L184400 | Mental disorder in puerperium - baby previously delivered | affective disorders |
| L184200 | Mental disorder in the puerperium - baby delivered | affective disorders |
| L184.00 | Mental disorders in pregnancy, childbirth and the puerperium | affective disorders |
| E116.00 | Mixed bipolar affective disorder | affective disorders |
| E116z00 | Mixed bipolar affective disorder, NOS | affective disorders |
| E116600 | Mixed bipolar affective disorder, in full remission | affective disorders |
| E116100 | Mixed bipolar affective disorder, mild | affective disorders |
| E116200 | Mixed bipolar affective disorder, moderate | affective disorders |
| E116500 | Mixed bipolar affective disorder, partial/unspec remission | affective disorders |
| E116400 | Mixed bipolar affective disorder, severe, with psychosis | affective disorders |
| E116300 | Mixed bipolar affective disorder, severe, without psychosis | affective disorders |
| E116000 | Mixed bipolar affective disorder, unspecified | affective disorders |
| 1BO..00 | Mood swings | affective disorders |
| E204.00 | Neurotic depression reactive type | affective disorders |
| 2257.00 | O/E - depressed | affective disorders |
| 225C.00 | O/E - elated | affective disorders |
| 9HA0.00 | On depression register | affective disorders |
| 9kQ..00 | On full dose long term treatment depression - enh serv admin | affective disorders |
| 6657.00 | On lithium | affective disorders |
| E11y.00 | Other and unspecified manic-depressive psychoses | affective disorders |
| E11yz00 | Other and unspecified manic-depressive psychoses NOS | affective disorders |
| E11y300 | Other mixed manic-depressive psychoses | affective disorders |
| E204.11 | Postnatal depression | affective disorders |
| E2B0.00 | Postviral depression | affective disorders |
| E001300 | Presenile dementia with depression | affective disorders |
| E291.00 | Prolonged depressive reaction | affective disorders |
| 212T.00 | Psychosis, schizophrenia + bipolar affective disord resolved | affective disorders |
| 62T1.00 | Puerperal depression | affective disorders |
| E130.00 | Reactive depressive psychosis | affective disorders |
| E11z100 | Rebound mood swings | affective disorders |
| E113700 | Recurrent depression | affective disorders |
| E113.00 | Recurrent major depressive episode | affective disorders |
| E113z00 | Recurrent major depressive episode NOS | affective disorders |
| E113600 | Recurrent major depressive episodes, in full remission | affective disorders |
| E113100 | Recurrent major depressive episodes, mild | affective disorders |
| E113200 | Recurrent major depressive episodes, moderate | affective disorders |
| E113300 | Recurrent major depressive episodes, severe, no psychosis | affective disorders |
| E113400 | Recurrent major depressive episodes, severe, with psychosis | affective disorders |
| E113000 | Recurrent major depressive episodes, unspecified | affective disorders |
| E113500 | Recurrent major depressive episodes,partial/unspec remission | affective disorders |
| E111z00 | Recurrent manic episode NOS | affective disorders |
| E111.00 | Recurrent manic episodes | affective disorders |
| E111600 | Recurrent manic episodes, in full remission | affective disorders |
| E111100 | Recurrent manic episodes, mild | affective disorders |
| E111200 | Recurrent manic episodes, moderate | affective disorders |
| E111500 | Recurrent manic episodes, partial or unspecified remission | affective disorders |
| E111300 | Recurrent manic episodes, severe without mention psychosis | affective disorders |
| E111400 | Recurrent manic episodes, severe, with psychosis | affective disorders |
| E111000 | Recurrent manic episodes, unspecified | affective disorders |
| 8HHq.00 | Referral for guided self-help for depression | affective disorders |
| 9HA1.00 | Removed from depression register | affective disorders |
| E118.00 | Seasonal affective disorder | affective disorders |
| E002100 | Senile dementia with depression | affective disorders |
| E112.00 | Single major depressive episode | affective disorders |
| E112z00 | Single major depressive episode NOS | affective disorders |
| E112600 | Single major depressive episode, in full remission | affective disorders |
| E112100 | Single major depressive episode, mild | affective disorders |
| E112200 | Single major depressive episode, moderate | affective disorders |
| E112500 | Single major depressive episode, partial or unspec remission | affective disorders |
| E112400 | Single major depressive episode, severe, with psychosis | affective disorders |
| E112300 | Single major depressive episode, severe, without psychosis | affective disorders |
| E112000 | Single major depressive episode, unspecified | affective disorders |
| E110600 | Single manic episode in full remission | affective disorders |
| E110500 | Single manic episode in partial or unspecified remission | affective disorders |
| E110100 | Single manic episode, mild | affective disorders |
| E110200 | Single manic episode, moderate | affective disorders |
| E110300 | Single manic episode, severe without mention of psychosis | affective disorders |
| E110400 | Single manic episode, severe, with psychosis | affective disorders |
| E110000 | Single manic episode, unspecified | affective disorders |
| 6657.12 | Started lithium | affective disorders |
| 1JJ..00 | Suspected depression | affective disorders |
| 1B1U.00 | Symptoms of depression | affective disorders |
| E117500 | Unspecified bipolar affect disord, partial/unspec remission | affective disorders |
| E117.00 | Unspecified bipolar affective disorder | affective disorders |
| E117z00 | Unspecified bipolar affective disorder, NOS | affective disorders |
| E117600 | Unspecified bipolar affective disorder, in full remission | affective disorders |
| E117100 | Unspecified bipolar affective disorder, mild | affective disorders |
| E117200 | Unspecified bipolar affective disorder, moderate | affective disorders |
| E117300 | Unspecified bipolar affective disorder, severe, no psychosis | affective disorders |
| E117000 | Unspecified bipolar affective disorder, unspecified | affective disorders |
| E117400 | Unspecified bipolar affective disorder,severe with psychosis | affective disorders |
| E11y000 | Unspecified manic-depressive psychoses | affective disorders |
| E03y300 | Unspecified puerperal psychosis | affective disorders |
| ZV11111 | [V]Personal history of manic-depressive psychosis | affective disorders |
| ZV11112 | [V]Personal history of manic-depressive psychosis | affective disorders |
| Eu32z14 | [X] Reactive depression NOS | affective disorders |
| Eu3z.11 | [X]Affective psychosis NOS | affective disorders |
| Eu32B00 | [X]Antenatal depression | affective disorders |
| Eu32y11 | [X]Atypical depression | affective disorders |
| Eu31400 | [X]Bipol aff disord, curr epis sev depress, no psychot symp | affective disorders |
| Eu31y11 | [X]Bipolar II disorder | affective disorders |
| Eu31911 | [X]Bipolar II disorder | affective disorders |
| Eu31500 | [X]Bipolar affect dis cur epi severe depres with psyc symp | affective disorders |
| Eu31200 | [X]Bipolar affect disorder cur epi manic with psychotic symp | affective disorders |
| Eu31100 | [X]Bipolar affect disorder cur epi manic wout psychotic symp | affective disorders |
| Eu31300 | [X]Bipolar affect disorder cur epi mild or moderate depressn | affective disorders |
| Eu31.00 | [X]Bipolar affective disorder | affective disorders |
| Eu31800 | [X]Bipolar affective disorder type I | affective disorders |
| Eu31900 | [X]Bipolar affective disorder type II | affective disorders |
| Eu31000 | [X]Bipolar affective disorder, current episode hypomanic | affective disorders |
| Eu31600 | [X]Bipolar affective disorder, current episode mixed | affective disorders |
| Eu31700 | [X]Bipolar affective disorder, currently in remission | affective disorders |
| Eu31z00 | [X]Bipolar affective disorder, unspecified | affective disorders |
| Eu30.11 | [X]Bipolar disorder, single manic episode | affective disorders |
| Eu34000 | [X]Cyclothymia | affective disorders |
| Eu32z11 | [X]Depression NOS | affective disorders |
| Eu92000 | [X]Depressive conduct disorder | affective disorders |
| Eu32z12 | [X]Depressive disorder NOS | affective disorders |
| Eu32.00 | [X]Depressive episode | affective disorders |
| Eu32z00 | [X]Depressive episode, unspecified | affective disorders |
| Eu34111 | [X]Depressive neurosis | affective disorders |
| Eu34100 | [X]Dysthymia | affective disorders |
| Eu33311 | [X]Endogenous depression with psychotic symptoms | affective disorders |
| Eu33211 | [X]Endogenous depression without psychotic symptoms | affective disorders |
| Eu30000 | [X]Hypomania | affective disorders |
| Eu32500 | [X]Major depression, mild | affective disorders |
| Eu32600 | [X]Major depression, moderately severe | affective disorders |
| Eu33212 | [X]Major depression, recurrent without psychotic symptoms | affective disorders |
| Eu32800 | [X]Major depression, severe with psychotic symptoms | affective disorders |
| Eu32700 | [X]Major depression, severe without psychotic symptoms | affective disorders |
| Eu30z11 | [X]Mania NOS | affective disorders |
| Eu30211 | [X]Mania with mood-congruent psychotic symptoms | affective disorders |
| Eu30212 | [X]Mania with mood-incongruent psychotic symptoms | affective disorders |
| Eu30200 | [X]Mania with psychotic symptoms | affective disorders |
| Eu30100 | [X]Mania without psychotic symptoms | affective disorders |
| Eu30.00 | [X]Manic episode | affective disorders |
| Eu30z00 | [X]Manic episode, unspecified | affective disorders |
| Eu33213 | [X]Manic-depress psychosis,depressd,no psychotic symptoms | affective disorders |
| Eu33312 | [X]Manic-depress psychosis,depressed type+psychotic symptoms | affective disorders |
| Eu31.11 | [X]Manic-depressive illness | affective disorders |
| Eu31.12 | [X]Manic-depressive psychosis | affective disorders |
| Eu31.13 | [X]Manic-depressive reaction | affective disorders |
| Eu53.00 | [X]Mental and behav disorders assoc with the puerperium NEC | affective disorders |
| Eu15500 | [X]Mental/behav dis oth stims inc caffeine: psychotic dis | affective disorders |
| Eu32400 | [X]Mild depression | affective disorders |
| Eu32000 | [X]Mild depressive episode | affective disorders |
| Eu53000 | [X]Mild mental/behav disorder assoc with the puerperium NEC | affective disorders |
| Eu3y011 | [X]Mixed affective episode | affective disorders |
| Eu32100 | [X]Moderate depressive episode | affective disorders |
| Eu33z11 | [X]Monopolar depression NOS | affective disorders |
| Eu3..00 | [X]Mood - affective disorders | affective disorders |
| Eu34113 | [X]Neurotic depression | affective disorders |
| Eu31y00 | [X]Other bipolar affective disorders | affective disorders |
| Eu32y00 | [X]Other depressive episodes | affective disorders |
| Eu30y00 | [X]Other manic episodes | affective disorders |
| Eu3y.00 | [X]Other mood affective disorders | affective disorders |
| Eu34y00 | [X]Other persistent mood affective disorders | affective disorders |
| Eu33y00 | [X]Other recurrent depressive disorders | affective disorders |
| Eu3y100 | [X]Other recurrent mood affective disorders | affective disorders |
| Eu3y000 | [X]Other single mood affective disorders | affective disorders |
| Eu3yy00 | [X]Other specified mood affective disorders | affective disorders |
| Eu34z00 | [X]Persistent mood affective disorder, unspecified | affective disorders |
| Eu34.00 | [X]Persistent mood affective disorders | affective disorders |
| Eu53011 | [X]Postnatal depression NOS | affective disorders |
| Eu53012 | [X]Postpartum depression NOS | affective disorders |
| Eu32z13 | [X]Prolonged single episode of reactive depression | affective disorders |
| Eu53z00 | [X]Puerperal mental disorder, unspecified | affective disorders |
| Eu33200 | [X]Recurr depress disorder cur epi severe without psyc sympt | affective disorders |
| Eu32A00 | [X]Recurr major depr ep, severe with psych, psych in remiss | affective disorders |
| Eu33313 | [X]Recurr severe episodes/major depression+psychotic symptom | affective disorders |
| Eu33314 | [X]Recurr severe episodes/psychogenic depressive psychosis | affective disorders |
| Eu3y111 | [X]Recurrent brief depressive episodes | affective disorders |
| Eu33300 | [X]Recurrent depress disorder cur epi severe with psyc symp | affective disorders |
| Eu33.00 | [X]Recurrent depressive disorder | affective disorders |
| Eu33000 | [X]Recurrent depressive disorder, current episode mild | affective disorders |
| Eu33100 | [X]Recurrent depressive disorder, current episode moderate | affective disorders |
| Eu33400 | [X]Recurrent depressive disorder, currently in remission | affective disorders |
| Eu33z00 | [X]Recurrent depressive disorder, unspecified | affective disorders |
| Eu33.11 | [X]Recurrent episodes of depressive reaction | affective disorders |
| Eu33.12 | [X]Recurrent episodes of psychogenic depression | affective disorders |
| Eu33.13 | [X]Recurrent episodes of reactive depression | affective disorders |
| Eu31y12 | [X]Recurrent manic episodes | affective disorders |
| Eu33315 | [X]Recurrent severe episodes of psychotic depression | affective disorders |
| Eu33316 | [X]Recurrent severe episodes/reactive depressive psychosis | affective disorders |
| Eu33.15 | [X]SAD - Seasonal affective disorder | affective disorders |
| Eu33.14 | [X]Seasonal depressive disorder | affective disorders |
| Eu32300 | [X]Severe depressive episode with psychotic symptoms | affective disorders |
| Eu32200 | [X]Severe depressive episode without psychotic symptoms | affective disorders |
| Eu53100 | [X]Severe mental and behav disorder assoc wth puerperium NEC | affective disorders |
| Eu32211 | [X]Single episode agitated depressn w'out psychotic symptoms | affective disorders |
| Eu32212 | [X]Single episode major depression w'out psychotic symptoms | affective disorders |
| Eu32.11 | [X]Single episode of depressive reaction | affective disorders |
| Eu32311 | [X]Single episode of major depression and psychotic symptoms | affective disorders |
| Eu32y12 | [X]Single episode of masked depression NOS | affective disorders |
| Eu32.12 | [X]Single episode of psychogenic depression | affective disorders |
| Eu32312 | [X]Single episode of psychogenic depressive psychosis | affective disorders |
| Eu32313 | [X]Single episode of psychotic depression | affective disorders |
| Eu32.13 | [X]Single episode of reactive depression | affective disorders |
| Eu32314 | [X]Single episode of reactive depressive psychosis | affective disorders |
| Eu32213 | [X]Single episode vital depression w'out psychotic symptoms | affective disorders |
| Eu32900 | [X]Single major depr ep, severe with psych, psych in remiss | affective disorders |
| Eu3z.00 | [X]Unspecified mood affective disorder | affective disorders |
| Eu33214 | [X]Vital depression, recurrent without psychotic symptoms | affective disorders |
| Z4I7.00 | Acknowledging anxiety | anxiety&neurosis |
| E202600 | Acrophobia | anxiety&neurosis |
| E281.00 | Acute fugue state due to acute stress reaction | anxiety&neurosis |
| E280.00 | Acute panic state due to acute stress reaction | anxiety&neurosis |
| E283100 | Acute posttrauma stress state | anxiety&neurosis |
| E28..00 | Acute reaction to stress | anxiety&neurosis |
| E283000 | Acute situational disturbance | anxiety&neurosis |
| E28z.00 | Acute stress reaction NOS | anxiety&neurosis |
| E282.00 | Acute stupor state due to acute stress reaction | anxiety&neurosis |
| E29..00 | Adjustment reaction | anxiety&neurosis |
| E29z.00 | Adjustment reaction NOS | anxiety&neurosis |
| E29y400 | Adjustment reaction due to hospitalisation | anxiety&neurosis |
| E293000 | Adjustment reaction with aggression | anxiety&neurosis |
| E293100 | Adjustment reaction with antisocial behaviour | anxiety&neurosis |
| E292400 | Adjustment reaction with anxious mood | anxiety&neurosis |
| E293200 | Adjustment reaction with destructiveness | anxiety&neurosis |
| E294.00 | Adjustment reaction with disturbance emotion and conduct | anxiety&neurosis |
| E292z00 | Adjustment reaction with disturbance of other emotion NOS | anxiety&neurosis |
| E292y00 | Adjustment reaction with mixed disturbance of emotion | anxiety&neurosis |
| E29y200 | Adjustment reaction with physical symptoms | anxiety&neurosis |
| E293.00 | Adjustment reaction with predominant disturbance of conduct | anxiety&neurosis |
| E292.00 | Adjustment reaction, predominant disturbance other emotions | anxiety&neurosis |
| E292100 | Adolescent emancipation disorder | anxiety&neurosis |
| E202100 | Agoraphobia with panic attacks | anxiety&neurosis |
| E202200 | Agoraphobia without mention of panic attacks | anxiety&neurosis |
| E264011 | Air swallowing - excessive | anxiety&neurosis |
| E203.11 | Anancastic neurosis | anxiety&neurosis |
| E202700 | Animal phobia | anxiety&neurosis |
| 8G52.00 | Antiphobic therapy | anxiety&neurosis |
| Z4L1.00 | Anxiety counselling | anxiety&neurosis |
| 8G94.00 | Anxiety management training | anxiety&neurosis |
| E200z00 | Anxiety state NOS | anxiety&neurosis |
| E200000 | Anxiety state unspecified | anxiety&neurosis |
| E200.00 | Anxiety states | anxiety&neurosis |
| E200300 | Anxiety with depression | anxiety&neurosis |
| E201z11 | Aphonia - hysterical | anxiety&neurosis |
| E201611 | Astasia - abasia, hysterical | anxiety&neurosis |
| E201z12 | Ataxia - hysterical | anxiety&neurosis |
| E290011 | Bereavement reaction | anxiety&neurosis |
| E20y011 | Briquet's disorder | anxiety&neurosis |
| E26y000 | Bruxism (teeth grinding) | anxiety&neurosis |
| E202B00 | Cancer phobia | anxiety&neurosis |
| E262000 | Cardiac neurosis | anxiety&neurosis |
| E200400 | Chronic anxiety | anxiety&neurosis |
| E202800 | Claustrophobia | anxiety&neurosis |
| E28..11 | Combat fatigue | anxiety&neurosis |
| E201B00 | Compensation neurosis | anxiety&neurosis |
| E203000 | Compulsive neurosis | anxiety&neurosis |
| E29y000 | Concentration camp syndrome | anxiety&neurosis |
| E292500 | Culture shock | anxiety&neurosis |
| E202C00 | Dental phobia | anxiety&neurosis |
| E206.00 | Depersonalisation syndrome | anxiety&neurosis |
| Z522400 | Desensitisation - phobia | anxiety&neurosis |
| E201A00 | Dissociative reaction unspecified | anxiety&neurosis |
| E292200 | Early adult emancipation disorder | anxiety&neurosis |
| E29y300 | Elective mutism due to an adjustment reaction | anxiety&neurosis |
| E28z.11 | Examination fear | anxiety&neurosis |
| E202900 | Fear of crowds | anxiety&neurosis |
| E202D00 | Fear of death | anxiety&neurosis |
| E202A00 | Fear of flying | anxiety&neurosis |
| E202E00 | Fear of pregnancy | anxiety&neurosis |
| E201511 | Fit - hysterical | anxiety&neurosis |
| Z522600 | Flooding - obsessional compulsive disorder | anxiety&neurosis |
| E28z.12 | Flying phobia | anxiety&neurosis |
| E201z13 | Ganser's syndrome - hysterical | anxiety&neurosis |
| E200200 | Generalised anxiety disorder | anxiety&neurosis |
| E264.11 | Globus abdominalis | anxiety&neurosis |
| E201612 | Globus hystericus | anxiety&neurosis |
| E290000 | Grief reaction | anxiety&neurosis |
| 146G.00 | H/O: agoraphobia | anxiety&neurosis |
| E207.00 | Hypochondriasis | anxiety&neurosis |
| E201.00 | Hysteria | anxiety&neurosis |
| E201z00 | Hysteria NOS | anxiety&neurosis |
| E201000 | Hysteria unspecified | anxiety&neurosis |
| E201700 | Hysterical amnesia | anxiety&neurosis |
| E201100 | Hysterical blindness | anxiety&neurosis |
| E201200 | Hysterical deafness | anxiety&neurosis |
| E201800 | Hysterical fugue | anxiety&neurosis |
| E201400 | Hysterical paralysis | anxiety&neurosis |
| E201500 | Hysterical seizures | anxiety&neurosis |
| E201300 | Hysterical tremor | anxiety&neurosis |
| E278111 | Muscular headache | anxiety&neurosis |
| E20z.11 | Nervous breakdown | anxiety&neurosis |
| E205.11 | Nervous exhaustion | anxiety&neurosis |
| E205.00 | Neurasthenia - nervous debility | anxiety&neurosis |
| E262200 | Neurocirculatory asthenia | anxiety&neurosis |
| E2z..00 | Neuroses or other mental disorder NOS | anxiety&neurosis |
| E20z.00 | Neurotic disorder NOS | anxiety&neurosis |
| E20..00 | Neurotic disorders | anxiety&neurosis |
| 225J.00 | O/E - panic attack | anxiety&neurosis |
| E203100 | Obsessional neurosis | anxiety&neurosis |
| 1Ba0.00 | Obsessional thoughts | anxiety&neurosis |
| E203z00 | Obsessive-compulsive disorder NOS | anxiety&neurosis |
| E203.00 | Obsessive-compulsive disorders | anxiety&neurosis |
| E283z00 | Other acute stress reaction NOS | anxiety&neurosis |
| E283.00 | Other acute stress reactions | anxiety&neurosis |
| E29y500 | Other adjustment reaction with withdrawal | anxiety&neurosis |
| E29y.00 | Other adjustment reactions | anxiety&neurosis |
| E29yz00 | Other adjustment reactions NOS | anxiety&neurosis |
| E201600 | Other conversion disorder | anxiety&neurosis |
| E20yz00 | Other neurotic disorder NOS | anxiety&neurosis |
| E20y.00 | Other neurotic disorders | anxiety&neurosis |
| E20y200 | Other occupational neurosis | anxiety&neurosis |
| E29y100 | Other post-traumatic stress disorder | anxiety&neurosis |
| E26y.00 | Other psychogenic malfunction | anxiety&neurosis |
| E26yz00 | Other psychogenic malfunction NOS | anxiety&neurosis |
| E2y..00 | Other specified neuroses or other mental disorders | anxiety&neurosis |
| E200111 | Panic attack | anxiety&neurosis |
| E200100 | Panic disorder | anxiety&neurosis |
| 8CAZ000 | Patient given advice about management of anxiety | anxiety&neurosis |
| E201C00 | Phantom pregnancy | anxiety&neurosis |
| Z481.00 | Phobia counselling | anxiety&neurosis |
| E202000 | Phobia unspecified | anxiety&neurosis |
| E202.12 | Phobic anxiety | anxiety&neurosis |
| E202z00 | Phobic disorder NOS | anxiety&neurosis |
| E202.00 | Phobic disorders | anxiety&neurosis |
| F481700 | Photophobia | anxiety&neurosis |
| E26..00 | Physiological malfunction arising from mental factors | anxiety&neurosis |
| ZS7C700 | Post-traumatic mutism | anxiety&neurosis |
| E278.00 | Psychalgia | anxiety&neurosis |
| E278z00 | Psychalgia NOS | anxiety&neurosis |
| E20y300 | Psychasthenic neurosis | anxiety&neurosis |
| E264000 | Psychogenic aerophagy | anxiety&neurosis |
| E261000 | Psychogenic air hunger | anxiety&neurosis |
| E261500 | Psychogenic aphonia | anxiety&neurosis |
| E278200 | Psychogenic backache | anxiety&neurosis |
| E262300 | Psychogenic cardiovascular disorder | anxiety&neurosis |
| E262z00 | Psychogenic cardiovascular symptom NOS | anxiety&neurosis |
| E262.00 | Psychogenic cardiovascular symptoms | anxiety&neurosis |
| E264500 | Psychogenic constipation | anxiety&neurosis |
| E261100 | Psychogenic cough | anxiety&neurosis |
| E264300 | Psychogenic diarrhoea | anxiety&neurosis |
| E265200 | Psychogenic dysmenorrhea | anxiety&neurosis |
| E264400 | Psychogenic dyspepsia | anxiety&neurosis |
| E265300 | Psychogenic dysuria | anxiety&neurosis |
| E264z00 | Psychogenic gastrointestinal tract symptom NOS | anxiety&neurosis |
| E264.00 | Psychogenic gastrointestinal tract symptoms | anxiety&neurosis |
| E265z00 | Psychogenic genitourinary tract symptom NOS | anxiety&neurosis |
| E265.00 | Psychogenic genitourinary tract symptoms | anxiety&neurosis |
| E261200 | Psychogenic hiccough | anxiety&neurosis |
| E261300 | Psychogenic hyperventilation | anxiety&neurosis |
| E260.00 | Psychogenic musculoskeletal symptoms | anxiety&neurosis |
| E260z00 | Psychogenic musculoskeletal symptoms NOS | anxiety&neurosis |
| E278000 | Psychogenic pain unspecified | anxiety&neurosis |
| E260000 | Psychogenic paralysis | anxiety&neurosis |
| E263000 | Psychogenic pruritus | anxiety&neurosis |
| E261z00 | Psychogenic respiratory symptom NOS | anxiety&neurosis |
| E261.00 | Psychogenic respiratory symptoms | anxiety&neurosis |
| E263.00 | Psychogenic skin symptoms | anxiety&neurosis |
| E263z00 | Psychogenic skin symptoms NOS | anxiety&neurosis |
| E267.00 | Psychogenic symptom of special sense organ | anxiety&neurosis |
| E260100 | Psychogenic torticollis | anxiety&neurosis |
| E265100 | Psychogenic vaginismus | anxiety&neurosis |
| E261400 | Psychogenic yawning | anxiety&neurosis |
| E26z.00 | Psychosomatic disorder NOS | anxiety&neurosis |
| Z4I7100 | Recognising anxiety | anxiety&neurosis |
| E200500 | Recurrent anxiety | anxiety&neurosis |
| 8HHp.00 | Referral for guided self-help for anxiety | anxiety&neurosis |
| 8IH5300 | Referral for guided self-help for anxiety declined | anxiety&neurosis |
| E292000 | Separation anxiety disorder | anxiety&neurosis |
| E202300 | Social phobia, fear of eating in public | anxiety&neurosis |
| E202400 | Social phobia, fear of public speaking | anxiety&neurosis |
| E202500 | Social phobia, fear of public washing | anxiety&neurosis |
| E202.11 | Social phobic disorders | anxiety&neurosis |
| E20y000 | Somatization disorder | anxiety&neurosis |
| E292311 | Specific academic or work inhibition | anxiety&neurosis |
| E292300 | Specific academic or work inhibition | anxiety&neurosis |
| E292312 | Specific work inhibition | anxiety&neurosis |
| E264311 | Spurious diarrhoea | anxiety&neurosis |
| E28z.13 | Stage fright | anxiety&neurosis |
| E284.00 | Stress reaction causing mixed disturbance of emotion/conduct | anxiety&neurosis |
| E278100 | Tension headache | anxiety&neurosis |
| E205.12 | Tired all the time | anxiety&neurosis |
| E202z11 | Weight fixation | anxiety&neurosis |
| E20y100 | Writer's cramp neurosis | anxiety&neurosis |
| Eu40211 | [X]Acrophobia | anxiety&neurosis |
| Eu43011 | [X]Acute crisis reaction | anxiety&neurosis |
| Eu43300 | [X]Acute post-traumatic stress disorder follow military comb | anxiety&neurosis |
| Eu43012 | [X]Acute reaction to stress | anxiety&neurosis |
| Eu43000 | [X]Acute stress reaction | anxiety&neurosis |
| Eu43200 | [X]Adjustment disorders | anxiety&neurosis |
| Eu40000 | [X]Agoraphobia | anxiety&neurosis |
| Eu40011 | [X]Agoraphobia without history of panic disorder | anxiety&neurosis |
| Eu42.11 | [X]Anankastic neurosis | anxiety&neurosis |
| Eu40212 | [X]Animal phobias | anxiety&neurosis |
| Eu41z11 | [X]Anxiety NOS | anxiety&neurosis |
| Eu41z00 | [X]Anxiety disorder, unspecified | anxiety&neurosis |
| Eu41y11 | [X]Anxiety hysteria | anxiety&neurosis |
| Eu41111 | [X]Anxiety neurosis | anxiety&neurosis |
| Eu41112 | [X]Anxiety reaction | anxiety&neurosis |
| Eu41113 | [X]Anxiety state | anxiety&neurosis |
| Eu45211 | [X]Body dysmorphic disorder | anxiety&neurosis |
| Eu46y11 | [X]Briquet's disorder | anxiety&neurosis |
| Eu45012 | [X]Briquet's syndrome | anxiety&neurosis |
| Eu45311 | [X]Cardiac neurosis | anxiety&neurosis |
| Eu93y12 | [X]Childhood overanxious disorder | anxiety&neurosis |
| Eu43400 | [X]Chron post-traumatic stress disorder follow military comb | anxiety&neurosis |
| Eu40213 | [X]Claustrophobia | anxiety&neurosis |
| Eu43013 | [X]Combat fatigue | anxiety&neurosis |
| Eu44.11 | [X]Conversion hysteria | anxiety&neurosis |
| Eu44.12 | [X]Conversion reaction | anxiety&neurosis |
| Eu43014 | [X]Crisis state | anxiety&neurosis |
| Eu43211 | [X]Culture shock | anxiety&neurosis |
| Eu45312 | [X]Da Costa's syndrome | anxiety&neurosis |
| Eu43500 | [X]Delayed post-traumat stress disorder follow military comb | anxiety&neurosis |
| Eu46100 | [X]Depersonalization - derealization syndrome | anxiety&neurosis |
| Eu46y12 | [X]Dhat syndrome | anxiety&neurosis |
| Eu44z00 | [X]Dissociative [conversion] disorder, unspecified | anxiety&neurosis |
| Eu44.00 | [X]Dissociative [conversion] disorders | anxiety&neurosis |
| Eu44000 | [X]Dissociative amnesia | anxiety&neurosis |
| Eu44600 | [X]Dissociative anaesthesia and sensory loss | anxiety&neurosis |
| Eu44500 | [X]Dissociative convulsions | anxiety&neurosis |
| Eu44100 | [X]Dissociative fugue | anxiety&neurosis |
| Eu44400 | [X]Dissociative motor disorders | anxiety&neurosis |
| Eu44200 | [X]Dissociative stupor | anxiety&neurosis |
| Eu51511 | [X]Dream anxiety disorder | anxiety&neurosis |
| Eu45212 | [X]Dysmorphophobia nondelusional | anxiety&neurosis |
| Eu46011 | [X]Fatigue syndrome | anxiety&neurosis |
| Eu44y11 | [X]Ganser's syndrome | anxiety&neurosis |
| Eu45313 | [X]Gastric neurosis | anxiety&neurosis |
| Eu41100 | [X]Generalized anxiety disorder | anxiety&neurosis |
| Eu45511 | [X]Globus hystericus | anxiety&neurosis |
| Eu45y12 | [X]Globus hystericus | anxiety&neurosis |
| Eu45500 | [X]Globus pharyngeus | anxiety&neurosis |
| Eu43212 | [X]Grief reaction | anxiety&neurosis |
| Eu43213 | [X]Hospitalism in children | anxiety&neurosis |
| Eu45200 | [X]Hypochondriacal disorder | anxiety&neurosis |
| Eu45213 | [X]Hypochondriacal neurosis | anxiety&neurosis |
| Eu45214 | [X]Hypochondriasis | anxiety&neurosis |
| Eu44.13 | [X]Hysteria | anxiety&neurosis |
| Eu41211 | [X]Mild anxiety depression | anxiety&neurosis |
| Eu41200 | [X]Mixed anxiety and depressive disorder | anxiety&neurosis |
| Eu44700 | [X]Mixed dissociative [conversion] disorders | anxiety&neurosis |
| Eu42200 | [X]Mixed obsessional thoughts and acts | anxiety&neurosis |
| Eu45011 | [X]Multiple psychosomatic disorder | anxiety&neurosis |
| Eu40300 | [X]Needle phobia | anxiety&neurosis |
| Eu46000 | [X]Neurasthenia | anxiety&neurosis |
| Eu45314 | [X]Neurocirculatory asthenia | anxiety&neurosis |
| Eu46z11 | [X]Neurosis NOS | anxiety&neurosis |
| Eu46z00 | [X]Neurotic disorder, unspecified | anxiety&neurosis |
| Eu4..00 | [X]Neurotic, stress - related and somoform disorders | anxiety&neurosis |
| Eu45215 | [X]Nosophobia | anxiety&neurosis |
| Eu42.00 | [X]Obsessive - compulsive disorder | anxiety&neurosis |
| Eu42z00 | [X]Obsessive-compulsive disorder, unspecified | anxiety&neurosis |
| Eu42.12 | [X]Obsessive-compulsive neurosis | anxiety&neurosis |
| Eu46y13 | [X]Occupational neurosis, including writer's cramp | anxiety&neurosis |
| Eu05400 | [X]Organic anxiety disorder | anxiety&neurosis |
| Eu41.00 | [X]Other anxiety disorders | anxiety&neurosis |
| Eu44y00 | [X]Other dissociative [conversion] disorders | anxiety&neurosis |
| Eu41300 | [X]Other mixed anxiety disorders | anxiety&neurosis |
| Eu46.00 | [X]Other neurotic disorders | anxiety&neurosis |
| Eu42y00 | [X]Other obsessive-compulsive disorders | anxiety&neurosis |
| Eu40y00 | [X]Other phobic anxiety disorders | anxiety&neurosis |
| Eu43y00 | [X]Other reactions to severe stress | anxiety&neurosis |
| Eu45y00 | [X]Other somatoform disorders | anxiety&neurosis |
| Eu41y00 | [X]Other specified anxiety disorders | anxiety&neurosis |
| Eu46y00 | [X]Other specified neurotic disorders | anxiety&neurosis |
| Eu41011 | [X]Panic attack | anxiety&neurosis |
| Eu41000 | [X]Panic disorder [episodic paroxysmal anxiety] | anxiety&neurosis |
| Eu40012 | [X]Panic disorder with agoraphobia | anxiety&neurosis |
| Eu41012 | [X]Panic state | anxiety&neurosis |
| Eu34114 | [X]Persistant anxiety depression | anxiety&neurosis |
| Eu45400 | [X]Persistent somatoform pain disorder | anxiety&neurosis |
| Eu40z11 | [X]Phobia NOS | anxiety&neurosis |
| Eu93100 | [X]Phobic anxiety disorder of childhood | anxiety&neurosis |
| Eu40z00 | [X]Phobic anxiety disorder, unspecified | anxiety&neurosis |
| Eu40.00 | [X]Phobic anxiety disorders | anxiety&neurosis |
| Eu40z12 | [X]Phobic state NOS | anxiety&neurosis |
| Eu43100 | [X]Post - traumatic stress disorder | anxiety&neurosis |
| Eu42100 | [X]Predominantly compulsive acts [obsessional rituals] | anxiety&neurosis |
| Eu42000 | [X]Predominantly obsessional thoughts or ruminations | anxiety&neurosis |
| Eu44511 | [X]Pseudoseizures | anxiety&neurosis |
| Eu45411 | [X]Psychalgia | anxiety&neurosis |
| Eu46y14 | [X]Psychasthenia | anxiety&neurosis |
| Eu46y15 | [X]Psychasthenia neurosis | anxiety&neurosis |
| Eu43015 | [X]Psychic shock | anxiety&neurosis |
| Eu45324 | [X]Psychogenic IBS | anxiety&neurosis |
| Eu44411 | [X]Psychogenic aphonia | anxiety&neurosis |
| Eu45412 | [X]Psychogenic backache | anxiety&neurosis |
| Eu44y13 | [X]Psychogenic confusion | anxiety&neurosis |
| Eu45316 | [X]Psychogenic cough | anxiety&neurosis |
| Eu44611 | [X]Psychogenic deafness | anxiety&neurosis |
| Eu45317 | [X]Psychogenic diarrhoea | anxiety&neurosis |
| Eu45y11 | [X]Psychogenic dysmenorrhoea | anxiety&neurosis |
| Eu45318 | [X]Psychogenic dyspepsia | anxiety&neurosis |
| Eu44412 | [X]Psychogenic dysphonia | anxiety&neurosis |
| Eu45319 | [X]Psychogenic dysuria | anxiety&neurosis |
| Eu45320 | [X]Psychogenic flatulence | anxiety&neurosis |
| Eu45323 | [X]Psychogenic freq micturit | anxiety&neurosis |
| Eu45413 | [X]Psychogenic headache | anxiety&neurosis |
| Eu45321 | [X]Psychogenic hiccough | anxiety&neurosis |
| Eu45322 | [X]Psychogenic hyperventilat | anxiety&neurosis |
| Eu45y13 | [X]Psychogenic pruritis | anxiety&neurosis |
| Eu45325 | [X]Psychogenic pylorospasm | anxiety&neurosis |
| Eu46y16 | [X]Psychogenic syncope | anxiety&neurosis |
| Eu45y14 | [X]Psychogenic torticollis | anxiety&neurosis |
| Eu44y14 | [X]Psychogenic twilight state | anxiety&neurosis |
| Eu45z11 | [X]Psychosomatic disorder NOS | anxiety&neurosis |
| Eu43.00 | [X]Reaction to severe stress, and adjustment disorders | anxiety&neurosis |
| Eu43z00 | [X]Reaction to severe stress, unspecified | anxiety&neurosis |
| Eu93000 | [X]Separation anxiety disorder of childhood | anxiety&neurosis |
| Eu40214 | [X]Simple phobia | anxiety&neurosis |
| Eu93200 | [X]Social anxiety disorder of childhood | anxiety&neurosis |
| Eu40112 | [X]Social neurosis | anxiety&neurosis |
| Eu40100 | [X]Social phobias | anxiety&neurosis |
| Eu45000 | [X]Somatization disorder | anxiety&neurosis |
| Eu45300 | [X]Somatoform autonomic dysfunction | anxiety&neurosis |
| Eu45z00 | [X]Somatoform disorder, unspecified | anxiety&neurosis |
| Eu45.00 | [X]Somatoform disorders | anxiety&neurosis |
| Eu45414 | [X]Somatoform pain disorder | anxiety&neurosis |
| Eu40200 | [X]Specific (isolated) phobias | anxiety&neurosis |
| Eu45y15 | [X]Teeth-grinding | anxiety&neurosis |
| Eu44300 | [X]Trance and possession disorders | anxiety&neurosis |
| Eu43111 | [X]Traumatic neurosis | anxiety&neurosis |
| Eu45111 | [X]Undifferentiated psychosomatic disorder | anxiety&neurosis |
| Eu45100 | [X]Undifferentiated somatoform disorder | anxiety&neurosis |
| E271.00 | Anorexia nervosa | eating disorders |
| 1612.11 | Anorexia symptom | eating disorders |
| 1612.00 | Appetite loss - anorexia | eating disorders |
| 1FF..00 | Binge eating | eating disorders |
| E275100 | Bulimia (non-organic overeating) | eating disorders |
| E275111 | Compulsive eating disorder | eating disorders |
| E264200 | Cyclical vomiting - psychogenic | eating disorders |
| ZC2CD00 | Dietary advice for eating disorder | eating disorders |
| 1G2..00 | Dissatisfied with body image | eating disorders |
| Z7D1200 | Distorted body image | eating disorders |
| Z4B5.00 | Eating disorder counselling | eating disorders |
| 1614.00 | Excessive eating - polyphagia | eating disorders |
| 1467.00 | H/O: anorexia nervosa | eating disorders |
| 1614.11 | Hyperalimentation - symptom | eating disorders |
| Fy05.00 | Nocturnal sleep-related eating disorder | eating disorders |
| E275z00 | Non-organic eating disorder NOS | eating disorders |
| E275.00 | Other and unspecified non-organic eating disorders | eating disorders |
| E275y00 | Other specified non-organic eating disorder | eating disorders |
| 1614.12 | Polyphagia symptom | eating disorders |
| 8HTN.00 | Referral to eating disorders clinic | eating disorders |
| 9Nk9.00 | Seen in eating disorder clinic | eating disorders |
| SN42100 | Starvation | eating disorders |
| 1JZ..00 | Suspected binge eating disorder | eating disorders |
| E275000 | Unspecified non-organic eating disorder | eating disorders |
| R030.00 | [D]Anorexia | eating disorders |
| R030z00 | [D]Anorexia NOS | eating disorders |
| R036011 | [D]Bulimia NOS | eating disorders |
| R036000 | [D]Excessive eating | eating disorders |
| R036100 | [D]Hyperalimentation | eating disorders |
| R036.00 | [D]Polyphagia | eating disorders |
| R036z00 | [D]Polyphagia NOS | eating disorders |
| Eu50000 | [X]Anorexia nervosa | eating disorders |
| Eu50100 | [X]Atypical anorexia nervosa | eating disorders |
| Eu50300 | [X]Atypical bulimia nervosa | eating disorders |
| Eu50211 | [X]Bulimia NOS | eating disorders |
| Eu50200 | [X]Bulimia nervosa | eating disorders |
| Eu50z00 | [X]Eating disorder, unspecified | eating disorders |
| Eu50.00 | [X]Eating disorders | eating disorders |
| Eu50212 | [X]Hyperorexia nervosa | eating disorders |
| Eu50y00 | [X]Other eating disorders | eating disorders |
| Eu50400 | [X]Overeating associated with other psychological disturbncs | eating disorders |
| Eu50y11 | [X]Pica in adults | eating disorders |
| Eu50411 | [X]Psychogenic overeating | eating disorders |
| U1B3.11 | [X]Starvation | eating disorders |
| E211.00 | Affective personality disorder | personality disorders |
| E211z00 | Affective personality disorder NOS | personality disorders |
| E213.11 | Aggressive personality | personality disorders |
| E2C0.00 | Aggressive unsocial conduct disorder | personality disorders |
| E217.11 | Amoral personality | personality disorders |
| E214.11 | Anancastic personality | personality disorders |
| E214000 | Anankastic personality | personality disorders |
| E217.00 | Antisocial or sociopathic personality disorder | personality disorders |
| E216.11 | Asthenic personality | personality disorders |
| E21y100 | Avoidant personality disorder | personality disorders |
| E21y200 | Borderline personality disorder | personality disorders |
| E214z00 | Compulsive personality disorder NOS | personality disorders |
| E214.00 | Compulsive personality disorders | personality disorders |
| E211300 | Cyclothymic personality disorder | personality disorders |
| E216.12 | Dependent personality | personality disorders |
| E211200 | Depressive personality disorder | personality disorders |
| E2C..00 | Disturbance of conduct NEC | personality disorders |
| E21y400 | Eccentric personality disorder | personality disorders |
| E215200 | Emotionally unstable personality | personality disorders |
| E213.00 | Explosive personality disorder | personality disorders |
| E215z00 | Histrionic personality disorder NOS | personality disorders |
| E215.00 | Histrionic personality disorders | personality disorders |
| E211100 | Hypomanic personality disorder | personality disorders |
| E215.11 | Hysterical personality disorders | personality disorders |
| E21y500 | Immature personality disorder | personality disorders |
| E216.00 | Inadequate personality disorder | personality disorders |
| E216.13 | Labile personality | personality disorders |
| E21y600 | Masochistic personality disorder | personality disorders |
| E2C4.00 | Mixed disturbance of conduct and emotion | personality disorders |
| E215100 | Munchausen's syndrome | personality disorders |
| E21y000 | Narcissistic personality disorder | personality disorders |
| E21y711 | Neurotic personality | personality disorders |
| E21..11 | Neurotic personality disorder | personality disorders |
| E214100 | Obsessional personality | personality disorders |
| E21yz00 | Other personality disorder NOS | personality disorders |
| E21y.00 | Other personality disorders | personality disorders |
| E210.00 | Paranoid personality disorder | personality disorders |
| E21y300 | Passive-aggressive personality disorder | personality disorders |
| E2C3100 | Pathological gambling | personality disorders |
| E21z.00 | Personality disorder NOS | personality disorders |
| E21..00 | Personality disorders | personality disorders |
| E21y700 | Psychoneurotic personality disorder | personality disorders |
| E21z.11 | Psychopathic personality | personality disorders |
| E212.00 | Schizoid personality disorder | personality disorders |
| E212z00 | Schizoid personality disorder NOS | personality disorders |
| E212200 | Schizotypal personality | personality disorders |
| E211000 | Unspecified affective personality disorder | personality disorders |
| E215000 | Unspecified histrionic personality disorder | personality disorders |
| E212000 | Unspecified schizoid personality disorder | personality disorders |
| Eu6y111 | [X] Munchausens syndrome | personality disorders |
| Eu60800 | [X]Addictive personality | personality disorders |
| Eu94211 | [X]Affectionless psychopathy | personality disorders |
| Eu34011 | [X]Affective personality disorder | personality disorders |
| Eu60311 | [X]Aggressive personality disorder | personality disorders |
| Eu60211 | [X]Amoral personality disorder | personality disorders |
| Eu60500 | [X]Anankastic personality disorder | personality disorders |
| Eu60212 | [X]Antisocial personality disorder | personality disorders |
| Eu60600 | [X]Anxious [avoidant] personality disorder | personality disorders |
| Eu60213 | [X]Asocial personality disorder | personality disorders |
| Eu60711 | [X]Asthenic personality disorder | personality disorders |
| Eu84511 | [X]Autistic psychopathy | personality disorders |
| Eu60312 | [X]Borderline personality disorder | personality disorders |
| Eu60z11 | [X]Character neurosis NOS | personality disorders |
| Eu62y11 | [X]Chronic pain personality syndrome | personality disorders |
| Eu63011 | [X]Compulsive gambling | personality disorders |
| Eu60511 | [X]Compulsive personality disorder | personality disorders |
| Eu34012 | [X]Cycloid personality | personality disorders |
| Eu34013 | [X]Cyclothymic personality | personality disorders |
| Eu60700 | [X]Dependent personality disorder | personality disorders |
| Eu34112 | [X]Depressive personality disorder | personality disorders |
| Eu6..00 | [X]Disorders of adult personality and behaviour | personality disorders |
| Eu60200 | [X]Dissocial personality disorder | personality disorders |
| Eu60y11 | [X]Eccentric personality disorder | personality disorders |
| Eu6y000 | [X]Elaboration of physical symptoms for psychological reason | personality disorders |
| Eu60300 | [X]Emotionally unstable personality disorder | personality disorders |
| Eu62000 | [X]Enduring personality change after catastrophic experience | personality disorders |
| Eu62100 | [X]Enduring personality change after psychiatric illness | personality disorders |
| Eu62.00 | [X]Enduring personality change not attrib to brain damag/dis | personality disorders |
| Eu62z00 | [X]Enduring personality change, unspecified | personality disorders |
| Eu60011 | [X]Expansive paranoid personality disorder | personality disorders |
| Eu60313 | [X]Explosive personality disorder | personality disorders |
| Eu6y300 | [X]Factitious disorder | personality disorders |
| Eu63z00 | [X]Habit and impulse disorder, unspecified | personality disorders |
| Eu63.00 | [X]Habit and impulse disorders | personality disorders |
| Eu60y12 | [X]Haltlose type personality disorder | personality disorders |
| Eu60400 | [X]Histrionic personality disorder | personality disorders |
| Eu60411 | [X]Hysterical personality disorder | personality disorders |
| Eu60y13 | [X]Immature personality disorder | personality disorders |
| Eu60712 | [X]Inadequate personality disorder | personality disorders |
| Eu6y100 | [X]Intent product/feign of symptom/disab eith physical/psych | personality disorders |
| Eu61.00 | [X]Mixed and other personality disorders | personality disorders |
| Eu6y200 | [X]Munchausen's by proxy | personality disorders |
| Eu60y14 | [X]Narcissistic personality disorder | personality disorders |
| Eu60512 | [X]Obsessional personality disorder | personality disorders |
| Eu60513 | [X]Obsessive-compulsive personality disorder | personality disorders |
| Eu06000 | [X]Organic personality disorder | personality disorders |
| Eu06011 | [X]Organic pseudopsychopathic personality | personality disorders |
| Eu6y.00 | [X]Other disorders of adult personality and behaviour | personality disorders |
| Eu62y00 | [X]Other enduring personality changes | personality disorders |
| Eu63y00 | [X]Other habit and impulse disorders | personality disorders |
| Eu60y00 | [X]Other specific personality disorders | personality disorders |
| Eu6yy00 | [X]Other specified disorders of adult personality/behaviour | personality disorders |
| Eu60000 | [X]Paranoid personality disorder | personality disorders |
| Eu60713 | [X]Passive personality disorder | personality disorders |
| Eu63100 | [X]Pathological fire-setting | personality disorders |
| Eu63000 | [X]Pathological gambling | personality disorders |
| Eu60z12 | [X]Pathological personality NOS | personality disorders |
| Eu63200 | [X]Pathological stealing | personality disorders |
| Eu62013 | [X]Personality change aft prolong captiv+possib/being killed | personality disorders |
| Eu62011 | [X]Personality change after concentration camp experiences | personality disorders |
| Eu62012 | [X]Personality change after disasters | personality disorders |
| Eu62015 | [X]Personality change after torture | personality disorders |
| Eu60z00 | [X]Personality disorder, unspecified | personality disorders |
| Eu62014 | [X]Personlty chang aft expos life-threat sit/victim/terrorsm | personality disorders |
| Eu60412 | [X]Psychoinfantile personality disorder | personality disorders |
| Eu60y16 | [X]Psychoneurotic personality disorder | personality disorders |
| Eu60214 | [X]Psychopathic personality disorder | personality disorders |
| Eu60013 | [X]Querulant personality disorder | personality disorders |
| Eu60100 | [X]Schizoid personality disorder | personality disorders |
| Eu21.18 | [X]Schizotypal personality disorder | personality disorders |
| Eu60714 | [X]Self defeating personality disorder | personality disorders |
| Eu60014 | [X]Sensitive paranoid personality disorder | personality disorders |
| Eu60215 | [X]Sociopathic personality disorder | personality disorders |
| Eu60.00 | [X]Specific personality disorders | personality disorders |
| Eu63300 | [X]Trichotillomania | personality disorders |
| Eu6z.00 | [X]Unspecified disorder of adult personality and behaviour | personality disorders |
| E102400 | Acute exacerbation of chronic catatonic schizophrenia | schizophrenia spectrum |
| E101400 | Acute exacerbation of chronic hebephrenic schizophrenia | schizophrenia spectrum |
| E103400 | Acute exacerbation of chronic paranoid schizophrenia | schizophrenia spectrum |
| E107400 | Acute exacerbation of chronic schizo-affective schizophrenia | schizophrenia spectrum |
| E100400 | Acute exacerbation of chronic schizophrenia | schizophrenia spectrum |
| E103300 | Acute exacerbation of subchronic paranoid schizophrenia | schizophrenia spectrum |
| E100300 | Acute exacerbation of subchronic schizophrenia | schizophrenia spectrum |
| E107300 | Acute exacerbation subchronic schizo-affective schizophrenia | schizophrenia spectrum |
| E131.00 | Acute hysterical psychosis | schizophrenia spectrum |
| E133.00 | Acute paranoid reaction | schizophrenia spectrum |
| E104.00 | Acute schizophrenic episode | schizophrenia spectrum |
| E10y000 | Atypical schizophrenia | schizophrenia spectrum |
| E133.11 | Bouffee delirante | schizophrenia spectrum |
| E13y100 | Brief reactive psychosis | schizophrenia spectrum |
| E102.00 | Catatonic schizophrenia | schizophrenia spectrum |
| E102z00 | Catatonic schizophrenia NOS | schizophrenia spectrum |
| E102500 | Catatonic schizophrenia in remission | schizophrenia spectrum |
| E10y.11 | Cenesthopathic schizophrenia | schizophrenia spectrum |
| E105200 | Chronic latent schizophrenia | schizophrenia spectrum |
| E121.00 | Chronic paranoid psychosis | schizophrenia spectrum |
| E103200 | Chronic paranoid schizophrenia | schizophrenia spectrum |
| E107200 | Chronic schizo-affective schizophrenia | schizophrenia spectrum |
| E100200 | Chronic schizophrenic | schizophrenia spectrum |
| E10y100 | Coenesthopathic schizophrenia | schizophrenia spectrum |
| E107.11 | Cyclic schizophrenia | schizophrenia spectrum |
| 1BH..11 | Delusion | schizophrenia spectrum |
| 1BH0.00 | Delusion of persecution | schizophrenia spectrum |
| 1BH..00 | Delusions | schizophrenia spectrum |
| E141.00 | Disintegrative psychosis | schizophrenia spectrum |
| E123.11 | Folie a deux | schizophrenia spectrum |
| 1BH1.00 | Grandiose delusions | schizophrenia spectrum |
| 146H.00 | H/O: psychosis | schizophrenia spectrum |
| 1464.00 | H/O: schizophrenia | schizophrenia spectrum |
| 1B1E.00 | Hallucinations | schizophrenia spectrum |
| E101.00 | Hebephrenic schizophrenia | schizophrenia spectrum |
| E101z00 | Hebephrenic schizophrenia NOS | schizophrenia spectrum |
| E101500 | Hebephrenic schizophrenia in remission | schizophrenia spectrum |
| 1BH2.00 | Ideas of reference | schizophrenia spectrum |
| E105.00 | Latent schizophrenia | schizophrenia spectrum |
| E105z00 | Latent schizophrenia NOS | schizophrenia spectrum |
| E105500 | Latent schizophrenia in remission | schizophrenia spectrum |
| E1...00 | Non-organic psychoses | schizophrenia spectrum |
| E1z..00 | Non-organic psychosis NOS | schizophrenia spectrum |
| E13z.00 | Nonorganic psychosis NOS | schizophrenia spectrum |
| 225F.00 | O/E - delusion of persecution | schizophrenia spectrum |
| 225E.00 | O/E - paranoid delusions | schizophrenia spectrum |
| E104.11 | Oneirophrenia | schizophrenia spectrum |
| E11zz00 | Other affective psychosis NOS | schizophrenia spectrum |
| E11z.00 | Other and unspecified affective psychoses | schizophrenia spectrum |
| E13..00 | Other nonorganic psychoses | schizophrenia spectrum |
| E12y.00 | Other paranoid states | schizophrenia spectrum |
| E12yz00 | Other paranoid states NOS | schizophrenia spectrum |
| E13y.00 | Other reactive psychoses | schizophrenia spectrum |
| E13yz00 | Other reactive psychoses NOS | schizophrenia spectrum |
| E10y.00 | Other schizophrenia | schizophrenia spectrum |
| E10yz00 | Other schizophrenia NOS | schizophrenia spectrum |
| E1y..00 | Other specified non-organic psychoses | schizophrenia spectrum |
| E12y000 | Paranoia querulans | schizophrenia spectrum |
| 1BH3.00 | Paranoid ideation | schizophrenia spectrum |
| E12z.00 | Paranoid psychosis NOS | schizophrenia spectrum |
| E103.00 | Paranoid schizophrenia | schizophrenia spectrum |
| E103z00 | Paranoid schizophrenia NOS | schizophrenia spectrum |
| E103500 | Paranoid schizophrenia in remission | schizophrenia spectrum |
| E12..00 | Paranoid states | schizophrenia spectrum |
| E122.00 | Paraphrenia | schizophrenia spectrum |
| 286..11 | Poor insight into psychotic condition | schizophrenia spectrum |
| E134.00 | Psychogenic paranoid psychosis | schizophrenia spectrum |
| E14..00 | Psychoses with origin in childhood | schizophrenia spectrum |
| 212X.00 | Psychosis resolved | schizophrenia spectrum |
| 285..11 | Psychotic condition, insight present | schizophrenia spectrum |
| E13z.11 | Psychotic episode NOS | schizophrenia spectrum |
| E130.11 | Psychotic reactive depression | schizophrenia spectrum |
| E132.00 | Reactive confusion | schizophrenia spectrum |
| E13..11 | Reactive psychoses | schizophrenia spectrum |
| 8HHs.00 | Referral to psychosis early intervention service | schizophrenia spectrum |
| E141100 | Residual disintegrative psychoses | schizophrenia spectrum |
| E106.00 | Residual schizophrenia | schizophrenia spectrum |
| E107.00 | Schizo-affective schizophrenia | schizophrenia spectrum |
| E107z00 | Schizo-affective schizophrenia NOS | schizophrenia spectrum |
| E107500 | Schizo-affective schizophrenia in remission | schizophrenia spectrum |
| E10z.00 | Schizophrenia NOS | schizophrenia spectrum |
| E100500 | Schizophrenia in remission | schizophrenia spectrum |
| 212W.00 | Schizophrenia resolved | schizophrenia spectrum |
| E100.11 | Schizophrenia simplex | schizophrenia spectrum |
| E10..00 | Schizophrenic disorders | schizophrenia spectrum |
| ZS7C611 | Schizophrenic language | schizophrenia spectrum |
| E123.00 | Shared paranoid disorder | schizophrenia spectrum |
| E120.00 | Simple paranoid state | schizophrenia spectrum |
| E100.00 | Simple schizophrenia | schizophrenia spectrum |
| E100z00 | Simple schizophrenia NOS | schizophrenia spectrum |
| E102100 | Subchronic catatonic schizophrenia | schizophrenia spectrum |
| E103100 | Subchronic paranoid schizophrenia | schizophrenia spectrum |
| E107100 | Subchronic schizo-affective schizophrenia | schizophrenia spectrum |
| E100100 | Subchronic schizophrenia | schizophrenia spectrum |
| E11z000 | Unspecified affective psychoses NOS | schizophrenia spectrum |
| E102000 | Unspecified catatonic schizophrenia | schizophrenia spectrum |
| E101000 | Unspecified hebephrenic schizophrenia | schizophrenia spectrum |
| E105000 | Unspecified latent schizophrenia | schizophrenia spectrum |
| E103000 | Unspecified paranoid schizophrenia | schizophrenia spectrum |
| E107000 | Unspecified schizo-affective schizophrenia | schizophrenia spectrum |
| E100000 | Unspecified schizophrenia | schizophrenia spectrum |
| F481K00 | Visual hallucinations | schizophrenia spectrum |
| R001.00 | [D]Hallucinations | schizophrenia spectrum |
| R001z00 | [D]Hallucinations NOS | schizophrenia spectrum |
| R001000 | [D]Hallucinations, auditory | schizophrenia spectrum |
| R001100 | [D]Hallucinations, gustatory | schizophrenia spectrum |
| R001200 | [D]Hallucinations, olfactory | schizophrenia spectrum |
| R001300 | [D]Hallucinations, tactile | schizophrenia spectrum |
| R001400 | [D]Visual hallucinations | schizophrenia spectrum |
| ZV11000 | [V]Personal history of schizophrenia | schizophrenia spectrum |
| Eu02z12 | [X] Presenile psychosis NOS | schizophrenia spectrum |
| Eu23z00 | [X]Acute and transient psychotic disorder, unspecified | schizophrenia spectrum |
| Eu23.00 | [X]Acute and transient psychotic disorders | schizophrenia spectrum |
| Eu23100 | [X]Acute polymorphic psychot disord with symp of schizophren | schizophrenia spectrum |
| Eu23000 | [X]Acute polymorphic psychot disord without symp of schizoph | schizophrenia spectrum |
| Eu23200 | [X]Acute schizophrenia-like psychotic disorder | schizophrenia spectrum |
| Eu20311 | [X]Atypical schizophrenia | schizophrenia spectrum |
| Eu21.12 | [X]Borderline schizophrenia | schizophrenia spectrum |
| Eu23011 | [X]Bouffee delirante | schizophrenia spectrum |
| Eu23z11 | [X]Brief reactive psychosis NOS | schizophrenia spectrum |
| Eu23211 | [X]Brief schizophreniform disorder | schizophrenia spectrum |
| Eu23212 | [X]Brief schizophrenifrm psych | schizophrenia spectrum |
| Eu22111 | [X]Capgras syndrome | schizophrenia spectrum |
| Eu20200 | [X]Catatonic schizophrenia | schizophrenia spectrum |
| Eu20211 | [X]Catatonic stupor | schizophrenia spectrum |
| Eu2y.11 | [X]Chronic hallucinatory psychosis | schizophrenia spectrum |
| Eu20511 | [X]Chronic undifferentiated schizophrenia | schizophrenia spectrum |
| Eu22200 | [X]Cotard syndrome | schizophrenia spectrum |
| Eu25211 | [X]Cyclic schizophrenia | schizophrenia spectrum |
| Eu23012 | [X]Cycloid psychosis | schizophrenia spectrum |
| Eu23112 | [X]Cycloid psychosis with symptoms of schizophrenia | schizophrenia spectrum |
| Eu22000 | [X]Delusional disorder | schizophrenia spectrum |
| Eu22y11 | [X]Delusional dysmorphophobia | schizophrenia spectrum |
| Eu22100 | [X]Delusional misidentification syndrome | schizophrenia spectrum |
| Eu84312 | [X]Disintegrative psychosis | schizophrenia spectrum |
| Eu20111 | [X]Disorganised schizophrenia | schizophrenia spectrum |
| Eu24.11 | [X]Folie a deux | schizophrenia spectrum |
| Eu20100 | [X]Hebephrenic schizophrenia | schizophrenia spectrum |
| Eu44.14 | [X]Hysterical psychosis | schizophrenia spectrum |
| Eu24.00 | [X]Induced delusional disorder | schizophrenia spectrum |
| Eu24.12 | [X]Induced paranoid disorder | schizophrenia spectrum |
| Eu24.13 | [X]Induced psychotic disorder | schizophrenia spectrum |
| Eu22y12 | [X]Involutional paranoid state | schizophrenia spectrum |
| Eu21.13 | [X]Latent schizophrenia | schizophrenia spectrum |
| Eu21.11 | [X]Latent schizophrenic reaction | schizophrenia spectrum |
| Eu25212 | [X]Mixed schizophrenic and affective psychosis | schizophrenia spectrum |
| Eu26.00 | [X]Nonorganic psychosis in remission | schizophrenia spectrum |
| Eu23y00 | [X]Other acute and transient psychotic disorders | schizophrenia spectrum |
| Eu23300 | [X]Other acute predominantly delusional psychotic disorders | schizophrenia spectrum |
| Ryu5300 | [X]Other hallucinations | schizophrenia spectrum |
| Eu2y.00 | [X]Other nonorganic psychotic disorders | schizophrenia spectrum |
| Eu22y00 | [X]Other persistent delusional disorders | schizophrenia spectrum |
| Eu25y00 | [X]Other schizoaffective disorders | schizophrenia spectrum |
| Eu20y00 | [X]Other schizophrenia | schizophrenia spectrum |
| Eu22015 | [X]Paranoia | schizophrenia spectrum |
| Eu22y13 | [X]Paranoia querulans | schizophrenia spectrum |
| Eu22011 | [X]Paranoid psychosis | schizophrenia spectrum |
| Eu20000 | [X]Paranoid schizophrenia | schizophrenia spectrum |
| Eu22012 | [X]Paranoid state | schizophrenia spectrum |
| Eu22300 | [X]Paranoid state in remission | schizophrenia spectrum |
| Eu22013 | [X]Paraphrenia - late | schizophrenia spectrum |
| Eu20011 | [X]Paraphrenic schizophrenia | schizophrenia spectrum |
| Eu22z00 | [X]Persistent delusional disorder, unspecified | schizophrenia spectrum |
| Eu22.00 | [X]Persistent delusional disorders | schizophrenia spectrum |
| Eu20400 | [X]Post-schizophrenic depression | schizophrenia spectrum |
| Eu21.14 | [X]Prepsychotic schizophrenia | schizophrenia spectrum |
| Eu21.15 | [X]Prodromal schizophrenia | schizophrenia spectrum |
| Eu21.16 | [X]Pseudoneurotic schizophrenia | schizophrenia spectrum |
| Eu21.17 | [X]Pseudopsychopathic schizophrenia | schizophrenia spectrum |
| Eu23312 | [X]Psychogenic paranoid psychosis | schizophrenia spectrum |
| Eu2z.11 | [X]Psychosis NOS | schizophrenia spectrum |
| Eu53111 | [X]Puerperal psychosis NOS | schizophrenia spectrum |
| Eu23z12 | [X]Reactive psychosis | schizophrenia spectrum |
| Eu20500 | [X]Residual schizophrenia | schizophrenia spectrum |
| Eu25100 | [X]Schizoaffective disorder, depressive type | schizophrenia spectrum |
| Eu25000 | [X]Schizoaffective disorder, manic type | schizophrenia spectrum |
| Eu25200 | [X]Schizoaffective disorder, mixed type | schizophrenia spectrum |
| Eu25z00 | [X]Schizoaffective disorder, unspecified | schizophrenia spectrum |
| Eu25.00 | [X]Schizoaffective disorders | schizophrenia spectrum |
| Eu25z11 | [X]Schizoaffective psychosis NOS | schizophrenia spectrum |
| Eu25111 | [X]Schizoaffective psychosis, depressive type | schizophrenia spectrum |
| Eu25011 | [X]Schizoaffective psychosis, manic type | schizophrenia spectrum |
| Eu20.00 | [X]Schizophrenia | schizophrenia spectrum |
| Eu2..00 | [X]Schizophrenia, schizotypal and delusional disorders | schizophrenia spectrum |
| Eu20z00 | [X]Schizophrenia, unspecified | schizophrenia spectrum |
| Eu20212 | [X]Schizophrenic catalepsy | schizophrenia spectrum |
| Eu20213 | [X]Schizophrenic catatonia | schizophrenia spectrum |
| Eu20214 | [X]Schizophrenic flexibilatis cerea | schizophrenia spectrum |
| Eu23214 | [X]Schizophrenic reaction | schizophrenia spectrum |
| Eu20y12 | [X]Schizophreniform disord NOS | schizophrenia spectrum |
| Eu25112 | [X]Schizophreniform psychosis, depressive type | schizophrenia spectrum |
| Eu25012 | [X]Schizophreniform psychosis, manic type | schizophrenia spectrum |
| Eu20y13 | [X]Schizophrenifrm psychos NOS | schizophrenia spectrum |
| Eu21.00 | [X]Schizotypal disorder | schizophrenia spectrum |
| Eu22014 | [X]Sensitiver Beziehungswahn | schizophrenia spectrum |
| Eu20600 | [X]Simple schizophrenia | schizophrenia spectrum |
| Eu84314 | [X]Symbiotic psychosis | schizophrenia spectrum |
| Eu0z.12 | [X]Symptomatic psychosis NOS | schizophrenia spectrum |
| Eu20300 | [X]Undifferentiated schizophrenia | schizophrenia spectrum |
| Eu2z.00 | [X]Unspecified nonorganic psychosis | schizophrenia spectrum |
| E274E00 | 'Short-sleeper' | sleep disorders |
| 1B1B.11 | C/O - insomnia | sleep disorders |
| 1B1D.00 | C/O nightmares | sleep disorders |
| 1B1B.00 | Cannot sleep - insomnia | sleep disorders |
| F270.00 | Cataplexy | sleep disorders |
| F27..00 | Cataplexy and narcolepsy | sleep disorders |
| F27z.00 | Cataplexy or narcolepsy NOS | sleep disorders |
| 1BX0.00 | Delayed onset of sleep | sleep disorders |
| Fy00.00 | Disorders of initiating and maintaining sleep | sleep disorders |
| Fy02.00 | Disorders of the sleep-wake schedule | sleep disorders |
| Z1M1.00 | Disturbing sleep | sleep disorders |
| 1BX1.00 | Excessive sleep | sleep disorders |
| E274311 | Hypersomnia NOS | sleep disorders |
| E274.11 | Hypersomnia of non-organic origin | sleep disorders |
| 1B1B000 | Initial insomnia | sleep disorders |
| E274111 | Insomnia NOS | sleep disorders |
| E274.12 | Insomnia due to nonorganic sleep disorder | sleep disorders |
| E274F00 | Inversion of sleep rhythm | sleep disorders |
| 1B1B200 | Late insomnia | sleep disorders |
| 1BX9.00 | Light sleep | sleep disorders |
| K5A2100 | Menopausal sleeplessness | sleep disorders |
| 1B1B100 | Middle insomnia | sleep disorders |
| F271.00 | Narcolepsy | sleep disorders |
| E274900 | Nightmares | sleep disorders |
| 1B1D.11 | Nightmares - symptom | sleep disorders |
| E274z00 | Non-organic sleep disorder NOS | sleep disorders |
| E274.00 | Non-organic sleep disorders | sleep disorders |
| H5B0.00 | Obstructive sleep apnoea | sleep disorders |
| Fy03.11 | Obstructive sleep apnoea | sleep disorders |
| 9Ngt.00 | On melatonin for sleep disorder | sleep disorders |
| E274y00 | Other non-organic sleep disorder | sleep disorders |
| E274C00 | Other sleep stage or arousal dysfunction | sleep disorders |
| E274400 | Persistent hypersomnia | sleep disorders |
| E274200 | Persistent insomnia | sleep disorders |
| 1B1Q.00 | Poor sleep pattern | sleep disorders |
| 8HTn.00 | Referral to sleep clinic | sleep disorders |
| E274B00 | Repeated rapid eye movement sleep interruptions | sleep disorders |
| E274D00 | Repetitive intrusions of sleep | sleep disorders |
| F13z200 | Restless legs syndrome | sleep disorders |
| E274D11 | Restless sleep | sleep disorders |
| 9Nk0.00 | Seen in sleep clinic | sleep disorders |
| Fy03.00 | Sleep apnoea | sleep disorders |
| H5B..00 | Sleep apnoea | sleep disorders |
| Fy0..00 | Sleep disorders | sleep disorders |
| 8G9B.00 | Sleep hygiene behaviour education | sleep disorders |
| 8Q0..00 | Sleep management | sleep disorders |
| A86..11 | Sleeping sickness | sleep disorders |
| E274700 | Somnambulism - sleep walking | sleep disorders |
| E274300 | Transient hypersomnia | sleep disorders |
| E274100 | Transient insomnia | sleep disorders |
| E274000 | Unspecified non-organic sleep disorder | sleep disorders |
| R005400 | [D]Hypersomnia NOS | sleep disorders |
| R005300 | [D]Hypersomnia with sleep apnoea | sleep disorders |
| R005.11 | [D]Insomnia - symptom | sleep disorders |
| R005200 | [D]Insomnia NOS | sleep disorders |
| R005100 | [D]Insomnia with sleep apnoea | sleep disorders |
| R005311 | [D]Sleep apnoea syndrome | sleep disorders |
| R005000 | [D]Sleep disturbance, unspecified | sleep disorders |
| R005.00 | [D]Sleep disturbances | sleep disorders |
| R005z00 | [D]Sleep dysfunction NOS | sleep disorders |
| R005900 | [D]Sleep dysfunction with arousal disturbance | sleep disorders |
| R005800 | [D]Sleep dysfunction with sleep stage disturbance | sleep disorders |
| R005500 | [D]Sleep rhythm inversion | sleep disorders |
| R005600 | [D]Sleep rhythm irregular | sleep disorders |
| R005.12 | [D]Sleep rhythm problems | sleep disorders |
| R005700 | [D]Sleep-wake rhythm non-24-hour cycle | sleep disorders |
| R005312 | [D]Syndrome sleep apnoea | sleep disorders |
| ZV1B100 | [V]Personal history of unhealthy sleep-wake schedule | sleep disorders |
| Eu51z11 | [X]Emotional sleep disorder NOS | sleep disorders |
| Eu51500 | [X]Nightmares | sleep disorders |
| Eu51200 | [X]Nonorganic disorder of the sleep-wake schedule | sleep disorders |
| Eu51100 | [X]Nonorganic hypersomnia | sleep disorders |
| Eu51000 | [X]Nonorganic insomnia | sleep disorders |
| Eu51z00 | [X]Nonorganic sleep disorder, unspecified | sleep disorders |
| Eu51.00 | [X]Nonorganic sleep disorders | sleep disorders |
| Eu51y00 | [X]Other nonorganic sleep disorders | sleep disorders |
| Fyu5800 | [X]Other sleep disorders | sleep disorders |
| Eu51211 | [X]Psychogenic inversion of circadian rhythm | sleep disorders |
| Eu51213 | [X]Psychogenic inversion of sleep rhythm | sleep disorders |
| Eu51400 | [X]Sleep terrors | sleep disorders |
| Eu51300 | [X]Sleepwalking | sleep disorders |
| J611.00 | Acute alcoholic hepatitis | substance misuse |
| E230.00 | Acute alcoholic intoxication in alcoholism | substance misuse |
| E230z00 | Acute alcoholic intoxication in alcoholism NOS | substance misuse |
| E230300 | Acute alcoholic intoxication in remission, in alcoholism | substance misuse |
| E230000 | Acute alcoholic intoxication, unspecified, in alcoholism | substance misuse |
| 8Hq..00 | Admission to substance misuse detoxification centre | substance misuse |
| 66e0.00 | Alcohol abuse monitoring | substance misuse |
| E011.00 | Alcohol amnestic syndrome | substance misuse |
| E011z00 | Alcohol amnestic syndrome NOS | substance misuse |
| E23..00 | Alcohol dependence syndrome | substance misuse |
| E23z.00 | Alcohol dependence syndrome NOS | substance misuse |
| E230.11 | Alcohol dependence with acute alcoholic intoxication | substance misuse |
| Z191.00 | Alcohol detoxification | substance misuse |
| 8BA8.00 | Alcohol detoxification | substance misuse |
| 66e..00 | Alcohol disorder monitoring | substance misuse |
| 1B1c.00 | Alcohol induced hallucinations | substance misuse |
| 136W.00 | Alcohol misuse | substance misuse |
| E010.00 | Alcohol withdrawal delirium | substance misuse |
| E013.00 | Alcohol withdrawal hallucinosis | substance misuse |
| E01y000 | Alcohol withdrawal syndrome | substance misuse |
| J671000 | Alcohol-induced chronic pancreatitis | substance misuse |
| F25B.00 | Alcohol-induced epilepsy | substance misuse |
| C150500 | Alcohol-induced pseudo-Cushing's syndrome | substance misuse |
| G555.00 | Alcoholic cardiomyopathy | substance misuse |
| J612.00 | Alcoholic cirrhosis of liver | substance misuse |
| E012.11 | Alcoholic dementia NOS | substance misuse |
| F11x011 | Alcoholic encephalopathy | substance misuse |
| J610.00 | Alcoholic fatty liver | substance misuse |
| J612000 | Alcoholic fibrosis and sclerosis of liver | substance misuse |
| J153.00 | Alcoholic gastritis | substance misuse |
| J613000 | Alcoholic hepatic failure | substance misuse |
| J617.00 | Alcoholic hepatitis | substance misuse |
| J613.00 | Alcoholic liver damage unspecified | substance misuse |
| F394100 | Alcoholic myopathy | substance misuse |
| E015.00 | Alcoholic paranoia | substance misuse |
| F375.00 | Alcoholic polyneuropathy | substance misuse |
| E01..00 | Alcoholic psychoses | substance misuse |
| E01z.00 | Alcoholic psychosis NOS | substance misuse |
| 13Y8.00 | Alcoholics anonymous | substance misuse |
| E23..11 | Alcoholism | substance misuse |
| Z4B1.00 | Alcoholism counselling | substance misuse |
| E244z11 | Amfetamine or psychostimulant dependence NOS | substance misuse |
| E244011 | Amfetamine or psychostimulant dependence, unspecified | substance misuse |
| SL97000 | Amfetamine poisoning | substance misuse |
| E244.00 | Amphetamine or other psychostimulant dependence | substance misuse |
| E244z00 | Amphetamine or psychostimulant dependence NOS | substance misuse |
| E244300 | Amphetamine or psychostimulant dependence in remission | substance misuse |
| E244100 | Amphetamine or psychostimulant dependence, continuous | substance misuse |
| E244200 | Amphetamine or psychostimulant dependence, episodic | substance misuse |
| E244000 | Amphetamine or psychostimulant dependence, unspecified | substance misuse |
| SL97011 | Amphetamine poisoning | substance misuse |
| 8G32.00 | Aversion therapy - alcoholism | substance misuse |
| E241.13 | Benzodiazepine dependence | substance misuse |
| 136R.00 | Binge drinker | substance misuse |
| E243300 | Cannabis dependence in remission | substance misuse |
| E243100 | Cannabis dependence, continuous | substance misuse |
| E243200 | Cannabis dependence, episodic | substance misuse |
| E243000 | Cannabis dependence, unspecified | substance misuse |
| E243z00 | Cannabis drug dependence NOS | substance misuse |
| E243.00 | Cannabis type drug dependence | substance misuse |
| F144000 | Cerebellar ataxia due to alcoholism | substance misuse |
| F11x000 | Cerebral degeneration due to alcoholism | substance misuse |
| 1V0A.00 | Chases the dragon | substance misuse |
| E012000 | Chronic alcoholic brain syndrome | substance misuse |
| J617000 | Chronic alcoholic hepatitis | substance misuse |
| E231.00 | Chronic alcoholism | substance misuse |
| E231z00 | Chronic alcoholism NOS | substance misuse |
| E231300 | Chronic alcoholism in remission | substance misuse |
| E242100 | Cocaine dependence, continuous | substance misuse |
| E242200 | Cocaine dependence, episodic | substance misuse |
| E242000 | Cocaine dependence, unspecified | substance misuse |
| E242z00 | Cocaine drug dependence NOS | substance misuse |
| SL85000 | Cocaine poisoning | substance misuse |
| E242.00 | Cocaine type drug dependence | substance misuse |
| E249z00 | Combined drug dependence, excluding opioid, NOS | substance misuse |
| E249100 | Combined drug dependence, excluding opioid, continuous | substance misuse |
| E249200 | Combined drug dependence, excluding opioid, episodic | substance misuse |
| E249000 | Combined drug dependence, excluding opioid, unspecified | substance misuse |
| E249.00 | Combined drug dependence, excluding opioids | substance misuse |
| E248.00 | Combined opioid with other drug dependence | substance misuse |
| E248z00 | Combined opioid with other drug dependence NOS | substance misuse |
| E248100 | Combined opioid with other drug dependence, continuous | substance misuse |
| E248200 | Combined opioid with other drug dependence, episodic | substance misuse |
| E248000 | Combined opioid with other drug dependence, unspecified | substance misuse |
| E230100 | Continuous acute alcoholic intoxication in alcoholism | substance misuse |
| E231100 | Continuous chronic alcoholism | substance misuse |
| E240100 | Continuous opioid dependence | substance misuse |
| 13c7.00 | Current drug user | substance misuse |
| 9N4i.00 | DNA - Did not attend substance misuse clinic | substance misuse |
| 7P22100 | Delivery of rehabilitation for alcohol addiction | substance misuse |
| 7P22000 | Delivery of rehabilitation for drug addiction | substance misuse |
| Z192.00 | Dependent drug detoxification | substance misuse |
| 1TF..00 | Does not use heroin on top of substitution therapy | substance misuse |
| 8AA..00 | Drug abuse monitoring | substance misuse |
| 1V0C.00 | Drug addict | substance misuse |
| 9G2..11 | Drug addict notific admin | substance misuse |
| 9G21.00 | Drug addict notific to CMO | substance misuse |
| 9G23.00 | Drug addict re-notif to CMO | substance misuse |
| 9G22.00 | Drug addict re-notific due | substance misuse |
| 9G24.00 | Drug addict-notify local SMR22 | substance misuse |
| E24..11 | Drug addiction | substance misuse |
| 8B2R.00 | Drug addiction detoxification therapy - buprenorphine | substance misuse |
| 8B2N.00 | Drug addiction detoxification therapy - methadone | substance misuse |
| 8B2Q.00 | Drug addiction maintenance therapy - buprenorphine | substance misuse |
| 8B2P.00 | Drug addiction maintenance therapy - methadone | substance misuse |
| 9G2Z.00 | Drug addiction notif NOS | substance misuse |
| 9G2..00 | Drug addiction notification | substance misuse |
| 8B23.00 | Drug addiction therapy | substance misuse |
| 8B23.11 | Drug addictn therap-methadone | substance misuse |
| E24..00 | Drug dependence | substance misuse |
| E24z.00 | Drug dependence NOS | substance misuse |
| L183100 | Drug dependence during pregnancy - baby delivered | substance misuse |
| L183300 | Drug dependence during pregnancy - baby not yet delivered | substance misuse |
| L183z00 | Drug dependence during pregnancy/childbirth/puerperium NOS | substance misuse |
| 8BAX.00 | Drug dependence home detoxification | substance misuse |
| 8I2N.00 | Drug dependence home detoxification contraindicated | substance misuse |
| L183.00 | Drug dependence in pregnancy, childbirth and the puerperium | substance misuse |
| 8BAW.00 | Drug dependence self detoxification | substance misuse |
| 8B23.13 | Drug dependence therapy | substance misuse |
| 9k50.00 | Drug misuse - enhanced service completed | substance misuse |
| 9k5..00 | Drug misuse - enhanced services administration | substance misuse |
| 9kS..00 | Drug misuse assessment declined - enhanced services administ | substance misuse |
| 9s...00 | Drug misuse clinic administration | substance misuse |
| 9k52.11 | Drug misuse treatment in primary care | substance misuse |
| 9k52.00 | Drug misuse treatment primary care - enhanced services admin | substance misuse |
| E02..00 | Drug psychoses | substance misuse |
| E02z.00 | Drug psychosis NOS | substance misuse |
| 13c..00 | Drug user | substance misuse |
| E02y400 | Drug-induced personality disorder | substance misuse |
| 1V6..00 | Drug-related offending behaviour | substance misuse |
| SL97200 | Ecstasy poisoning | substance misuse |
| E24A.00 | Ecstasy type drug dependence | substance misuse |
| E230200 | Episodic acute alcoholic intoxication in alcoholism | substance misuse |
| E231200 | Episodic chronic alcoholism | substance misuse |
| E240200 | Episodic opioid dependence | substance misuse |
| 1283.00 | FH: Drug dependency | substance misuse |
| 146C.00 | Failed heroin detoxification | substance misuse |
| 9HC1.00 | Follow up substance misuse assessment | substance misuse |
| E246.00 | Glue sniffing dependence | substance misuse |
| E246z00 | Glue sniffing dependence NOS | substance misuse |
| E246100 | Glue sniffing dependence, continuous | substance misuse |
| E246200 | Glue sniffing dependence, episodic | substance misuse |
| E246000 | Glue sniffing dependence, unspecified | substance misuse |
| 1T00.00 | H/O daily heroin misuse | substance misuse |
| 1TD0.00 | H/O daily opiate misuse | substance misuse |
| 1T90.00 | H/O daily solvent misuse | substance misuse |
| 1T0..00 | H/O heroin misuse | substance misuse |
| 1T42.00 | H/O infrequent amphetamine misuse | substance misuse |
| 1T52.00 | H/O infrequent cocaine misuse | substance misuse |
| 1T62.00 | H/O infrequent crack cocaine misuse | substance misuse |
| 1T03.00 | H/O infrequent heroin misuse | substance misuse |
| 1TD2.00 | H/O infrequent opiate misuse | substance misuse |
| 1T92.00 | H/O infrequent solvent misuse | substance misuse |
| 1TG..00 | H/O novel psychoactive substance misuse | substance misuse |
| 1TD..00 | H/O opiate misuse | substance misuse |
| 1T9..00 | H/O solvent misuse | substance misuse |
| 1T41.00 | H/O weekly amphetamine misuse | substance misuse |
| 1T51.00 | H/O weekly cocaine misuse | substance misuse |
| 1T61.00 | H/O weekly crack cocaine misuse | substance misuse |
| 1T01.00 | H/O weekly heroin misuse | substance misuse |
| 1TD1.00 | H/O weekly opiate misuse | substance misuse |
| 1T91.00 | H/O weekly solvent misuse | substance misuse |
| 1462.00 | H/O: alcoholism | substance misuse |
| 146F.00 | H/O: drug abuse | substance misuse |
| 1463.00 | H/O: drug dependency | substance misuse |
| E245.00 | Hallucinogen dependence | substance misuse |
| E245z00 | Hallucinogen dependence NOS | substance misuse |
| E245100 | Hallucinogen dependence, continuous | substance misuse |
| E245200 | Hallucinogen dependence, episodic | substance misuse |
| E245000 | Hallucinogen dependence, unspecified | substance misuse |
| 136T.00 | Harmful alcohol use | substance misuse |
| E243.11 | Hashish dependence | substance misuse |
| 136S.00 | Hazardous alcohol use | substance misuse |
| 1V0E.00 | Health problem secondary to drug misuse | substance misuse |
| E240.11 | Heroin dependence | substance misuse |
| Z1Q6214 | Heroin maintenance | substance misuse |
| 1V65.00 | Heroin misuse | substance misuse |
| SL50100 | Heroin poisoning | substance misuse |
| 1T...00 | History of substance misuse | substance misuse |
| E241200 | Hypnotic or anxiolytic dependence, episodic | substance misuse |
| 9NX2.00 | In-house substance misuse treatment | substance misuse |
| 9HC0.00 | Initial substance misuse assessment | substance misuse |
| 13c0.00 | Injecting drug user | substance misuse |
| 1V30.00 | Injects drugs subcutaneously | substance misuse |
| E250.14 | Intoxication - alcohol | substance misuse |
| 13c1.00 | Intravenous drug user | substance misuse |
| E011000 | Korsakov's alcoholic psychosis | substance misuse |
| E011100 | Korsakov's alcoholic psychosis with peripheral neuritis | substance misuse |
| E245.11 | LSD dependence | substance misuse |
| E253.12 | LSD reaction | substance misuse |
| 1V01.00 | Long-term drug misuser | substance misuse |
| E245.12 | Lysergic acid diethylamide dependence | substance misuse |
| E243.13 | Marihuana dependence | substance misuse |
| E240.12 | Methadone dependence | substance misuse |
| Z1Q6200 | Methadone maintenance | substance misuse |
| Z1Q6212 | Methadone therapy | substance misuse |
| E259400 | Misuse of prescription only drugs | substance misuse |
| 13cB.00 | Misuses drugs orally | substance misuse |
| SL50500 | Morphine poisoning | substance misuse |
| E25..00 | Nondependent abuse of drugs | substance misuse |
| E250.00 | Nondependent alcohol abuse | substance misuse |
| E250z00 | Nondependent alcohol abuse NOS | substance misuse |
| E250300 | Nondependent alcohol abuse in remission | substance misuse |
| E250100 | Nondependent alcohol abuse, continuous | substance misuse |
| E250200 | Nondependent alcohol abuse, episodic | substance misuse |
| E250000 | Nondependent alcohol abuse, unspecified | substance misuse |
| E257.00 | Nondependent amphetamine or other psychostimulant abuse | substance misuse |
| E257z00 | Nondependent amphetamine or psychostimulant abuse NOS | substance misuse |
| E257200 | Nondependent amphetamine or psychostimulant abuse, episodic | substance misuse |
| E257300 | Nondependent amphetamine/psychostimulant abuse in remission | substance misuse |
| E257100 | Nondependent amphetamine/psychostimulant abuse, continuous | substance misuse |
| E257000 | Nondependent amphetamine/psychostimulant abuse, unspecified | substance misuse |
| E258.00 | Nondependent antidepressant type drug abuse | substance misuse |
| E258z00 | Nondependent antidepressant type drug abuse NOS | substance misuse |
| E252.00 | Nondependent cannabis abuse | substance misuse |
| E252z00 | Nondependent cannabis abuse NOS | substance misuse |
| E252100 | Nondependent cannabis abuse, continuous | substance misuse |
| E252200 | Nondependent cannabis abuse, episodic | substance misuse |
| E252000 | Nondependent cannabis abuse, unspecified | substance misuse |
| E256.00 | Nondependent cocaine abuse | substance misuse |
| E256z00 | Nondependent cocaine abuse NOS | substance misuse |
| E256300 | Nondependent cocaine abuse in remission | substance misuse |
| E256100 | Nondependent cocaine abuse, continuous | substance misuse |
| E256200 | Nondependent cocaine abuse, episodic | substance misuse |
| E256000 | Nondependent cocaine abuse, unspecified | substance misuse |
| E253.00 | Nondependent hallucinogen abuse | substance misuse |
| E253z00 | Nondependent hallucinogen abuse NOS | substance misuse |
| E253300 | Nondependent hallucinogen abuse in remission | substance misuse |
| E253100 | Nondependent hallucinogen abuse, continuous | substance misuse |
| E253200 | Nondependent hallucinogen abuse, episodic | substance misuse |
| E253000 | Nondependent hallucinogen abuse, unspecified | substance misuse |
| E259.00 | Nondependent mixed drug abuse | substance misuse |
| E259z00 | Nondependent mixed drug abuse NOS | substance misuse |
| E259300 | Nondependent mixed drug abuse in remission | substance misuse |
| E259100 | Nondependent mixed drug abuse, continuous | substance misuse |
| E259200 | Nondependent mixed drug abuse, episodic | substance misuse |
| E259000 | Nondependent mixed drug abuse, unspecified | substance misuse |
| E255.00 | Nondependent opioid abuse | substance misuse |
| E255z00 | Nondependent opioid abuse NOS | substance misuse |
| E255300 | Nondependent opioid abuse in remission | substance misuse |
| E255100 | Nondependent opioid abuse, continuous | substance misuse |
| E255200 | Nondependent opioid abuse, episodic | substance misuse |
| E255000 | Nondependent opioid abuse, unspecified | substance misuse |
| E25y.00 | Nondependent other drug abuse | substance misuse |
| E25yz00 | Nondependent other drug abuse NOS | substance misuse |
| E25y300 | Nondependent other drug abuse in remission | substance misuse |
| E25y100 | Nondependent other drug abuse, continuous | substance misuse |
| E25y200 | Nondependent other drug abuse, episodic | substance misuse |
| E25y000 | Nondependent other drug abuse, unspecified | substance misuse |
| 1V07.00 | Notified addict | substance misuse |
| 13cM000 | Novel psychoactive substance misuse | substance misuse |
| 2577.11 | O/E - alcoholic breath | substance misuse |
| G852300 | Oesophageal varices in alcoholic cirrhosis of the liver | substance misuse |
| 9HCC.00 | On substance misuse programme | substance misuse |
| SL50.00 | Opiate and narcotic poisoning | substance misuse |
| SL50.12 | Opiate poisoning | substance misuse |
| 8B2S.00 | Opioid agonist substitution therapy | substance misuse |
| 8B2T.00 | Opioid antagonist therapy | substance misuse |
| E240z00 | Opioid drug dependence NOS | substance misuse |
| E240.00 | Opioid type drug dependence | substance misuse |
| E240.14 | Opium dependence | substance misuse |
| E012.00 | Other alcoholic dementia | substance misuse |
| E01y.00 | Other alcoholic psychosis | substance misuse |
| E02y.00 | Other drug psychoses | substance misuse |
| E02yz00 | Other drug psychoses NOS | substance misuse |
| E247.00 | Other specified drug dependence | substance misuse |
| E247z00 | Other specified drug dependence NOS | substance misuse |
| E247100 | Other specified drug dependence, continuous | substance misuse |
| E247200 | Other specified drug dependence, episodic | substance misuse |
| E247000 | Other specified drug dependence, unspecified | substance misuse |
| E014.00 | Pathological alcohol intoxication | substance misuse |
| 13cH.00 | Persistent substance misuse | substance misuse |
| 9k53.11 | Pharmacy attended for drug misuse | substance misuse |
| 9k53.00 | Pharmacy attended for drug misuse - enhanced services admin | substance misuse |
| 1V02.00 | Poly-drug misuser | substance misuse |
| L183.11 | Pregnancy and drug dependence | substance misuse |
| 13cF.00 | Preoccupied with substance misuse | substance misuse |
| 1T43.00 | Previous history of amphetamine misuse | substance misuse |
| 1T53.00 | Previous history of cocaine misuse | substance misuse |
| 1T63.00 | Previous history of crack cocaine misuse | substance misuse |
| 1T02.00 | Previous history of heroin misuse | substance misuse |
| 1TD3.00 | Previous history of opiate misuse | substance misuse |
| 1T93.00 | Previous history of solvent misuse | substance misuse |
| 13cE.00 | Prolonged high dose use of cannabis | substance misuse |
| E257.11 | Psychostimulant abuse | substance misuse |
| E244.11 | Psychostimulant dependence | substance misuse |
| 8W2..00 | Refer to MH services deferred until alcohol misuse resolved | substance misuse |
| 8H7p.00 | Referral to community alcohol team | substance misuse |
| 8IEA.00 | Referral to community alcohol team declined | substance misuse |
| 8HHe.00 | Referral to community drug and alcohol team | substance misuse |
| 8HHL.00 | Referral to community drug dependency team | substance misuse |
| 8H7x.00 | Referral to drug abuse counsellor | substance misuse |
| 8Hl5.00 | Referral to drugs therapist | substance misuse |
| 8HkG.00 | Referral to specialist alcohol treatment service | substance misuse |
| 8HkF.00 | Referral to substance misuse service | substance misuse |
| 8BE0.00 | Reinduction to methadone maintenance therapy | substance misuse |
| 9N1yJ00 | Seen in drug misuse clinic | substance misuse |
| 9No5.00 | Seen in substance misuse clinic | substance misuse |
| 8Hh1.00 | Self referral to substance misuse service | substance misuse |
| 9k51.11 | Shared care drug misuse treatment | substance misuse |
| 9k51.00 | Shared care drug misuse treatment - enhanced services admin | substance misuse |
| 1V35.00 | Shares drug equipment | substance misuse |
| 1V08.00 | Smokes drugs in cigarette form | substance misuse |
| 1V09.00 | Smokes drugs through a pipe | substance misuse |
| 1V0B.00 | Sniffs drugs | substance misuse |
| E257.12 | Stimulant abuse | substance misuse |
| E244.12 | Stimulant dependence | substance misuse |
| 13cM.00 | Substance misuse | substance misuse |
| 9HC2.00 | Substance misuse clinical management plan agreed | substance misuse |
| 9HC3.00 | Substance misuse clinical management plan reviewed | substance misuse |
| 8BAc.00 | Substance misuse management stopped - self withdrawal | substance misuse |
| 9HC..00 | Substance misuse monitoring | substance misuse |
| 9HCA.00 | Substance misuse monitoring 6 month review | substance misuse |
| 677T.00 | Substance misuse structured counselling | substance misuse |
| 9HC6.00 | Substance misuse treatment declined | substance misuse |
| 9HC5.00 | Substance misuse treatment programme completed | substance misuse |
| 9HC4.00 | Substance misuse treatment withdrawn | substance misuse |
| 9HC9.00 | Substnce misse treatmnt pgme del other healththcare provider | substance misuse |
| 1369.00 | Suspect alcohol abuse - denied | substance misuse |
| 1J11.00 | Suspected abuse hard drugs | substance misuse |
| 1J10.00 | Suspected abuse soft drugs | substance misuse |
| 1J1..00 | Suspected drug abuse | substance misuse |
| E231000 | Unspecified chronic alcoholism | substance misuse |
| E240000 | Unspecified opioid dependence | substance misuse |
| 1TE..00 | Uses heroin on top of substitution therapy | substance misuse |
| E011200 | Wernicke-Korsakov syndrome | substance misuse |
| R10B200 | [D]Finding of psychotropic drug in blood | substance misuse |
| ZV6D600 | [V]Alcohol abuse counselling and surveillance | substance misuse |
| ZV6D700 | [V]Drug abuse counselling and surveillance | substance misuse |
| ZV11300 | [V]Personal history of alcoholism | substance misuse |
| ZV11500 | [V]Personal history of drug abuse by injection | substance misuse |
| ZV11400 | [V]Personal history of psychoactive substance abuse | substance misuse |
| U1A5.11 | [X]Accidental poisoning with heroin | substance misuse |
| Eu10211 | [X]Alcohol addiction | substance misuse |
| Eu10800 | [X]Alcohol withdrawal-induced seizure | substance misuse |
| Eu10711 | [X]Alcoholic dementia NOS | substance misuse |
| Eu10511 | [X]Alcoholic hallucinosis | substance misuse |
| Eu10512 | [X]Alcoholic jealousy | substance misuse |
| Eu10513 | [X]Alcoholic paranoia | substance misuse |
| Eu10514 | [X]Alcoholic psychosis NOS | substance misuse |
| Eu10712 | [X]Chronic alcoholic brain syndrome | substance misuse |
| Eu10212 | [X]Chronic alcoholism | substance misuse |
| Eu11311 | [X]Cold turkey, opiate withdrawal | substance misuse |
| Eu10411 | [X]Delirium tremens, alcohol induced | substance misuse |
| Eu12211 | [X]Drug addiction - cannabis | substance misuse |
| Eu14211 | [X]Drug addiction - cocaine | substance misuse |
| Eu16211 | [X]Drug addiction - hallucinogen | substance misuse |
| Eu11211 | [X]Drug addiction - opioids | substance misuse |
| Eu18211 | [X]Drug addiction - solvent | substance misuse |
| Eu19211 | [X]Drug addiction NOS | substance misuse |
| Eu13211 | [X]Drug addiction- sedative / hypnotics | substance misuse |
| Eu15211 | [X]Drug addiction-other stimul | substance misuse |
| U81..00 | [X]Evid of alcohol involv determind by level of intoxication | substance misuse |
| Eu11212 | [X]Heroin addiction | substance misuse |
| Eu10611 | [X]Korsakov's psychosis, alcohol induced | substance misuse |
| Eu14700 | [X]Men & beh dis due cocaine: resid & late-onset psychot dis | substance misuse |
| Eu11700 | [X]Men & beh dis due opioids: resid & late-onset psychot dis | substance misuse |
| Eu18400 | [X]Men & beh dis vol solvents: withdrawal state wth delirium | substance misuse |
| Eu10700 | [X]Men & behav dis due alcoh: resid & late-onset psychot dis | substance misuse |
| Eu10400 | [X]Men & behav dis due alcohl: withdrawl state with delirium | substance misuse |
| Eu11400 | [X]Men & behav dis due opioid: withdrawl state with delirium | substance misuse |
| Eu10y00 | [X]Men & behav dis due to use alcohol: oth men & behav dis | substance misuse |
| Eu11y00 | [X]Men & behav dis due to use opioids: oth men & behav dis | substance misuse |
| Eu19.00 | [X]Men & behav disorder multiple drug use/psychoactive subst | substance misuse |
| Eu19y00 | [X]Men/beh dis mlt drg use/oth psy sbs: oth men & behav dis | substance misuse |
| Eu19700 | [X]Men/beh dis mlt drg use/oth subs: resid/late psychot dis | substance misuse |
| Eu12y00 | [X]Men/behav dis due to use cannabinoids: oth men/behav disd | substance misuse |
| Eu10z00 | [X]Ment & behav dis due use alcohol: unsp ment & behav dis | substance misuse |
| Eu11z00 | [X]Ment & behav dis due use opioids: unsp ment & behav dis | substance misuse |
| Eu1Az00 | [X]Ment behav dis due crack cocaine: unsp ment and behav dis | substance misuse |
| Eu19z00 | [X]Ment/beh dis multi drug use/oth psy sbs unsp mnt/beh dis | substance misuse |
| Eu15z00 | [X]Ment/beh dis oth stims inc caffeine: unsp ment/behav disd | substance misuse |
| Eu12z00 | [X]Ment/behav dis due use cannabinoids: unsp ment/behav disd | substance misuse |
| Eu16z00 | [X]Ment/behav dis due use hallucinogens: unsp ment/behav dis | substance misuse |
| Eu18z00 | [X]Ment/behav dis due use vol solvents: unsp ment/behav dis | substance misuse |
| Eu19500 | [X]Ment/behav dis mlti drug use/oth psyc sbs: psychotc dis | substance misuse |
| Eu12500 | [X]Mental & behav dis due to cannabinoids: psychotic disordr | substance misuse |
| Eu16500 | [X]Mental & behav dis due to hallucinogens: psychotic disord | substance misuse |
| Eu13500 | [X]Mental & behav dis due to seds/hypntcs: psychotic disordr | substance misuse |
| Eu10000 | [X]Mental & behav dis due to use alcohol: acute intoxication | substance misuse |
| Eu10500 | [X]Mental & behav dis due to use alcohol: psychotic disorder | substance misuse |
| Eu14500 | [X]Mental & behav dis due to use cocaine: psychotic disorder | substance misuse |
| Eu11500 | [X]Mental & behav dis due to use opioids: psychotic disorder | substance misuse |
| Eu18500 | [X]Mental & behav dis due to vol solvents: psychotic disordr | substance misuse |
| Eu15.00 | [X]Mental & behav disorder due other stimulants inc caffein | substance misuse |
| Eu18.00 | [X]Mental & behav disorders due to use of volatile solvents | substance misuse |
| Eu12300 | [X]Mental and behav dis due cannabinoids: withdrawal state | substance misuse |
| Eu16300 | [X]Mental and behav dis due hallucinogens: withdrawal state | substance misuse |
| Eu13300 | [X]Mental and behav dis due seds/hypntcs: withdrawal state | substance misuse |
| Eu12200 | [X]Mental and behav dis due to cannabinoids: dependence synd | substance misuse |
| Eu16200 | [X]Mental and behav dis due to hallucinogens: dependence syn | substance misuse |
| Eu13200 | [X]Mental and behav dis due to seds/hypntcs: dependence synd | substance misuse |
| Eu10600 | [X]Mental and behav dis due to use alcohol: amnesic syndrome | substance misuse |
| Eu10200 | [X]Mental and behav dis due to use alcohol: dependence syndr | substance misuse |
| Eu10300 | [X]Mental and behav dis due to use alcohol: withdrawal state | substance misuse |
| Eu12600 | [X]Mental and behav dis due to use cannabinoids: amnesic syn | substance misuse |
| Eu12100 | [X]Mental and behav dis due to use cannabinoids: harmful use | substance misuse |
| Eu14200 | [X]Mental and behav dis due to use cocaine: dependence syndr | substance misuse |
| Eu14300 | [X]Mental and behav dis due to use cocaine: withdrawal state | substance misuse |
| Eu16100 | [X]Mental and behav dis due to use hallucinogens: harmfl use | substance misuse |
| Eu10100 | [X]Mental and behav dis due to use of alcohol: harmful use | substance misuse |
| Eu14100 | [X]Mental and behav dis due to use of cocaine: harmful use | substance misuse |
| Eu11100 | [X]Mental and behav dis due to use of opioids: harmful use | substance misuse |
| Eu11600 | [X]Mental and behav dis due to use opioids: amnesic syndrome | substance misuse |
| Eu11200 | [X]Mental and behav dis due to use opioids: dependence syndr | substance misuse |
| Eu11300 | [X]Mental and behav dis due to use opioids: withdrawal state | substance misuse |
| Eu13100 | [X]Mental and behav dis due to use seds/hypntcs: harmful use | substance misuse |
| Eu18200 | [X]Mental and behav dis due to vol solvents: dependence synd | substance misuse |
| Eu18100 | [X]Mental and behav dis due volatile solvents: harmful use | substance misuse |
| Eu19100 | [X]Mental and behav dis mlti drg/oth psychoa sbs: harmfl use | substance misuse |
| Eu19200 | [X]Mental and behav dis mlti/oth psych sbs: dependence syndr | substance misuse |
| Eu19300 | [X]Mental and behav dis mlti/oth psychoa sbs: withdrwl state | substance misuse |
| Eu15200 | [X]Mental and behav dis oth stim inc caffein: dependnce synd | substance misuse |
| Eu13.00 | [X]Mental and behavioural dis due use sedatives/hypnotics | substance misuse |
| Eu1..00 | [X]Mental and behavioural disorders due to psychoactive subs | substance misuse |
| Eu12.00 | [X]Mental and behavioural disorders due to use cannabinoids | substance misuse |
| Eu16.00 | [X]Mental and behavioural disorders due to use hallucinogens | substance misuse |
| Eu10.00 | [X]Mental and behavioural disorders due to use of alcohol | substance misuse |
| Eu14.00 | [X]Mental and behavioural disorders due to use of cocaine | substance misuse |
| Eu11.00 | [X]Mental and behavioural disorders due to use of opioids | substance misuse |
| Eu1A.00 | [X]Mental and behavioural disorders due use of crack cocaine | substance misuse |
| Eu1A500 | [X]Mental behav disord due crack cocaine: psychotic disorder | substance misuse |
| Eu1A300 | [X]Mental behav disord due crack cocaine: withdrawal state | substance misuse |
| Eu1A200 | [X]Mental behav disorders due use crack cocaine: depend synd | substance misuse |
| Eu1A100 | [X]Mental behav disorders due use crack cocaine: harmful use | substance misuse |
| Eu19600 | [X]Mental/behav dis multi drg use/oth psy sbs: amnesic syndr | substance misuse |
| Eu12700 | [X]Mnt/bh dis due cannabinds: resid & late-onset psychot dis | substance misuse |
| Eu16700 | [X]Mnt/bh dis due hallucngns: resid & late-onset psychot dis | substance misuse |
| Eu19400 | [X]Mnt/bh dis mlti drg use/oth psy sbs: wthdr state + dlrium | substance misuse |
| Eu16711 | [X]Post hallucinogen perception disorder | substance misuse |

Table S2: Medical code IDs and terms used for identifying psychiatric illnesses in CPRD Aurum.

| Medical Code ID | Term | Category |
| --- | --- | --- |
| 481850015 | Agitated | affective disorders |
| 474171000006112 | Agitated depression | affective disorders |
| 138421012 | Agitated depression | affective disorders |
| 1771531000006110 | Antenatal depression | affective disorders |
| 294655014 | Arteriosclerotic dementia with depression | affective disorders |
| 294894013 | Atypical depressive disorder | affective disorders |
| 294893019 | Atypical manic disorder | affective disorders |
| 6357571000006119 | Bipolar 1 disorder | affective disorders |
| 3854401000006113 | Bipolar 2 disorder | affective disorders |
| 3301331000006119 | Bipolar I disorder, most recent episode depressed, in partial remission | affective disorders |
| 3006671000006118 | Bipolar I disorder, most recent episode hypomanic | affective disorders |
| 3528681000006111 | Bipolar I disorder, most recent episode manic, in partial remission | affective disorders |
| 2761681000006119 | Bipolar I disorder, most recent episode mixed | affective disorders |
| 513691000006116 | Bipolar affect disord, currently manic, severe, no psychosis | affective disorders |
| 513701000006116 | Bipolar affect disord, currently manic,severe with psychosis | affective disorders |
| 513711000006118 | Bipolar affect disord, now depressed, part/unspec remission | affective disorders |
| 513721000006114 | Bipolar affect disord, now depressed, severe with psychosis | affective disorders |
| 513731000006112 | Bipolar affect disord, now depressed, severe, no psychosis | affective disorders |
| 513741000006119 | Bipolar affect disord,currently manic, part/unspec remission | affective disorders |
| 1975941000006115 | Bipolar affect disorder cur epi mild or moderate depressn, with somatic syndrome | affective disorders |
| 1975931000006113 | Bipolar affect disorder cur epi mild or moderate depressn, without somatic syndrome | affective disorders |
| 492761000000114 | Bipolar affective disorder resolved | affective disorders |
| 4767311000006117 | Bipolar affective disorder, current episode mixed | affective disorders |
| 513751000006117 | Bipolar affective disorder, currently depressed | affective disorders |
| 294868016 | Bipolar affective disorder, currently depressed, NOS | affective disorders |
| 294862015 | Bipolar affective disorder, currently depressed, mild | affective disorders |
| 294863013 | Bipolar affective disorder, currently depressed, moderate | affective disorders |
| 294861010 | Bipolar affective disorder, currently depressed, unspecified | affective disorders |
| 513801000006112 | Bipolar affective disorder, currently manic | affective disorders |
| 294858014 | Bipolar affective disorder, currently manic, NOS | affective disorders |
| 513811000006110 | Bipolar affective disorder, currently manic, full remission | affective disorders |
| 294849019 | Bipolar affective disorder, currently manic, mild | affective disorders |
| 294850019 | Bipolar affective disorder, currently manic, moderate | affective disorders |
| 294848010 | Bipolar affective disorder, currently manic, unspecified | affective disorders |
| 513861000006113 | Bipolar affective disorder, now depressed, in full remission | affective disorders |
| 3174071000006116 | Bipolar disorder, full remission | affective disorders |
| 3886291000006119 | Bipolar disorder, in remission | affective disorders |
| 2590511000006116 | Bipolar disorder, partial remission | affective disorders |
| 513871000006118 | Bipolar psychoses | affective disorders |
| 525921000006119 | Brief depressive reaction | affective disorders |
| 295494011 | Brief depressive reaction NOS | affective disorders |
| 407062014 | C/O - feeling depressed | affective disorders |
| 407066012 | C/O - feeling unhappy | affective disorders |
| 454064011 | C/O weepiness | affective disorders |
| 295537016 | Chronic depression | affective disorders |
| 6550791000006111 | Counseling for postnatal depression | affective disorders |
| 6550801000006112 | Counselling for postnatal depression | affective disorders |
| 3071801000006112 | Depressed | affective disorders |
| 2164006016 | Depressed | affective disorders |
| 2164005017 | Depressed mood | affective disorders |
| 882671000006112 | Depression | affective disorders |
| 226411000000110 | Depression - enhanced services administration | affective disorders |
| 882681000006110 | Depression NOS | affective disorders |
| 2534096013 | Depression annual review | affective disorders |
| 8245201000006115 | Depression care management | affective disorders |
| 1823881000006110 | Depression confirmed | affective disorders |
| 2534091015 | Depression interim review | affective disorders |
| 6649631000006118 | Depression management program | affective disorders |
| 1773563015 | Depression management programme | affective disorders |
| 2534092010 | Depression medication review | affective disorders |
| 294621000000118 | Depression resolved | affective disorders |
| 295535012 | Depressive disorder NEC | affective disorders |
| 12727931000006110 | Depressive disorder NEC | affective disorders |
| 8460861000006115 | Depressive personality disorder | affective disorders |
| 613791000006115 | Depressive psychoses | affective disorders |
| 1494612017 | Depressive symptoms | affective disorders |
| 4763361000006113 | Drug-induced depression | affective disorders |
| 135278017 | Elevated mood | affective disorders |
| 441826016 | Endogenous depression | affective disorders |
| 410861011 | Endogenous depression - recurrent | affective disorders |
| 642461000006116 | Endogenous depression first episode | affective disorders |
| 346972018 | Endogenous depression first episode | affective disorders |
| 251629019 | H/O: depression | affective disorders |
| 1780205015 | H/O: manic depressive disorder | affective disorders |
| 4539961000006115 | Has had depression | affective disorders |
| 4539971000006110 | History of depression | affective disorders |
| 789221000006116 | Hypomanic psychoses | affective disorders |
| 411890010 | Lithium monitoring | affective disorders |
| 369972011 | Loss of capacity for enjoyment | affective disorders |
| 369974012 | Loss of hope for the future | affective disorders |
| 2164009011 | Low mood | affective disorders |
| 3094941000006114 | Major depression, single episode | affective disorders |
| 2809821000006115 | Major depression, single episode, in complete remission | affective disorders |
| 2809831000006117 | Major depression, single episode, in full remission | affective disorders |
| 3650771000006113 | Major depression, single episode, in partial remission | affective disorders |
| 3094951000006111 | Major depressive disorder, single episode | affective disorders |
| 882321000006119 | Mania/hypomania | affective disorders |
| 401765010 | Manic disorder, single episode | affective disorders |
| 294809010 | Manic disorder, single episode NOS | affective disorders |
| 2157096015 | Manic mood | affective disorders |
| 223601000000119 | Manic psychoses | affective disorders |
| 294860011 | Manic-depressive - now depressed | affective disorders |
| 294847017 | Manic-depressive - now manic | affective disorders |
| 346973011 | Masked depression | affective disorders |
| 2620391000000111 | Maternal postnatal depression | affective disorders |
| 306153015 | Mental disorder during pregnancy - baby not yet delivered | affective disorders |
| 705571000006116 | Mental disorder in puerperium - baby previously delivered | affective disorders |
| 306152013 | Mental disorder in the puerperium - baby delivered | affective disorders |
| 705591000006115 | Mental disorders in pregnancy, childbirth and the puerperium | affective disorders |
| 6000691000006114 | Mild depression | affective disorders |
| 882811000006119 | Mild depression | affective disorders |
| 3789581000006112 | Mild major depression, single episode | affective disorders |
| 294869012 | Mixed bipolar affective disorder | affective disorders |
| 294877011 | Mixed bipolar affective disorder, NOS | affective disorders |
| 294876019 | Mixed bipolar affective disorder, in full remission | affective disorders |
| 8235161000006110 | Mixed bipolar affective disorder, in partial remission | affective disorders |
| 294871012 | Mixed bipolar affective disorder, mild | affective disorders |
| 294872017 | Mixed bipolar affective disorder, moderate | affective disorders |
| 701051000006118 | Mixed bipolar affective disorder, partial/unspec remission | affective disorders |
| 294874016 | Mixed bipolar affective disorder, severe, with psychosis | affective disorders |
| 701071000006111 | Mixed bipolar affective disorder, severe, without psychosis | affective disorders |
| 294870013 | Mixed bipolar affective disorder, unspecified | affective disorders |
| 6000711000006112 | Moderate depression | affective disorders |
| 882821000006110 | Moderate depression | affective disorders |
| 2748031000006110 | Moderate major depression, single episode | affective disorders |
| 981181000006117 | Mood swings | affective disorders |
| 31960011 | Mood swings | affective disorders |
| 675861000006113 | Neurotic depression reactive type | affective disorders |
| 253619019 | O/E - depressed | affective disorders |
| 253624016 | O/E - elated | affective disorders |
| 2474715017 | On depression register | affective disorders |
| 1680571000006118 | On full dose long term treatment depression - enh serv admin | affective disorders |
| 681801000000113 | On full dose long term treatment for depression | affective disorders |
| 401522012 | On lithium | affective disorders |
| 1972101000006111 | Organic bipolar affective disorder | affective disorders |
| 294891017 | Other and unspecified manic-depressive psychoses | affective disorders |
| 294896010 | Other and unspecified manic-depressive psychoses NOS | affective disorders |
| 294895014 | Other mixed manic-depressive psychoses | affective disorders |
| 1839561000006119 | PHQ9 total score 10-14 (moderate depression) | affective disorders |
| 1839581000006112 | PHQ9 total score 20-27 (severe depression) | affective disorders |
| 1839551000006116 | PHQ9 total score 5-9 (mild depression) | affective disorders |
| 2533375017 | Patient given advice about management of depression | affective disorders |
| 8035351000006117 | Positive screening for depression on Patient Health Questionnaire 9 | affective disorders |
| 853871000006111 | Post natal depression | affective disorders |
| 223651000000118 | Postnatal depression | affective disorders |
| 6550781000006113 | Postnatal depression counseling | affective disorders |
| 1488771018 | Postnatal depression counselling | affective disorders |
| 3837121000006116 | Postoperative depression | affective disorders |
| 295536013 | Postviral depression | affective disorders |
| 294642013 | Presenile dementia with depression | affective disorders |
| 202561000006114 | Prolonged depressive reaction | affective disorders |
| 3700911000006115 | Psychotic depression | affective disorders |
| 1231868010 | Puerperal depression | affective disorders |
| 882401000006115 | Reactive (neurotic) depression | affective disorders |
| 294917018 | Reactive depressive psychosis | affective disorders |
| 294899015 | Rebound mood swings | affective disorders |
| 294844012 | Recurrent depression | affective disorders |
| 3247081000006110 | Recurrent major depression in complete remission | affective disorders |
| 3247071000006112 | Recurrent major depression in full remission | affective disorders |
| 182721000006111 | Recurrent major depressive episode | affective disorders |
| 294845013 | Recurrent major depressive episode NOS | affective disorders |
| 294843018 | Recurrent major depressive episodes, in full remission | affective disorders |
| 8237831000006115 | Recurrent major depressive episodes, in partial remission | affective disorders |
| 294837015 | Recurrent major depressive episodes, mild | affective disorders |
| 294838013 | Recurrent major depressive episodes, moderate | affective disorders |
| 182771000006112 | Recurrent major depressive episodes, severe, no psychosis | affective disorders |
| 294840015 | Recurrent major depressive episodes, severe, with psychosis | affective disorders |
| 8232591000006117 | Recurrent major depressive episodes, severe, with psychosis, psychosis in remission | affective disorders |
| 1755911000006110 | Recurrent major depressive episodes, severe, with psychosis, psychosis in remission | affective disorders |
| 294836012 | Recurrent major depressive episodes, unspecified | affective disorders |
| 182801000006114 | Recurrent major depressive episodes,partial/unspec remission | affective disorders |
| 294818012 | Recurrent manic episode NOS | affective disorders |
| 294810017 | Recurrent manic episodes | affective disorders |
| 294817019 | Recurrent manic episodes, in full remission | affective disorders |
| 294812013 | Recurrent manic episodes, mild | affective disorders |
| 294813015 | Recurrent manic episodes, moderate | affective disorders |
| 182861000006110 | Recurrent manic episodes, partial or unspecified remission | affective disorders |
| 182871000006115 | Recurrent manic episodes, severe without mention psychosis | affective disorders |
| 294815010 | Recurrent manic episodes, severe, with psychosis | affective disorders |
| 294811018 | Recurrent manic episodes, unspecified | affective disorders |
| 300711000000119 | Referral for guided self-help for depression | affective disorders |
| 2474716016 | Removed from depression register | affective disorders |
| 369982012 | Seasonal affective disorder | affective disorders |
| 294646011 | Senile dementia with depression | affective disorders |
| 3497641000006117 | Severe bipolar I disorder, most recent episode depressed without psychotic features | affective disorders |
| 2501011000006119 | Severe bipolar I disorder, most recent episode manic, without psychotic features | affective disorders |
| 6000721000006116 | Severe depression | affective disorders |
| 882831000006113 | Severe depression | affective disorders |
| 7965131000006117 | Severe major depression, single episode | affective disorders |
| 2957311000006113 | Severe recurrent major depression with psychotic features | affective disorders |
| 3087971000006112 | Severe recurrent major depression without psychotic features | affective disorders |
| 2809811000006111 | Single episode of major depression in full remission | affective disorders |
| 401766011 | Single major depressive episode | affective disorders |
| 294832014 | Single major depressive episode NOS | affective disorders |
| 294831019 | Single major depressive episode, in full remission | affective disorders |
| 12451451000006110 | Single major depressive episode, in full remission | affective disorders |
| 294825017 | Single major depressive episode, mild | affective disorders |
| 294826016 | Single major depressive episode, moderate | affective disorders |
| 12451461000006112 | Single major depressive episode, moderate | affective disorders |
| 142521000006110 | Single major depressive episode, partial or unspec remission | affective disorders |
| 294828015 | Single major depressive episode, severe, with psychosis | affective disorders |
| 1755901000006112 | Single major depressive episode, severe, with psychosis, psychosis in remission | affective disorders |
| 8232571000006118 | Single major depressive episode, severe, with psychosis, psychosis in remission | affective disorders |
| 142541000006115 | Single major depressive episode, severe, without psychosis | affective disorders |
| 294824018 | Single major depressive episode, unspecified | affective disorders |
| 294808019 | Single manic episode in full remission | affective disorders |
| 294807012 | Single manic episode in partial or unspecified remission | affective disorders |
| 294803011 | Single manic episode, mild | affective disorders |
| 294804017 | Single manic episode, moderate | affective disorders |
| 294805016 | Single manic episode, severe without mention of psychosis | affective disorders |
| 294806015 | Single manic episode, severe, with psychosis | affective disorders |
| 294802018 | Single manic episode, unspecified | affective disorders |
| 401523019 | Started lithium | affective disorders |
| 1650771000000113 | Suspected depression | affective disorders |
| 1488626018 | Symptoms of depression | affective disorders |
| 82091000006116 | Unspecified bipolar affect disord, partial/unspec remission | affective disorders |
| 294880012 | Unspecified bipolar affective disorder | affective disorders |
| 294888017 | Unspecified bipolar affective disorder, NOS | affective disorders |
| 294887010 | Unspecified bipolar affective disorder, in full remission | affective disorders |
| 294882016 | Unspecified bipolar affective disorder, mild | affective disorders |
| 294883014 | Unspecified bipolar affective disorder, moderate | affective disorders |
| 82151000006114 | Unspecified bipolar affective disorder, severe, no psychosis | affective disorders |
| 294881011 | Unspecified bipolar affective disorder, unspecified | affective disorders |
| 82171000006116 | Unspecified bipolar affective disorder,severe with psychosis | affective disorders |
| 294892012 | Unspecified manic-depressive psychoses | affective disorders |
| 12718441000006115 | Unspecified puerperal psychosis | affective disorders |
| 294714016 | Unspecified puerperal psychosis | affective disorders |
| 1222477019 | [D]Postoperative depression | affective disorders |
| 909681000006110 | [RFC] Depression | affective disorders |
| 908731000006114 | [RFC] Postnatal depression | affective disorders |
| 345141000006111 | [V]Personal history of manic-depressive psychosis | affective disorders |
| 1227584015 | [V]Personal history of manic-depressive psychosis | affective disorders |
| 359121000006116 | [X] Reactive depression NOS | affective disorders |
| 362781000006116 | [X]Affective psychosis NOS | affective disorders |
| 1785881000006119 | [X]Antenatal depression | affective disorders |
| 366561000006119 | [X]Atypical depression | affective disorders |
| 367051000006112 | [X]Bipol aff disord, curr epis sev depress, no psychot symp | affective disorders |
| 367161000006113 | [X]Bipolar II disorder | affective disorders |
| 1785871000006117 | [X]Bipolar II disorder | affective disorders |
| 367061000006114 | [X]Bipolar affect dis cur epi severe depres with psyc symp | affective disorders |
| 367071000006119 | [X]Bipolar affect disorder cur epi manic with psychotic symp | affective disorders |
| 367081000006116 | [X]Bipolar affect disorder cur epi manic wout psychotic symp | affective disorders |
| 367091000006118 | [X]Bipolar affect disorder cur epi mild or moderate depressn | affective disorders |
| 367101000006112 | [X]Bipolar affective disorder | affective disorders |
| 1785851000006110 | [X]Bipolar affective disorder type I | affective disorders |
| 1785861000006112 | [X]Bipolar affective disorder type II | affective disorders |
| 367111000006110 | [X]Bipolar affective disorder, current episode hypomanic | affective disorders |
| 367121000006119 | [X]Bipolar affective disorder, current episode mixed | affective disorders |
| 296130018 | [X]Bipolar affective disorder, currently in remission | affective disorders |
| 296135011 | [X]Bipolar affective disorder, unspecified | affective disorders |
| 367151000006111 | [X]Bipolar disorder, single manic episode | affective disorders |
| 376291000006118 | [X]Cyclothymia | affective disorders |
| 376691000006116 | [X]Depression NOS | affective disorders |
| 376701000006116 | [X]Depressive conduct disorder | affective disorders |
| 376711000006118 | [X]Depressive disorder NOS | affective disorders |
| 376721000006114 | [X]Depressive episode | affective disorders |
| 401872015 | [X]Depressive episode, unspecified | affective disorders |
| 379431000006113 | [X]Dysthymia | affective disorders |
| 379771000006116 | [X]Endogenous depression with psychotic symptoms | affective disorders |
| 379781000006118 | [X]Endogenous depression without psychotic symptoms | affective disorders |
| 389401000006111 | [X]Hypomania | affective disorders |
| 1715771000006112 | [X]Major depression, mild | affective disorders |
| 1715181000006114 | [X]Major depression, moderately severe | affective disorders |
| 396081000006116 | [X]Major depression, recurrent without psychotic symptoms | affective disorders |
| 1715191000006112 | [X]Major depression, severe with psychotic symptoms | affective disorders |
| 1715781000006110 | [X]Major depression, severe without psychotic symptoms | affective disorders |
| 396691000006111 | [X]Mania NOS | affective disorders |
| 396701000006111 | [X]Mania with mood-congruent psychotic symptoms | affective disorders |
| 396711000006114 | [X]Mania with mood-incongruent psychotic symptoms | affective disorders |
| 401863011 | [X]Mania with psychotic symptoms | affective disorders |
| 296110017 | [X]Mania without psychotic symptoms | affective disorders |
| 396741000006113 | [X]Manic episode | affective disorders |
| 401864017 | [X]Manic episode, unspecified | affective disorders |
| 396771000006117 | [X]Manic-depress psychosis,depressd,no psychotic symptoms | affective disorders |
| 396781000006119 | [X]Manic-depress psychosis,depressed type+psychotic symptoms | affective disorders |
| 396791000006116 | [X]Manic-depressive illness | affective disorders |
| 396801000006115 | [X]Manic-depressive psychosis | affective disorders |
| 396071000006119 | [X]Manic-depressive reaction | affective disorders |
| 398101000006113 | [X]Mental and behav disorders assoc with the puerperium NEC | affective disorders |
| 398281000006112 | [X]Mental/behav dis oth stims inc caffeine: psychotic dis | affective disorders |
| 213641000000111 | [X]Mild depression | affective disorders |
| 11918531000006118 | [X]Mild depressive episode | affective disorders |
| 296137015 | [X]Mild depressive episode | affective disorders |
| 398421000006112 | [X]Mild mental/behav disorder assoc with the puerperium NEC | affective disorders |
| 398541000006116 | [X]Mixed affective episode | affective disorders |
| 11918561000006110 | [X]Moderate depressive episode | affective disorders |
| 296138013 | [X]Moderate depressive episode | affective disorders |
| 398841000006119 | [X]Monopolar depression NOS | affective disorders |
| 398851000006117 | [X]Mood - affective disorders | affective disorders |
| 399961000006118 | [X]Neurotic depression | affective disorders |
| 401865016 | [X]Other bipolar affective disorders | affective disorders |
| 401871010 | [X]Other depressive episodes | affective disorders |
| 296118012 | [X]Other manic episodes | affective disorders |
| 296209016 | [X]Other mood affective disorders | affective disorders |
| 296207019 | [X]Other persistent mood affective disorders | affective disorders |
| 296199015 | [X]Other recurrent depressive disorders | affective disorders |
| 401878016 | [X]Other recurrent mood affective disorders | affective disorders |
| 401877014 | [X]Other single mood affective disorders | affective disorders |
| 296218019 | [X]Other specified mood affective disorders | affective disorders |
| 296208012 | [X]Persistent mood affective disorder, unspecified | affective disorders |
| 296204014 | [X]Persistent mood affective disorders | affective disorders |
| 423131000006113 | [X]Postnatal depression NOS | affective disorders |
| 423141000006115 | [X]Postpartum depression NOS | affective disorders |
| 423611000006111 | [X]Prolonged single episode of reactive depression | affective disorders |
| 296443010 | [X]Puerperal mental disorder, unspecified | affective disorders |
| 424531000006118 | [X]Recurr depress disorder cur epi severe without psyc sympt | affective disorders |
| 424541000006111 | [X]Recurr severe episodes/major depression+psychotic symptom | affective disorders |
| 424551000006113 | [X]Recurr severe episodes/psychogenic depressive psychosis | affective disorders |
| 424561000006110 | [X]Recurrent brief depressive episodes | affective disorders |
| 424571000006115 | [X]Recurrent depress disorder cur epi severe with psyc symp | affective disorders |
| 401873013 | [X]Recurrent depressive disorder | affective disorders |
| 296180012 | [X]Recurrent depressive disorder, current episode mild | affective disorders |
| 296181011 | [X]Recurrent depressive disorder, current episode moderate | affective disorders |
| 296198011 | [X]Recurrent depressive disorder, currently in remission | affective disorders |
| 401876017 | [X]Recurrent depressive disorder, unspecified | affective disorders |
| 424631000006119 | [X]Recurrent episodes of depressive reaction | affective disorders |
| 424641000006112 | [X]Recurrent episodes of psychogenic depression | affective disorders |
| 424651000006114 | [X]Recurrent episodes of reactive depression | affective disorders |
| 424661000006111 | [X]Recurrent manic episodes | affective disorders |
| 424671000006116 | [X]Recurrent severe episodes of psychotic depression | affective disorders |
| 424681000006118 | [X]Recurrent severe episodes/reactive depressive psychosis | affective disorders |
| 11921141000006115 | [X]Recurrent severe episodes/reactive depressive psychosis | affective disorders |
| 425411000006110 | [X]SAD - Seasonal affective disorder | affective disorders |
| 425751000006115 | [X]Seasonal depressive disorder | affective disorders |
| 401869010 | [X]Severe depressive episode with psychotic symptoms | affective disorders |
| 401866015 | [X]Severe depressive episode without psychotic symptoms | affective disorders |
| 11921301000006119 | [X]Severe depressive episode without psychotic symptoms | affective disorders |
| 426581000006113 | [X]Severe mental and behav disorder assoc wth puerperium NEC | affective disorders |
| 426891000006114 | [X]Single episode agitated depressn w'out psychotic symptoms | affective disorders |
| 223741000000112 | [X]Single episode major depression w'out psychotic symptoms | affective disorders |
| 426911000006111 | [X]Single episode of depressive reaction | affective disorders |
| 426921000006115 | [X]Single episode of major depression and psychotic symptoms | affective disorders |
| 426931000006117 | [X]Single episode of masked depression NOS | affective disorders |
| 426941000006110 | [X]Single episode of psychogenic depression | affective disorders |
| 426951000006112 | [X]Single episode of psychogenic depressive psychosis | affective disorders |
| 426961000006114 | [X]Single episode of psychotic depression | affective disorders |
| 426971000006119 | [X]Single episode of reactive depression | affective disorders |
| 426981000006116 | [X]Single episode of reactive depressive psychosis | affective disorders |
| 426991000006118 | [X]Single episode vital depression w'out psychotic symptoms | affective disorders |
| 401879012 | [X]Unspecified mood affective disorder | affective disorders |
| 432511000006119 | [X]Vital depression, recurrent without psychotic symptoms | affective disorders |
| 972931000006117 | ** The treatment of anxiety disorders | anxiety&neurosis |
| 4981541000006112 | AMT - Anxiety management training | anxiety&neurosis |
| 97986013 | Acrophobia | anxiety&neurosis |
| 295475018 | Acute fugue state due to acute stress reaction | anxiety&neurosis |
| 295474019 | Acute panic state due to acute stress reaction | anxiety&neurosis |
| 459711000006119 | Acute posttrauma stress state | anxiety&neurosis |
| 500650019 | Acute reaction to stress | anxiety&neurosis |
| 295478016 | Acute situational disturbance | anxiety&neurosis |
| 401810015 | Acute stress reaction NOS | anxiety&neurosis |
| 295476017 | Acute stupor state due to acute stress reaction | anxiety&neurosis |
| 3265831000006118 | Adjustment disorder with anxiety | anxiety&neurosis |
| 478089014 | Adjustment reaction | anxiety&neurosis |
| 295519017 | Adjustment reaction NOS | anxiety&neurosis |
| 461701000006114 | Adjustment reaction due to hospitalisation | anxiety&neurosis |
| 295505017 | Adjustment reaction with aggression | anxiety&neurosis |
| 295507013 | Adjustment reaction with antisocial behaviour | anxiety&neurosis |
| 1230451012 | Adjustment reaction with anxious mood | anxiety&neurosis |
| 295508015 | Adjustment reaction with destructiveness | anxiety&neurosis |
| 461761000006110 | Adjustment reaction with disturbance emotion and conduct | anxiety&neurosis |
| 295504018 | Adjustment reaction with disturbance of other emotion NOS | anxiety&neurosis |
| 497343017 | Adjustment reaction with mixed disturbance of emotion | anxiety&neurosis |
| 295513016 | Adjustment reaction with physical symptoms | anxiety&neurosis |
| 506324018 | Adjustment reaction with predominant disturbance of conduct | anxiety&neurosis |
| 461821000006111 | Adjustment reaction, predominant disturbance other emotions | anxiety&neurosis |
| 295498014 | Adolescent emancipation disorder | anxiety&neurosis |
| 882381000006115 | Agoraphobia | anxiety&neurosis |
| 988881000006118 | Agoraphobia | anxiety&neurosis |
| 294992011 | Agoraphobia with panic attacks | anxiety&neurosis |
| 294993018 | Agoraphobia without mention of panic attacks | anxiety&neurosis |
| 12716981000006115 | Agoraphobia without mention of panic attacks | anxiety&neurosis |
| 484680014 | Air swallowing - excessive | anxiety&neurosis |
| 3870011000006111 | Amnesia neurosis | anxiety&neurosis |
| 295011010 | Anancastic neurosis | anxiety&neurosis |
| 4764861000006119 | Anankastic neurosis | anxiety&neurosis |
| 1231321013 | Animal phobia | anxiety&neurosis |
| 283464017 | Antiphobic therapy | anxiety&neurosis |
| 3287741000006111 | Anxiety | anxiety&neurosis |
| 2287071000000115 | Anxiety about breathlessness | anxiety&neurosis |
| 6025531000006111 | Anxiety counseling | anxiety&neurosis |
| 6025521000006113 | Anxiety counselling | anxiety&neurosis |
| 5024071000006118 | Anxiety depression | anxiety&neurosis |
| 342665019 | Anxiety management training | anxiety&neurosis |
| 3287771000006115 | Anxiety reaction | anxiety&neurosis |
| 2618241000000117 | Anxiety resolved | anxiety&neurosis |
| 304838011 | Anxiety state | anxiety&neurosis |
| 294963012 | Anxiety state NOS | anxiety&neurosis |
| 294953016 | Anxiety state unspecified | anxiety&neurosis |
| 488201000006114 | Anxiety states | anxiety&neurosis |
| 488211000006112 | Anxiety with depression | anxiety&neurosis |
| 489141000006110 | Aphonia - hysterical | anxiety&neurosis |
| 496061000006116 | Astasia - abasia, hysterical | anxiety&neurosis |
| 223621000000111 | Ataxia - hysterical | anxiety&neurosis |
| 502727013 | Bereavement reaction | anxiety&neurosis |
| 1861181000006114 | Breathlessness causing anxiety | anxiety&neurosis |
| 1785963019 | Briquet's disorder | anxiety&neurosis |
| 295393016 | Bruxism (teeth grinding) | anxiety&neurosis |
| 1488717011 | C/O - panic attack | anxiety&neurosis |
| 485870016 | Cancer phobia | anxiety&neurosis |
| 2162219012 | Cardiac neurosis | anxiety&neurosis |
| 294960010 | Chronic anxiety | anxiety&neurosis |
| 33475016 | Claustrophobia | anxiety&neurosis |
| 101627011 | Combat fatigue | anxiety&neurosis |
| 56251019 | Compensation neurosis | anxiety&neurosis |
| 6550101000006115 | Complaining of panic attack | anxiety&neurosis |
| 295015018 | Compulsive neurosis | anxiety&neurosis |
| 295511019 | Concentration camp syndrome | anxiety&neurosis |
| 6025541000006118 | Counseling for anxiety | anxiety&neurosis |
| 6025551000006116 | Counselling for anxiety | anxiety&neurosis |
| 295503012 | Culture shock | anxiety&neurosis |
| 65032014 | Dental phobia | anxiety&neurosis |
| 3651081000006115 | Depersonalisation neurosis | anxiety&neurosis |
| 1216885017 | Depersonalisation syndrome | anxiety&neurosis |
| 3651071000006118 | Depersonalization neurosis | anxiety&neurosis |
| 294978017 | Dissociative reaction unspecified | anxiety&neurosis |
| 295499018 | Early adult emancipation disorder | anxiety&neurosis |
| 8297491000006114 | Education about anxiety | anxiety&neurosis |
| 295514010 | Elective mutism due to an adjustment reaction | anxiety&neurosis |
| 6358151000006115 | Episodic paroxysmal anxiety disorder | anxiety&neurosis |
| 346985015 | Examination fear | anxiety&neurosis |
| 294998010 | Fear of crowds | anxiety&neurosis |
| 166341019 | Fear of death | anxiety&neurosis |
| 370048011 | Fear of flying | anxiety&neurosis |
| 295004011 | Fear of pregnancy | anxiety&neurosis |
| 760791000006116 | Fit - hysterical | anxiety&neurosis |
| 370049015 | Flying phobia | anxiety&neurosis |
| 2848311000006117 | GAD - Generalised anxiety disorder | anxiety&neurosis |
| 2848301000006115 | GAD - Generalized anxiety disorder | anxiety&neurosis |
| 798571000006111 | Ganser's syndrome - hysterical | anxiety&neurosis |
| 481154010 | Generalised anxiety disorder | anxiety&neurosis |
| 566931000000114 | Generalised anxiety disorder 7 item score | anxiety&neurosis |
| 2848291000006116 | Generalized anxiety disorder | anxiety&neurosis |
| 346997019 | Globus abdominalis | anxiety&neurosis |
| 73437013 | Globus hystericus | anxiety&neurosis |
| 123751014 | Grief reaction | anxiety&neurosis |
| 2533632018 | H/O: agoraphobia | anxiety&neurosis |
| 251630012 | H/O: anxiety state | anxiety&neurosis |
| 6857231000006117 | History of agoraphobia | anxiety&neurosis |
| 4539991000006111 | History of anxiety state | anxiety&neurosis |
| 30729012 | Hypochondriasis | anxiety&neurosis |
| 493878012 | Hysteria | anxiety&neurosis |
| 401779019 | Hysteria NOS | anxiety&neurosis |
| 294964018 | Hysteria unspecified | anxiety&neurosis |
| 787521000006112 | Hysterical amnesia | anxiety&neurosis |
| 147404016 | Hysterical blindness | anxiety&neurosis |
| 117876016 | Hysterical deafness | anxiety&neurosis |
| 3214951000006118 | Hysterical disorder | anxiety&neurosis |
| 4764661000006115 | Hysterical fit | anxiety&neurosis |
| 7046013 | Hysterical fugue | anxiety&neurosis |
| 3214941000006115 | Hysterical neurosis, conversion type | anxiety&neurosis |
| 3214931000006113 | Hysterical neurosis, dissociative type | anxiety&neurosis |
| 147537017 | Hysterical paralysis | anxiety&neurosis |
| 882431000006111 | Hysterical personality | anxiety&neurosis |
| 3398571000006119 | Hysterical personality disorder | anxiety&neurosis |
| 294968015 | Hysterical seizures | anxiety&neurosis |
| 294965017 | Hysterical tremor | anxiety&neurosis |
| 3099611000006115 | Hysterical vomiting | anxiety&neurosis |
| 3385931000006117 | Isolated phobia | anxiety&neurosis |
| 7663271000006111 | Management of anxiety | anxiety&neurosis |
| 1976491000006113 | Mixed anxiety and depressive reaction | anxiety&neurosis |
| 1777688018 | Muscular headache | anxiety&neurosis |
| 130538010 | Nervous breakdown | anxiety&neurosis |
| 677031000006115 | Nervous exhaustion | anxiety&neurosis |
| 677321000006115 | Neurasthenia - nervous debility | anxiety&neurosis |
| 295360013 | Neurocirculatory asthenia | anxiety&neurosis |
| 675731000006112 | Neuroses or other mental disorder NOS | anxiety&neurosis |
| 401783019 | Neurotic disorder NOS | anxiety&neurosis |
| 675881000006115 | Neurotic disorders | anxiety&neurosis |
| 2549895012 | O/E - panic attack | anxiety&neurosis |
| 4764871000006114 | OCD - Obsessive-compulsive disorder | anxiety&neurosis |
| 853201000006116 | Obsessional compulsive psychosis | anxiety&neurosis |
| 295016017 | Obsessional neurosis | anxiety&neurosis |
| 7488401000006113 | Obsessional personality disorder | anxiety&neurosis |
| 3600941000006114 | Obsessional ruminations | anxiety&neurosis |
| 500817017 | Obsessional thoughts | anxiety&neurosis |
| 2698661000006113 | Obsessive compulsive behavior | anxiety&neurosis |
| 2698651000006111 | Obsessive compulsive behaviour | anxiety&neurosis |
| 4764881000006112 | Obsessive compulsive disorder | anxiety&neurosis |
| 4764891000006110 | Obsessive compulsive neurosis | anxiety&neurosis |
| 2520371000006111 | Obsessive compulsive personality disorder | anxiety&neurosis |
| 295017014 | Obsessive-compulsive disorder NOS | anxiety&neurosis |
| 269821000006113 | Obsessive-compulsive disorders | anxiety&neurosis |
| 6910391000006112 | On examination - panic attack | anxiety&neurosis |
| 2777721000006112 | Organic anxiety disorder | anxiety&neurosis |
| 295483012 | Other acute stress reaction NOS | anxiety&neurosis |
| 295477014 | Other acute stress reactions | anxiety&neurosis |
| 295517015 | Other adjustment reaction with withdrawal | anxiety&neurosis |
| 295510018 | Other adjustment reactions | anxiety&neurosis |
| 295518013 | Other adjustment reactions NOS | anxiety&neurosis |
| 401778010 | Other conversion disorder | anxiety&neurosis |
| 295033012 | Other neurotic disorder NOS | anxiety&neurosis |
| 295026012 | Other neurotic disorders | anxiety&neurosis |
| 295031014 | Other occupational neurosis | anxiety&neurosis |
| 882391000006117 | Other phobias | anxiety&neurosis |
| 295512014 | Other post-traumatic stress disorder | anxiety&neurosis |
| 12730101000006118 | Other post-traumatic stress disorder | anxiety&neurosis |
| 295392014 | Other psychogenic malfunction | anxiety&neurosis |
| 295394010 | Other psychogenic malfunction NOS | anxiety&neurosis |
| 9331000006113 | Other specified neuroses or other mental disorders | anxiety&neurosis |
| 3268081000006115 | PTSD - Post-traumatic stress disorder | anxiety&neurosis |
| 339044013 | Panic attack | anxiety&neurosis |
| 1210253015 | Panic disorder | anxiety&neurosis |
| 1808521000006110 | Panic disorder without agoraphobia | anxiety&neurosis |
| 2335571000000115 | Patient given advice about management of anxiety | anxiety&neurosis |
| 2648186013 | Phantom pregnancy | anxiety&neurosis |
| 3649691000006114 | Phobia of going out | anxiety&neurosis |
| 294991016 | Phobia unspecified | anxiety&neurosis |
| 223641000000116 | Phobic anxiety | anxiety&neurosis |
| 853241000006119 | Phobic anxiety | anxiety&neurosis |
| 12731491000006112 | Phobic disorder NOS | anxiety&neurosis |
| 401780016 | Phobic disorder NOS | anxiety&neurosis |
| 223631000000113 | Phobic disorders | anxiety&neurosis |
| 882371000006118 | Phobic state | anxiety&neurosis |
| 103848017 | Photophobia | anxiety&neurosis |
| 232501000006118 | Physiological malfunction arising from mental factors | anxiety&neurosis |
| 3268091000006117 | Post-traumatic stress syndrome | anxiety&neurosis |
| 199101000006113 | Psychalgia | anxiety&neurosis |
| 295465016 | Psychalgia NOS | anxiety&neurosis |
| 295032019 | Psychasthenic neurosis | anxiety&neurosis |
| 1806261000006117 | Psycho-education - anxiety | anxiety&neurosis |
| 484681013 | Psychogenic aerophagy | anxiety&neurosis |
| 295348012 | Psychogenic air hunger | anxiety&neurosis |
| 158091017 | Psychogenic aphonia | anxiety&neurosis |
| 295464017 | Psychogenic backache | anxiety&neurosis |
| 199321000006118 | Psychogenic cardiovascular disorder | anxiety&neurosis |
| 295362017 | Psychogenic cardiovascular symptom NOS | anxiety&neurosis |
| 199341000006113 | Psychogenic cardiovascular symptoms | anxiety&neurosis |
| 295382018 | Psychogenic constipation | anxiety&neurosis |
| 295349016 | Psychogenic cough | anxiety&neurosis |
| 401804015 | Psychogenic diarrhoea | anxiety&neurosis |
| 295387012 | Psychogenic dysmenorrhea | anxiety&neurosis |
| 295381013 | Psychogenic dyspepsia | anxiety&neurosis |
| 295388019 | Psychogenic dysuria | anxiety&neurosis |
| 295383011 | Psychogenic gastrointestinal tract symptom NOS | anxiety&neurosis |
| 199441000006117 | Psychogenic gastrointestinal tract symptoms | anxiety&neurosis |
| 295389010 | Psychogenic genitourinary tract symptom NOS | anxiety&neurosis |
| 295384017 | Psychogenic genitourinary tract symptoms | anxiety&neurosis |
| 295350016 | Psychogenic hiccough | anxiety&neurosis |
| 295351017 | Psychogenic hyperventilation | anxiety&neurosis |
| 295342013 | Psychogenic musculoskeletal symptoms | anxiety&neurosis |
| 295346011 | Psychogenic musculoskeletal symptoms NOS | anxiety&neurosis |
| 295460014 | Psychogenic pain unspecified | anxiety&neurosis |
| 199531000006114 | Psychogenic paralysis | anxiety&neurosis |
| 295364016 | Psychogenic pruritus | anxiety&neurosis |
| 295357018 | Psychogenic respiratory symptom NOS | anxiety&neurosis |
| 199581000006110 | Psychogenic respiratory symptoms | anxiety&neurosis |
| 295363010 | Psychogenic skin symptoms | anxiety&neurosis |
| 295366019 | Psychogenic skin symptoms NOS | anxiety&neurosis |
| 295391019 | Psychogenic symptom of special sense organ | anxiety&neurosis |
| 108724018 | Psychogenic torticollis | anxiety&neurosis |
| 199671000006111 | Psychogenic vaginismus | anxiety&neurosis |
| 295355014 | Psychogenic yawning | anxiety&neurosis |
| 295395011 | Psychosomatic disorder NOS | anxiety&neurosis |
| 294961014 | Recurrent anxiety | anxiety&neurosis |
| 1808511000006119 | Recurrent panic attacks | anxiety&neurosis |
| 300681000000118 | Referral for guided self-help for anxiety | anxiety&neurosis |
| 2391581000000111 | Referral for guided self-help for anxiety declined | anxiety&neurosis |
| 2618361000000113 | Referral for psychological management of anxiety | anxiety&neurosis |
| 2618401000000116 | Referral for psychological management of anxiety declined | anxiety&neurosis |
| 851351000006112 | School phobia | anxiety&neurosis |
| 441512015 | Separation anxiety disorder | anxiety&neurosis |
| 90724016 | Simple phobia | anxiety&neurosis |
| 2907991000006110 | Social anxiety disorder | anxiety&neurosis |
| 294994012 | Social phobia, fear of eating in public | anxiety&neurosis |
| 294995013 | Social phobia, fear of public speaking | anxiety&neurosis |
| 294996014 | Social phobia, fear of public washing | anxiety&neurosis |
| 138401000006119 | Social phobic disorders | anxiety&neurosis |
| 1774451016 | Somatization disorder | anxiety&neurosis |
| 401811016 | Specific academic or work inhibition | anxiety&neurosis |
| 133361000006119 | Specific academic or work inhibition | anxiety&neurosis |
| 411460011 | Specific work inhibition | anxiety&neurosis |
| 196858012 | Spurious diarrhoea | anxiety&neurosis |
| 346984016 | Stage fright | anxiety&neurosis |
| 121871000006118 | Stress reaction causing mixed disturbance of emotion/conduct | anxiety&neurosis |
| 1809331000006113 | Symptoms of agoraphobia | anxiety&neurosis |
| 1786078018 | Tension headache | anxiety&neurosis |
| 397879018 | Tired all the time | anxiety&neurosis |
| 3268071000006118 | Traumatic neurosis | anxiety&neurosis |
| 347004010 | Weight fixation | anxiety&neurosis |
| 295029017 | Writer's cramp neurosis | anxiety&neurosis |
| 909691000006113 | [RFC] Anxiety management | anxiety&neurosis |
| 460275012 | [V]Personal history of neurosis | anxiety&neurosis |
| 362181000006117 | [X]Acrophobia | anxiety&neurosis |
| 362301000006112 | [X]Acute crisis reaction | anxiety&neurosis |
| 1755921000006119 | [X]Acute post-traumatic stress disorder follow military comb | anxiety&neurosis |
| 362401000006115 | [X]Acute reaction to stress | anxiety&neurosis |
| 362441000006118 | [X]Acute stress reaction | anxiety&neurosis |
| 362691000006113 | [X]Adjustment disorders | anxiety&neurosis |
| 362871000006111 | [X]Agoraphobia | anxiety&neurosis |
| 362881000006114 | [X]Agoraphobia without history of panic disorder | anxiety&neurosis |
| 363211000006112 | [X]Anankastic neurosis | anxiety&neurosis |
| 363281000006117 | [X]Animal phobias | anxiety&neurosis |
| 363361000006117 | [X]Anthropophobia | anxiety&neurosis |
| 363661000006113 | [X]Anxiety NOS | anxiety&neurosis |
| 296249012 | [X]Anxiety disorder, unspecified | anxiety&neurosis |
| 363641000006114 | [X]Anxiety hysteria | anxiety&neurosis |
| 363651000006111 | [X]Anxiety neurosis | anxiety&neurosis |
| 363671000006118 | [X]Anxiety reaction | anxiety&neurosis |
| 363681000006115 | [X]Anxiety state | anxiety&neurosis |
| 367931000006118 | [X]Body dysmorphic disorder | anxiety&neurosis |
| 368021000006119 | [X]Briquet's disorder | anxiety&neurosis |
| 215901000000115 | [X]Briquet's syndrome | anxiety&neurosis |
| 2162217014 | [X]Cardiac neurosis | anxiety&neurosis |
| 370861000006110 | [X]Childhood overanxious disorder | anxiety&neurosis |
| 1755931000006116 | [X]Chron post-traumatic stress disorder follow military comb | anxiety&neurosis |
| 371071000006117 | [X]Claustrophobia | anxiety&neurosis |
| 371231000006113 | [X]Combat fatigue | anxiety&neurosis |
| 375721000006119 | [X]Conversion hysteria | anxiety&neurosis |
| 375731000006116 | [X]Conversion reaction | anxiety&neurosis |
| 375971000006116 | [X]Crisis state | anxiety&neurosis |
| 376221000006115 | [X]Culture shock | anxiety&neurosis |
| 2162210011 | [X]Da Costa's syndrome | anxiety&neurosis |
| 1755941000006114 | [X]Delayed post-traumat stress disorder follow military comb | anxiety&neurosis |
| 376681000006119 | [X]Depersonalization - derealization syndrome | anxiety&neurosis |
| 376741000006119 | [X]Depressive neurosis | anxiety&neurosis |
| 376991000006113 | [X]Dhat syndrome | anxiety&neurosis |
| 296298015 | [X]Dissociative [conversion] disorder, unspecified | anxiety&neurosis |
| 296273016 | [X]Dissociative [conversion] disorders | anxiety&neurosis |
| 378101000006119 | [X]Dissociative amnesia | anxiety&neurosis |
| 401883012 | [X]Dissociative anaesthesia and sensory loss | anxiety&neurosis |
| 378121000006112 | [X]Dissociative convulsions | anxiety&neurosis |
| 378131000006110 | [X]Dissociative fugue | anxiety&neurosis |
| 378141000006117 | [X]Dissociative motor disorders | anxiety&neurosis |
| 378151000006115 | [X]Dissociative stupor | anxiety&neurosis |
| 378291000006117 | [X]Dream anxiety disorder | anxiety&neurosis |
| 379411000006119 | [X]Dysmorphophobia nondelusional | anxiety&neurosis |
| 386731000006116 | [X]Fatigue syndrome | anxiety&neurosis |
| 387831000006114 | [X]Ganser's syndrome | anxiety&neurosis |
| 1494942017 | [X]Gastric neurosis | anxiety&neurosis |
| 388071000006116 | [X]Generalized anxiety disorder | anxiety&neurosis |
| 388131000006118 | [X]Globus hystericus | anxiety&neurosis |
| 1742861000006114 | [X]Globus hystericus | anxiety&neurosis |
| 345691000000118 | [X]Globus pharyngeus | anxiety&neurosis |
| 388271000006110 | [X]Grief reaction | anxiety&neurosis |
| 389151000006118 | [X]Hospitalism in children | anxiety&neurosis |
| 389371000006113 | [X]Hypochondriacal disorder | anxiety&neurosis |
| 389381000006111 | [X]Hypochondriacal neurosis | anxiety&neurosis |
| 389391000006114 | [X]Hypochondriasis | anxiety&neurosis |
| 389431000006115 | [X]Hysteria | anxiety&neurosis |
| 398351000006110 | [X]Mild anxiety depression | anxiety&neurosis |
| 398561000006117 | [X]Mixed anxiety and depressive disorder | anxiety&neurosis |
| 296289018 | [X]Mixed dissociative [conversion] disorders | anxiety&neurosis |
| 296253014 | [X]Mixed obsessional thoughts and acts | anxiety&neurosis |
| 399111000006118 | [X]Multiple psychosomatic disorder | anxiety&neurosis |
| 980191000006111 | [X]Needle phobia | anxiety&neurosis |
| 399841000006110 | [X]Neurasthenia | anxiety&neurosis |
| 2162212015 | [X]Neurocirculatory asthenia | anxiety&neurosis |
| 1222256015 | [X]Neurosis NOS | anxiety&neurosis |
| 296358015 | [X]Neurotic disorder, unspecified | anxiety&neurosis |
| 399981000006111 | [X]Neurotic, stress - related and somoform disorders | anxiety&neurosis |
| 400201000006111 | [X]Nosophobia | anxiety&neurosis |
| 400321000006113 | [X]Obsessive - compulsive disorder | anxiety&neurosis |
| 296255019 | [X]Obsessive-compulsive disorder, unspecified | anxiety&neurosis |
| 400341000006118 | [X]Obsessive-compulsive neurosis | anxiety&neurosis |
| 403531000006111 | [X]Occupational neurosis, including writer's cramp | anxiety&neurosis |
| 12703831000006115 | [X]Organic anxiety disorder | anxiety&neurosis |
| 403931000006116 | [X]Organic anxiety disorder | anxiety&neurosis |
| 296238018 | [X]Other anxiety disorders | anxiety&neurosis |
| 401885017 | [X]Other dissociative [conversion] disorders | anxiety&neurosis |
| 296245018 | [X]Other mixed anxiety disorders | anxiety&neurosis |
| 296341013 | [X]Other neurotic disorders | anxiety&neurosis |
| 296254015 | [X]Other obsessive-compulsive disorders | anxiety&neurosis |
| 296236019 | [X]Other phobic anxiety disorders | anxiety&neurosis |
| 296271019 | [X]Other reactions to severe stress | anxiety&neurosis |
| 401887013 | [X]Other somatoform disorders | anxiety&neurosis |
| 401881014 | [X]Other specified anxiety disorders | anxiety&neurosis |
| 401888015 | [X]Other specified neurotic disorders | anxiety&neurosis |
| 418031000006113 | [X]Panic attack | anxiety&neurosis |
| 296239014 | [X]Panic disorder [episodic paroxysmal anxiety] | anxiety&neurosis |
| 418051000006118 | [X]Panic disorder with agoraphobia | anxiety&neurosis |
| 418061000006116 | [X]Panic state | anxiety&neurosis |
| 419841000006116 | [X]Persistant anxiety depression | anxiety&neurosis |
| 401886016 | [X]Persistent somatoform pain disorder | anxiety&neurosis |
| 11920891000006113 | [X]Phobia NOS | anxiety&neurosis |
| 420631000006114 | [X]Phobia NOS | anxiety&neurosis |
| 420641000006116 | [X]Phobic anxiety disorder of childhood | anxiety&neurosis |
| 296237011 | [X]Phobic anxiety disorder, unspecified | anxiety&neurosis |
| 296224013 | [X]Phobic anxiety disorders | anxiety&neurosis |
| 420671000006112 | [X]Phobic state NOS | anxiety&neurosis |
| 423021000006114 | [X]Post - traumatic stress disorder | anxiety&neurosis |
| 296252016 | [X]Predominantly compulsive acts [obsessional rituals] | anxiety&neurosis |
| 296251011 | [X]Predominantly obsessional thoughts or ruminations | anxiety&neurosis |
| 423751000006119 | [X]Pseudoseizures | anxiety&neurosis |
| 423781000006110 | [X]Psychalgia | anxiety&neurosis |
| 423791000006113 | [X]Psychasthenia | anxiety&neurosis |
| 423801000006114 | [X]Psychasthenia neurosis | anxiety&neurosis |
| 423811000006112 | [X]Psychic shock | anxiety&neurosis |
| 1495560015 | [X]Psychogenic IBS | anxiety&neurosis |
| 423841000006111 | [X]Psychogenic aphonia | anxiety&neurosis |
| 423851000006113 | [X]Psychogenic backache | anxiety&neurosis |
| 423861000006110 | [X]Psychogenic confusion | anxiety&neurosis |
| 1494938015 | [X]Psychogenic cough | anxiety&neurosis |
| 423881000006117 | [X]Psychogenic deafness | anxiety&neurosis |
| 1490285011 | [X]Psychogenic diarrhoea | anxiety&neurosis |
| 215921000000112 | [X]Psychogenic dysmenorrhoea | anxiety&neurosis |
| 1494941012 | [X]Psychogenic dyspepsia | anxiety&neurosis |
| 215881000000118 | [X]Psychogenic dysphonia | anxiety&neurosis |
| 1494943010 | [X]Psychogenic dysuria | anxiety&neurosis |
| 423951000006116 | [X]Psychogenic flatulence | anxiety&neurosis |
| 423961000006119 | [X]Psychogenic freq micturit | anxiety&neurosis |
| 423971000006114 | [X]Psychogenic headache | anxiety&neurosis |
| 1494939011 | [X]Psychogenic hiccough | anxiety&neurosis |
| 1494940013 | [X]Psychogenic hyperventilat | anxiety&neurosis |
| 424091000006113 | [X]Psychogenic pruritis | anxiety&neurosis |
| 1491595012 | [X]Psychogenic pylorospasm | anxiety&neurosis |
| 424111000006116 | [X]Psychogenic syncope | anxiety&neurosis |
| 424121000006112 | [X]Psychogenic torticollis | anxiety&neurosis |
| 424131000006110 | [X]Psychogenic twilight state | anxiety&neurosis |
| 424241000006114 | [X]Psychosomatic disorder NOS | anxiety&neurosis |
| 424471000006119 | [X]Reaction to severe stress, and adjustment disorders | anxiety&neurosis |
| 296272014 | [X]Reaction to severe stress, unspecified | anxiety&neurosis |
| 296722013 | [X]Separation anxiety disorder of childhood | anxiety&neurosis |
| 426871000006113 | [X]Simple phobia | anxiety&neurosis |
| 427051000006113 | [X]Social anxiety disorder of childhood | anxiety&neurosis |
| 427061000006110 | [X]Social neurosis | anxiety&neurosis |
| 427071000006115 | [X]Social phobias | anxiety&neurosis |
| 215891000000116 | [X]Somatization disorder | anxiety&neurosis |
| 1495057015 | [X]Somatoform autonomic dysfunction | anxiety&neurosis |
| 296340014 | [X]Somatoform disorder, unspecified | anxiety&neurosis |
| 427131000006117 | [X]Somatoform disorders | anxiety&neurosis |
| 427141000006110 | [X]Somatoform pain disorder | anxiety&neurosis |
| 427231000006111 | [X]Specific (isolated) phobias | anxiety&neurosis |
| 428641000006119 | [X]Teeth-grinding | anxiety&neurosis |
| 296277015 | [X]Trance and possession disorders | anxiety&neurosis |
| 429421000006116 | [X]Traumatic neurosis | anxiety&neurosis |
| 429691000006116 | [X]Undifferentiated psychosomatic disorder | anxiety&neurosis |
| 429711000006118 | [X]Undifferentiated somatoform disorder | anxiety&neurosis |
| 94597012 | Anorexia nervosa | eating disorders |
| 372208017 | Anorexia symptom | eating disorders |
| 404431014 | Appetite loss - anorexia | eating disorders |
| 370419010 | Binge eating | eating disorders |
| 527421000006116 | Bulimia (non-organic overeating) | eating disorders |
| 577901000006119 | Compulsive eating disorder | eating disorders |
| 295374018 | Cyclical vomiting - psychogenic | eating disorders |
| 1127871000000119 | Dissatisfied with body image | eating disorders |
| 397863019 | Excessive eating - polyphagia | eating disorders |
| 251631011 | H/O: anorexia nervosa | eating disorders |
| 830401000006110 | Hyperalimentation - symptom | eating disorders |
| 2159265012 | Nocturnal sleep-related eating disorder | eating disorders |
| 295452013 | Non-organic eating disorder NOS | eating disorders |
| 295436010 | Other and unspecified non-organic eating disorders | eating disorders |
| 295451018 | Other specified non-organic eating disorder | eating disorders |
| 407104010 | Polyphagia symptom | eating disorders |
| 1780181019 | Referral to eating disorders clinic | eating disorders |
| 664031000000110 | Seen in eating disorder clinic | eating disorders |
| 324685013 | Starvation | eating disorders |
| 2308361000000115 | Suspected binge eating disorder | eating disorders |
| 295437018 | Unspecified non-organic eating disorder | eating disorders |
| 317233013 | [D]Anorexia | eating disorders |
| 317235018 | [D]Anorexia NOS | eating disorders |
| 1222496015 | [D]Bulimia NOS | eating disorders |
| 317257013 | [D]Excessive eating | eating disorders |
| 317258015 | [D]Hyperalimentation | eating disorders |
| 317255017 | [D]Polyphagia | eating disorders |
| 317259011 | [D]Polyphagia NOS | eating disorders |
| 363321000006111 | [X]Anorexia nervosa | eating disorders |
| 366521000006113 | [X]Atypical anorexia nervosa | eating disorders |
| 366541000006118 | [X]Atypical bulimia nervosa | eating disorders |
| 368061000006113 | [X]Bulimia NOS | eating disorders |
| 368051000006111 | [X]Bulimia nervosa | eating disorders |
| 296379015 | [X]Eating disorder, unspecified | eating disorders |
| 296361019 | [X]Eating disorders | eating disorders |
| 389311000006116 | [X]Hyperorexia nervosa | eating disorders |
| 401889011 | [X]Other eating disorders | eating disorders |
| 417851000006113 | [X]Overeating associated with other psychological disturbncs | eating disorders |
| 420741000006110 | [X]Pica in adults | eating disorders |
| 424061000006117 | [X]Psychogenic overeating | eating disorders |
| 334547013 | [X]Starvation | eating disorders |
| 1495439012 | Affective personality disorder | personality disorders |
| 295048018 | Affective personality disorder NOS | personality disorders |
| 474121000006111 | Aggressive personality | personality disorders |
| 295543019 | Aggressive unsocial conduct disorder | personality disorders |
| 480071000006112 | Amoral personality | personality disorders |
| 481471000006112 | Anancastic personality | personality disorders |
| 481481000006110 | Anankastic personality | personality disorders |
| 487991000006113 | Antisocial or sociopathic personality disorder | personality disorders |
| 496091000006112 | Asthenic personality | personality disorders |
| 62962010 | Avoidant personality disorder | personality disorders |
| 33677019 | Borderline personality disorder | personality disorders |
| 295063015 | Compulsive personality disorder NOS | personality disorders |
| 577931000006110 | Compulsive personality disorders | personality disorders |
| 605901000006114 | Cyclothymic personality disorder | personality disorders |
| 223661000000115 | Dependent personality | personality disorders |
| 613781000006118 | Depressive personality disorder | personality disorders |
| 401813018 | Disturbance of conduct NEC | personality disorders |
| 295079019 | Eccentric personality disorder | personality disorders |
| 640481000006113 | Emotionally unstable personality | personality disorders |
| 347011014 | Explosive personality disorder | personality disorders |
| 295067019 | Histrionic personality disorder NOS | personality disorders |
| 824751000006111 | Histrionic personality disorders | personality disorders |
| 295045015 | Hypomanic personality disorder | personality disorders |
| 787571000006113 | Hysterical personality disorders | personality disorders |
| 295080016 | Immature personality disorder | personality disorders |
| 782871000006113 | Inadequate personality disorder | personality disorders |
| 735811000006119 | Labile personality | personality disorders |
| 295081017 | Masochistic personality disorder | personality disorders |
| 701591000006118 | Mixed disturbance of conduct and emotion | personality disorders |
| 481069014 | Munchausen's syndrome | personality disorders |
| 133885012 | Narcissistic personality disorder | personality disorders |
| 675901000006118 | Neurotic personality | personality disorders |
| 675911000006115 | Neurotic personality disorder | personality disorders |
| 475487017 | Obsessional personality | personality disorders |
| 401786010 | Other personality disorder NOS | personality disorders |
| 295077017 | Other personality disorders | personality disorders |
| 23211011 | Paranoid personality disorder | personality disorders |
| 1230168015 | Passive-aggressive personality disorder | personality disorders |
| 30550017 | Pathological gambling | personality disorders |
| 401787018 | Personality disorder NOS | personality disorders |
| 230091000006118 | Personality disorders | personality disorders |
| 401785014 | Psychoneurotic personality disorder | personality disorders |
| 199821000006114 | Psychopathic personality | personality disorders |
| 88108012 | Schizoid personality disorder | personality disorders |
| 295052018 | Schizoid personality disorder NOS | personality disorders |
| 155281000006119 | Schizotypal personality | personality disorders |
| 295044016 | Unspecified affective personality disorder | personality disorders |
| 295064014 | Unspecified histrionic personality disorder | personality disorders |
| 295049014 | Unspecified schizoid personality disorder | personality disorders |
| 359041000006112 | [X] Munchausens syndrome | personality disorders |
| 2160091013 | [X]Addictive personality | personality disorders |
| 362761000006114 | [X]Affectionless psychopathy | personality disorders |
| 362771000006119 | [X]Affective personality disorder | personality disorders |
| 362851000006118 | [X]Aggressive personality disorder | personality disorders |
| 363081000006112 | [X]Amoral personality disorder | personality disorders |
| 363221000006116 | [X]Anankastic personality disorder | personality disorders |
| 363561000006112 | [X]Antisocial personality disorder | personality disorders |
| 363691000006117 | [X]Anxious [avoidant] personality disorder | personality disorders |
| 363921000006112 | [X]Asocial personality disorder | personality disorders |
| 366421000006114 | [X]Asthenic personality disorder | personality disorders |
| 366591000006110 | [X]Autistic psychopathy | personality disorders |
| 367941000006111 | [X]Borderline personality disorder | personality disorders |
| 1222259010 | [X]Character neurosis NOS | personality disorders |
| 371011000006114 | [X]Chronic pain personality syndrome | personality disorders |
| 371331000006117 | [X]Compulsive gambling | personality disorders |
| 371341000006110 | [X]Compulsive personality disorder | personality disorders |
| 376261000006114 | [X]Cycloid personality | personality disorders |
| 376301000006117 | [X]Cyclothymic personality | personality disorders |
| 376671000006117 | [X]Dependent personality disorder | personality disorders |
| 376751000006117 | [X]Depressive personality disorder | personality disorders |
| 296458019 | [X]Disorders of adult personality and behaviour | personality disorders |
| 378071000006112 | [X]Dissocial personality disorder | personality disorders |
| 379511000006118 | [X]Eccentric personality disorder | personality disorders |
| 379571000006110 | [X]Elaboration of physical symptoms for psychological reason | personality disorders |
| 379701000006110 | [X]Emotionally unstable personality disorder | personality disorders |
| 379811000006116 | [X]Enduring personality change after catastrophic experience | personality disorders |
| 379821000006112 | [X]Enduring personality change after psychiatric illness | personality disorders |
| 379831000006110 | [X]Enduring personality change not attrib to brain damag/dis | personality disorders |
| 296508011 | [X]Enduring personality change, unspecified | personality disorders |
| 380231000006114 | [X]Expansive paranoid personality disorder | personality disorders |
| 380611000006117 | [X]Explosive personality disorder | personality disorders |
| 1870551000006113 | [X]Factitious disorder | personality disorders |
| 296514016 | [X]Habit and impulse disorder, unspecified | personality disorders |
| 388311000006110 | [X]Habit and impulse disorders | personality disorders |
| 388391000006117 | [X]Haltlose type personality disorder | personality disorders |
| 388871000006111 | [X]Histrionic personality disorder | personality disorders |
| 389441000006113 | [X]Hysterical personality disorder | personality disorders |
| 389491000006116 | [X]Immature personality disorder | personality disorders |
| 389581000006115 | [X]Inadequate personality disorder | personality disorders |
| 392271000006112 | [X]Intent product/feign of symptom/disab eith physical/psych | personality disorders |
| 296489015 | [X]Mixed and other personality disorders | personality disorders |
| 850721000006111 | [X]Munchausen's by proxy | personality disorders |
| 399451000006114 | [X]Narcissistic personality disorder | personality disorders |
| 400311000006117 | [X]Obsessional personality disorder | personality disorders |
| 400351000006116 | [X]Obsessive-compulsive personality disorder | personality disorders |
| 404021000006116 | [X]Organic personality disorder | personality disorders |
| 404031000006118 | [X]Organic pseudopsychopathic personality | personality disorders |
| 296543012 | [X]Other disorders of adult personality and behaviour | personality disorders |
| 401900011 | [X]Other enduring personality changes | personality disorders |
| 296513010 | [X]Other habit and impulse disorders | personality disorders |
| 401898014 | [X]Other specific personality disorders | personality disorders |
| 414781000006118 | [X]Other specified disorders of adult personality/behaviour | personality disorders |
| 418201000006111 | [X]Paranoid personality disorder | personality disorders |
| 418531000006117 | [X]Passive personality disorder | personality disorders |
| 418551000006112 | [X]Pathological fire-setting | personality disorders |
| 418561000006114 | [X]Pathological gambling | personality disorders |
| 418571000006119 | [X]Pathological personality NOS | personality disorders |
| 418581000006116 | [X]Pathological stealing | personality disorders |
| 420421000006113 | [X]Personality change aft prolong captiv+possib/being killed | personality disorders |
| 420431000006111 | [X]Personality change after concentration camp experiences | personality disorders |
| 420441000006118 | [X]Personality change after disasters | personality disorders |
| 420451000006116 | [X]Personality change after torture | personality disorders |
| 296488011 | [X]Personality disorder, unspecified | personality disorders |
| 420501000006119 | [X]Personlty chang aft expos life-threat sit/victim/terrorsm | personality disorders |
| 424161000006118 | [X]Psychoinfantile personality disorder | personality disorders |
| 424201000006112 | [X]Psychoneurotic personality disorder | personality disorders |
| 424211000006110 | [X]Psychopathic personality disorder | personality disorders |
| 424331000006112 | [X]Querulant personality disorder | personality disorders |
| 425591000006118 | [X]Schizoid personality disorder | personality disorders |
| 425731000006110 | [X]Schizotypal personality disorder | personality disorders |
| 425841000006112 | [X]Self defeating personality disorder | personality disorders |
| 425911000006118 | [X]Sensitive paranoid personality disorder | personality disorders |
| 427091000006119 | [X]Sociopathic personality disorder | personality disorders |
| 296460017 | [X]Specific personality disorders | personality disorders |
| 429561000006111 | [X]Trichotillomania | personality disorders |
| 296549011 | [X]Unspecified disorder of adult personality and behaviour | personality disorders |
| 294751010 | Acute exacerbation of chronic catatonic schizophrenia | schizophrenia spectrum |
| 4763641000006116 | Acute exacerbation of chronic disorganised schizophrenia | schizophrenia spectrum |
| 4763661000006117 | Acute exacerbation of chronic disorganized schizophrenia | schizophrenia spectrum |
| 294739013 | Acute exacerbation of chronic hebephrenic schizophrenia | schizophrenia spectrum |
| 294769018 | Acute exacerbation of chronic latent schizophrenia | schizophrenia spectrum |
| 294758016 | Acute exacerbation of chronic paranoid schizophrenia | schizophrenia spectrum |
| 456731000006115 | Acute exacerbation of chronic schizo-affective schizophrenia | schizophrenia spectrum |
| 4763921000006118 | Acute exacerbation of chronic schizoaffective schizophrenia | schizophrenia spectrum |
| 294731011 | Acute exacerbation of chronic schizophrenia | schizophrenia spectrum |
| 294750011 | Acute exacerbation of subchronic catatonic schizophrenia | schizophrenia spectrum |
| 294738017 | Acute exacerbation of subchronic hebephrenic schizophrenia | schizophrenia spectrum |
| 294768014 | Acute exacerbation of subchronic latent schizophrenia | schizophrenia spectrum |
| 294757014 | Acute exacerbation of subchronic paranoid schizophrenia | schizophrenia spectrum |
| 4763911000006114 | Acute exacerbation of subchronic schizoaffective schizophrenia | schizophrenia spectrum |
| 294730012 | Acute exacerbation of subchronic schizophrenia | schizophrenia spectrum |
| 456801000006115 | Acute exacerbation subchronic schizo-affective schizophrenia | schizophrenia spectrum |
| 294919015 | Acute hysterical psychosis | schizophrenia spectrum |
| 401770015 | Acute paranoid reaction | schizophrenia spectrum |
| 1975691000006115 | Acute polymorphic psychot disord with symp of schizophren, with associated acute stress | schizophrenia spectrum |
| 1975681000006118 | Acute polymorphic psychot disord with symp of schizophren, without associated acute stress | schizophrenia spectrum |
| 1975671000006116 | Acute polymorphic psychot disord without symp of schizoph, with associated acute stress | schizophrenia spectrum |
| 1975621000006117 | Acute polymorphic psychot disord without symp of schizoph, without associated acute stress | schizophrenia spectrum |
| 7696261000006113 | Acute polymorphic psychotic disorder with symptoms of schizophrenia | schizophrenia spectrum |
| 882301000006112 | Acute schizo affective psychosis | schizophrenia spectrum |
| 1975731000006111 | Acute schizophrenia-like psychotic disorder, with associated acute stress | schizophrenia spectrum |
| 1975711000006117 | Acute schizophrenia-like psychotic disorder, without associated acute stress | schizophrenia spectrum |
| 401763015 | Acute schizophrenic episode | schizophrenia spectrum |
| 1219653018 | Atypical schizophrenia | schizophrenia spectrum |
| 523481000006114 | Bouffee delirante | schizophrenia spectrum |
| 10122017 | Brief reactive psychosis | schizophrenia spectrum |
| 294742019 | Catatonic schizophrenia | schizophrenia spectrum |
| 294753013 | Catatonic schizophrenia NOS | schizophrenia spectrum |
| 178723016 | Catatonic schizophrenia in remission | schizophrenia spectrum |
| 1974891000006117 | Catatonic schizophrenia, complete remission | schizophrenia spectrum |
| 1974751000006117 | Catatonic schizophrenia, continuous | schizophrenia spectrum |
| 1974921000006111 | Catatonic schizophrenia, course uncertain, period of observation too short | schizophrenia spectrum |
| 1974831000006116 | Catatonic schizophrenia, episodic remittent | schizophrenia spectrum |
| 1974781000006113 | Catatonic schizophrenia, episodic with progressive deficit | schizophrenia spectrum |
| 1974801000006112 | Catatonic schizophrenia, episodic with stable deficit | schizophrenia spectrum |
| 1974861000006113 | Catatonic schizophrenia, incomplete remission | schizophrenia spectrum |
| 1976901000006114 | Catatonic schizophrenia, other | schizophrenia spectrum |
| 294788013 | Cenesthopathic schizophrenia | schizophrenia spectrum |
| 114616017 | Chronic catatonic schizophrenia | schizophrenia spectrum |
| 4763751000006112 | Chronic catatonic schizophrenia with acute exacerbation | schizophrenia spectrum |
| 2705561000006112 | Chronic disorganised schizophrenia | schizophrenia spectrum |
| 2705571000006117 | Chronic disorganized schizophrenia | schizophrenia spectrum |
| 294737010 | Chronic hebephrenic schizophrenia | schizophrenia spectrum |
| 294767016 | Chronic latent schizophrenia | schizophrenia spectrum |
| 401768012 | Chronic paranoid psychosis | schizophrenia spectrum |
| 52897013 | Chronic paranoid schizophrenia | schizophrenia spectrum |
| 4763791000006118 | Chronic paranoid schizophrenia with acute exacerbation | schizophrenia spectrum |
| 556631000006116 | Chronic schizo-affective schizophrenia | schizophrenia spectrum |
| 3862661000006112 | Chronic schizophrenia | schizophrenia spectrum |
| 1234861017 | Chronic schizophrenic | schizophrenia spectrum |
| 294787015 | Coenesthopathic schizophrenia | schizophrenia spectrum |
| 294773015 | Cyclic schizophrenia | schizophrenia spectrum |
| 4569010 | Delusion | schizophrenia spectrum |
| 1452014 | Delusion of persecution | schizophrenia spectrum |
| 480834016 | Delusions | schizophrenia spectrum |
| 119579013 | Disintegrative psychosis | schizophrenia spectrum |
| 3068121000006112 | Disorganised schizophrenia | schizophrenia spectrum |
| 3005591000006114 | Disorganised schizophrenia in remission | schizophrenia spectrum |
| 3068131000006110 | Disorganized schizophrenia | schizophrenia spectrum |
| 3005601000006118 | Disorganized schizophrenia in remission | schizophrenia spectrum |
| 3005611000006115 | Disorganized schizophrenia, in remission | schizophrenia spectrum |
| 294908018 | Folie a deux | schizophrenia spectrum |
| 369805011 | Grandiose delusions | schizophrenia spectrum |
| 300411000000110 | H/O: psychosis | schizophrenia spectrum |
| 251628010 | H/O: schizophrenia | schizophrenia spectrum |
| 501492014 | Hallucinations | schizophrenia spectrum |
| 819351000006115 | Hebephrenic schizophrenia | schizophrenia spectrum |
| 294741014 | Hebephrenic schizophrenia NOS | schizophrenia spectrum |
| 294740010 | Hebephrenic schizophrenia in remission | schizophrenia spectrum |
| 1974691000006116 | Hebephrenic schizophrenia, complete remission | schizophrenia spectrum |
| 1974601000006111 | Hebephrenic schizophrenia, continuous | schizophrenia spectrum |
| 1974721000006114 | Hebephrenic schizophrenia, course uncertain, period of observation too short | schizophrenia spectrum |
| 1974641000006113 | Hebephrenic schizophrenia, episodic remittent | schizophrenia spectrum |
| 1974611000006114 | Hebephrenic schizophrenia, episodic with progressive deficit | schizophrenia spectrum |
| 1974621000006118 | Hebephrenic schizophrenia, episodic with stable deficit | schizophrenia spectrum |
| 1974671000006117 | Hebephrenic schizophrenia, incomplete remission | schizophrenia spectrum |
| 1976891000006110 | Hebephrenic schizophrenia, other | schizophrenia spectrum |
| 4539931000006112 | History of schizophrenia | schizophrenia spectrum |
| 492910018 | Ideas of reference | schizophrenia spectrum |
| 294764011 | Latent schizophrenia | schizophrenia spectrum |
| 294771018 | Latent schizophrenia NOS | schizophrenia spectrum |
| 294770017 | Latent schizophrenia in remission | schizophrenia spectrum |
| 4763861000006116 | Latent schizophrenia, in remission | schizophrenia spectrum |
| 1974651000006110 | Mental & behav dis due to use tobacco: psychotic disorder, schizophrenia-like | schizophrenia spectrum |
| 5517971000006118 | Mixed schizophrenic and affective psychosis | schizophrenia spectrum |
| 294724012 | Non-organic psychoses | schizophrenia spectrum |
| 401771016 | Non-organic psychosis NOS | schizophrenia spectrum |
| 223611000000117 | Nonorganic psychosis NOS | schizophrenia spectrum |
| 2549426013 | O/E - delusion of persecution | schizophrenia spectrum |
| 2549129012 | O/E - paranoid delusions | schizophrenia spectrum |
| 450785011 | Oneirophrenia | schizophrenia spectrum |
| 294902017 | Other affective psychosis NOS | schizophrenia spectrum |
| 294897018 | Other and unspecified affective psychoses | schizophrenia spectrum |
| 25461000006115 | Other nonorganic psychoses | schizophrenia spectrum |
| 294910016 | Other paranoid states | schizophrenia spectrum |
| 294912012 | Other paranoid states NOS | schizophrenia spectrum |
| 294926015 | Other reactive psychoses | schizophrenia spectrum |
| 294929010 | Other reactive psychoses NOS | schizophrenia spectrum |
| 401764014 | Other schizophrenia | schizophrenia spectrum |
| 294789017 | Other schizophrenia NOS | schizophrenia spectrum |
| 1975881000006119 | Other schizophrenia, complete remission | schizophrenia spectrum |
| 1975751000006116 | Other schizophrenia, continuous | schizophrenia spectrum |
| 1975911000006119 | Other schizophrenia, course uncertain, period of observation too short | schizophrenia spectrum |
| 1975831000006115 | Other schizophrenia, episodic remittent | schizophrenia spectrum |
| 1975771000006114 | Other schizophrenia, episodic with progressive deficit | schizophrenia spectrum |
| 1975801000006111 | Other schizophrenia, episodic with stable deficit | schizophrenia spectrum |
| 1975841000006113 | Other schizophrenia, incomplete remission | schizophrenia spectrum |
| 1976951000006113 | Other schizophrenia, other | schizophrenia spectrum |
| 294949018 | Other specified non-organic psychoses | schizophrenia spectrum |
| 294911017 | Paranoia querulans | schizophrenia spectrum |
| 2548181016 | Paranoid ideation | schizophrenia spectrum |
| 294913019 | Paranoid psychosis NOS | schizophrenia spectrum |
| 107878010 | Paranoid schizophrenia | schizophrenia spectrum |
| 294760019 | Paranoid schizophrenia NOS | schizophrenia spectrum |
| 105029017 | Paranoid schizophrenia in remission | schizophrenia spectrum |
| 1974511000006113 | Paranoid schizophrenia, complete remission | schizophrenia spectrum |
| 1974351000006118 | Paranoid schizophrenia, continuous | schizophrenia spectrum |
| 1974541000006112 | Paranoid schizophrenia, course uncertain, period of observation too short | schizophrenia spectrum |
| 1974451000006113 | Paranoid schizophrenia, episodic remittent | schizophrenia spectrum |
| 1974391000006112 | Paranoid schizophrenia, episodic with progressive deficit | schizophrenia spectrum |
| 1974431000006118 | Paranoid schizophrenia, episodic with stable deficit | schizophrenia spectrum |
| 3527411000006119 | Paranoid schizophrenia, in remission | schizophrenia spectrum |
| 1974481000006117 | Paranoid schizophrenia, incomplete remission | schizophrenia spectrum |
| 1976881000006112 | Paranoid schizophrenia, other | schizophrenia spectrum |
| 243361000006117 | Paranoid states | schizophrenia spectrum |
| 44335019 | Paraphrenia | schizophrenia spectrum |
| 3555731000006114 | Paraphrenic schizophrenia | schizophrenia spectrum |
| 215691000006112 | Poor insight into psychotic condition | schizophrenia spectrum |
| 5023901000006110 | Post-schizophrenic depression | schizophrenia spectrum |
| 1975301000006116 | Post-schizophrenic depression, complete remission | schizophrenia spectrum |
| 1975191000006114 | Post-schizophrenic depression, continuous | schizophrenia spectrum |
| 1975321000006114 | Post-schizophrenic depression, course uncertain, period of observation too short | schizophrenia spectrum |
| 1975261000006113 | Post-schizophrenic depression, episodic remittent | schizophrenia spectrum |
| 1975211000006110 | Post-schizophrenic depression, episodic with progressive deficit | schizophrenia spectrum |
| 1975231000006116 | Post-schizophrenic depression, episodic with stable deficit | schizophrenia spectrum |
| 1975281000006115 | Post-schizophrenic depression, incomplete remission | schizophrenia spectrum |
| 1976921000006116 | Post-schizophrenic depression, other | schizophrenia spectrum |
| 294924017 | Psychogenic paranoid psychosis | schizophrenia spectrum |
| 294933015 | Psychoses with origin in childhood | schizophrenia spectrum |
| 492881000000114 | Psychosis resolved | schizophrenia spectrum |
| 996171000006114 | Psychosis, schizophrenia + bipolar affective disord resolved | schizophrenia spectrum |
| 402504012 | Psychotic condition, insight present | schizophrenia spectrum |
| 346895011 | Psychotic episode NOS | schizophrenia spectrum |
| 294918011 | Psychotic reactive depression | schizophrenia spectrum |
| 294920014 | Reactive confusion | schizophrenia spectrum |
| 346896012 | Reactive psychoses | schizophrenia spectrum |
| 300931000000113 | Referral to psychosis early intervention service | schizophrenia spectrum |
| 294939016 | Residual disintegrative psychoses | schizophrenia spectrum |
| 43595011 | Residual schizophrenia | schizophrenia spectrum |
| 1975471000006119 | Residual schizophrenia, complete remission | schizophrenia spectrum |
| 1975351000006117 | Residual schizophrenia, continuous | schizophrenia spectrum |
| 1975481000006116 | Residual schizophrenia, course uncertain, period of observation too short | schizophrenia spectrum |
| 1975431000006117 | Residual schizophrenia, episodic remittent | schizophrenia spectrum |
| 1975381000006113 | Residual schizophrenia, episodic with progressive deficit | schizophrenia spectrum |
| 1975401000006113 | Residual schizophrenia, episodic with stable deficit | schizophrenia spectrum |
| 1975441000006110 | Residual schizophrenia, incomplete remission | schizophrenia spectrum |
| 1976931000006118 | Residual schizophrenia, other | schizophrenia spectrum |
| 169061000006112 | Restzustand - schizophrenia | schizophrenia spectrum |
| 155141000006116 | Schizo-affective schizophrenia | schizophrenia spectrum |
| 155161000006117 | Schizo-affective schizophrenia NOS | schizophrenia spectrum |
| 155151000006119 | Schizo-affective schizophrenia in remission | schizophrenia spectrum |
| 4763941000006113 | Schizoaffective schizophrenia, in remission | schizophrenia spectrum |
| 294790014 | Schizophrenia NOS | schizophrenia spectrum |
| 4538451000006117 | Schizophrenia assoc'n member | schizophrenia spectrum |
| 9225016 | Schizophrenia in remission | schizophrenia spectrum |
| 492821000000113 | Schizophrenia resolved | schizophrenia spectrum |
| 294726014 | Schizophrenia simplex | schizophrenia spectrum |
| 4763691000006113 | Schizophrenia, catatonic | schizophrenia spectrum |
| 4196171000006118 | Schizophrenia, catatonic, in remission | schizophrenia spectrum |
| 2577941000006118 | Schizophrenia, in remission | schizophrenia spectrum |
| 4763951000006110 | Schizophrenia, schizoaffective, in remission | schizophrenia spectrum |
| 1976151000006111 | Schizophrenia, unspecified, complete remission | schizophrenia spectrum |
| 1976001000006113 | Schizophrenia, unspecified, continuous | schizophrenia spectrum |
| 1976171000006118 | Schizophrenia, unspecified, course uncertain, period of observation too short | schizophrenia spectrum |
| 1976121000006119 | Schizophrenia, unspecified, episodic remittent | schizophrenia spectrum |
| 1976061000006114 | Schizophrenia, unspecified, episodic with progressive deficit | schizophrenia spectrum |
| 1976101000006112 | Schizophrenia, unspecified, episodic with stable deficit | schizophrenia spectrum |
| 1976141000006114 | Schizophrenia, unspecified, incomplete remission | schizophrenia spectrum |
| 1976961000006110 | Schizophrenia, unspecified, other | schizophrenia spectrum |
| 294725013 | Schizophrenic disorders | schizophrenia spectrum |
| 4763701000006113 | Schizophrenic flexibilatis cerea | schizophrenia spectrum |
| 5248961000006113 | Schizophrenic prodrome | schizophrenia spectrum |
| 882291000006111 | Schizophrenic psychoses | schizophrenia spectrum |
| 882281000006113 | Schizophrenic psychoses NOS | schizophrenia spectrum |
| 3878611000006116 | Schizophreniform psychosis, depressive type | schizophrenia spectrum |
| 5524571000006112 | Schizophreniform psychosis, manic type | schizophrenia spectrum |
| 294909014 | Shared paranoid disorder | schizophrenia spectrum |
| 294904016 | Simple paranoid state | schizophrenia spectrum |
| 294727017 | Simple schizophrenia | schizophrenia spectrum |
| 294734015 | Simple schizophrenia NOS | schizophrenia spectrum |
| 1975601000006110 | Simple schizophrenia, complete remission | schizophrenia spectrum |
| 1975491000006118 | Simple schizophrenia, continuous | schizophrenia spectrum |
| 1975641000006112 | Simple schizophrenia, course uncertain, period of observation too short | schizophrenia spectrum |
| 1975551000006113 | Simple schizophrenia, episodic remittent | schizophrenia spectrum |
| 1975501000006114 | Simple schizophrenia, episodic with progressive deficit | schizophrenia spectrum |
| 1975521000006116 | Simple schizophrenia, episodic with stable deficit | schizophrenia spectrum |
| 1975571000006115 | Simple schizophrenia, incomplete remission | schizophrenia spectrum |
| 1976941000006111 | Simple schizophrenia, other | schizophrenia spectrum |
| 71539017 | Subchronic catatonic schizophrenia | schizophrenia spectrum |
| 4763731000006117 | Subchronic catatonic schizophrenia with acute exacerbation | schizophrenia spectrum |
| 2938951000006113 | Subchronic disorganised schizophrenia | schizophrenia spectrum |
| 2726261000006113 | Subchronic disorganised schizophrenia with acute exacerbations | schizophrenia spectrum |
| 2938961000006110 | Subchronic disorganized schizophrenia | schizophrenia spectrum |
| 2726271000006118 | Subchronic disorganized schizophrenia with acute exacerbations | schizophrenia spectrum |
| 294736018 | Subchronic hebephrenic schizophrenia | schizophrenia spectrum |
| 294766013 | Subchronic latent schizophrenia | schizophrenia spectrum |
| 132503015 | Subchronic paranoid schizophrenia | schizophrenia spectrum |
| 4763771000006119 | Subchronic paranoid schizophrenia with acute exacerbation | schizophrenia spectrum |
| 123611000006110 | Subchronic schizo-affective schizophrenia | schizophrenia spectrum |
| 4763891000006112 | Subchronic schizoaffective schizophrenia | schizophrenia spectrum |
| 28758018 | Subchronic schizophrenia | schizophrenia spectrum |
| 4196151000006111 | Subchronic schizophrenia with acute exacerbations | schizophrenia spectrum |
| 1975081000006110 | Undifferentiated schizophrenia, complete remission | schizophrenia spectrum |
| 1974951000006119 | Undifferentiated schizophrenia, continuous | schizophrenia spectrum |
| 1975161000006118 | Undifferentiated schizophrenia, course uncertain, period of observation too short | schizophrenia spectrum |
| 1975021000006111 | Undifferentiated schizophrenia, episodic remittent | schizophrenia spectrum |
| 1974971000006112 | Undifferentiated schizophrenia, episodic with progressive deficit | schizophrenia spectrum |
| 1975001000006118 | Undifferentiated schizophrenia, episodic with stable deficit | schizophrenia spectrum |
| 1975051000006119 | Undifferentiated schizophrenia, incomplete remission | schizophrenia spectrum |
| 1976911000006112 | Undifferentiated schizophrenia, other | schizophrenia spectrum |
| 294898011 | Unspecified affective psychoses NOS | schizophrenia spectrum |
| 294744018 | Unspecified catatonic schizophrenia | schizophrenia spectrum |
| 294735019 | Unspecified hebephrenic schizophrenia | schizophrenia spectrum |
| 294765012 | Unspecified latent schizophrenia | schizophrenia spectrum |
| 294754019 | Unspecified paranoid schizophrenia | schizophrenia spectrum |
| 78531000006116 | Unspecified schizo-affective schizophrenia | schizophrenia spectrum |
| 294728010 | Unspecified schizophrenia | schizophrenia spectrum |
| 499799011 | Visual hallucinations | schizophrenia spectrum |
| 317003012 | [D]Hallucinations | schizophrenia spectrum |
| 317009011 | [D]Hallucinations NOS | schizophrenia spectrum |
| 317004018 | [D]Hallucinations, auditory | schizophrenia spectrum |
| 317005017 | [D]Hallucinations, gustatory | schizophrenia spectrum |
| 317006016 | [D]Hallucinations, olfactory | schizophrenia spectrum |
| 317007013 | [D]Hallucinations, tactile | schizophrenia spectrum |
| 317008015 | [D]Visual hallucinations | schizophrenia spectrum |
| 460273017 | [V]Personal history of schizophrenia | schizophrenia spectrum |
| 359091000006115 | [X] Presenile psychosis NOS | schizophrenia spectrum |
| 401860014 | [X]Acute and transient psychotic disorder, unspecified | schizophrenia spectrum |
| 362271000006110 | [X]Acute and transient psychotic disorders | schizophrenia spectrum |
| 362381000006115 | [X]Acute polymorphic psychot disord with symp of schizophren | schizophrenia spectrum |
| 362391000006117 | [X]Acute polymorphic psychot disord without symp of schizoph | schizophrenia spectrum |
| 362421000006113 | [X]Acute schizophrenia-like psychotic disorder | schizophrenia spectrum |
| 366571000006114 | [X]Atypical schizophrenia | schizophrenia spectrum |
| 367951000006113 | [X]Borderline schizophrenia | schizophrenia spectrum |
| 367961000006110 | [X]Bouffee delirante | schizophrenia spectrum |
| 367971000006115 | [X]Bouffee delirante with symptoms of schizophrenia | schizophrenia spectrum |
| 367991000006119 | [X]Brief reactive psychosis NOS | schizophrenia spectrum |
| 368001000006112 | [X]Brief schizophreniform disorder | schizophrenia spectrum |
| 368011000006110 | [X]Brief schizophrenifrm psych | schizophrenia spectrum |
| 914461000006118 | [X]Capgras syndrome | schizophrenia spectrum |
| 370451000006110 | [X]Catatonic schizophrenia | schizophrenia spectrum |
| 370461000006112 | [X]Catatonic stupor | schizophrenia spectrum |
| 370631000006117 | [X]Cenesthopathic schizophrenia | schizophrenia spectrum |
| 370981000006112 | [X]Chronic hallucinatory psychosis | schizophrenia spectrum |
| 371031000006115 | [X]Chronic undifferentiated schizophrenia | schizophrenia spectrum |
| 967791000006111 | [X]Cotard syndrome | schizophrenia spectrum |
| 376251000006112 | [X]Cyclic schizophrenia | schizophrenia spectrum |
| 376271000006119 | [X]Cycloid psychosis | schizophrenia spectrum |
| 376281000006116 | [X]Cycloid psychosis with symptoms of schizophrenia | schizophrenia spectrum |
| 215841000000114 | [X]Delusional disorder | schizophrenia spectrum |
| 376501000006110 | [X]Delusional dysmorphophobia | schizophrenia spectrum |
| 914471000006113 | [X]Delusional misidentification syndrome | schizophrenia spectrum |
| 377461000006113 | [X]Disintegrative psychosis | schizophrenia spectrum |
| 378051000006119 | [X]Disorganised schizophrenia | schizophrenia spectrum |
| 387071000006117 | [X]Folie a deux | schizophrenia spectrum |
| 388741000006110 | [X]Hebephrenic schizophrenia | schizophrenia spectrum |
| 389451000006110 | [X]Hysterical psychosis | schizophrenia spectrum |
| 389641000006110 | [X]Induced delusional disorder | schizophrenia spectrum |
| 389651000006112 | [X]Induced paranoid disorder | schizophrenia spectrum |
| 389661000006114 | [X]Induced psychotic disorder | schizophrenia spectrum |
| 394461000006110 | [X]Involutional paranoid state | schizophrenia spectrum |
| 395021000006111 | [X]Latent schizophrenia | schizophrenia spectrum |
| 395031000006114 | [X]Latent schizophrenic reaction | schizophrenia spectrum |
| 398631000006113 | [X]Mixed schizophrenic and affective psychosis | schizophrenia spectrum |
| 1667591000000111 | [X]Nonorganic psychosis in remission | schizophrenia spectrum |
| 403961000006113 | [X]Organic delusional [schizophrenia-like] disorder | schizophrenia spectrum |
| 296083012 | [X]Other acute and transient psychotic disorders | schizophrenia spectrum |
| 401859016 | [X]Other acute predominantly delusional psychotic disorders | schizophrenia spectrum |
| 318027012 | [X]Other hallucinations | schizophrenia spectrum |
| 412201000006113 | [X]Other nonorganic psychotic disorders | schizophrenia spectrum |
| 401857019 | [X]Other persistent delusional disorders | schizophrenia spectrum |
| 296095012 | [X]Other schizoaffective disorders | schizophrenia spectrum |
| 401855010 | [X]Other schizophrenia | schizophrenia spectrum |
| 215871000000115 | [X]Paranoia | schizophrenia spectrum |
| 418181000006110 | [X]Paranoia querulans | schizophrenia spectrum |
| 215851000000112 | [X]Paranoid psychosis | schizophrenia spectrum |
| 418221000006118 | [X]Paranoid schizophrenia | schizophrenia spectrum |
| 418231000006115 | [X]Paranoid state | schizophrenia spectrum |
| 1667581000000114 | [X]Paranoid state in remission | schizophrenia spectrum |
| 418251000006110 | [X]Paraphrenia - late | schizophrenia spectrum |
| 418261000006112 | [X]Paraphrenic schizophrenia | schizophrenia spectrum |
| 296066015 | [X]Persistent delusional disorder, unspecified | schizophrenia spectrum |
| 419861000006117 | [X]Persistent delusional disorders | schizophrenia spectrum |
| 423041000006119 | [X]Post-schizophrenic depression | schizophrenia spectrum |
| 423271000006116 | [X]Prepsychotic schizophrenia | schizophrenia spectrum |
| 423471000006117 | [X]Prodromal schizophrenia | schizophrenia spectrum |
| 423731000006114 | [X]Pseudoneurotic schizophrenia | schizophrenia spectrum |
| 423741000006116 | [X]Pseudopsychopathic schizophrenia | schizophrenia spectrum |
| 424071000006112 | [X]Psychogenic paranoid psychosis | schizophrenia spectrum |
| 424231000006116 | [X]Psychosis NOS | schizophrenia spectrum |
| 424281000006115 | [X]Puerperal psychosis NOS | schizophrenia spectrum |
| 424511000006112 | [X]Reactive psychosis | schizophrenia spectrum |
| 424841000006113 | [X]Residual schizophrenia | schizophrenia spectrum |
| 424931000006113 | [X]Restzustand schizophrenic | schizophrenia spectrum |
| 425501000006113 | [X]Schizoaffective disorder, depressive type | schizophrenia spectrum |
| 425511000006111 | [X]Schizoaffective disorder, manic type | schizophrenia spectrum |
| 425521000006115 | [X]Schizoaffective disorder, mixed type | schizophrenia spectrum |
| 296096013 | [X]Schizoaffective disorder, unspecified | schizophrenia spectrum |
| 425541000006110 | [X]Schizoaffective disorders | schizophrenia spectrum |
| 425551000006112 | [X]Schizoaffective psychosis NOS | schizophrenia spectrum |
| 425561000006114 | [X]Schizoaffective psychosis, depressive type | schizophrenia spectrum |
| 425571000006119 | [X]Schizoaffective psychosis, manic type | schizophrenia spectrum |
| 425601000006114 | [X]Schizophrenia | schizophrenia spectrum |
| 12480451000006112 | [X]Schizophrenia, schizotypal and delusional disorders | schizophrenia spectrum |
| 296022017 | [X]Schizophrenia, schizotypal and delusional disorders | schizophrenia spectrum |
| 296040018 | [X]Schizophrenia, unspecified | schizophrenia spectrum |
| 425641000006111 | [X]Schizophrenic catalepsy | schizophrenia spectrum |
| 425651000006113 | [X]Schizophrenic catatonia | schizophrenia spectrum |
| 294743012 | [X]Schizophrenic flexibilatis cerea | schizophrenia spectrum |
| 425671000006115 | [X]Schizophrenic reaction | schizophrenia spectrum |
| 425681000006117 | [X]Schizophreniform disord NOS | schizophrenia spectrum |
| 425691000006119 | [X]Schizophreniform psychosis, depressive type | schizophrenia spectrum |
| 425701000006119 | [X]Schizophreniform psychosis, manic type | schizophrenia spectrum |
| 425711000006116 | [X]Schizophrenifrm psychos NOS | schizophrenia spectrum |
| 401856011 | [X]Schizotypal disorder | schizophrenia spectrum |
| 425921000006114 | [X]Sensitiver Beziehungswahn | schizophrenia spectrum |
| 426881000006111 | [X]Simple schizophrenia | schizophrenia spectrum |
| 428441000006116 | [X]Symbiotic psychosis | schizophrenia spectrum |
| 428451000006119 | [X]Symptomatic psychosis NOS | schizophrenia spectrum |
| 296031017 | [X]Undifferentiated schizophrenia | schizophrenia spectrum |
| 401862018 | [X]Unspecified nonorganic psychosis | schizophrenia spectrum |
| 295427011 | 'Short-sleeper' | sleep disorders |
| 3191221000006119 | Acute sleeping sickness | sleep disorders |
| 3593421000006114 | Broken sleep | sleep disorders |
| 407067015 | C/O - insomnia | sleep disorders |
| 252940010 | C/O nightmares | sleep disorders |
| 8058301000006110 | COPD (chronic obstructive pulmonary disease) disturbs sleep | sleep disorders |
| 2939291000006118 | CSA - Central sleep apnea | sleep disorders |
| 2939271000006119 | CSA - Central sleep apnoea | sleep disorders |
| 3459351000006112 | Cannot get off to sleep | sleep disorders |
| 217721000000117 | Cannot sleep - insomnia | sleep disorders |
| 77147016 | Cataplexy | sleep disorders |
| 297357017 | Cataplexy and narcolepsy | sleep disorders |
| 297358010 | Cataplexy or narcolepsy NOS | sleep disorders |
| 2939301000006117 | Central sleep apnea | sleep disorders |
| 2939261000006114 | Central sleep apnea syndrome | sleep disorders |
| 1772651000006110 | Central sleep apnoea | sleep disorders |
| 2939251000006112 | Central sleep apnoea syndrome | sleep disorders |
| 299001000000116 | Chronic obstructive pulmonary disease disturbs sleep | sleep disorders |
| 3732591000006114 | Chronic sleeping sickness | sleep disorders |
| 2558681000006115 | Circadian rhythm sleep disorder | sleep disorders |
| 5008741000006111 | Circadian rhythm sleep disorder, free-running type | sleep disorders |
| 841621000006110 | Delayed Onset Of Sleep | sleep disorders |
| 1780361011 | Delayed onset of sleep | sleep disorders |
| 917101000006119 | Delayed onset of sleep | sleep disorders |
| 3459321000006115 | Difficulty falling asleep | sleep disorders |
| 3459341000006110 | Difficulty getting to sleep | sleep disorders |
| 3459331000006117 | Difficulty in sleep initiation | sleep disorders |
| 3593451000006117 | Difficulty in sleep maintenance | sleep disorders |
| 1806241000006116 | Difficulty sleeping | sleep disorders |
| 3593391000006118 | Difficulty staying asleep | sleep disorders |
| 5529451000006119 | Disorder of sleep-wake cycle | sleep disorders |
| 5529491000006113 | Disorder of sleep-wake schedule | sleep disorders |
| 299274011 | Disorders of initiating and maintaining sleep | sleep disorders |
| 406739012 | Disorders of the sleep-wake schedule | sleep disorders |
| 1234153012 | Excessive sleep | sleep disorders |
| 3764071000006116 | Excessive sleepiness | sleep disorders |
| 5008721000006116 | Free-running sleep-wake cycle disorder | sleep disorders |
| 1994261000006118 | History of rough sleeping | sleep disorders |
| 1772801000006112 | Hypersomnia | sleep disorders |
| 3764081000006118 | Hypersomnia (excessive sleeping) | sleep disorders |
| 345379011 | Hypersomnia NOS | sleep disorders |
| 345382018 | Hypersomnia of non-organic origin | sleep disorders |
| 3789311000006111 | Hypersomnia with sleep apnea | sleep disorders |
| 5008591000006110 | Hypersomnia, nonorganic | sleep disorders |
| 1772821000006119 | Idiopathic hypersomnia associated with long sleep time | sleep disorders |
| 7263971000006116 | Idiopathic hypersomnia with long sleep time | sleep disorders |
| 1772831000006116 | Idiopathic hypersomnia without long sleep time | sleep disorders |
| 2562371000006117 | Idiopathic insomnia | sleep disorders |
| 98133013 | Initial insomnia | sleep disorders |
| 960621000006117 | Insomnia | sleep disorders |
| 345377013 | Insomnia NOS | sleep disorders |
| 772561000006111 | Insomnia due to nonorganic sleep disorder | sleep disorders |
| 3176551000006111 | Insomnia with sleep apnea | sleep disorders |
| 960641000006112 | Insufficient sleep/rest for age/physical condition | sleep disorders |
| 3593431000006112 | Interrupted sleep | sleep disorders |
| 295430016 | Inversion of sleep rhythm | sleep disorders |
| 252935019 | Late insomnia | sleep disorders |
| 49145012 | Light sleep | sleep disorders |
| 305092019 | Menopausal sleeplessness | sleep disorders |
| 111701012 | Middle insomnia | sleep disorders |
| 100304016 | Narcolepsy | sleep disorders |
| 3480591000006112 | Narcoleptic syndrome | sleep disorders |
| 675161000006119 | Nightmares | sleep disorders |
| 252939013 | Nightmares - symptom | sleep disorders |
| 5506811000006116 | Non-organic disorder of the sleep-wake schedule | sleep disorders |
| 295435014 | Non-organic sleep disorder NOS | sleep disorders |
| 223701000000114 | Non-organic sleep disorders | sleep disorders |
| 3773281000006119 | OSA - Obstructive sleep apnea | sleep disorders |
| 3773271000006117 | OSA - Obstructive sleep apnoea | sleep disorders |
| 3773291000006116 | Obstructive sleep apnea | sleep disorders |
| 3773261000006112 | Obstructive sleep apnea syndrome | sleep disorders |
| 267911000006119 | Obstructive sleep apnoea | sleep disorders |
| 219501000000111 | Obstructive sleep apnoea | sleep disorders |
| 3773251000006110 | Obstructive sleep apnoea syndrome | sleep disorders |
| 2287351000000110 | On melatonin for sleep disorder | sleep disorders |
| 401808017 | Other non-organic sleep disorder | sleep disorders |
| 295423010 | Other sleep stage or arousal dysfunction | sleep disorders |
| 1820971000006110 | PCL-C - Trouble falling or staying asleep: A little bit | sleep disorders |
| 1821001000006112 | PCL-C - Trouble falling or staying asleep: Extremely | sleep disorders |
| 1820981000006113 | PCL-C - Trouble falling or staying asleep: Moderately | sleep disorders |
| 1820991000006111 | PCL-C - Trouble falling or staying asleep: Quite a bit | sleep disorders |
| 3453391000006116 | Parasomnia (sleep disorder) | sleep disorders |
| 3480571000006111 | Paroxysmal sleep | sleep disorders |
| 1772781000006113 | Periodic leg movements of sleep | sleep disorders |
| 295416014 | Persistent hypersomnia | sleep disorders |
| 295412011 | Persistent insomnia | sleep disorders |
| 3887451000006112 | Poisoning by sleeping drug | sleep disorders |
| 3887461000006114 | Poisoning by sleeping pill | sleep disorders |
| 3887471000006119 | Poisoning caused by sleeping drug | sleep disorders |
| 3887491000006118 | Poisoning caused by sleeping pill | sleep disorders |
| 3887481000006116 | Poisoning caused by sleeping tablet | sleep disorders |
| 5892471000006112 | Poor sleep | sleep disorders |
| 459355013 | Poor sleep pattern | sleep disorders |
| 1772811000006110 | Primary hypersomnia | sleep disorders |
| 1806231000006114 | Primary insomnia | sleep disorders |
| 1806271000006112 | Psycho-education - sleep | sleep disorders |
| 6872511000006113 | REM sleep behavior disorder | sleep disorders |
| 1772851000006111 | REM sleep behaviour disorder | sleep disorders |
| 6872521000006117 | REM sleep disorder | sleep disorders |
| 6872531000006119 | Rapid eye movement sleep behavior disorder | sleep disorders |
| 6872541000006112 | Rapid eye movement sleep behaviour disorder | sleep disorders |
| 1856301000006117 | Rcp difficulty sleeping | sleep disorders |
| 1777001000006116 | Reason for referral: Sleep Problems | sleep disorders |
| 11639631000006119 | Recommendation to use digital cognitive behavioural therapy for insomnia | sleep disorders |
| 1951091000006113 | Referral to rough sleeper outreach team | sleep disorders |
| 634561000000115 | Referral to sleep clinic | sleep disorders |
| 295422017 | Repeated rapid eye movement sleep interruptions | sleep disorders |
| 401807010 | Repetitive intrusions of sleep | sleep disorders |
| 5506631000006111 | Repetitive sleep intrusions | sleep disorders |
| 54936019 | Restless legs syndrome | sleep disorders |
| 21110011 | Restless sleep | sleep disorders |
| 4766561000006111 | Reversed sleep-wake cycle | sleep disorders |
| 3693971000006113 | SAS - Sleep apnea syndrome | sleep disorders |
| 3694001000006117 | SAS - Sleep apnoea syndrome | sleep disorders |
| 2694806018 | Seen in sleep clinic | sleep disorders |
| 981761000006114 | Sleep apnea | sleep disorders |
| 3693961000006118 | Sleep apnea | sleep disorders |
| 3847221000006110 | Sleep apnea monitoring with alarm | sleep disorders |
| 3693991000006114 | Sleep apnea syndrome | sleep disorders |
| 200354014 | Sleep apnoea | sleep disorders |
| 136581000006114 | Sleep apnoea | sleep disorders |
| 3847211000006119 | Sleep apnoea monitoring with alarm | sleep disorders |
| 11989401000006115 | Sleep apnoea syndrome | sleep disorders |
| 12702791000006116 | Sleep apnoea syndrome | sleep disorders |
| 136601000006116 | Sleep disorders | sleep disorders |
| 643141000000111 | Sleep hygiene behaviour education | sleep disorders |
| 3693981000006111 | Sleep hypopnea | sleep disorders |
| 3694011000006119 | Sleep hypopnoea | sleep disorders |
| 1488768014 | Sleep management | sleep disorders |
| 1772791000006111 | Sleep paralysis | sleep disorders |
| 6649641000006111 | Sleep pattern | sleep disorders |
| 3211751000006118 | Sleep problem | sleep disorders |
| 7282631000006117 | Sleep related hypoventilation | sleep disorders |
| 4766571000006116 | Sleep rhythm inversion | sleep disorders |
| 2558691000006117 | Sleep rhythm problem | sleep disorders |
| 3957671000006117 | Sleep terror disorder | sleep disorders |
| 3957681000006119 | Sleep terrors | sleep disorders |
| 3809191000006116 | Sleep walking | sleep disorders |
| 3809171000006117 | Sleep walking disorder | sleep disorders |
| 5008031000006115 | Sleep-related epilepsy | sleep disorders |
| 345396012 | Sleep-related respiratory failure | sleep disorders |
| 2558671000006118 | Sleep-wake schedule disorder | sleep disorders |
| 5529331000006119 | Sleepiness | sleep disorders |
| 841611000006119 | Sleeping Pattern | sleep disorders |
| 503961011 | Sleeping sickness | sleep disorders |
| 2932791000006119 | Sleeping sickness | sleep disorders |
| 4771851000006111 | Sleeplessness | sleep disorders |
| 3764061000006111 | Sleeps too much | sleep disorders |
| 960611000006113 | Sleepwalking | sleep disorders |
| 3809201000006118 | Sleepwalking disorder | sleep disorders |
| 5529361000006111 | Sleepy | sleep disorders |
| 139641000006114 | Somnambulism - sleep walking | sleep disorders |
| 5529351000006114 | Somnolence (sleepiness) | sleep disorders |
| 2695301000006113 | Tosses and turns in sleep | sleep disorders |
| 2695291000006112 | Tossing and turning during sleep | sleep disorders |
| 401806018 | Transient hypersomnia | sleep disorders |
| 401805019 | Transient insomnia | sleep disorders |
| 295408017 | Unspecified non-organic sleep disorder | sleep disorders |
| 317048011 | [D]Hypersomnia NOS | sleep disorders |
| 405691012 | [D]Hypersomnia with sleep apnoea | sleep disorders |
| 1222474014 | [D]Insomnia - symptom | sleep disorders |
| 317035012 | [D]Insomnia NOS | sleep disorders |
| 317034011 | [D]Insomnia with sleep apnoea | sleep disorders |
| 457106016 | [D]Sleep apnoea syndrome | sleep disorders |
| 317032010 | [D]Sleep disturbance, unspecified | sleep disorders |
| 317031015 | [D]Sleep disturbances | sleep disorders |
| 317054012 | [D]Sleep dysfunction NOS | sleep disorders |
| 317053018 | [D]Sleep dysfunction with arousal disturbance | sleep disorders |
| 317052011 | [D]Sleep dysfunction with sleep stage disturbance | sleep disorders |
| 317049015 | [D]Sleep rhythm inversion | sleep disorders |
| 317050015 | [D]Sleep rhythm irregular | sleep disorders |
| 1222473015 | [D]Sleep rhythm problems | sleep disorders |
| 317051016 | [D]Sleep-wake rhythm non-24-hour cycle | sleep disorders |
| 303181000006112 | [D]Syndrome sleep apnoea | sleep disorders |
| 460422010 | [V]Personal history of unhealthy sleep-wake schedule | sleep disorders |
| 357711000006119 | [X] Adverse reaction to sleeping pill NOS | sleep disorders |
| 334363016 | [X]Accidental poisoning with sleeping tablets | sleep disorders |
| 379691000006110 | [X]Emotional sleep disorder NOS | sleep disorders |
| 399991000006114 | [X]Nightmares | sleep disorders |
| 400071000006111 | [X]Nonorganic disorder of the sleep-wake schedule | sleep disorders |
| 400111000006115 | [X]Nonorganic hypersomnia | sleep disorders |
| 400121000006111 | [X]Nonorganic insomnia | sleep disorders |
| 296396019 | [X]Nonorganic sleep disorder, unspecified | sleep disorders |
| 400141000006116 | [X]Nonorganic sleep disorders | sleep disorders |
| 296394016 | [X]Other nonorganic sleep disorders | sleep disorders |
| 299344018 | [X]Other sleep disorders | sleep disorders |
| 334643017 | [X]Overdose - sleeping tabs | sleep disorders |
| 424021000006111 | [X]Psychogenic inversion of circadian rhythm | sleep disorders |
| 424041000006116 | [X]Psychogenic inversion of sleep rhythm | sleep disorders |
| 427011000006112 | [X]Sleep terrors | sleep disorders |
| 427021000006116 | [X]Sleepwalking | sleep disorders |
| 17390014 | Acute alcoholic hepatitis | substance misuse |
| 295127018 | Acute alcoholic intoxication in alcoholism | substance misuse |
| 295132017 | Acute alcoholic intoxication in alcoholism NOS | substance misuse |
| 295131012 | Acute alcoholic intoxication in remission, in alcoholism | substance misuse |
| 295128011 | Acute alcoholic intoxication, unspecified, in alcoholism | substance misuse |
| 1550291000000111 | Admission to substance misuse detoxification centre | substance misuse |
| 405461000000114 | Alcohol abuse monitoring | substance misuse |
| 476361000006114 | Alcohol amnestic syndrome | substance misuse |
| 294664016 | Alcohol amnestic syndrome NOS | substance misuse |
| 500478011 | Alcohol dependence syndrome | substance misuse |
| 295144019 | Alcohol dependence syndrome NOS | substance misuse |
| 295126010 | Alcohol dependence with acute alcoholic intoxication | substance misuse |
| 106878015 | Alcohol detoxification | substance misuse |
| 2474678017 | Alcohol disorder monitoring | substance misuse |
| 2549849017 | Alcohol induced hallucinations | substance misuse |
| 606991000000117 | Alcohol misuse | substance misuse |
| 15243013 | Alcohol withdrawal delirium | substance misuse |
| 294669014 | Alcohol withdrawal hallucinosis | substance misuse |
| 294674018 | Alcohol withdrawal syndrome | substance misuse |
| 353698013 | Alcohol-induced chronic pancreatitis | substance misuse |
| 478024019 | Alcohol-induced epilepsy | substance misuse |
| 356292013 | Alcohol-induced pseudo-Cushing's syndrome | substance misuse |
| 138527014 | Alcoholic cardiomyopathy | substance misuse |
| 476611000006116 | Alcoholic cirrhosis of liver | substance misuse |
| 346929012 | Alcoholic dementia NOS | substance misuse |
| 297011014 | Alcoholic encephalopathy | substance misuse |
| 83834016 | Alcoholic fatty liver | substance misuse |
| 353594015 | Alcoholic fibrosis and sclerosis of liver | substance misuse |
| 4525015 | Alcoholic gastritis | substance misuse |
| 353595019 | Alcoholic hepatic failure | substance misuse |
| 353587018 | Alcoholic hepatitis | substance misuse |
| 303399018 | Alcoholic liver damage unspecified | substance misuse |
| 476701000006114 | Alcoholic myopathy | substance misuse |
| 294672019 | Alcoholic paranoia | substance misuse |
| 14103017 | Alcoholic polyneuropathy | substance misuse |
| 476741000006111 | Alcoholic psychoses | substance misuse |
| 294676016 | Alcoholic psychosis NOS | substance misuse |
| 251195015 | Alcoholics anonymous | substance misuse |
| 12878014 | Alcoholism | substance misuse |
| 2475575018 | Amfetamine or psychostimulant dependence NOS | substance misuse |
| 2475571010 | Amfetamine or psychostimulant dependence, unspecified | substance misuse |
| 2478714011 | Amfetamine poisoning | substance misuse |
| 401794015 | Amphetamine or other psychostimulant dependence | substance misuse |
| 295201013 | Amphetamine or psychostimulant dependence NOS | substance misuse |
| 295200014 | Amphetamine or psychostimulant dependence in remission | substance misuse |
| 295198015 | Amphetamine or psychostimulant dependence, continuous | substance misuse |
| 295199011 | Amphetamine or psychostimulant dependence, episodic | substance misuse |
| 295197013 | Amphetamine or psychostimulant dependence, unspecified | substance misuse |
| 1230261015 | Amphetamine poisoning | substance misuse |
| 283451018 | Aversion therapy - alcoholism | substance misuse |
| 346942012 | Benzodiazepine dependence | substance misuse |
| 342366014 | Binge drinker | substance misuse |
| 295190010 | Cannabis dependence in remission | substance misuse |
| 295188014 | Cannabis dependence, continuous | substance misuse |
| 295189018 | Cannabis dependence, episodic | substance misuse |
| 295187016 | Cannabis dependence, unspecified | substance misuse |
| 295191014 | Cannabis drug dependence NOS | substance misuse |
| 401793014 | Cannabis type drug dependence | substance misuse |
| 478046010 | Cerebellar ataxia due to alcoholism | substance misuse |
| 542611000006114 | Cerebral degeneration due to alcoholism | substance misuse |
| 342446016 | Chases the dragon | substance misuse |
| 294668018 | Chronic alcoholic brain syndrome | substance misuse |
| 451124014 | Chronic alcoholic hepatitis | substance misuse |
| 110629012 | Chronic alcoholism | substance misuse |
| 295143013 | Chronic alcoholism NOS | substance misuse |
| 295142015 | Chronic alcoholism in remission | substance misuse |
| 295176012 | Cocaine dependence, continuous | substance misuse |
| 295177015 | Cocaine dependence, episodic | substance misuse |
| 295173016 | Cocaine dependence, unspecified | substance misuse |
| 295179017 | Cocaine drug dependence NOS | substance misuse |
| 571351000006110 | Cocaine poisoning | substance misuse |
| 295172014 | Cocaine type drug dependence | substance misuse |
| 295239017 | Combined drug dependence, excluding opioid, NOS | substance misuse |
| 295236012 | Combined drug dependence, excluding opioid, continuous | substance misuse |
| 295237015 | Combined drug dependence, excluding opioid, episodic | substance misuse |
| 295235011 | Combined drug dependence, excluding opioid, unspecified | substance misuse |
| 295234010 | Combined drug dependence, excluding opioids | substance misuse |
| 295228015 | Combined opioid with other drug dependence | substance misuse |
| 295233016 | Combined opioid with other drug dependence NOS | substance misuse |
| 295230018 | Combined opioid with other drug dependence, continuous | substance misuse |
| 295231019 | Combined opioid with other drug dependence, episodic | substance misuse |
| 295229011 | Combined opioid with other drug dependence, unspecified | substance misuse |
| 295129015 | Continuous acute alcoholic intoxication in alcoholism | substance misuse |
| 295139014 | Continuous chronic alcoholism | substance misuse |
| 295154015 | Continuous opioid dependence | substance misuse |
| 2548235012 | Current drug user | substance misuse |
| 2537051014 | DNA - Did not attend substance misuse clinic | substance misuse |
| 371501000000110 | Delivery of rehabilitation for alcohol addiction | substance misuse |
| 371621000000117 | Delivery of rehabilitation for drug addiction | substance misuse |
| 512661000000111 | Does not use heroin on top of substitution therapy | substance misuse |
| 405581000000116 | Drug abuse monitoring | substance misuse |
| 342438017 | Drug addict | substance misuse |
| 630051000006114 | Drug addict notific admin | substance misuse |
| 630061000006111 | Drug addict notific to CMO | substance misuse |
| 21261000000113 | Drug addict re-notif to CMO | substance misuse |
| 285115018 | Drug addict re-notific due | substance misuse |
| 295146017 | Drug addiction | substance misuse |
| 316931000000112 | Drug addiction detoxification therapy - buprenorphine | substance misuse |
| 302981000000113 | Drug addiction detoxification therapy - methadone | substance misuse |
| 316901000000118 | Drug addiction maintenance therapy - buprenorphine | substance misuse |
| 303011000000111 | Drug addiction maintenance therapy - methadone | substance misuse |
| 285119012 | Drug addiction notif NOS | substance misuse |
| 405031011 | Drug addiction notification | substance misuse |
| 1676621000006110 | Drug addiction notification local SMR22 | substance misuse |
| 397153012 | Drug addiction therapy | substance misuse |
| 630131000006118 | Drug addictn therap-methadone | substance misuse |
| 295145018 | Drug dependence | substance misuse |
| 295241016 | Drug dependence NOS | substance misuse |
| 306144010 | Drug dependence during pregnancy - baby delivered | substance misuse |
| 306146012 | Drug dependence during pregnancy - baby not yet delivered | substance misuse |
| 630311000006110 | Drug dependence during pregnancy/childbirth/puerperium NOS | substance misuse |
| 2533722018 | Drug dependence home detoxification | substance misuse |
| 2533733015 | Drug dependence home detoxification contraindicated | substance misuse |
| 630321000006119 | Drug dependence in pregnancy, childbirth and the puerperium | substance misuse |
| 2533721013 | Drug dependence self detoxification | substance misuse |
| 630361000006113 | Drug dependence therapy | substance misuse |
| 237641000000113 | Drug misuse - enhanced service completed | substance misuse |
| 235741000000110 | Drug misuse - enhanced services administration | substance misuse |
| 1680621000006115 | Drug misuse assessment declined - enhanced services administ | substance misuse |
| 2246691000000115 | Drug misuse clinic administration | substance misuse |
| 1153431000000110 | Drug misuse treatment in primary care | substance misuse |
| 1704471000006116 | Drug misuse treatment primary care - enhanced services admin | substance misuse |
| 630831000006113 | Drug psychoses | substance misuse |
| 294693016 | Drug psychosis NOS | substance misuse |
| 631001000006115 | Drug user | substance misuse |
| 294691019 | Drug-induced personality disorder | substance misuse |
| 342520017 | Drug-related offending behaviour | substance misuse |
| 324297018 | Ecstasy poisoning | substance misuse |
| 295240015 | Ecstasy type drug dependence | substance misuse |
| 295130013 | Episodic acute alcoholic intoxication in alcoholism | substance misuse |
| 295141010 | Episodic chronic alcoholism | substance misuse |
| 295155019 | Episodic opioid dependence | substance misuse |
| 249855011 | FH: Drug dependency | substance misuse |
| 1485129010 | Failed heroin detoxification | substance misuse |
| 2533651012 | Follow up substance misuse assessment | substance misuse |
| 295212013 | Glue sniffing dependence | substance misuse |
| 295218012 | Glue sniffing dependence NOS | substance misuse |
| 295215010 | Glue sniffing dependence, continuous | substance misuse |
| 295216011 | Glue sniffing dependence, episodic | substance misuse |
| 295214014 | Glue sniffing dependence, unspecified | substance misuse |
| 512241000000113 | H/O daily heroin misuse | substance misuse |
| 512421000000117 | H/O daily opiate misuse | substance misuse |
| 512601000000112 | H/O daily solvent misuse | substance misuse |
| 512631000000118 | H/O heroin misuse | substance misuse |
| 512351000000115 | H/O infrequent amphetamine misuse | substance misuse |
| 512471000000118 | H/O infrequent cocaine misuse | substance misuse |
| 512501000000113 | H/O infrequent crack cocaine misuse | substance misuse |
| 512261000000114 | H/O infrequent heroin misuse | substance misuse |
| 512441000000112 | H/O infrequent opiate misuse | substance misuse |
| 512621000000115 | H/O infrequent solvent misuse | substance misuse |
| 2418861000000117 | H/O novel psychoactive substance misuse | substance misuse |
| 512201000000110 | H/O opiate misuse | substance misuse |
| 512171000000114 | H/O solvent misuse | substance misuse |
| 512341000000118 | H/O weekly amphetamine misuse | substance misuse |
| 512461000000113 | H/O weekly cocaine misuse | substance misuse |
| 512491000000119 | H/O weekly crack cocaine misuse | substance misuse |
| 512251000000111 | H/O weekly heroin misuse | substance misuse |
| 512431000000115 | H/O weekly opiate misuse | substance misuse |
| 512611000000114 | H/O weekly solvent misuse | substance misuse |
| 251626014 | H/O: alcoholism | substance misuse |
| 2476701016 | H/O: drug abuse | substance misuse |
| 251627017 | H/O: drug dependency | substance misuse |
| 64025010 | Hallucinogen dependence | substance misuse |
| 295211018 | Hallucinogen dependence NOS | substance misuse |
| 295208019 | Hallucinogen dependence, continuous | substance misuse |
| 295209010 | Hallucinogen dependence, episodic | substance misuse |
| 295207012 | Hallucinogen dependence, unspecified | substance misuse |
| 299081000000114 | Harmful alcohol use | substance misuse |
| 817251000006118 | Hashish dependence | substance misuse |
| 299061000000117 | Hazardous alcohol use | substance misuse |
| 757341000000110 | Health problem secondary to drug misuse | substance misuse |
| 346947018 | Heroin dependence | substance misuse |
| 309921000000119 | Heroin misuse | substance misuse |
| 1220925010 | Heroin poisoning | substance misuse |
| 497451000000119 | History of substance misuse | substance misuse |
| 295169019 | Hypnotic or anxiolytic dependence, episodic | substance misuse |
| 2301851000000116 | In-house substance misuse treatment | substance misuse |
| 2533642016 | Initial substance misuse assessment | substance misuse |
| 339576014 | Injecting drug user | substance misuse |
| 342455018 | Injects drugs subcutaneously | substance misuse |
| 769451000006111 | Intoxication - alcohol | substance misuse |
| 342465012 | Intravenous drug user | substance misuse |
| 753331000006118 | Korsakov's alcoholic psychosis | substance misuse |
| 294662017 | Korsakov's alcoholic psychosis with peripheral neuritis | substance misuse |
| 346936013 | LSD dependence | substance misuse |
| 369851012 | LSD reaction | substance misuse |
| 342434015 | Long-term drug misuser | substance misuse |
| 346935012 | Lysergic acid diethylamide dependence | substance misuse |
| 718011000006110 | Marihuana dependence | substance misuse |
| 346949015 | Methadone dependence | substance misuse |
| 295327010 | Misuse of prescription only drugs | substance misuse |
| 342441014 | Misuses drugs orally | substance misuse |
| 1230527016 | Morphine poisoning | substance misuse |
| 500344018 | Nondependent abuse of drugs | substance misuse |
| 401797010 | Nondependent alcohol abuse | substance misuse |
| 295255012 | Nondependent alcohol abuse NOS | substance misuse |
| 295254011 | Nondependent alcohol abuse in remission | substance misuse |
| 295252010 | Nondependent alcohol abuse, continuous | substance misuse |
| 295253017 | Nondependent alcohol abuse, episodic | substance misuse |
| 295251015 | Nondependent alcohol abuse, unspecified | substance misuse |
| 401800012 | Nondependent amphetamine or other psychostimulant abuse | substance misuse |
| 295314011 | Nondependent amphetamine or psychostimulant abuse NOS | substance misuse |
| 295312010 | Nondependent amphetamine or psychostimulant abuse, episodic | substance misuse |
| 586641000006118 | Nondependent amphetamine/psychostimulant abuse in remission | substance misuse |
| 586651000006116 | Nondependent amphetamine/psychostimulant abuse, continuous | substance misuse |
| 586661000006119 | Nondependent amphetamine/psychostimulant abuse, unspecified | substance misuse |
| 586671000006114 | Nondependent antidepressant type drug abuse | substance misuse |
| 295321011 | Nondependent antidepressant type drug abuse NOS | substance misuse |
| 295261010 | Nondependent cannabis abuse | substance misuse |
| 295266017 | Nondependent cannabis abuse NOS | substance misuse |
| 295263013 | Nondependent cannabis abuse, continuous | substance misuse |
| 295264019 | Nondependent cannabis abuse, episodic | substance misuse |
| 295262015 | Nondependent cannabis abuse, unspecified | substance misuse |
| 295299015 | Nondependent cocaine abuse | substance misuse |
| 295305018 | Nondependent cocaine abuse NOS | substance misuse |
| 295303013 | Nondependent cocaine abuse in remission | substance misuse |
| 295301010 | Nondependent cocaine abuse, continuous | substance misuse |
| 295302015 | Nondependent cocaine abuse, episodic | substance misuse |
| 295300011 | Nondependent cocaine abuse, unspecified | substance misuse |
| 401798017 | Nondependent hallucinogen abuse | substance misuse |
| 295276019 | Nondependent hallucinogen abuse NOS | substance misuse |
| 295275015 | Nondependent hallucinogen abuse in remission | substance misuse |
| 295272017 | Nondependent hallucinogen abuse, continuous | substance misuse |
| 295274016 | Nondependent hallucinogen abuse, episodic | substance misuse |
| 295271012 | Nondependent hallucinogen abuse, unspecified | substance misuse |
| 295322016 | Nondependent mixed drug abuse | substance misuse |
| 295328017 | Nondependent mixed drug abuse NOS | substance misuse |
| 295326018 | Nondependent mixed drug abuse in remission | substance misuse |
| 295324015 | Nondependent mixed drug abuse, continuous | substance misuse |
| 295325019 | Nondependent mixed drug abuse, episodic | substance misuse |
| 295323014 | Nondependent mixed drug abuse, unspecified | substance misuse |
| 295290016 | Nondependent opioid abuse | substance misuse |
| 295298011 | Nondependent opioid abuse NOS | substance misuse |
| 295297018 | Nondependent opioid abuse in remission | substance misuse |
| 295295014 | Nondependent opioid abuse, continuous | substance misuse |
| 295296010 | Nondependent opioid abuse, episodic | substance misuse |
| 295293019 | Nondependent opioid abuse, unspecified | substance misuse |
| 401801011 | Nondependent other drug abuse | substance misuse |
| 295339019 | Nondependent other drug abuse NOS | substance misuse |
| 295338010 | Nondependent other drug abuse in remission | substance misuse |
| 295336014 | Nondependent other drug abuse, continuous | substance misuse |
| 295337017 | Nondependent other drug abuse, episodic | substance misuse |
| 295335013 | Nondependent other drug abuse, unspecified | substance misuse |
| 342439013 | Notified addict | substance misuse |
| 2371251000000114 | Novel psychoactive substance misuse | substance misuse |
| 254306016 | O/E - alcoholic breath | substance misuse |
| 453265010 | Oesophageal varices in alcoholic cirrhosis of the liver | substance misuse |
| 309021000000115 | On substance misuse programme | substance misuse |
| 1495571019 | Opiate and narcotic poisoning | substance misuse |
| 19399016 | Opiate poisoning | substance misuse |
| 541451000000111 | Opioid agonist substitution therapy | substance misuse |
| 541541000000112 | Opioid antagonist therapy | substance misuse |
| 295157010 | Opioid drug dependence NOS | substance misuse |
| 44291000006113 | Opioid type drug dependence | substance misuse |
| 346951016 | Opium dependence | substance misuse |
| 401760017 | Other alcoholic dementia | substance misuse |
| 294673012 | Other alcoholic psychosis | substance misuse |
| 294686015 | Other drug psychoses | substance misuse |
| 294692014 | Other drug psychoses NOS | substance misuse |
| 401796018 | Other specified drug dependence | substance misuse |
| 295227013 | Other specified drug dependence NOS | substance misuse |
| 295224018 | Other specified drug dependence, continuous | substance misuse |
| 295225017 | Other specified drug dependence, episodic | substance misuse |
| 295222019 | Other specified drug dependence, unspecified | substance misuse |
| 294671014 | Pathological alcohol intoxication | substance misuse |
| 2548434014 | Persistent substance misuse | substance misuse |
| 1153411000000119 | Pharmacy attended for drug misuse | substance misuse |
| 1714461000006113 | Pharmacy attended for drug misuse - enhanced services admin | substance misuse |
| 342435019 | Poly-drug misuser | substance misuse |
| 355550012 | Pregnancy and drug dependence | substance misuse |
| 2548950012 | Preoccupied with substance misuse | substance misuse |
| 476311000000116 | Previous history of amphetamine misuse | substance misuse |
| 477271000000118 | Previous history of cocaine misuse | substance misuse |
| 477511000000114 | Previous history of crack cocaine misuse | substance misuse |
| 475531000000111 | Previous history of heroin misuse | substance misuse |
| 477031000000112 | Previous history of opiate misuse | substance misuse |
| 478471000000116 | Previous history of solvent misuse | substance misuse |
| 2548750019 | Prolonged high dose use of cannabis | substance misuse |
| 199931000006119 | Psychostimulant abuse | substance misuse |
| 411458014 | Psychostimulant dependence | substance misuse |
| 1926131000006117 | Refer to MH services deferred until alcohol misuse resolved | substance misuse |
| 1484890014 | Referral to community alcohol team | substance misuse |
| 1746481000000112 | Referral to community alcohol team declined | substance misuse |
| 2549646014 | Referral to community drug and alcohol team | substance misuse |
| 189631000000117 | Referral to community drug dependency team | substance misuse |
| 1780455014 | Referral to drug abuse counsellor | substance misuse |
| 494891000000118 | Referral to drugs therapist | substance misuse |
| 622961000000116 | Referral to specialist alcohol treatment service | substance misuse |
| 540591000000111 | Referral to substance misuse service | substance misuse |
| 495611000000112 | Reinduction to methadone maintenance therapy | substance misuse |
| 2264491000000115 | Seen in drug misuse clinic | substance misuse |
| 1063801000000119 | Seen in substance misuse clinic | substance misuse |
| 540681000000110 | Self referral to substance misuse service | substance misuse |
| 1153451000000115 | Shared care drug misuse treatment | substance misuse |
| 1704461000006111 | Shared care drug misuse treatment - enhanced services admin | substance misuse |
| 342475010 | Shares drug equipment | substance misuse |
| 342444018 | Smokes drugs in cigarette form | substance misuse |
| 342445017 | Smokes drugs through a pipe | substance misuse |
| 342447013 | Sniffs drugs | substance misuse |
| 127611000006118 | Stimulant abuse | substance misuse |
| 127621000006114 | Stimulant dependence | substance misuse |
| 1627491000006112 | Substance misuse | substance misuse |
| 2533726015 | Substance misuse clinical management plan agreed | substance misuse |
| 300081000000111 | Substance misuse clinical management plan reviewed | substance misuse |
| 305121000000119 | Substance misuse management stopped - self withdrawal | substance misuse |
| 2533634017 | Substance misuse monitoring | substance misuse |
| 2307771000000112 | Substance misuse monitoring 6 month review | substance misuse |
| 531051000000115 | Substance misuse structured counselling | substance misuse |
| 530931000000116 | Substance misuse treatment declined | substance misuse |
| 305731000000118 | Substance misuse treatment programme completed | substance misuse |
| 305701000000112 | Substance misuse treatment withdrawn | substance misuse |
| 1907701000006117 | Substnce misse treatmnt pgme del other healththcare provider | substance misuse |
| 117761000006116 | Suspect alcohol abuse - denied | substance misuse |
| 253490011 | Suspected abuse hard drugs | substance misuse |
| 253489019 | Suspected abuse soft drugs | substance misuse |
| 253488010 | Suspected drug abuse | substance misuse |
| 295136019 | Unspecified chronic alcoholism | substance misuse |
| 295153014 | Unspecified opioid dependence | substance misuse |
| 512651000000113 | Uses heroin on top of substitution therapy | substance misuse |
| 501294012 | Wernicke-Korsakov syndrome | substance misuse |
| 317676016 | [D]Finding of psychotropic drug in blood | substance misuse |
| 461112017 | [V]Alcohol abuse counselling and surveillance | substance misuse |
| 461113010 | [V]Drug abuse counselling and surveillance | substance misuse |
| 460276013 | [V]Personal history of alcoholism | substance misuse |
| 2474734017 | [V]Personal history of drug abuse by injection | substance misuse |
| 460277016 | [V]Personal history of psychoactive substance abuse | substance misuse |
| 361941000006116 | [X]Accidental poisoning with heroin | substance misuse |
| 362921000006118 | [X]Alcohol addiction | substance misuse |
| 315091000000117 | [X]Alcohol withdrawal-induced seizure | substance misuse |
| 362941000006113 | [X]Alcoholic dementia NOS | substance misuse |
| 362951000006110 | [X]Alcoholic hallucinosis | substance misuse |
| 362961000006112 | [X]Alcoholic jealousy | substance misuse |
| 362971000006117 | [X]Alcoholic paranoia | substance misuse |
| 362981000006119 | [X]Alcoholic psychosis NOS | substance misuse |
| 370961000006119 | [X]Chronic alcoholic brain syndrome | substance misuse |
| 370971000006114 | [X]Chronic alcoholism | substance misuse |
| 371181000006118 | [X]Cold turkey, opiate withdrawal | substance misuse |
| 376451000006113 | [X]Delirium tremens, alcohol induced | substance misuse |
| 379251000006119 | [X]Drug addiction - cannabis | substance misuse |
| 379261000006117 | [X]Drug addiction - cocaine | substance misuse |
| 379271000006112 | [X]Drug addiction - hallucinogen | substance misuse |
| 379281000006110 | [X]Drug addiction - opioids | substance misuse |
| 295213015 | [X]Drug addiction - solvent | substance misuse |
| 379301000006114 | [X]Drug addiction NOS | substance misuse |
| 379311000006112 | [X]Drug addiction- sedative / hypnotics | substance misuse |
| 379321000006116 | [X]Drug addiction-other stimul | substance misuse |
| 380031000006116 | [X]Evid of alcohol involv determind by level of intoxication | substance misuse |
| 388831000006113 | [X]Heroin addiction | substance misuse |
| 394681000006119 | [X]Korsakov's psychosis, alcohol induced | substance misuse |
| 397141000006111 | [X]Men & beh dis due cocaine: resid & late-onset psychot dis | substance misuse |
| 397161000006110 | [X]Men & beh dis due opioids: resid & late-onset psychot dis | substance misuse |
| 397191000006119 | [X]Men & beh dis vol solvents: withdrawal state wth delirium | substance misuse |
| 397201000006116 | [X]Men & behav dis due alcoh: resid & late-onset psychot dis | substance misuse |
| 397211000006118 | [X]Men & behav dis due alcohl: withdrawl state with delirium | substance misuse |
| 397231000006112 | [X]Men & behav dis due opioid: withdrawl state with delirium | substance misuse |
| 397241000006119 | [X]Men & behav dis due to use alcohol: oth men & behav dis | substance misuse |
| 397261000006115 | [X]Men & behav dis due to use opioids: oth men & behav dis | substance misuse |
| 397291000006111 | [X]Men & behav disorder multiple drug use/psychoactive subst | substance misuse |
| 397301000006112 | [X]Men/beh dis mlt drg use/oth psy sbs: oth men & behav dis | substance misuse |
| 397311000006110 | [X]Men/beh dis mlt drg use/oth subs: resid/late psychot dis | substance misuse |
| 397321000006119 | [X]Men/behav dis due to use cannabinoids: oth men/behav disd | substance misuse |
| 397431000006111 | [X]Ment & behav dis due use alcohol: unsp ment & behav dis | substance misuse |
| 397451000006116 | [X]Ment & behav dis due use opioids: unsp ment & behav dis | substance misuse |
| 980181000006113 | [X]Ment behav dis due crack cocaine: unsp ment and behav dis | substance misuse |
| 397471000006114 | [X]Ment/beh dis multi drug use/oth psy sbs unsp mnt/beh dis | substance misuse |
| 397481000006112 | [X]Ment/beh dis oth stims inc caffeine: unsp ment/behav disd | substance misuse |
| 397501000006119 | [X]Ment/behav dis due use cannabinoids: unsp ment/behav disd | substance misuse |
| 397511000006116 | [X]Ment/behav dis due use hallucinogens: unsp ment/behav dis | substance misuse |
| 397531000006110 | [X]Ment/behav dis due use vol solvents: unsp ment/behav dis | substance misuse |
| 397541000006117 | [X]Ment/behav dis mlti drug use/oth psyc sbs: psychotc dis | substance misuse |
| 397581000006111 | [X]Mental & behav dis due to cannabinoids: psychotic disordr | substance misuse |
| 397591000006114 | [X]Mental & behav dis due to hallucinogens: psychotic disord | substance misuse |
| 397601000006118 | [X]Mental & behav dis due to seds/hypntcs: psychotic disordr | substance misuse |
| 397611000006115 | [X]Mental & behav dis due to use alcohol: acute intoxication | substance misuse |
| 397621000006111 | [X]Mental & behav dis due to use alcohol: psychotic disorder | substance misuse |
| 397641000006116 | [X]Mental & behav dis due to use cocaine: psychotic disorder | substance misuse |
| 397661000006117 | [X]Mental & behav dis due to use opioids: psychotic disorder | substance misuse |
| 397691000006113 | [X]Mental & behav dis due to vol solvents: psychotic disordr | substance misuse |
| 397711000006111 | [X]Mental & behav disorder due other stimulants inc caffein | substance misuse |
| 397721000006115 | [X]Mental & behav disorders due to use of volatile solvents | substance misuse |
| 397731000006117 | [X]Mental and behav dis due cannabinoids: withdrawal state | substance misuse |
| 397741000006110 | [X]Mental and behav dis due hallucinogens: withdrawal state | substance misuse |
| 397751000006112 | [X]Mental and behav dis due seds/hypntcs: withdrawal state | substance misuse |
| 397761000006114 | [X]Mental and behav dis due to cannabinoids: dependence synd | substance misuse |
| 397771000006119 | [X]Mental and behav dis due to hallucinogens: dependence syn | substance misuse |
| 397781000006116 | [X]Mental and behav dis due to seds/hypntcs: dependence synd | substance misuse |
| 397791000006118 | [X]Mental and behav dis due to use alcohol: amnesic syndrome | substance misuse |
| 397801000006117 | [X]Mental and behav dis due to use alcohol: dependence syndr | substance misuse |
| 397811000006119 | [X]Mental and behav dis due to use alcohol: withdrawal state | substance misuse |
| 397821000006110 | [X]Mental and behav dis due to use cannabinoids: amnesic syn | substance misuse |
| 397831000006113 | [X]Mental and behav dis due to use cannabinoids: harmful use | substance misuse |
| 397851000006118 | [X]Mental and behav dis due to use cocaine: dependence syndr | substance misuse |
| 397861000006116 | [X]Mental and behav dis due to use cocaine: withdrawal state | substance misuse |
| 397871000006111 | [X]Mental and behav dis due to use hallucinogens: harmfl use | substance misuse |
| 397881000006114 | [X]Mental and behav dis due to use of alcohol: harmful use | substance misuse |
| 397891000006112 | [X]Mental and behav dis due to use of cocaine: harmful use | substance misuse |
| 397901000006111 | [X]Mental and behav dis due to use of opioids: harmful use | substance misuse |
| 397921000006118 | [X]Mental and behav dis due to use opioids: amnesic syndrome | substance misuse |
| 397931000006115 | [X]Mental and behav dis due to use opioids: dependence syndr | substance misuse |
| 397941000006113 | [X]Mental and behav dis due to use opioids: withdrawal state | substance misuse |
| 397961000006112 | [X]Mental and behav dis due to use seds/hypntcs: harmful use | substance misuse |
| 398011000006112 | [X]Mental and behav dis due to vol solvents: dependence synd | substance misuse |
| 398041000006111 | [X]Mental and behav dis due volatile solvents: harmful use | substance misuse |
| 398051000006113 | [X]Mental and behav dis mlti drg/oth psychoa sbs: harmfl use | substance misuse |
| 398061000006110 | [X]Mental and behav dis mlti/oth psych sbs: dependence syndr | substance misuse |
| 398071000006115 | [X]Mental and behav dis mlti/oth psychoa sbs: withdrwl state | substance misuse |
| 398081000006117 | [X]Mental and behav dis oth stim inc caffein: dependnce synd | substance misuse |
| 398111000006111 | [X]Mental and behavioural dis due use sedatives/hypnotics | substance misuse |
| 398141000006110 | [X]Mental and behavioural disorders due to psychoactive subs | substance misuse |
| 398151000006112 | [X]Mental and behavioural disorders due to use cannabinoids | substance misuse |
| 398161000006114 | [X]Mental and behavioural disorders due to use hallucinogens | substance misuse |
| 295765017 | [X]Mental and behavioural disorders due to use of alcohol | substance misuse |
| 295877018 | [X]Mental and behavioural disorders due to use of cocaine | substance misuse |
| 295803018 | [X]Mental and behavioural disorders due to use of opioids | substance misuse |
| 980081000006112 | [X]Mental and behavioural disorders due use of crack cocaine | substance misuse |
| 980141000006119 | [X]Mental behav disord due crack cocaine: psychotic disorder | substance misuse |
| 980121000006114 | [X]Mental behav disord due crack cocaine: withdrawal state | substance misuse |
| 980111000006118 | [X]Mental behav disorders due use crack cocaine: depend synd | substance misuse |
| 980101000006116 | [X]Mental behav disorders due use crack cocaine: harmful use | substance misuse |
| 398261000006119 | [X]Mental/behav dis multi drg use/oth psy sbs: amnesic syndr | substance misuse |
| 398691000006112 | [X]Mnt/bh dis due cannabinds: resid & late-onset psychot dis | substance misuse |
| 398701000006112 | [X]Mnt/bh dis due hallucngns: resid & late-onset psychot dis | substance misuse |
| 398721000006119 | [X]Mnt/bh dis mlti drg use/oth psy sbs: wthdr state + dlrium | substance misuse |
| 423031000006112 | [X]Post hallucinogen perception disorder | substance misuse |

Table S3 : ICD-10 codes and categories used for identifying psychiatric illnesses in HES.

| Category | ICD-10 code |
| --- | --- |
| sleep disorders | G47 |
| sleep disorders | F51 |
| sleep disorders | Z72.82 |
| sleep disorders | G25.81 |
| anxiety&neurosis | F40 |
| anxiety&neurosis | F41 |
| anxiety&neurosis | F42 |
| anxiety&neurosis | F43 |
| anxiety&neurosis | F44 |
| anxiety&neurosis | F45 |
| anxiety&neurosis | F46 |
| anxiety&neurosis | F47 |
| anxiety&neurosis | F48 |
| personality disorders | F60 |
| personality disorders | F61 |
| personality disorders | F62 |
| personality disorders | F63 |
| personality disorders | F68 |
| personality disorders | F69 |
| affective disorders | F30 |
| affective disorders | F31 |
| affective disorders | F32 |
| affective disorders | F33 |
| affective disorders | F34 |
| affective disorders | F35 |
| affective disorders | F36 |
| affective disorders | F37 |
| affective disorders | F38 |
| affective disorders | F39 |
| affective disorders | F53.0 |
| schizophrenia spectrum | F20 |
| schizophrenia spectrum | F21 |
| schizophrenia spectrum | F22 |
| schizophrenia spectrum | F23 |
| schizophrenia spectrum | F24 |
| schizophrenia spectrum | F25 |
| schizophrenia spectrum | F28 |
| schizophrenia spectrum | F29 |
| substance misuse | F10 |
| substance misuse | F11 |
| substance misuse | F12 |
| substance misuse | F13 |
| substance misuse | F14 |
| substance misuse | F15 |
| substance misuse | F16 |
| substance misuse | F17 |
| substance misuse | F18 |
| substance misuse | F19 |
| eating disorders | F50 |
