# Supplementary material for: Evaluation of common prescription analgesics and adjuvant analgesics as markers of suicide risk: a longitudinal population-based study in England
Source: Lancet Reg Health Eur. 2023 Jul 20;32:100695. doi: 10.1016/j.lanepe.2023.100695 (PMC10393825; doi:10.1016/j.lanepe.2023.100695)
Supplement: Supplementary Information 3 [file mmc3.docx]

## Supplementary information 3

Post-hoc analysis: Suicide by poisoning vs non-poisoning across different time periods

A post-hoc analysis was conducted to estimate the odds ratios of suicide by poisoning (using any substance) versus non-poisoning across two time periods: from 2001 to 2016 and from 2016 to 2019. International Classification of Diseases 10th Revision (ICD-10) codes in the Office for National Statistics were used to identify poisoning suicide (and hence segregate them from non-poisoning suicide) as the cause of death. Those ICD-10 codes were X60 to X69 (self-poisoning) and Y10 to Y19 (poisoning of undetermined intent). Odds ratios adjusted for sex and age at index/suicide date were estimated using conditional logistic regression. Age was fitted in the regression model into four quantiles. Figure 1 below depicts the results.

Figure 1: Odds ratios of suicide by poisoning (by any substance) versus non-poisoning across different time periods.

Error bars represent the 95% confidence intervals.
