# Supplementary material for: Evaluation of common prescription analgesics and adjuvant analgesics as markers of suicide risk: a longitudinal population-based study in England
Source: Lancet Reg Health Eur. 2023 Jul 20;32:100695. doi: 10.1016/j.lanepe.2023.100695 (PMC10393825; doi:10.1016/j.lanepe.2023.100695)
Supplement: Supplementary Information 4 [file mmc4.docx]

## Supplementary information 4

**Sensitivity analysis**

Two sensitivity analysis were conducted. In the first sensitivity analysis (Table S1), we excluded all patients in any database (Clinical Practice Research Datalink or Hospital Episode Statistics inpatient records) with a code for epilepsy, bipolar mood disorder diagnosis and/or generalized anxiety disorder. In our sample, there were 7493 patients with a record of epilepsy, 2427 patients with a record of bipolar mood disorder and 1979 patients with a record of generalized anxiety disorder. After excluding patients with epilepsy, bipolar mood disorder, and generalized anxiety disorder there were 583096 patients left in the dataset for sensitivity analysis. All odds ratios pertaining to prescriptions of adjuvant analgesics were adjusted for the effect of age, gender and psychiatric diagnosis.

Table S1: Odds ratios for suicide pertaining to patients prescribed adjuvant analgesics, relative to those not prescribed analgesics, in whole sample versus in a sample excluding patients with epilepsy, bipolar mood disorder and generalized anxiety disorder.

|  | OR (95% CI) ^a^  *whole sample*  *N=*594674 | OR (95% CI) ^a^  *Sensitivity Analysis 1**  *N=583096* |
| --- | --- | --- |
| Adjuvant analgesics  All  Pregabalin  Gabapentin  Carbamazepine | 2.10 (1.86-2.37)  3.12 (2.57-3.78)  1.64 (1.36-1.99)  1.74 (1.39-2.18) | 2.01 (1.73-2.34)  2.75 (2.19-3.46)  1.58 (1.27-1.96)  1.82 (1.27-2.59)) |
| Number of different analgesic categories**  0 categories  1 category  2 categories  3 categories  P value for linearity | 1  1.08 (1.00-1.15)  1.24 (1.14-1.36)  2.02 (1.66-2.47)  <0.001 | 1  1.05 (0.97-1.133)  1.18 (1.07-1.30)  1.89 (1.50-2.39)  <0.001 |
| Adjuvant analgesics  15-35  35-55  +55 | 2.12 (1.23-3.66)  2.47 (2.01-3.04)  1.96 (1.59-2.41) | 2.50 (1.25-5.00)  2.28 (1.77-2.95)  1.92 (1.52-2.45) |

a multivariable model adjusted for gender and age (at suicide death date for cases and index date for controls) and psychiatric diagnoses for main effects, and adjusted for gender and psychiatric illnesses across age-strata.

* In a sample excluding patients with bipolar mood disorder, epilepsy and generalized anxity disorder.

**The three main categories for analgesics were opioid analgesics, non-opioid analgesics and adjuvant analgesics.

In the second sensitivity analysis (Table S2), we added amitriptyline to the adjuvant analgesic category and compared the results to when amitriptyline was excluded (the original sample). Odds ratios were adjusted for the effect of age, gender and psychiatric diagnosis (including depressive disorders). There were 8434 patients prescribed amitriptyline in the sample (1.42% of the sample), of whom 452 died of suicide (5.36% of those prescribed amitriptyline).

Table S2: Comparison of odds ratios for suicide pertaining to patients prescribed adjuvant analgesics (without and with amitriptyline), relative to those not prescribed analgesics.

|  | OR (95% CI) ^a^  *N=*594674  *Original analysis where amitriptyline was* ***not*** *included to adjuvant analgesic category* | OR (95% CI) ^a^  *N=*594674  *Sensitivity analysis 2 where amitriptyline was included to adjuvant analgesic category* |
| --- | --- | --- |
| Adjuvant analgesics  All  Amitriptyline alone | 2.10 (1.86-2.37)  - | 1.82 (1.67-1.99)  1.62 (1.46-1.80) |
| Number of different analgesic categories**  0 categories  1 category  2 categories  3 categories  P value for linearity | 1  1.08 (1.00-1.15)  1.24 (1.14-1.36)  2.02 (1.66-2.47)  <0.001 | 1  1.15 (1.08-1.23)  1.27 (1.16-1.39)  2.08 (1.70-2.54)  <0.001 |
| Adjuvant analgesics  15-35  35-55  +55 | 2.12 (1.23-3.66)  2.47 (2.01-3.04)  1.96 (1.59-2.41) | 1.94 (1.41-2.68)  2.05 (1.77-2.37)  1.70 (1.48-1.96) |
